# Detection of IMP-4 and SFO-1 co-producing ST51 *Enterobacter hormaechei* clinical isolates

**DOI:** 10.3389/fcimb.2022.998578

**Published:** 2022-10-27

**Authors:** Jie Qiao, Haoyu Ge, Hao Xu, Xiaobing Guo, Ruishan Liu, Chenyu Li, Ruyan Chen, Beiwen Zheng, Jianjun Gou

**Affiliations:** ^1^ Department of Laboratory Medicine, The First Affiliated Hospital of Zhengzhou University, Zhengzhou, China; ^2^ Collaborative Innovation Center for Diagnosis and Treatment of Infectious Diseases, State Key Laboratory for Diagnosis and Treatment of Infectious Diseases, The First Affiliated Hospital, College of Medicine, Zhejiang University, Hangzhou, China; ^3^ Department of Structure and Morphology, Jinan Microecological Biomedicine Shandong Laboratory, Jinan, Shandong, China; ^4^ Research Units of Infectious Diseases and Microecology, Chinese Academy of Medical Sciences, Beijing, China

**Keywords:** *Enterobacter hormaechei*, ST51, *bla*_IMP-4_, *bla*_SFO-1_, IncN, multidrug-resistant, genomics

## Abstract

**Purpose:**

To explore the genetic characteristics of the IMP-4 and SFO-1 co-producing multidrug-resistant (MDR) clinical isolates, *Enterobacter hormaechei* YQ13422hy and YQ13530hy.

**Methods:**

MALDI-TOF MS was used for species identification. Antibiotic resistance genes (ARGs) were tested by PCR and Sanger sequencing analysis. In addition to agar dilution, broth microdilution was used for antimicrobial susceptibility testing (AST). Whole-genome sequencing (WGS) analysis was conducted using the Illumina NovaSeq 6000 and Oxford Nanopore platforms. Annotation was performed by RAST on the genome. The phylogenetic tree was achieved using kSNP3.0. Plasmid characterization was conducted using S1-pulsed-field gel electrophoresis (S1-PFGE), Southern blotting, conjugation experiments, and whole genome sequencing (WGS). An in-depth study of the conjugation module was conducted using the OriTFinder website. The genetic context of *bla*_IMP-4_ and *bla*_SFO-1_ was analyzed using BLAST Ring Image Generator (BRIG) and Easyfig 2.3.

**Results:**

YQ13422hy and YQ13530hy, two MDR strains of ST51 *E. hormaechei* harboring *bla*_IMP-4_ and *bla*_SFO-1_, were identified. They were only sensitive to meropenem, amikacin and polymyxin B, and were resistant to cephalosporins, aztreonam, piperacillin/tazobactam and aminoglycosides, intermediate to imipenem. The genetic context surrounding *bla*_IMP-4_ was 5′CS-*hin-1*-IS*26*-In*tI1*-*bla*_IMP-4_-IS*6100*-*ecoRII*. The integron of *bla*_IMP-4_ is In*823*, which is the array of gene cassettes of 5′CS-*bla*_IMP-4_. Phylogenetic analysis demonstrated that *E. hormaechei* YQ13422hy and YQ13530hy belonged to the same small clusters with a high degree of homology.

**Conclusion:**

This observation revealed the dissemination of the *bla*_IMP-4_ gene in *E. hormaechei* in China. We found that *bla*_IMP-4_ and *bla*_SFO-1_ co-exist in MDR clinical *E. hormaechei* isolates. This work showed a transferable IncN-type plasmid carrying the *bla*_IMP-4_ resistance gene in *E. hormaechei*. We examined the potential resistance mechanisms of pYQ13422-IMP-4 and pYQ13422-SFO-1, along with their detailed genetic contexts.

## Introduction

*Enterobacter cloacae* complex (ECC) is the most common group of species among the genus *Enterobacter*, including six closely related species: *E. cloacae*, *E. asburiae*, *E. hormaechei*, *E. kobei*, *E. ludwigii*, and *E. nimipressuralis* (Mezzatesta et al., 2012). *Enterobacter hormaechei* can be widely found in different environments such as the nature (Osei Sekyere and Reta, 2021), feces of humans or animals. But it is also an important pathogenic bacteria in hospitals, which can be responsible for nosocomial infections, such as wounds, urinary tract, and soft tissue infections (Xu et al., 2015). The horizontal spread of bacterial resistance genes, especially the carbapenemase-encoding gene, has brought great difficulties to clinical treatment (Annavajhala et al., 2019).

Since *bla*_IMP-1_ was firstly declared in Japan in 1991 (Watanabe et al., 1991), IMP- producing ECC has been playing an increasingly significant role in the world antibiotic resistance stage, like Malaysia (Liew et al., 2018), Portugal (Goncalves et al., 2021), and Korea (Lee et al., 2017). As time progressed, more and more IMP variants appeared in China, including IMP-2 (Riccio et al., 2000), IMP-8 (Yan et al., 2001), IMP-4 (Chu et al., 2001), IMP-26 (Gou et al., 2020). In China, IMP-4-positive carbapenemase-producing *Enterobacterales* (CPE) have become important carbapenem-resistant bacteria (Hu et al., 2014), since it was first discovered in Hongkong (Chu et al., 2001) in 2001. The *bla*_IMP-4_ is mainly found in *Pseudomonas aeruginosa*, but has been gradually reported in Enterobacteriaceae (Matsumura et al., 2017), such as *E. hormaechei* (Chen et al., 2022). More importantly, the coincidence of *bla*_IMP_ and other antibiotic resistance genes is becoming increasingly common, such as co-carrying *bla*_IMP-4_ and *bla*_NDM-1_ (Zhang et al., 2021a), further increasing the tremendous pressure of clinical treatment.

In 1999, a clinical *E. cloacae* 8009 isolate possessing a transferable plasmid harboring *bla*_SFO-1_ was reported in Japan (Matsumoto and Inoue, 1999). The reports of *bla*_SFO-1_ and coexisting antibiotic resistance genes have recently increased in China (Zhou et al., 2020). In comparison with other broad-spectrum-beta-lactamases, the *bla*_SFO-1_ gene has a low occurrence of antimicrobial resistance that has been ignored by routine monitoring. We found a carbapenem-resistant *E. hormaechei* clinical isolate co-harboring *bla*_SFO-1_ and *bla*_IMP-4_.

There are few studies on the transmission of *bla*_SFO-1_ and *bla*_IMP-4_ in ECC in China, especially *E. hormaechei.* Therefore, it is vital to further explore the genome and phenotypic characteristics of the *bla*_SFO-1_ and *bla*_IMP-4_ in *E. hormaechei* in China. We identified clinical isolates of *E. hormaechei* YQ13422hy and YQ13530hy co-producing *bla*_IMP-4_ and *bla*_SFO-1_, and described the detailed content of a conjugative IncN-plasmid. Furthermore, we revealed the underlying transmission mechanisms of *bla*_IMP-4_.

## Materials and methods

### Bacterial strains

We continuously collected ECC clinical isolates from a tertiary hospital affiliated to Wenzhou Medical University from 2015 to 2017 for routine surveillance. A total of eight carbapenemase producing ECC clinical isolates have been identified using the MALDI-TOF MS (Bruker, Bremen, Germany). Among them, the two isolates of IMP-4-producing *E. hormaechei* strains (25%) were identified using PCR and next-generation sequencing (NGS), designated as YQ13422hy and YQ13530hy.

### Multilocus sequence typing and antimicrobial susceptibility testing

As described previously, multilocus sequence typing (MLST) was conducted (Gou et al., 2020). A new sequence type has been submitted to MLST and have been approved by PubMLST (http://pubmlst.org/ecloacae). Agar dilution and broth microdilution were used for antimicrobial susceptibility testing (AST), and *Escherichia coli* ATCC 25922 was used as control. AST results were interpreted based on the Clinical and Laboratory Standards Institute (CLSI) 2021 standards, while tigecycline and colistin clinical breakpoints were based on the 2022 EUCAST (http://www.eucast.org). Sixteen antimicrobial resistance genes were searched using PCR, including *bla*_KPC_, *bla*_NDM_, *bla*_IMP_, *bla*_OXA-23_, *bla*_OXA-48_, *bla*_VIM_, and *mcr-*1-10.

### Plasmid characterization and conjugation assays

Pulsed-field gel electrophoresis (PFGE) was used to determine the homology between strains YQ13422hy and YQ13530hy. PFGE was undertaken on the CHEF-DR III system (Bio-Rad. Hercules, CA, United States), and patterns were evaluated and interpreted according to the published guidelines (Xu et al., 2018). The profiles of plasmids in strains YQ13422hy and YQ13530hy were analyzed by the S1-PFGE, as previously described (Wang et al., 2019). Then we used a digoxigenin-labeled *bla*_IMP-4_ probe made by a dig-high prime DNA Labeling and Detection Starter Kit II (Roche Diagnostics) to determine the location of plasmid harboring *bla*_IMP-4_ *via* southern blotting and hybridization. The transferability of plasmids was investigated by using *E. coli* J53, a NaN_3_-resistant standard strain, as a receptor for conjugation assays. Subsequently, transconjugants carrying *bla*_IMP-4_ were first selected using Mueller-Hinton agar (OXOID, Hampshire, United Kingdom) plates containing both 1 mg/L meropenem and 200 mg/L NaN_3_. Further, the selected conjugates were confirmed by MALDI-TOF/MS, PCR identified the *bla*_IMP-4_ gene, and AST was used to confirm the expression of drug resistance genes.

### Whole genome sequencing and *in silico* analyses

Genomic DNA was extracted using a Genomic DNA Isolation Kit (QIAGEN, Hilden, Germany) and sequenced using Illumina Novaseq 6000 (Illumina, San Diego, CA, United States) and Oxford Nanopore platforms (Oxford Nanopore Technologies, Oxford, United Kingdom). RAST 2.0 was used to annotate the draft genomes obtained by SPAdes version 3.9.1 (Aziz et al., 2008) (http://rast.nmpdr.org/). ISfinder and INTEGRALL were used to detect insertion sequence elements and integrons (https://www-is.biotoul.fr/). Antimicrobial resistance genes (ARGs) were identified by Resfinder (https://cge.cbs.dtu.dk/services/ResFinder/). Different plasmid genome sequences were compared by BLAST Ring Image Generator (Alikhan et al., 2011) (BRIG). The figures about the genetic context surrounding the antibiotic resistance genes were drawn by Easyfig 2.3 (Sullivan et al., 2011). To verify whether the plasmids pYQ13422-IMP-4, pYQ13530-IMP-4, pYQ13422-SFO-1 and pYQ13530-SFO-1 were conjugative plasmids, we used the OriTFinder website (https://tool-mml.sjtu.edu.cn/oriTfinder/oriTfinder.html).

### Phylogenetic analysis

We downloaded all available IMP-carrying ECC from the NCBI genome database in May 2022 to study the phylogenetic relationships of YQ13422hy and YQ13530hy with other ECC. KSNP3.0 (Gardner et al., 2015) was used to construct the phylogenetic tree based on the previously-mentioned downloaded data *via* SNPS. ITOL was used to visualize and modify the phylogenetic tree (https://itol.embl.de/).

## Results

### Species confirmation and homology analysis

The YQ13422hy strain was isolated from a sputum specimen of a 36-year-old male suffering from hypoxic encephalopathy on March 12, 2017. YQ13530hy was isolated from a sputum specimen of a 60-year-old male with brain herniation on April 2, 2017. The patient carrying YQ13422hy was hospitalized for 3 months, from March 01, 2017 to June 10, 2022. The patient carrying YQ13530hy was hospitalized for 1 month from April 01, 2017 to April 19, 2022. Patient carrying YQ13422hy was treated with intravenous vancomycin, Imipenem and Cilastatin Sodium, as well as Cefoperazone Sodium and Sulbactam Sodium. Patient carrying YQ13530hy was treated with vancomycin, meropenem and levofloxacin. Both patients were hospitalized in the same ward. ANI analysis (**Figure S2** and **Table S2**) and WGS showed that the two isolates were highly homologous, and the *bla*_IMP-4_-bearing plasmids had 99.97% similarity, indicating the isolates’ clonal spread. In fact, it is not clear how the clonal spread happened, but we suspect that it may have been through contact or through the air, because both strains were detected in sputum.

### AST of *Enterobacter hormaechei* YQ13422hy and YQ13530hy

The isolates YQ13422hy and YQ13530hy both displayed resistance to aztreonam, ceftriaxone, cefotaxime, ceftazidime, levofloxacin, ciprofloxacin, gentamicin, piperacillin/tazobactam, chloromycin, amoxicillin-clavulanate, cefepime, with sensitivity to meropenem, amikacin, and polymyxin B. For imipenem, YQ13422hy and YQ13530hy were determined as intermediate. In the case of YQ13422hy, it exhibited intermediate resistance to fosfomycin, and susceptibility to trimethoprim/sulfamethoxazole and tigecycline. On the other hand, YQ13530hy showed resistance to trimethoprim/sulfamethoxazole and tigecycline, while it was susceptible to fosfomycin. AST results revealed that both strains were MDR *E. hormaechei*. The results of AST of *E. hormaechei* YQ13422hy and YQ13530hy are shown in **Table 1**.

**Table 1:** MIC values of antimicrobials for *E. hormaechei* YQ13422hy andYQ13530hy, recipient strain J53, transconjugants YQ13422hy-J53 and YQ13530hy-J53, and control strain *E. coli* 25922.

Antimicrobials	MIC values (mg/L)					
	YQ13422hy	YQ13422hy-J53	YQ13530hy	YQ13530hy-J53	J53	25922
Aztreonam	>128 (R)	0.5 (S)	128 (R)	0.5 (S)	0.5 (S)	0.5 (S)
Imipenem	2 (I)	2 (I)	2 (I)	2 (I)	0.5 (S)	0.25 (S)
Meropenem	1 (S)	1 (S)	1 (S)	1 (S)	0.03 (S)	0.03 (S)
Ceftriaxone	>128 (R)	128 (R)	>128 (R)	128 (R)	0.06 (S)	0.06 (S)
Cefotaxime	>128 (R)	128 (R)	>128 (R)	128 (R)	0.125 (S)	0.125 (S)
Ceftazidime	>128 (R)	>128 (R)	>128 (R)	>128 (R)	0.25 (S)	0.5 (S)
Levofloxacin	4 (R)	1 (I)	8 (R)	1 (I)	0.015 (S)	0.03 (S)
Ciprofloxacin	2 (R)	1 (R)	8 (R)	1 (R)	0.03 (S)	0.015 (S)
Amikacin	16 (S)	16 (S)	16 (S)	16 (S)	16 (S)	16 (S)
Gentamicin	>128 (R)	4 (S)	>128 (R)	4 (S)	4 (S)	4 (S)
Piperacillin/Tazobactam	>128/4 (R)	16/4 (S)	128/4 (R)	16/4 (S)	4/1 (S)	4/1 (S)
Fosfomycin	128 (I)	0.5 (S)	64 (S)	0.5 (S)	0.25 (S)	0.5 (S)
Chloromycin	>128 (R)	4 (S)	64 (R)	4 (S)	4 (S)	4 (S)
Trimethoprim/Sulfamethoxazole	0.5/9.5 (S)	0.125/2.375 (S)	4/76 (R)	0.125/2.375 (S)	0.125/2.375 (S)	0.125/2.375 (S)
Amoxicillin-Clavulanic acid	128/64 (R)	128/64 (R)	128/64 (R)	128/64 (R)	4/2 (S)	8/4 (S)
Cefepime	32 (R)	16 (R)	32 (R)	16 (R)	0.06 (S)	0.06 (S)
Tigecycline	0.5 (S)	0.25 (S)	8 (R)	0.25 (S)	0.5 (S)	0.25 (S)
Polymyxin B	1 (S)	1 (S)	1 (S)	0.5 (S)	1 (S)	1 (S)

R, resistant; S, susceptible; I, intermediate.

### Location of *bla*_IMP-4_ and *bla*_SFO-1_ and the conjugation assays

S1-PFGE and hybridization experiments on YQ13422hy and YQ13530hy (**Figure S1**) showed that the plasmid harboring the *blaIMP-4* resistance gene was about 53 kb and it was named pYQ13422-IMP-4. The plasmid carrying *bla*_SFO-1_ resistance gene was designated as pYQ13422-SFO-1.

**Figure 1:**
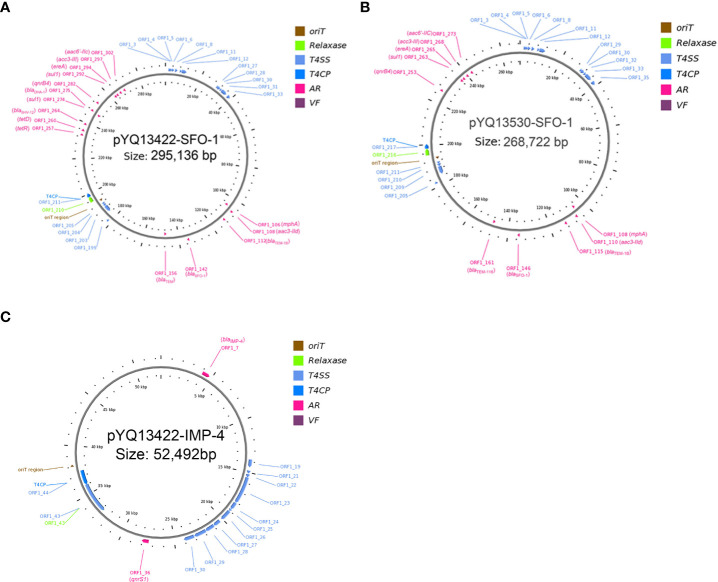
Three conjugative plasmids pYQ13422-SFO-1 **(A)** and pYQ13530-SFO-1 **(B)** and pYQ13422-IMP-4 **(C)**. AR (ARGs), acquired antibiotic resistance determinant genes; VF, virulence factors.

The transconjugant was identified as *E. coli* by MALDI-TOF/MS. Then PCR and Sanger sequencing were performed to determine that the transconjugant was carrying the *bla*_IMP-4_ resistance gene. The results of AST also indicated that the plasmid pYQ13422-IMP-4 was successfully transferred into recipient J53. A comparison of AST results between YQ13422hy and YQ13422-J53, YQ13530hy and YQ13530-J53 showed that the transconjugant was resistant to ceftriaxone, cefotaxime, ciprofloxacin, ceftazidime, amoxicillin-clavulanic acid and cefepime, sensitive to aztreonam, gentamicin, piperacillin/tazobactam, chloromycin and fosfomycin, and intermediate to levofloxacin and imipenem, but significantly increased the MIC value of the transconjugant to levofloxacin. Through the analysis by the OriTFinder website, the complete conjugative modules on the plasmid pYQ13422-IMP-4, pYQ13530-IMP-4, pYQ13422-SFO-1, and pYQ13530-SFO-1 were identified, including the origin of transfer site (*oriT*), gene cluster for bacterial type IV secretion system (T4SS), gene encoding type IV coupling protein (T4CP), and relaxase gene (**Table S4**). Based on these results, it appears they are MDR plasmids that can be horizontal transferred (**Figure 1**). Because pYQ13422-IMP-4 and pYQ13530-IMP-4 are exactly the same, we only show pYQ13422-IMP-4 in **Figure 1**.

### Characterization of the genome of *E. hormaechei* YQ13422hy and YQ13530hy

The result of S1-PFGE showed that YQ13422hy and YQ13530hy both carried three plasmids of different sizes, as mentioned above. WGS showed that YQ13422hy and YQ13530hy both carried four plasmids of different sizes. The plasmid pYQ13422hy-3 and Pyq13530hy-3 are not visible in the S1-PFGE result due to its small size; 4995bp.

According to the WGS results, YQ13422hy and YQ13530hy were shown by MLST to carry the genes *fusA* (4), *leuS* (6), *rplB* (4), *rpoB* (6), *dnaA* (4), *gyrB* (4), *pyrG* (37), confirming its typing as ST51. Specific genome information on plasmid sizes, Inc and MLST typing and resistance genes is displayed in **Table 2**.

**Table 2:** Genome information and acquired antibiotic resistance genes of *E. hormaechei* YQ13422hy and YQ13530hy.

Genome	Size (bp)	G + C (%)	Typing	Resistance gene
YQ13422hy			
Chromosome	4,570,859	55.72%	ST51	*fosA, bla*_ACT-7_
Plasmids				
pYQ13422hy-SFO-1	295,136	47.62%	IncHI2/2A	*aac(6’)-IIc, aac(3)-IId, ere(A), mph(A), qnrB4, bla*_SHV-12_ *, bla*_DHA-1_, *bla*_TEM-1B_, *bla*_SFO-1_, *sul1, tet(D), qacE, catA2*
pYQ13422hy-2	60,348	42.47%	undefined	
pYQ13422hy-IMP-4	52,492	50.85%	IncN	*qnrS1, bla*_IMP-4_
pYQ13422-3	4,995bp	51.73%	undefined	/
YQ13530hy				
Chromosome	4,571,686	55.73%	ST51	*fosA, bla*_ACT-7_
Plasmids				
pYQ13530hy-SFO-1	268,722	46.72%	IncHI2/2A	*aac(6’)-IIc, aac(3)-IId, ere(A),mph(A), qnrB4, sul1, bla*_SFO-1_, *bla*_TEM-1B_, *qacE*
pYQ13530hy-2	60,311	42.45%	undefined	/
pYQ13530hy-IMP-4	52,492	50.85%	IncN	*qnrS1, bla*_IMP-4_
pYQ13530hy-3	4,995bp	51.73%	undefined	/

### Structural characterization of the transferable plasmid

**Figure 2:**
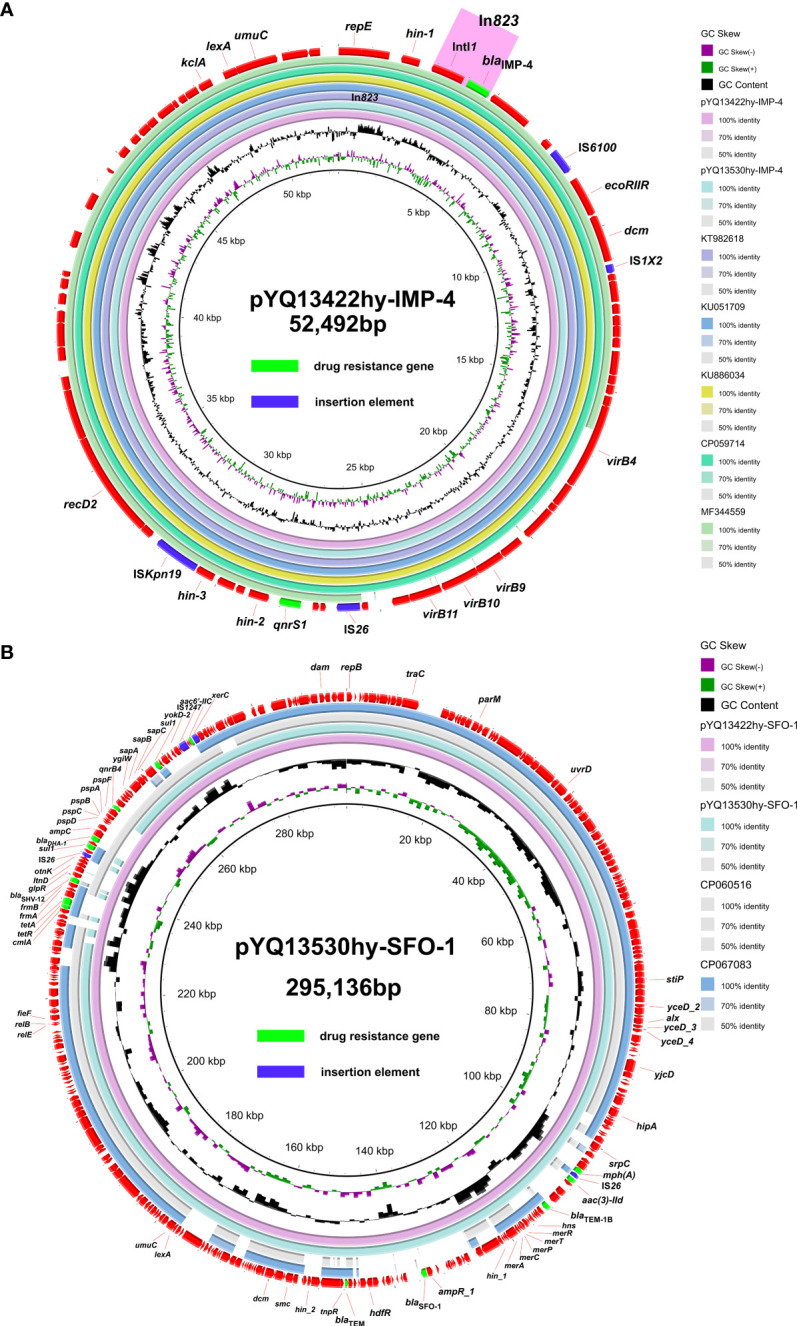
Genomic analyses of plasmid pYQ13422-IMP-4 **(A)** and pYQ13422-SFO-1 **(B)**. The comparative plasmid circular map of pYQ13422-IMP-4 and pYQ13422-SFO-1, generated using BLAST Ring Image Generator (BRIG), shows the genes and their locations.

The sequence length of plasmid pYQ13422-IMP-4 is 52,492bp, including 92 protein-encoding genes, and its G + C content is 50.85%. This plasmid carries the *bla*_IMP-4_ gene and the *qnrS1* gene, which is known from above. Its plasmid type is IncN by Plasmidfinder. The most similar plasmids (with 100% coverage and 99% identities) identified by NCBI blast are as follows: pIMP-GZ1517 (KT982618.1), pZHH-3 (CP059714), p128379-IMP (MF344559) and pIMP-GZ1058 (KU051709.1) from *E. coli*, and pIMP-HZ1 (KU886034) from *K. pneumoniae*. BLAST, Ring Image Generator (BRIG) generated the circular image of multiple plasmid comparisons, and the results were demonstrated in **Figure 2**. The plasmids carry multiple insertion sequences at different positions, such as IS*6100*, IS*1X2*, IS*26* and IS*Kpn19*. Further, we investigated the genetic environment of the IMP-4 resistance gene and found that it has an In*tI1* upstream and also carries a group II intron reverse transcriptase/maturase gene downstream of it. Comparison with pIMP-GZ1517 (KT982618.1) and pIMP-GZ1058 (KU051709.1) revealed that an insertion sequence IS*26* was missing on the YQ13422-IMP-4 plasmid (**Figure S5**). Integron In*823* was identified by INTEGRALL, whose array of gene cassettes is 5′CS-*bla*_IMP-4_. YQ13422-SFO-1 is demonstrated in **Figure 2B**.

In addition to analyzing MDR plasmid characteristics, we also examined mobile elements flanking the resistant genes (**Figure S5**). The *bla*_SFO−1_ was detected on a Tn*3* unit (Tn*AS3*-IS*5075*-*traX*-*Δ*Tn*3*-*ampR*-*bla*_SFO-1_-*Δ*IS*3*). According to the genetic mapping of *bla*_SFO-1_, *ampR* was upstream of *bla*_SFO−1_. Regulation of SFO-1 is carried out by the regulator *ampR*, which is inversely oriented upstream (Fernandez et al., 2011). Tn*3* and IS*5075* were located upstream of *ampR*, and genetic mapping also showed that the transposon Tn*3* was interrupted.

### Phylogenetic analysis

We downloaded all genomic data of the *bla*_IMP_-carrying ECC isolates (n = 167) from NCBI publicly available data and performed a phylogenetic analysis with YQ13422hy and YQ13530hy (**Table S1**). The data showed that the vast majority of bacteria carrying the *bla*_IMP_ resistance genes in the ECC are *E. hormaechei*, with 154 strains accounting for 91.12% of all strains. The results revealed that the *bla*_IMP_ resistance genes carried by the ECC were *bla*_IMP-1_ (n = 66), *bla*_IMP-4_ (n = 77), *bla*_IMP-8_ (n = 8), *bla*_IMP-13_ (n = 2), *bla*_IMP-16_ (n = 1), *bla*_IMP-26_ (n = 3), and *bla*_IMP-70_ (n = 12). Of these, 158 strains were isolated from humans, and only 11 strains had no host information. The majority of isolates were from Japan, China and Australia. The source of these strains is almost exclusively clinical, mainly blood, urine, sputum, and screening swab. YQ13422hy and YQ13530hy form a small cluster alone, and a larger cluster with GCA_015684015, GCA_021165665, GCA_015683815, but GCA_015684015 and GCA_015683815 are isolated from Australia, while GCA_021165665.1 is recovered from Ireland. They are both *E. hormaechei* and carry the drug resistance gene *bla*_IMP-4_. More specific information is shown in **Figure S6**.

## Discussion

ECC is increasingly being isolated from clinical specimens and is now one of the world’s most critical nosocomial infectious pathogens (Bolourchi et al., 2022). The ECC carrying *bla*_IMP_ has emerged in six countries, including United Kingdom, the United States, Ireland, Japan, China, and Australia. Thus, the prevalence of the *bla*_IMP_ gene worldwide should be given sufficient attention.

According to our susceptibility results, strains carrying *bla*_IMP-4_ are intermediate to imipenem and sensitive to meropenem. There is evidence suggesting that IMP-4 enzyme has much stronger hydrolytic activity for imipenem than meropenem, which is consistent with previous findings (Chu et al., 2001). A considerable amount of literature now exists suggesting that multiple different species of bacteria carrying *bla*_IMP-4_ are intermediate or sensitive to imipenem and meropenem (Chu et al., 2001; Lee et al., 2017; Tarabai et al., 2021; Zhang et al., 2021b). However, the exact mechanism is still unclear. Intermediate susceptibility to imipenem and susceptibility to meropenem in strains carrying *bla*_IMP-4_ possible mechanism could be: i) related to the activity of efflux pumps (Zhang et al., 2021b), or (ii) It is possibly that the organisms had little or no expression of their *bla*_IMP-4_ gene (Chu et al., 2001), or (iii) It seems that IMP enzymes confer carbapenem resistance only in members of the family Enterobacteriaceae with concomitant permeability lesions (Chu et al., 2001).

Plasmids play a major role in the dissemination of antibiotic resistance genes among Enterobacteriaceae (Huang et al., 2013). Although there have been many studies on IncN-type plasmids, few studies have found that IncN-type plasmids carrying the IMP-4 resistance gene in *E. hormaechei.* IncN-type plasmids carrying genes such as *bla*_KPC_ (Gomez-Simmonds et al., 2022) and *bla*_NDM_ (Hirabayashi et al., 2021) have been found in *E. coli* (Dorr et al., 2022) and *Citrobacter* (Yao et al., 2021). Also, a lot of IncN *bla*_IMP-4_-carrying plasmids were described in *Enterobacterales*, including one study showing the isolation of *Klebsiella pneumoniae* carrying an IncN-type plasmid with *bla*_KPC-2_ from dogs (Sellera et al., 2021). Wang and colleagues have already reported that an IncN ST7 plasmid carrying *bla*_IMP-4_ is disseminated in a variety of enterobacterial species originating from patients with epidemiological links in remote areas of China (Wang et al., 2017). The plasmids carrying the *bla*_IMP-4_ gene of YQ13422hy and YQ13530hy are entirely identical. Besides, it’s worth noting that we collected these two bacteria from different patients in the same ward at different times in the same hospital. In addition, based on the INTEGRALL database, *bla*_IMP-4_ is located on a class 1 integron In*823*, which is rare in *E. hormaechei*, with the array of gene cassettes 5′CS-*bla*_IMP-4_. It has become a consensus that the proliferation of integrons has exacerbated the prevalence of drug-resistant genes, especially class 1 integrons (Souque et al., 2021). The 3′CS of most class I integrons include three open reading frames (ORFs): sulfa resistance gene (*sul1*), quaternary ammonium compound and ethidium bromide tolerance gene (*qacEΔ1*) and an ORF of unknown function. However, unlike the classical class 1 integron, the 3’CS of the class 1 integron of YQ13422-IMP-4, *sul1* and *qacEΔ1* was not found.

Meanwhile, we confirmed the presence of a transposon Tn*AS3* carrying the *bla*_SFO-1_ gene, which belongs to the transposon family Tn*3*. Studies on the Tn*3* family of transposons have been relatively extensive. Previous studies indicate that the most characteristic resolvases of the Tn*3* transposon family are members of the serine recombinase (S recombinase) family, but rarely are members of the tyrosine recombinase (Y recombinase) family (Nicolas et al., 2015). The plasmid YQ13422hy-SFO-1 carries tyrosine recombinase *xerC*. Meanwhile, through our study on the structure of the YQ13422-IMP-4 plasmid and comparative analysis with other plasmids, we found that the *bla*_IMP-4_ genes all contain a group II intron reverse transcriptase/maturase downstream, and speculated that this gene might be associated with the transfer and spread of *bla*_IMP-4_. Compared with plasmids pIMP-GZ1517 (KT982618) and pIMP-GZ1517 (KU051709), pYQ13422-IMP-4 and pZHH-3 (CP059714) have no insert sequence IS*26*, which suggests that IntI*1* can transfer *bla*_IMP-4_ independently and IS*26* may not be the critical gene for *bla*_IMP-4_ gene transfer. In pIMP-GZ1517 (KT982618) and pIMP-GZ1517 (KU051709), the IntI*1* gene was interrupted by an IS*26* element, but *bla*_IMP-4_ could still be transferred. We believed that the truncated IntI*1* was out of its function, and the transfer was achieved by IS26. We also found that p128379-IMP does not have the integrase IntI*1*, but contains IS*26*. We discovered the entire complete conjugative modules on the plasmids pYQ13422-IMP-4, pYQ13422-SFO-1 and pYQ13530-SFO-1.

The studies on *bla*_IMP-4_ in *E. hormaechei* are rare worldwide, with significant differences between countries. A prospective cohort study (Roberts et al., 2020a) in Australia showed that the primary ST type of ECC carrying *bla*_IMP-4_ was ST90, and the plasmid carrying *bla*_IMP-4_ was IncHI2-type. Currently, *bla*_IMP-4_ is Australia’s most common resistance gene (Sidjabat et al., 2015), and our phylogenetic analysis based on published data from NCBI confirmed this. Another study (Roberts et al., 2020b) also supported a similar view. Furthermore, we found that all the integrons of *E. hormaechei* carrying *bla*_IMP-4_ in the published studies rarely contain In*823*. This further indicates that the context of the *bla*_IMP-4_ gene may be different in China.

In China, few reports described the detection of *bla*_IMP-4_ gene in *E. hormaechei* (Chen et al., 2022). Kai Zhou et al. found a strain of *E. hormaechei* of ST418 carrying *bla*_NDM-1_, *mcr-9.1*, and *bla*_IMP-4_ (Zhou et al., 2017). According to its research, the plasmid carrying *bla*_IMP-4_ was IncHI2-type, which is consistent with the global trend. The *bla*_SFO-1_ gene is not routinely monitored, but it could be an important weapon against antibiotics. So, the coexistence of *bla*_SFO-1_ and other antibiotic resistance genes should not be ignored. A previous study reported the co-producing of SFO-1 and IMP-4 in *Klebsiella pneumoniae* clinical isolate (Zhou et al., 2017). Moreover, in our work, we not only found the co-producing of SFO-1 and IMP-4 in *E. hormaechei*, but also found they are located at two different transferable plasmids. Antibiotic resistance may be increased by the presence of *bla*_SFO-1_. The research of AST results on IMP-producing ECC (Hickey et al., 2021) also suggested that IMP metalloenzymes production in ECC infections is serious, and our work also validated the study. *E. hormaechei* carrying the *bla*_IMP-4_ gene spread rapidly, with enhanced drug resistance and changes in the genetic environment. Therefore, the coexistence of *bla*_SFO-1_ and *bla*_IMP-4_ undoubtedly complicates the treatment of *E. hormaechei* infections. The limitation of our work is that only two samples were studied, and there were no more samples to further elaborate on the prevalence of IncN-plasmid carrying IMP-4 in *E. hormaechei*.

## Conclusion

Our study found the co-production of IMP-4 and SFO-1 in *E. hormaechei.* Besides, it revealed the IncN-type plasmid carrying *bla*_IMP-4_ in *E. hormaechei*, which indicated the potential horizontal transformation of ARGs. In conclusion, our work supplemented the studies of *E. hormaechei* carrying *bla*_IMP-4_ and *bla*_SFO-1_ in China, and also suggested that focusing on *E. hormaechei* will be important in future studies.

## Data availabilitiy statement

The datasets presented in this study can be found in online repositories. The names of the repository/repositories and accession number(s) can be found in the article/**Supplementary Material**.

## Ethics statement

Written informed consent was obtained from the individual(s) for the publication of any potentially identifiable images or data included in this article.

## Author contributions

The experiments were conceived and designed by JG and BZ. The samples and experiments were collected and performed by JQ, HG, RL, CL and RC. The data was analyzed by HX and XG. The manuscript was written by JQ. All authors contributed to the article and approved the submitted version.

## Funding

This work was supported by research grants from Henan Science and Technology Department (No. 192102310059), the National Natural Science Foundation of China (82072314), the Research Project of Jinan Microecological Biomedicine Shandong Laboratory (JNL-2022011B), the Fundamental Research Funds for the Central Universities (2022ZFJH003), CAMS Innovation Fund for Medical Sciences (2019-I2M-5-045), and Henan Province Medical Science and Technology Research Project Joint Construction Project (No. LHGJ20190232).

## Conflict of interest

The authors declare that the research was conducted in the absence of any commercial or financial relationships that could be construed as a potential conflict of interest.

## Publisher’s note

All claims expressed in this article are solely those of the authors and do not necessarily represent those of their affiliated organizations, or those of the publisher, the editors and the reviewers. Any product that may be evaluated in this article, or claim that may be made by its manufacturer, is not guaranteed or endorsed by the publisher.

## Supplementary material

Supplementary Figure 1 Plasmid profiles of **(E)** hormaechei YQ13422hyh and YQ13530hy. **(A)** Plasmid size determination by S1-PFGE, with Salmonella enterica serotype Braenderup H9812 as the size marker. **(B)** Southern blotting hybridization with an IMP-4-specific probe.

Supplementary Figure 2 The ANI analysis between YQ13422hy and YQ13530hy showed that there is a high level of similarity between the two genomes.

Supplementary Figure 3 Genomic analyses of plasmid pYQ13422-IMP-4. The comparative plasmid circular map of pYQ13422-IMP-4 generated using BLAST Ring Image Generator (BRIG), shows the genes and their locations.

Supplementary Figure 4 YQ13422hy and YQ13530hy ‘s genomes contain a wealth of information

Supplementary Figure 5 Genetic context of blaIMP-4 on pYQ13422-IMP-4 and blaSFO-1 on pYQ13422-SFO-1. Genes are denoted by arrows. Genes, mobile elements, and other features are colored based on their functional classification.

Supplementary Figure 6 The phylogenetic tree of 167 strains ECC based on the blaIMP resistance genes, generated by kSNP3.0 plus *E. hormaechei* YQ13422hy (Biosample SAMN28918927) and YQ13530hy (Biosample SAMN28919657). The sources of strains are identified as clinical. We used different colors to represent different meanings. The seven circles around the phylogenetic tree indicate the species (inner circle), ST type, IMP-type, host, location, year, and source (outer circle) of these strains. We marked YQ13422hy and YQ13530hy in red.
