# Supplementary figures and images for: Detection of IMP-4 and SFO-1 co-producing ST51 Enterobacter hormaechei clinical isolates
Source: Front Cell Infect Microbiol. 2022 Oct 27;12:998578. doi: 10.3389/fcimb.2022.998578 (PMC9647121; doi:10.3389/fcimb.2022.998578)

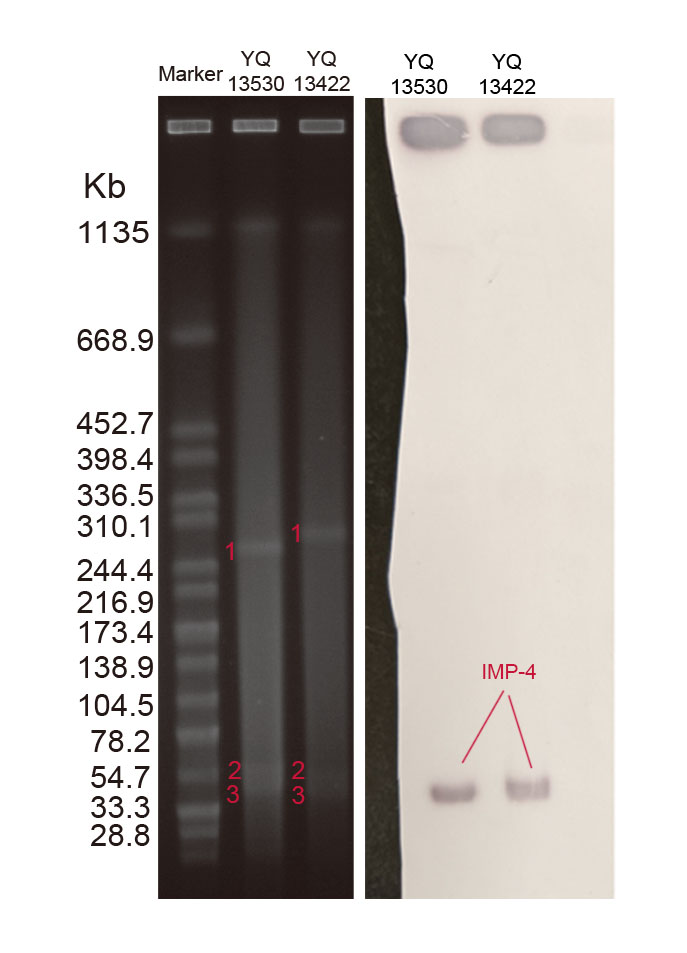

Supplement: Supplementary Figure 1 — Plasmid profiles of (E) hormaechei YQ13422hyh and YQ13530hy. (A) Plasmid size determination by S1-PFGE, with Salmonella enterica serotype Braenderup H9812 as the size marker. (B) Southern blotting hybridization with an IMP-4-specific probe. [file Image_1.jpeg]

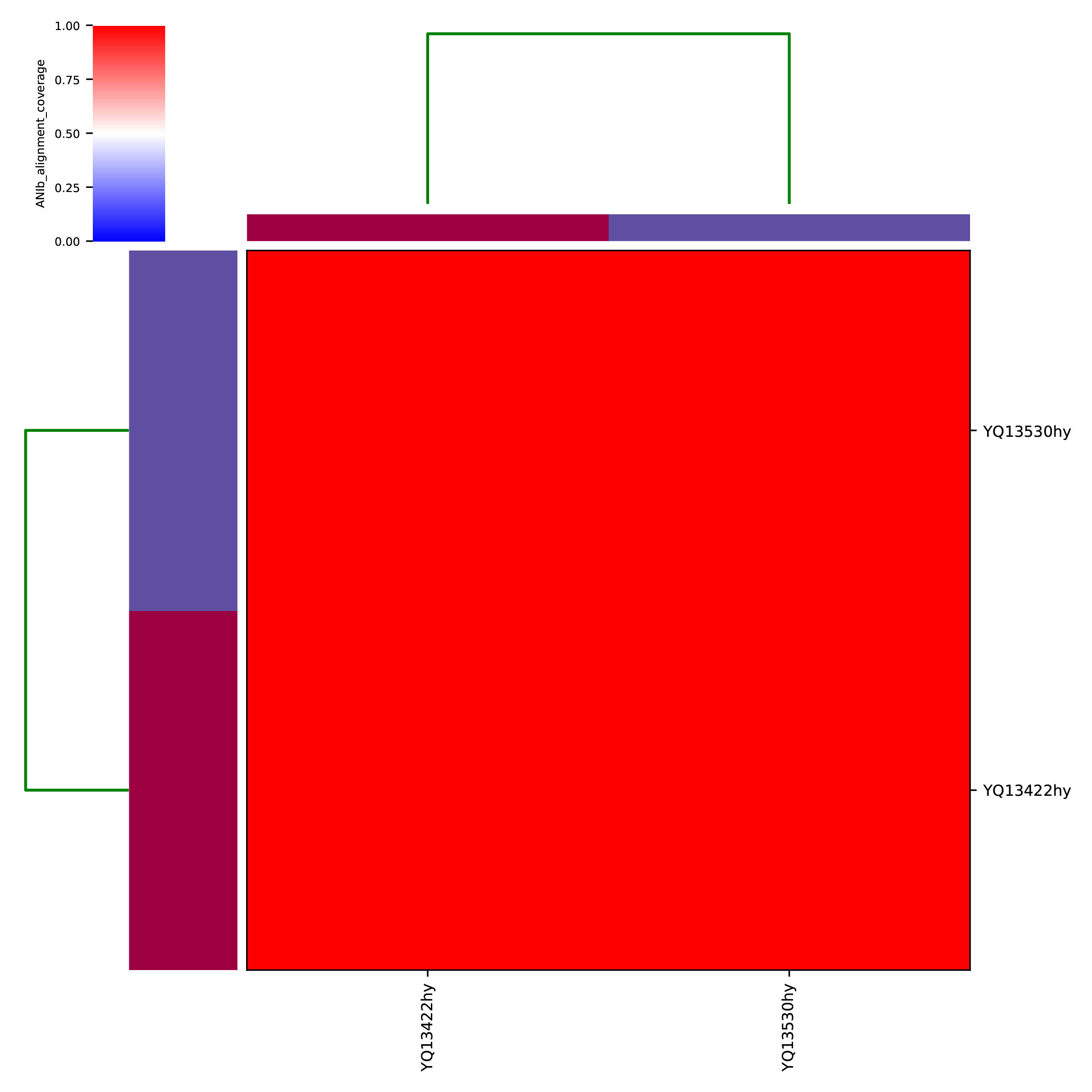

Supplement: Supplementary Figure 2 — The ANI analysis between YQ13422hy and YQ13530hy showed that there is a high level of similarity between the two genomes. [file Image_2.jpeg]

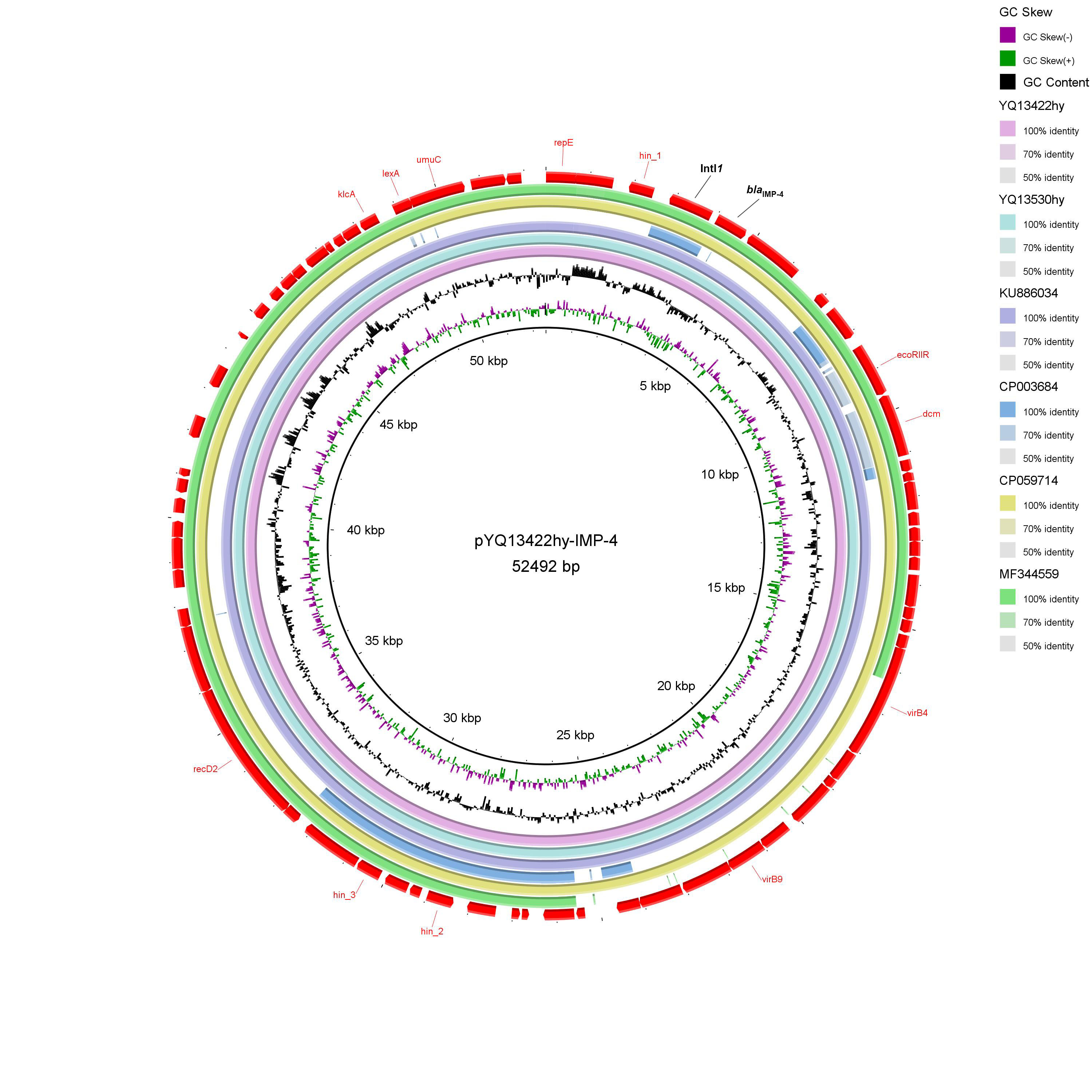

Supplement: Supplementary Figure 3 — Genomic analyses of plasmid pYQ13422-IMP-4. The comparative plasmid circular map of pYQ13422-IMP-4 generated using BLAST Ring Image Generator (BRIG), shows the genes and their locations. [file Image_3.jpeg]

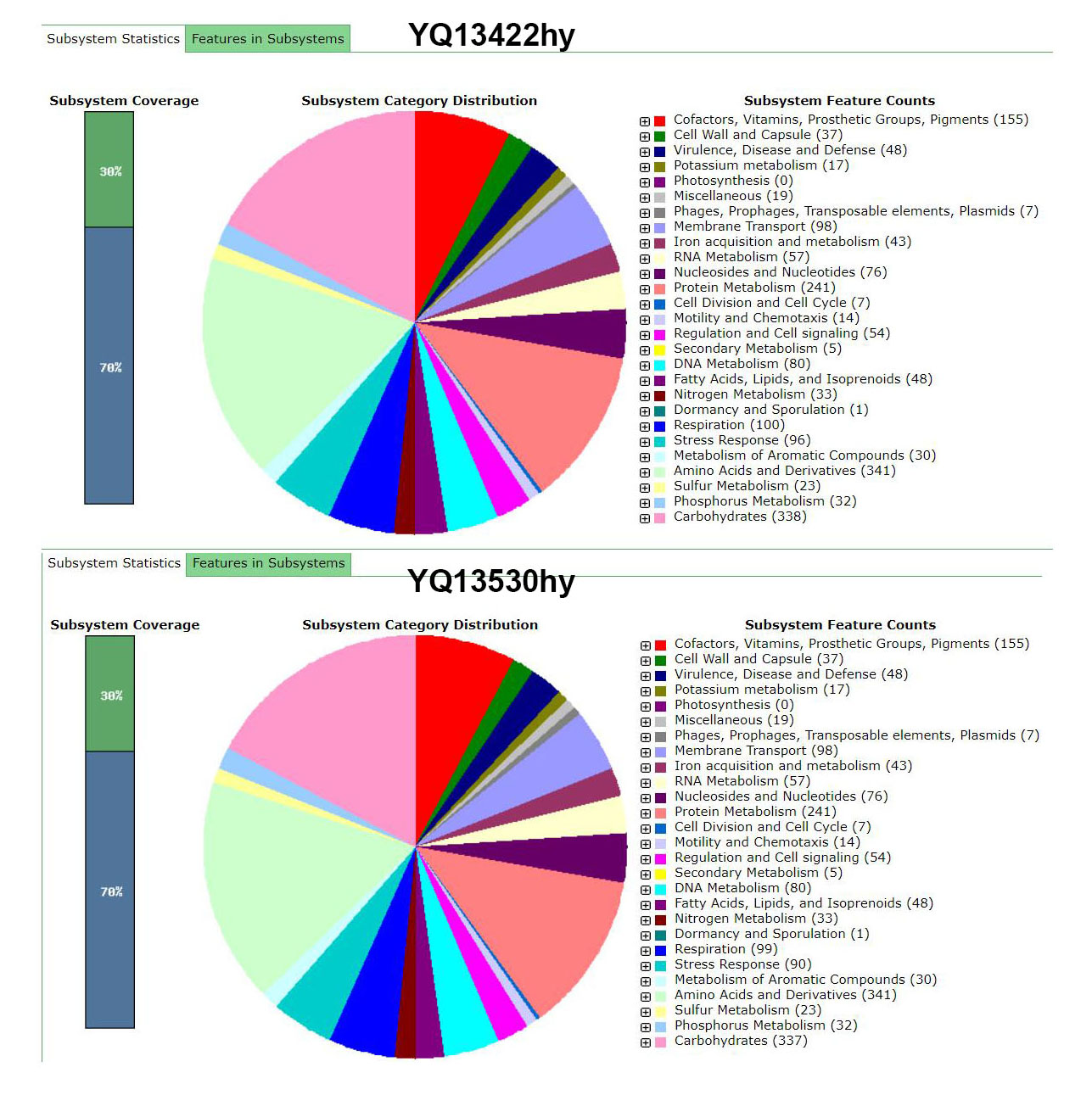

Supplement: Supplementary Figure 4 — YQ13422hy and YQ13530hy ‘s genomes contain a wealth of information [file Image_4.jpeg]

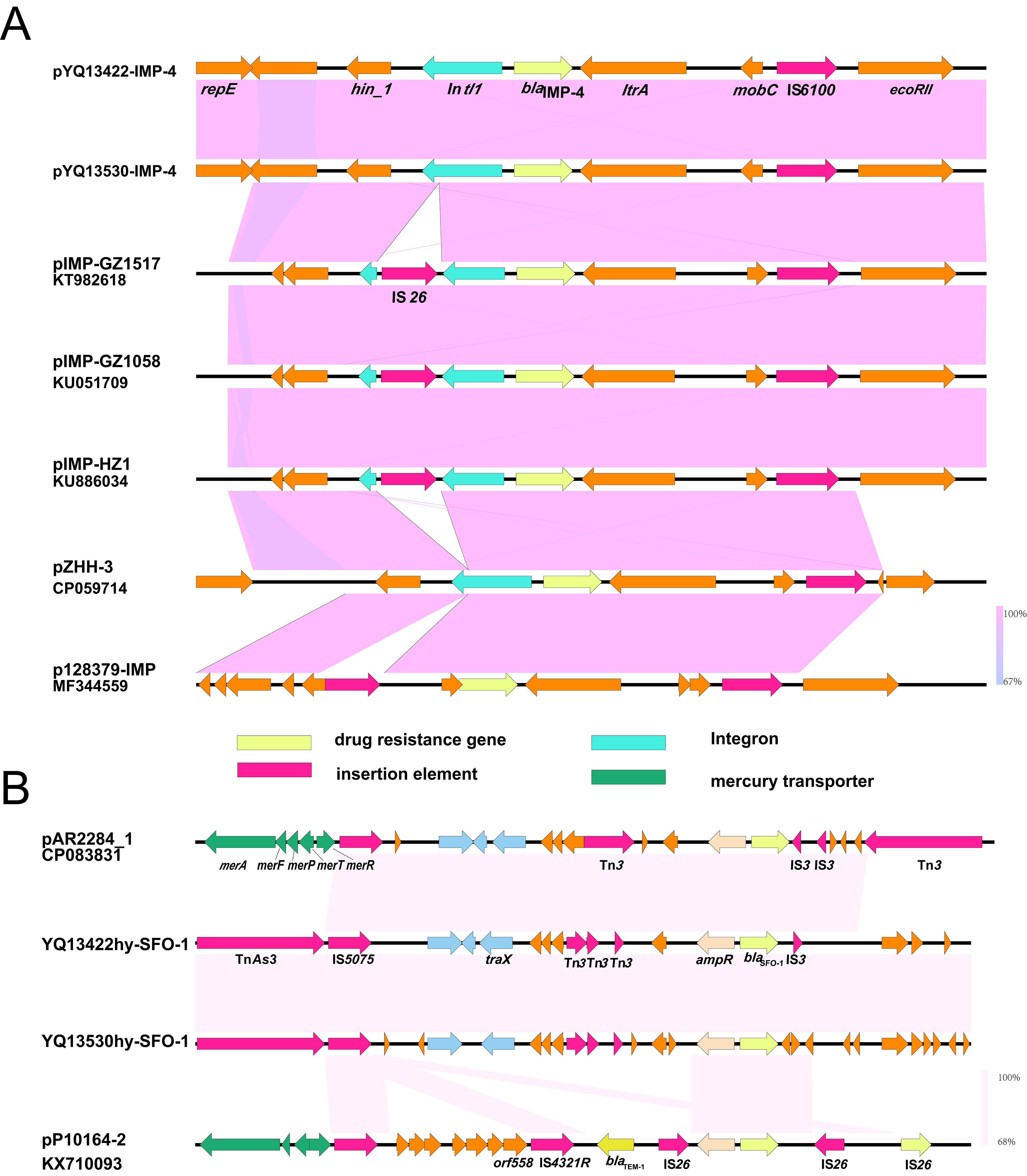

Supplement: Supplementary Figure 5 — Genetic context of blaIMP-4 on pYQ13422-IMP-4 and blaSFO-1 on pYQ13422-SFO-1. Genes are denoted by arrows. Genes, mobile elements, and other features are colored based on their functional classification. [file Image_5.jpeg]

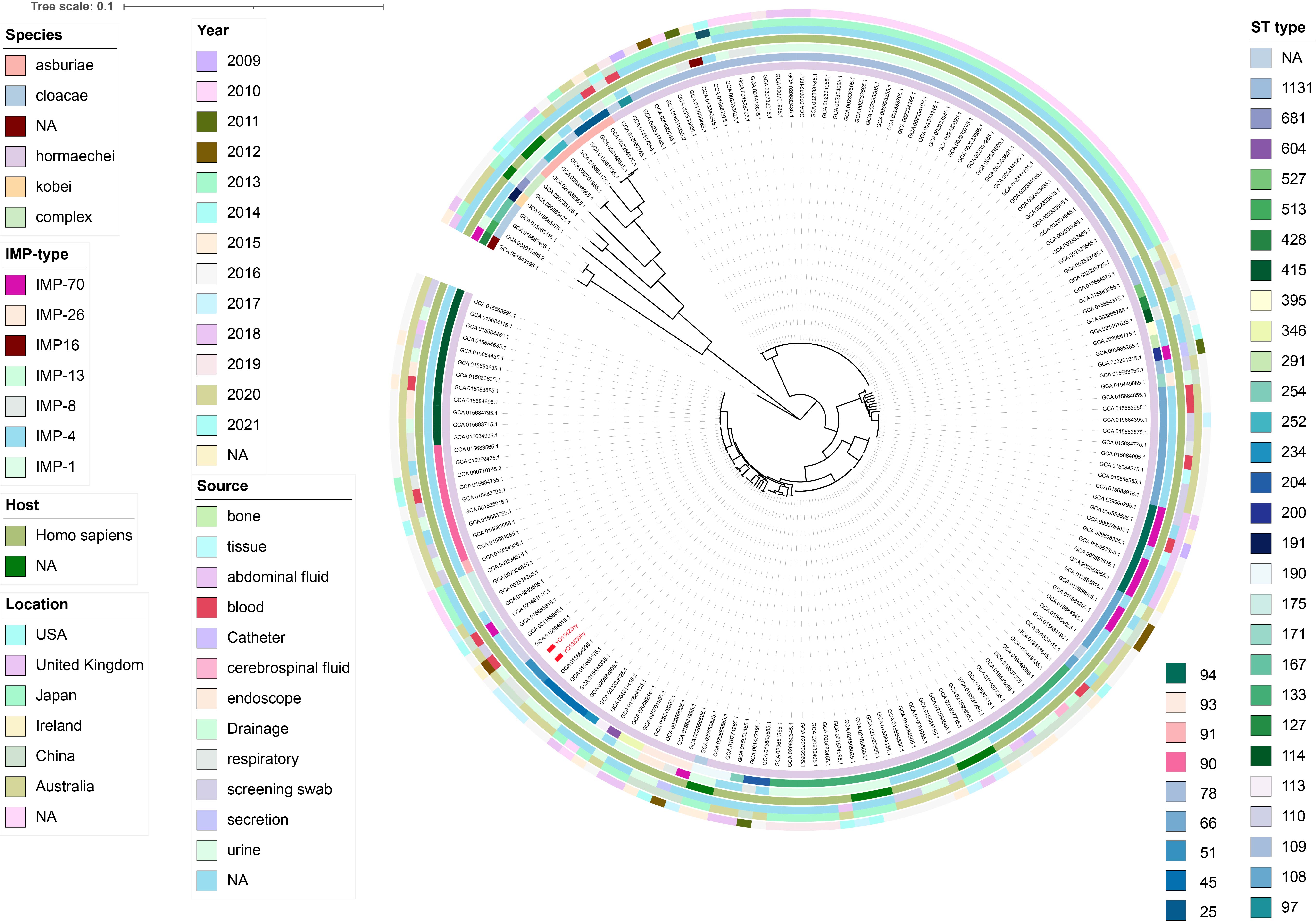

Supplement: Supplementary Figure 6 — The phylogenetic tree of 167 strains ECC based on the blaIMP resistance genes, generated by kSNP3.0 plus E. hormaechei YQ13422hy (Biosample SAMN28918927) and YQ13530hy (Biosample SAMN28919657). The sources of strains are identified as clinical. We used different colors to represent different meanings. The seven circles around the phylogenetic tree indicate the species (inner circle), ST type, IMP-type, host, location, year, and source (outer circle) of these strains. We marked YQ13422hy and YQ13530hy in red. [file Image_6.jpeg]
