# Supplementary material for: Detection of IMP-4 and SFO-1 co-producing ST51 Enterobacter hormaechei clinical isolates
Source: Front Cell Infect Microbiol. 2022 Oct 27;12:998578. doi: 10.3389/fcimb.2022.998578 (PMC9647121; doi:10.3389/fcimb.2022.998578)
Supplement: Supplementary file 8 [file Table_1.docx]

| GC number | BioSample ID | Collection date | Geo loc name | Host | resoure | IMP-type | species | ST | | | |  |  |  |
| --- | --- | --- | --- | --- | --- | --- | --- | --- | --- | --- | --- | --- | --- | --- |
| GCA_021543195.1 | SAMN24296918 | 31-Jul-15 | United Kingdom | Homo sapiens | NA | 70 | NA | 428 | | | |  |  |  |
| GCA_004011395.2 | SAMD00143517 | 13-Oct-18 | Japan:Nagoya | Homo sapiens | NA | 1 | cloacae | 513 | | | |  |  |  |
| GCA_015683495.1 | SAMN11230954 | 2016 | Australia: Brisbane | Homo sapiens | NA | 4 | cloacae | 167 | | | |  |  |  |
| GCA_015683115.1 | SAMN14867396 | 2016 | Australia: Brisbane | Homo sapiens | NA | 4 | cloacae | 167 | | | |  |  |  |
| GCA_015685475.1 | SAMN11230929 | 2016 | Australia: Brisbane | Homo sapiens | NA | 4 | kobei | 191 | | | |  |  |  |
| GCA_020889425.1 | SAMD00412797 | 16-Aug-16 | Japan | NA | NA | 1 | cloacae | 681 | | | |  |  |  |
| GCA_020733125.1 | SAMN22231266 | 2021 | USA | Homo sapiens | NA | 13 | cloacae | NA | | | |  |  |  |
| GCA_020889385.1 | SAMD00412821 | 20-Apr-17 | Japan | NA | NA | 1 | asburiae | NA | | | |  |  |  |
| GCA_020888965.1 | SAMD00412800 | 21-Apr-17 | Japan | NA | NA | 1 | asburiae | 252 | | | |  |  |  |
| GCA_020701955.1 | SAMN21893148 | 25-Jun-18 | Japan:Aichi | Homo sapiens | NA | 1 | asburiae | 252 | | | |  |  |  |
| GCA_015684175.1 | SAMN11230945 | 2016 | Australia: Brisbane | Homo sapiens | NA | 4 | asburiae | NA | | | |  |  |  |
| GCA_015681395.1 | SAMN07501532 | 2013 | Taiwan | Homo sapiens | NA | 8 | asburiae | 25 | | | |  |  |  |
| GCA_020149545.1 | SAMN21399027 | 24-Sep-20 | China:Yongchuan District, Chongqing | Homo sapiens | NA | 4 | asburiae | 25 | | | |  |  |  |
| GCA_002264125.1 | SAMN07345015 | 8-May-15 | China:Anhui | Homo sapiens, | blood | 8 | asburiae | 25 | | | |  |  |  |
| GCA_018067745.1 | SAMN18511070 | 2020 | USA | Homo sapiens, | Sputum, | 13 | hormaechei | NA | | | |  |  |  |
| GCA_014117285.1 | SAMN15648651 | 8-Aug-19 | China: Hangzhou | Homo sapiens | blood | 4 | hormaechei | 97 | | | |  |  |  |
| GCA_002334745.1 | SAMD00089518 | 25-Oct-09 | JAPAN | Homo sapiens | NA | 1 | hormaechei | 78 | | | |  |  |  |
| GCA_020682245.1 | SAMN21893168 | 28-Aug-19 | Japan:Aichi | Homo sapiens | NA | 1 | hormaechei | 78 | | | |  |  |  |
| GCA_004011355.2 | SAMD00143514 | 2012 | Japan:Nagoya | Homo sapiens | NA | 1 | hormaechei | 78 | | | |  |  |  |
| GCA_002333825.1 | SAMD00089472 | 12-Aug-10 | Tokyo, Japan | Homo sapiens | NA | 1 | hormaechei | 78 | | | |  |  |  |
| GCA_015685485.1 | SAMD00055766 | 2011 | Taiwan | Homo sapiens | NA | 8 | hormaechei | 78 | | | |  |  |  |
| GCA_013340545.1 | SAMD00229143 | 2015 | Toho University | Homo sapiens | NA | 16 | hormaechei | 78 | | | |  |  |  |
| GCA_015681375.1 | SAMN07501547 | 2014 | University of Calgary | Homo sapiens | urine | 4 | hormaechei | 78 | | | |  |  |  |
| GCA_002333525.1 | SAMD00089457 | 5-Jul-10 | Toho University | Homo sapiens | NA | 1 | hormaechei | 78 | | | |  |  |  |
| GCA_001526005.1 | SAMN04252894 | 2010 | Taiwan | Homo sapiens | NA | 8 | hormaechei | 78 | | | |  |  |  |
| GCA_001472005.1 | SAMN04252893 | 2010 | Taiwan | Homo sapiens | NA | 8 | hormaechei | 78 | | | |  |  |  |
| GCA_020702015.1 | SAMN21893165 | 20-Aug-19 | Japan:Aichi | Homo sapiens | NA | 1 | hormaechei | 78 | | | |  |  |  |
| GCA_020701995.1 | SAMN21893153 | 30-Aug-18 | Japan:Aichi | Homo sapiens | NA | 1 | hormaechei | 78 | | | |  |  |  |
| GCA_020682485.1 | SAMN21893137 | 23-Dec-18 | Japan:Aichi | Homo sapiens | NA | 1 | hormaechei | 78 | | | |  |  |  |
| GCA_020682185.1 | SAMN21893158 | 21-Nov-18 | Japan:Aichi | Homo sapiens | NA | 1 | hormaechei | 78 | | | |  |  |  |
| GCA_002333585.1 | SAMD00089460 | 9-Jul-10 | Tokyo, Japan | Homo sapiens | NA | 1 | hormaechei | 78 | | | |  |  |  |
| GCA_002334085.1 | SAMD00089485 | 24-Aug-10 | Tokyo, Japan | Homo sapiens | NA | 1 | hormaechei | 78 | | | |  |  |  |
| GCA_002334065.1 | SAMD00089484 | 24-Aug-10 | Tokyo, Japan | Homo sapiens | NA | 1 | hormaechei | 78 | | | |  |  |  |
| GCA_002333865.1 | SAMD00089474 | 17-Aug-10 | Tokyo, Japan | Homo sapiens | NA | 1 | hormaechei | 78 | | | |  |  |  |
| GCA_002333565.1 | SAMD00089459 | 7-Jul-10 | Tokyo, Japan | Homo sapiens | NA | 1 | hormaechei | 78 | | | |  |  |  |
| GCA_002333905.1 | SAMD00089476 | 18-Aug-10 | Tokyo, Japan | Homo sapiens | NA | 1 | hormaechei | 78 | | | |  |  |  |
| GCA_002923255.1 | SAMD00089491 | 11-Sep-10 | Tokyo, Japan | Homo sapiens | NA | 1 | hormaechei | 78 | | | |  |  |  |
| GCA_002333765.1 | SAMD00089469 | 10-Aug-10 | Tokyo, Japan | Homo sapiens | NA | 1 | hormaechei | 78 | | | |  |  |  |
| GCA_002334165.1 | SAMD00089489 | 7-Sep-10 | Tokyo, Japan | Homo sapiens | NA | 1 | hormaechei | 78 | | | |  |  |  |
| GCA_002334105.1 | SAMD00089486 | 30-Aug-10 | Tokyo, Japan | Homo sapiens | NA | 1 | hormaechei | 78 | | | |  |  |  |
| GCA_002334145.1 | SAMD00089488 | 1-Sep-10 | Tokyo, Japan | Homo sapiens | NA | 1 | hormaechei | 78 | | | |  |  |  |
| GCA_002333945.1 | SAMD00089478 | 19-Aug-10 | Tokyo, Japan | Homo sapiens | NA | 1 | hormaechei | 78 | | | |  |  |  |
| GCA_002333925.1 | SAMD00089477 | 19-Aug-10 | Tokyo, Japan | Homo sapiens | NA | 1 | hormaechei | 78 | | | |  |  |  |
| GCA_002333745.1 | SAMD00089468 | 9-Aug-10 | Tokyo, Japan | Homo sapiens | NA | 1 | hormaechei | 78 | | | |  |  |  |
| GCA_002333985.1 | SAMD00089480 | 23-Aug-10 | Tokyo, Japan | Homo sapiens | NA | 1 | hormaechei | 78 | | | |  |  |  |
| GCA_002333965.1 | SAMD00089479 | 23-Aug-10 | Tokyo, Japan | Homo sapiens | NA | 1 | hormaechei | 78 | | | |  |  |  |
| GCA_002333805.1 | SAMD00089471 | 12-Aug-10 | Tokyo, Japan | Homo sapiens | NA | 1 | hormaechei | 78 | | | |  |  |  |
| GCA_002333605.1 | SAMD00089461 | 10-Jul-10 | Tokyo, Japan | Homo sapiens | NA | 1 | hormaechei | 78 | | | |  |  |  |
| GCA_002334125.1 | SAMD00089487 | 30-Aug-10 | Tokyo, Japan | Homo sapiens | NA | 1 | hormaechei | 78 | | | |  |  |  |
| GCA_002333705.1 | SAMD00089466 | 26-Jul-10 | Tokyo, Japan | Homo sapiens | NA | 1 | hormaechei | 78 | | | |  |  |  |
| GCA_002334185.1 | SAMD00089490 | 11-Sep-10 | Tokyo, Japan | Homo sapiens | NA | 1 | hormaechei | 78 | | | |  |  |  |
| GCA_002333485.1 | SAMD00089455 | 24-Jun-10 | Tokyo, Japan | Homo sapiens | NA | 1 | hormaechei | 78 | | | |  |  |  |
| GCA_002333645.1 | SAMD00089463 | 6-May-10 | Tokyo, Japan | Homo sapiens | NA | 1 | hormaechei | 78 | | | |  |  |  |
| GCA_002333505.1 | SAMD00089456 | 5-Jul-10 | Tokyo, Japan | Homo sapiens | NA | 1 | hormaechei | 78 | | | |  |  |  |
| GCA_002333845.1 | SAMD00089473 | 12-Aug-10 | Tokyo, Japan | Homo sapiens | NA | 1 | hormaechei | 78 | | | |  |  |  |
| GCA_002333665.1 | SAMD00089464 | 14-Jul-10 | Tokyo, Japan | Homo sapiens | NA | 1 | hormaechei | 78 | | | |  |  |  |
| GCA_002333465.1 | SAMD00089454 | 24-Jun-10 | Tokyo, Japan | Homo sapiens | NA | 1 | hormaechei | 78 | | | |  |  |  |
| GCA_002333545.1 | SAMD00089458 | 6-Jul-10 | Tokyo, Japan | Homo sapiens | NA | 1 | hormaechei | 78 | | | |  |  |  |
| GCA_002333785.1 | SAMD00089470 | 11-Aug-10 | Tokyo, Japan | Homo sapiens | NA | 1 | hormaechei | 78 | | | |  |  |  |
| GCA_002333725.1 | SAMD00089467 | 31-Jul-10 | Tokyo, Japan | Homo sapiens | NA | 1 | hormaechei | 78 | | | |  |  |  |
| GCA_015684875.1 | SAMN11230928 | 2016 | Australia: Brisbane | Homo sapiens | urine | 4 | hormaechei | 109 | | | |  |  |  |
| GCA_015683855.1 | SAMN11230922 | 2015 | Australia: Brisbane | Homo sapiens | screening swab | 4 | hormaechei | 109 | | | |  |  |  |
| GCA_015684315.1 | SAMN11230986 | 2016 | Australia: Brisbane | Homo sapiens | respiratory | 4 | hormaechei | 527 | | | |  |  |  |
| GCA_003965785.1 | SAMN08027259 | 18-Oct-16 | China: Sichuan | Homo sapiens | NA | 4 | hormaechei | 127 | | | |  |  |  |
| GCA_021491635.1 | SAMN24596082 | Jul-17 | China:Hojhot | Homo sapiens | urine | 8 | hormaechei | 114 | | | |  |  |  |
| GCA_003986775.1 | SAMN08932723 | 2016 | China: Sichuan | Homo sapiens | NA | 4 | hormaechei | 395 | | | |  |  |  |
| GCA_003985265.1 | SAMN08027261 | 1-Jul-14 | China: Sichuan, Chengdu | Homo sapiens | NA | 4 | hormaechei | 291 | | | |  |  |  |
| GCA_003261215.1 | SAMN09425566 | Sep-11 | China:Shandong | Homo sapiens | secretion | 70 | hormaechei | 200 | | | |  |  |  |
| GCA_015683555.1 | SAMN11230983 | 2016 | Australia: Brisbane | Homo sapiens | screening swab | 4 | hormaechei | 1131 | | | |  |  |  |
| GCA_019449085.1 | SAMN20500115 | 2016 | China: Shenzhen | Homo sapiens | urine | 26 | hormaechei | 171 | | | |  |  |  |
| GCA_015684855.1 | SAMN11230957 | 2016 | Australia: Brisbane | Homo sapiens | blood | 4 | hormaechei | 66 | | | |  |  |  |
| GCA_015683955.1 | SAMN11230958 | 2016 | Australia: Brisbane | Homo sapiens | blood | 4 | hormaechei | 66 | | | |  |  |  |
| GCA_015684395.1 | SAMN11230991 | 2017 | Australia: Brisbane | Homo sapiens | screening swab | 4 | hormaechei | 66 | | | |  |  |  |
| GCA_015683875.1 | SAMN11230940 | 2016 | Australia: Brisbane | Homo sapiens | screening swab | 4 | hormaechei | 66 | | | |  |  |  |
| GCA_015684775.1 | SAMN11230936 | 2016 | Australia: Brisbane | Homo sapiens | respiratory | 4 | hormaechei | 66 | | | |  |  |  |
| GCA_015684095.1 | SAMN11230989 | 2016 | Australia: Brisbane | Homo sapiens | blood | 4 | hormaechei | 66 | | | |  |  |  |
| GCA_015684275.1 | SAMN11230984 | 2016 | Australia: Brisbane | Homo sapiens | wound/skin/soft tissue | 4 | hormaechei | 66 | | | |  |  |  |
| GCA_015686355.1 | SAMN11230933 | 2016 | Australia: Brisbane | Homo sapiens | screening swab | 4 | hormaechei | 66 | | | |  |  |  |
| GCA_015683915.1 | SAMN11230938 | 2016 | Australia: Brisbane | Homo sapiens | screening swab | 4 | hormaechei | 66 | | | |  |  |  |
| GCA_929606295.1 | SAMEA5226467 | 5-Feb-18 | United Kingdom | Homo sapiens | screen | 70 | hormaechei | 94 | | | |  |  |  |
| GCA_900558525.1 | SAMEA2053718 | NA | United Kingdom | Homo sapiens | NA | 70 | hormaechei | 94 | | | |  |  |  |
| GCA_900076405.1 | SAMEA2273438 | 2009 | United Kingdom | Homo sapiens | blood | 70 | hormaechei | 94 | | | |  |  |  |
| GCA_929608385.1 | SAMEA6451146 | 20-Dec-16 | United Kingdom | Homo sapiens | tissue | 4 | hormaechei | 94 | | | |  |  |  |
| GCA_900558695.1 | SAMEA2053978 | NA | United Kingdom | Homo sapiens | NA | 70 | hormaechei | 94 | | | |  |  |  |
| GCA_900558675.1 | SAMEA2053723 | NA | United Kingdom | Homo sapiens | NA | 70 | hormaechei | 94 | | | |  |  |  |
| GCA_900558665.1 | SAMEA1966964 | NA | United Kingdom | Homo sapiens | NA | 70 | hormaechei | 94 | | | |  |  |  |
| GCA_015683615.1 | SAMN11230987 | 2016 | Australia: Brisbane | Homo sapiens | urine | 4 | hormaechei | 108 | | | |  |  |  |
| GCA_015959985.1 | SAMN07501518 | 2012 | China | Homo sapiens | NA | 70 | hormaechei | 108 | | | |  |  |  |
| GCA_015681205.1 | hormaechei | 2012 | China | Homo sapiens | urine | 70 | hormaechei | 108 | | | |  |  |  |
| GCA_015684945.1 | SAMN11230930 | 2016 | Australia: Brisbane | Homo sapiens | screening swab | 4 | hormaechei | 108 | | | |  |  |  |
| GCA_015684025.1 | SAMN11230942 | 2016 | Australia: Brisbane | Homo sapiens | screening swab | 4 | hormaechei | 108 | | | |  |  |  |
| GCA_015684195.1 | SAMN11230931 | 2016 | Australia: Brisbane | Homo sapiens | bone/joint | 4 | hormaechei | NA | | | |  |  |  |
| GCA_001524915.1 | SAMN04430990 | 2014 | Australia | Homo sapiens | NA | 4 | hormaechei | 108 | | | |  |  |  |
| GCA_019448645.1 | SAMN20500136 | 2015 | China: Shenzhen | Homo sapiens | blood | 4 | hormaechei | 133 | | | |  |  |  |
| GCA_019449135.1 | SAMN20500114 | 2016 | China: Shenzhen | Homo sapiens | Drainage | 4 | hormaechei | 133 | | | |  |  |  |
| GCA_019449055.1 | SAMN20500113 | 2016 | China: Shenzhen | Homo sapiens | cerebrospinal fluid | 4 | hormaechei | 133 | | | |  |  |  |
| GCA_019537235.1 | SAMN20587857 | 2016 | China: Shenzhen | Homo sapiens | urine | 4 | hormaechei | 133 | | | |  |  |  |
| GCA_019449205.1 | SAMN20500137 | 2015 | China: Shenzhen | Homo sapiens | urine | 4 | hormaechei | 133 | | | |  |  |  |
| GCA_019537335.1 | SAMN20587855 | 2015 | China: Shenzhen | Homo sapiens | Catheter | 4 | hormaechei | 133 | | | |  |  |  |
| GCA_019537315.1 | SAMN20587858 | 2016 | China: Shenzhen | Homo sapiens | abdominal fluid | 4 | hormaechei | 133 | | | |  |  |  |
| GCA_019537255.1 | SAMN20587856 | 2015 | China: Shenzhen | Homo sapiens | urine | 4 | hormaechei | 133 | | | |  |  |  |
| GCA_021599525.1 | SAMD00246035 | 2017 | Japan:Tokyo | NA | NA | 1 | hormaechei | 133 | | | |  |  |  |
| GCA_021597725.1 | SAMD00246038 | 2018 | Japan:Tokyo | NA | NA | 1 | hormaechei | | 133 | | | | |  |
| GCA_021595045.1 | SAMD00246034 | 2017 | Japan:Tokyo | NA | NA | 1 | hormaechei | | 133 | | | | |  |
| GCA_015684755.1 | SAMN11230926 | 2015 | Australia: Brisbane | Homo sapiens | screening swab | 4 | hormaechei | | 133 | | | | |  |
| GCA_015684055.1 | SAMN11230985 | 2016 | Australia: Brisbane | Homo sapiens | urine | 4 | hormaechei | 133 | | | |  |  |  |
| GCA_015684505.1 | SAMN11230968 | 2016 | Australia: Brisbane | Homo sapiens | urine | 4 | hormaechei | 133 | | | |  |  |  |
| GCA_015684535.1 | SAMN11230967 | 2016 | Australia: Brisbane | Homo sapiens | screening swab | 4 | hormaechei | 133 | | | |  |  |  |
| GCA_015684155.1 | SAMN11230964 | 2016 | Australia: Brisbane | Homo sapiens | screening swab | 4 | hormaechei | 133 | | | |  |  |  |
| GCA_021596685.1 | SAMD00246039 | 2016 | Japan:Tokyo | NA | NA | 1 | hormaechei | | | 133 | | | | |
| GCA_021595605.1 | SAMD00246031 | 2014 | Japan:Tokyo | NA | NA | 1 | hormaechei | | | 133 | | | | |
| GCA_021595025.1 | SAMD00246033 | 2017 | Japan:Tokyo | NA | NA | 1 | hormaechei | | | 133 | | | | |
| GCA_001524995.1 | SAMN04430989 | 2014 | Australia | Homo sapiens | abdomen | 1 | hormaechei | | | 133 | | | | |
| GCA_020682465.1 | SAMN21893138 | 43578 | Japan:Aichi | Homo sapiens | NA | 1 | hormaechei | 133 | | | |  |  |  |
| GCA_020682405.1 | SAMN21893139 | 43675 | Japan:Aichi | Homo sapiens | NA | 1 | hormaechei | | 133 | |  |  |  |  |
| GCA_020702055.1 | SAMN21893163 | 43620 | Japan:Aichi | Homo sapiens | NA | 1 | hormaechei | | 133 | |  |  |  |  |
| GCA_020682345.1 | SAMN21893134 | 43640 | Japan:Aichi | Homo sapiens | NA | 1 | hormaechei | 133 | | | |  |  |  |
| GCA_020681565.1 | SAMN21893136 | 43662 | Japan:Aichi | Homo sapiens | NA | 1 | hormaechei | | 133 | |  |  |  |  |
| GCA_015865585.1 | SAMN11230976 | 2016 | Australia: Brisbane | Homo sapiens | NA | 4 | hormaechei | | 204 | |  |  |  |  |
| GCA_001472195.1 | SAMN04252903 | 2011 | Taiwan | Homo sapiens | NA | 8 | hormaechei | 204 | | | |  |  |  |
| GCA_015959185.1 | SAMN11262772 | 2018 | Australia: Brisbane | Homo sapiens | NA | 4 | hormaechei | 254 | | | |  |  |  |
| GCA_016774285.1 | SAMN17487907 | 43455 | Australia: Melbourne | Homo sapiens | NA | 4 | hormaechei | 190 | | | |  |  |  |
| GCA_020889565.1 | SAMD00412791 | 42312 | Japan | NA | NA | 1 | hormaechei | 190 | | | |  |  |  |
| GCA_020889525.1 | SAMD00412792 | 42313 | Japan | NA | NA | 1 | cloacae | 190 | | | |  |  |  |
| GCA_002850625.1 | SAMN07816158 | 29-Mar-17 | China: Sichuan, Chengdu | Homo sapiens | NA | 70 | hormaechei | 93 | | | |  |  |  |
| GCA_015681995.1 | SAMN07501520 | 2012 | China | Homo sapiens | wound | 8 | hormaechei | 93 | | | |  |  |  |
| GCA_008369025.1 | SAMN10856226 | 2-Jul-14 | China: Nanjing | Homo sapiens | urine | 26 | hormaechei | 93 | | | |  |  |  |
| GCA_008369005.1 | SAMN10856227 | 2-Sep-15 | China: Nanjing | Homo sapiens | urine | 26 | hormaechei | 93 | | | |  |  |  |
| GCA_020701935.1 | SAMN21893145 | 26-May-18 | Japan:Aichi | Homo sapiens | NA | 1 | hormaechei | 346 | | | |  |  |  |
| GCA_020682545.1 | SAMN21893150 | 13-Aug-18 | Japan:Aichi | Homo sapiens | NA | 1 | hormaechei | 346 | | | |  |  |  |
| GCA_015684135.1 | SAMN11230977 | 2016 | Australia: Brisbane | Homo sapiens | urine | 4 | hormaechei | 604 | | | |  |  |  |
| GCA_004011415.2 | SAMD00143515 | 2015 | Japan:Nagoya | Homo sapiens | NA | 1 | hormaechei | 113 | | | |  |  |  |
| GCA_002333625.1 | SAMD00089462 | 16-Jul-10 | NA | Homo sapiens | NA | 1 | hormaechei | 234 | | | |  |  |  |
| GCA_020682505.1 | SAMN21893146 | 31-May-18 | Japan:Aichi | Homo sapiens | NA | 1 | hormaechei | 45 | | | |  |  |  |
| GCA_015684335.1 | SAMN11230970 | 2016 | Australia: Brisbane | Homo sapiens | urine | 4 | hormaechei | 45 | | | |  |  |  |
| GCA_015684575.1 | SAMN11230970 | 2016 | Australia: Brisbane | Homo sapiens | urine | 4 | hormaechei | 45 | | | |  |  |  |
| GCA_015684295.1 | SAMN11231003 | 2017 | Australia: Brisbane | Homo sapiens | urine | 4 | hormaechei | 45 | | | |  |  |  |
| YQ13530hy | SAMN28918927 | 2017 | China | Homo sapiens | sputum | 4 | hormaechei | 51 | | | |  |  |  |
| YQ13422hy | SAMN28919657 | 2017 | China | Homo sapiens | sputum | 4 | hormaechei | 51 | | | |  |  |  |
| GCA_015684015.1 | SAMN11230988 | 2016 | Australia: Brisbane | Homo sapiens | urine | 4 | hormaechei | 110 | | | |  |  |  |
| GCA_021165665.1 | SAMN23673798 | NA | Ireland | Homo sapiens | blood culture | 4 | hormaechei | 110 | | | |  |  |  |
| GCA_015683815.1 | SAMN11230943 | 2016 | Australia: Brisbane | Homo sapiens | screening swab | 4 | hormaechei | 110 | | | |  |  |  |
| GCA_021491615.1 | SAMN24596081 | Jun-21 | China:Hohhot | Homo sapiens | blood | imp-70 | hormaechei | 175 | | | |  |  |  |
| GCA_015959505.1 | SAMN11262768 | 2017 | Australia: Brisbane | Homo sapiens | NA | 4 | hormaechei | 175 | | | |  |  |  |
| GCA_002334865.1 | SAMD00089524 | 12-Oct-10 | Japan | Homo sapiens | NA | IMP-1 | hormaechei | 175 | | | |  |  |  |
| GCA_002334845.1 | SAMD00089523 | 13-Sep-10 | Japan | Homo sapiens | NA | I-1 | hormaechei | 175 | | | |  |  |  |
| GCA_002334825.1 | SAMD00089522 | 26-Jul-10 | Japan | Homo sapiens | NA | 1 | hormaechei | 175 | | | |  |  |  |
| GCA_015684935.1 | SAMN11230934 | 2016 | Australia: Brisbane | Homo sapiens | screening swab | 4 | hormaechei | 91 | | | |  |  |  |
| GCA_015684655.1 | SAMN11230979 | 2016 | Australia: Brisbane | Homo sapiens | urine | 4 | hormaechei | 90 | | | |  |  |  |
| GCA_015683655.1 | SAMN11230963 | 2016 | Australia: Brisbane | Homo sapiens | screening swab | 4 | hormaechei | 90 | | | |  |  |  |
| GCA_015683755.1 | SAMN11230978 | 2016 | Australia: Brisbane | Homo sapiens | other | 4 | hormaechei | 90 | | | |  |  |  |
| GCA_001525015.1 | SAMN04430991 | 2014 | Australia | Homo sapiens | urine | 4 | hormaechei | 90 | | | |  |  |  |
| GCA_015683595.1 | SAMN11230946 | 2016 | Australia: Brisbane | Homo sapiens | screening swab | 4 | hormaechei | 90 | | | |  |  |  |
| GCA_015684735.1 | SAMN11230918 | 2014 | Australia: Brisbane | Homo sapiens | blood | 4 | hormaechei | 90 | | | |  |  |  |
| GCA_000770745.2 | SAMN03013105 | 8-Jun-13 | Australia | Homo sapiens | endotracheal aspirate | 4 | hormaechei | 90 | | | |  |  |  |
| GCA_015959425.1 | SAMN11262769 | 2016 | Australia: Brisbane | Homo sapiens | NA | 4 | hormaechei | 90 | | | |  |  |  |
| GCA_015683565.1 | SAMN11230955 | 2016 | Australia: Brisbane | Homo sapiens | respiratory | 4 | hormaechei | 90 | | | |  |  |  |
| GCA_015684995.1 | SAMN11230932 | 2016 | Australia: Brisbane | Homo sapiens | respiratory | 4 | hormaechei | 415 | | | |  |  |  |
| GCA_015683715.1 | SAMN11230973 | 2016 | Australia: Brisbane | Homo sapiens | urine | 4 | hormaechei | 415 | | | |  |  |  |
| GCA_015684795.1 | SAMN11231012 | 2016 | Australia: Brisbane | Homo sapiens | Ureteroscope | 4 | hormaechei | 415 | | | |  |  |  |
| GCA_015684695.1 | SAMN11231013 | 2016 | Australia: Brisbane | Homo sapiens | Bronchoscope | 4 | hormaechei | 415 | | | |  |  |  |
| GCA_015683885.1 | SAMN11230980 | 2015 | Australia: Brisbane | Homo sapiens | blood | 4 | hormaechei | 415 | | | |  |  |  |
| GCA_015683835.1 | SAMN11231011 | 2016 | Australia: Brisbane | Homo sapiens | Colonoscope | 4 | hormaechei | 415 | | | |  |  |  |
| GCA_015683635.1 | SAMN11230962 | 2016 | Australia: Brisbane | Homo sapiens | urine | 4 | hormaechei | 415 | | | |  |  |  |
| GCA_015684435.1 | SAMN11230924 | 2015 | Australia: Brisbane | Homo sapiens | screening swab | 4 | hormaechei | 415 | | | |  |  |  |
| GCA_015684635.1 | SAMN11230956 | 2016 | Australia: Brisbane | Homo sapiens | Intra-abdominal | 4 | hormaechei | 415 | | | |  |  |  |
| GCA_015684455.1 | SAMN11230974 | 2016 | Australia: Brisbane | Homo sapiens | urine | 4 | hormaechei | 415 | | | |  |  |  |
| GCA_015684115.1 | SAMN11230939 | 2016 | Australia: Brisbane | Homo sapiens | screening swab | 4 | hormaechei | 415 | | | |  |  |  |
| GCA_015683995.1 | SAMN11230950 | 2016 | Australia: Brisbane | Homo sapiens | screening swab | 4 | hormaechei | 415 | | | |  |  |  |

Table S1
