# Supplementary material for: Detection of IMP-4 and SFO-1 co-producing ST51 Enterobacter hormaechei clinical isolates
Source: Front Cell Infect Microbiol. 2022 Oct 27;12:998578. doi: 10.3389/fcimb.2022.998578 (PMC9647121; doi:10.3389/fcimb.2022.998578)
Supplement: Supplementary file 9 [file Table_2.docx]

YQ13422hy YQ13530hy

YQ13422hy 1.0 0.9967394553987596

YQ13530hy 0.999717639807624 1.0
