# Supplementary material for: Detection of IMP-4 and SFO-1 co-producing ST51 Enterobacter hormaechei clinical isolates
Source: Front Cell Infect Microbiol. 2022 Oct 27;12:998578. doi: 10.3389/fcimb.2022.998578 (PMC9647121; doi:10.3389/fcimb.2022.998578)
Supplement: Supplementary file 10 [file Table_3.docx]

| **Genome** | **YQ13422hy** | **YQ13530hy** |
| --- | --- | --- |
| **Size** | **4,983,830** | **4,958,206** |
| **GC Content** | **55.0%** | **55.0%** |
| **N50** | **4,570,859** | **4571686** |
| **L50** | **1** | **1** |
| **Number of Contigs (with PEGs)** | **5** | **5** |
| **Number of Subsystems** | **377** | **377** |
| **Number of Coding Sequences** | **4835** | **4811** |
| **Number of RNAs** | **110** | **110** |
