# Supplementary material for: Detection of IMP-4 and SFO-1 co-producing ST51 Enterobacter hormaechei clinical isolates
Source: Front Cell Infect Microbiol. 2022 Oct 27;12:998578. doi: 10.3389/fcimb.2022.998578 (PMC9647121; doi:10.3389/fcimb.2022.998578)
Supplement: Supplementary file 11 [file Table_4.docx]

|  | pYQ13422-IMP-4 |  | pYQ13422-SFO-1 |  | pYQ13530-SFO-1 |  |
| --- | --- | --- | --- | --- | --- | --- |
| \| Type \| \| --- \| | Location | Gene/locus tag | Location | Gene/locus tag | Location | Gene/locus tag |
| *ori*T region | [38086...38186](http://tool-mml.sjtu.edu.cn/oriTfinder/report_conjugal.php?job_id=pIWoUGj5D#oriT) (+) | - | [193150…193401](http://tool-mml.sjtu.edu.cn/oriTfinder/report_conjugal.php?job_id=pvKyaUJld#oriT)(-) | - | [193149...193400](http://tool-mml.sjtu.edu.cn/oriTfinder/report_conjugal.php?job_id=pyO5GPYLl#oriT)(-) | - |
| Relaxase | [032754...35990](http://tool-mml.sjtu.edu.cn/oriTfinder/report_conjugal.php?job_id=pIWoUGj5D#oriT)(-) | ORF1_43 | [195153...198299](http://tool-mml.sjtu.edu.cn/oriTfinder/report_conjugal.php?job_id=pvKyaUJld#oriT)(+) | ORF1_210 | [195152...198298](http://tool-mml.sjtu.edu.cn/oriTfinder/report_conjugal.php?job_id=pyO5GPYLl#oriT)(+) | ORF1_216 |
| T4CP | [35990…37519](http://tool-mml.sjtu.edu.cn/oriTfinder/report_conjugal.php?job_id=pIWoUGj5D#T4CP)(-) | ORF1_44 | [0198299...200383](http://tool-mml.sjtu.edu.cn/oriTfinder/report_conjugal.php?job_id=pvKyaUJld#T4CP)(+) | ORF1_211 | [0198298...200382](http://tool-mml.sjtu.edu.cn/oriTfinder/report_conjugal.php?job_id=pyO5GPYLl#T4CP)(+) | ORF1_217 |
| T4SS  gene cluster | [13777...37519](http://tool-mml.sjtu.edu.cn/oriTfinder/report_conjugal.php?job_id=pIWoUGj5D#T4SS) | ORF1_19 ORF1_21 ORF1_22 ORF1_23 ORF1_24 ORF1_25 ORF1_26 ORF1_27 ORF1_28 ORF1_29 ORF1_30 ORF1_43 ORF1_44 | [2025...11636](http://tool-mml.sjtu.edu.cn/oriTfinder/report_conjugal.php?job_id=pvKyaUJld#T4SS) | ORF1_3 ORF1_4 ORF1_5 ORF1_6 ORF1_8 ORF1_11 ORF1_12 | [2025…11636](http://tool-mml.sjtu.edu.cn/oriTfinder/report_conjugal.php?job_id=pyO5GPYLl#T4SS) | ORF1_3 ORF1_4 ORF1_5 ORF1_6 ORF1_8 ORF1_11 ORF1_12 |
| T4SS  gene cluster |  |  | [30123...39405](http://tool-mml.sjtu.edu.cn/oriTfinder/report_conjugal.php?job_id=pvKyaUJld#T4SS) | ORF1_27 ORF1_28 ORF1_30 ORF1_31 ORF1_33 | [30123...39405](http://tool-mml.sjtu.edu.cn/oriTfinder/report_conjugal.php?job_id=pyO5GPYLl#T4SS) | ORF1_29 ORF1_30 ORF1_32 ORF1_33 ORF1_35 |
| T4SS  gene cluster |  |  | [181845...200383](http://tool-mml.sjtu.edu.cn/oriTfinder/report_conjugal.php?job_id=pvKyaUJld#T4SS) | ORF1_199 ORF1_203 ORF1_204 ORF1_205 ORF1_211 | [181844...200382](http://tool-mml.sjtu.edu.cn/oriTfinder/report_conjugal.php?job_id=pyO5GPYLl#T4SS) | ORF1_205 ORF1_209 ORF1_210 ORF1_211 ORF1_217 |
| AR |  | ORF1_7 ORF1_36 |  | ORF1_106 ORF1_108 ORF1_112 ORF1_142 ORF1_156 ORF1_257 ORF1_260 ORF1_264 ORF1_274 ORF1_275 ORF1_282 ORF1_292 ORF1_294 ORF1_297 ORF1_302 |  | ORF1_108 ORF1_110 ORF1_115 ORF1_146 ORF1_161 ORF1_253 ORF1_263 ORF1_265 ORF1_268 ORF1_273 |

TABLE S4 Type IV secretion system components. T4CP, type IV coupling protein; T4SS, type IV secretion system; ORF,

|  | pYQ13422-IMP-4 |  |
| --- | --- | --- |
| \| Type \| \| --- \| | Location | Gene/locus tag |
| *ori*T region | [38086...38186](http://tool-mml.sjtu.edu.cn/oriTfinder/report_conjugal.php?job_id=pIWoUGj5D#oriT) (+) | - |
| Relaxase | [032754...35990](http://tool-mml.sjtu.edu.cn/oriTfinder/report_conjugal.php?job_id=pIWoUGj5D#oriT)(-) | ORF1_43 |
| T4CP | [35990…37519](http://tool-mml.sjtu.edu.cn/oriTfinder/report_conjugal.php?job_id=pIWoUGj5D#T4CP)(-) | ORF1_44 |
| T4SS  gene cluster | [13777...37519](http://tool-mml.sjtu.edu.cn/oriTfinder/report_conjugal.php?job_id=pIWoUGj5D#T4SS) | ORF1_19 ORF1_21 ORF1_22 ORF1_23 ORF1_24 ORF1_25 ORF1_26 ORF1_27 ORF1_28 ORF1_29 ORF1_30 ORF1_43 ORF1_44 |
| T4SS  gene cluster |  |  |
| T4SS  gene cluster |  |  |
| AR |  | ORF1_7 ORF1_36 |

open reading frame.
