# Supplementary material for: Detection of IMP-4 and SFO-1 co-producing ST51 Enterobacter hormaechei clinical isolates
Source: Front Cell Infect Microbiol. 2022 Oct 27;12:998578. doi: 10.3389/fcimb.2022.998578 (PMC9647121; doi:10.3389/fcimb.2022.998578)
Supplement: Supplementary file 12 [file Table_5.docx]

GCA_004011395 GCA_000770745 GCA_004011355 GCA_001472005 GCA_015681375 GCA_001472195 GCA_015681395 GCA_001524915 GCA_015681995 GCA_001524995 GCA_015683115 GCA_001525015 GCA_015683495 GCA_001526005 GCA_015683555 GCA_002264125 GCA_015683565 GCA_002333465 GCA_015683595 GCA_002333485 GCA_015683615 GCA_002333505 GCA_015683635 GCA_002333525 GCA_015683655 GCA_002333545 GCA_015683715 GCA_002333565 GCA_015683755 GCA_002333585 GCA_015683815 GCA_002333605 GCA_015683835 GCA_002333625 GCA_015683855 GCA_002333645 GCA_015683875 GCA_002333665 GCA_015683885 GCA_002333705 GCA_015683915 GCA_002333725 GCA_015683955 GCA_002333745 GCA_015683995 GCA_002333765 GCA_015684015 GCA_002333785 GCA_015684025 GCA_002333805 GCA_015684055 GCA_002333825 GCA_015684095 GCA_002333845 GCA_015684115 GCA_002333865 GCA_015684135 GCA_002333905 GCA_015684155 GCA_002333925 GCA_015684175 GCA_002333945 GCA_015684195 GCA_002333965 GCA_015684275 GCA_002333985 GCA_015684295 GCA_002334065 GCA_015684315 GCA_002334085 GCA_015684335 GCA_002334105 GCA_015684395 GCA_002334125 GCA_015684435 GCA_002334145 GCA_015684455 GCA_002334165 GCA_015684505 GCA_002334185 GCA_015684535 GCA_002334745 GCA_015684575 GCA_002334825 GCA_015684635 GCA_002334845 GCA_015684655 GCA_002334865 GCA_015684695 GCA_002850625 GCA_015684735 GCA_002923255 GCA_015684755 GCA_003261215 GCA_015684775 GCA_003965785 GCA_015684795 GCA_003985265 GCA_015684855 GCA_003986775 GCA_004011415 GCA_015684875 GCA_008369005 GCA_015684935 GCA_008369025 GCA_015684945 GCA_013340545 GCA_015684995 GCA_014117285 GCA_015685475 GCA_015681205 GCA_015685485 GCA_015686355 GCA_015865585 GCA_015959185 GCA_015959425 GCA_015959505 GCA_015959985 GCA_016774285 GCA_018067745 GCA_019448645 GCA_019449055 GCA_019449085 GCA_019449135 GCA_019449205 GCA_019537235 GCA_019537255 GCA_019537315 GCA_019537335 GCA_020149545 GCA_020681565 GCA_020682185 GCA_020682245 GCA_020682345 GCA_020682405 GCA_020682465 GCA_020682485 GCA_020682505 GCA_020682545 GCA_020701935 GCA_020701955 GCA_020701995 GCA_020702015 GCA_020702055 GCA_020733125 GCA_020888965 GCA_020889385 GCA_020889425 GCA_020889525 GCA_020889565 GCA_021165665 GCA_021491615 GCA_021491635 GCA_021543195 GCA_021595025 GCA_021595045 GCA_021595605 GCA_021596685 GCA_021597725 GCA_021599525 GCA_900076405 GCA_900558525 GCA_900558665 GCA_900558675 GCA_900558695 GCA_929606295 GCA_929608385 YQ13422hy YQ13530hy

GCA_004011395 1.0 0.8822785626331993 0.8785065975609756 0.8789951627573003 0.869901002617801 0.8784498172069217 0.8950877187423838 0.8838129095583083 0.8807262278787878 0.882783336461755 0.9873663565565144 0.8799044615384615 0.9874411486486487 0.8788807831325302 0.868260590078329 0.894324858190709 0.881890146318062 0.8712108238045738 0.8825043291676492 0.8710166927695119 0.8723362840967576 0.8700629726918075 0.8818237862703469 0.8703042447368422 0.8798819315167592 0.8705665023474178 0.8818559117716442 0.8702493356092437 0.8828993479284368 0.8702087509758002 0.8812919497463156 0.8712689413298565 0.8817452044433941 0.8695779888564608 0.8822778163653664 0.8702022166971998 0.8819267802585193 0.8701325695708713 0.8819868518518519 0.8709271664516129 0.8820228866463272 0.8702812688116244 0.8710949744637385 0.8708703095975234 0.8821020355871888 0.870733082706767 0.8823931534090911 0.8702889160384716 0.8822358552946814 0.8708449612403101 0.8827493868921776 0.8701101819599688 0.8816603569751057 0.8702968860561916 0.8820482841633233 0.8700321223958332 0.8824266021765418 0.8711374339720352 0.8823304536771731 0.8701744949099454 0.8869588200207469 0.8700431766233766 0.8829080180180181 0.8707682756840476 0.8818336514619882 0.8709255972078593 0.8719025073984397 0.8698861894846434 0.8818229628748214 0.869818454946767 0.8722196090534979 0.870181946100026 0.8819251069330202 0.8702806337662338 0.881863714625446 0.8699584829560238 0.8818202771855012 0.8702278100674623 0.8826396052009458 0.8710060950413225 0.8821796255400863 0.8796785503388189 0.8719924715025908 0.8726639199183882 0.8817888976377954 0.8726639199183882 0.8800898665696264 0.8814243802445456 0.8819953474676091 0.8820457331097268 0.8826997438330172 0.8708182913021102 0.8827334023809524 0.8801746671371443 0.8817904722744362 0.8714403219160443 0.8821415676059674 0.8707011169102298 0.8708769252288913 0.8714287187894073 0.8787019330024815 0.8824388614800758 0.8805112900096994 0.8809012452198853 0.880560009680542 0.8823898502495839 0.8710368412903227 0.8820967563104505 0.880575397234591 0.8920221087314663 0.8810373888091823 0.8766007325728771 0.8817651864165282 0.881362394571013 0.8829338018709523 0.8821565592168099 0.8848362340966922 0.8809123944001931 0.882989851764706 0.8797058256210424 0.8803479045346062 0.8803870873091603 0.8781124198602074 0.8804864084842707 0.8803395349391844 0.8816415690120127 0.8804842829964328 0.8804879001189061 0.8805712422655878 0.8867680046948356 0.8791067060525681 0.8780089719853839 0.8778199731510862 0.879626948989413 0.8800941970278043 0.8791699178942285 0.8790474045432576 0.8786565574584264 0.8700425709033615 0.8701089206932773 0.8949397449344458 0.8791748686430465 0.8792715393880992 0.8800752169743467 0.893866880799429 0.8957870364198999 0.8859800498007968 0.8939543815564771 0.8808409398049013 0.8808124976145039 0.8830245051521688 0.8739039834544997 0.8688312923728814 0.8725281717597472 0.8807862965616046 0.8799270895522388 0.8807139727337958 0.8809483452722064 0.8797372308063739 0.8798916791314838 0.872513622816033 0.8723131442505134 0.872284319465845 0.8722868489984592 0.8721768115196709 0.8719393199381763 0.8721815378690628 0.8782578239488567 0.8781599311193112

GCA_000770745 0.8769218270432393 1.0 0.9577291764705882 0.9593035285945821 0.9564753517649963 0.9876520508287293 0.8836624935798666 0.9775770490701322 0.9873174662394142 0.9881995925444301 0.8792232768361582 0.9982249926362298 0.8791070628544425 0.9590100023218017 0.9696727923038974 0.8843641298767968 0.9993793486438329 0.956595651425762 0.9993867520189088 0.9565240222772278 0.9751394681107101 0.9561989366351057 0.991137938034188 0.9562571673189826 0.9977927103594081 0.9558918084589206 0.9915590721649484 0.9557320390243902 0.998192998965874 0.9560831734047736 0.9896860306530431 0.9558082194113354 0.991140746300665 0.9883095827439887 0.9713136369740376 0.9565760871706477 0.9719697874745648 0.9562108577659317 0.9910803923259325 0.9559608908739741 0.9720206814580032 0.9560226957575758 0.9692137478746662 0.9559598235436307 0.9911283723930338 0.955869355620155 0.9890282164502164 0.9559129022082019 0.9776206215846993 0.9559102464958918 0.9873584158847917 0.9561969323964139 0.9718899525209134 0.9562696871210283 0.9911436478391099 0.9559057229353354 0.9880662463343108 0.956080084848485 0.9880969329214476 0.9558535411108416 0.8796206774707759 0.9559632412121211 0.9766337225616657 0.9559344305019305 0.9718921628326567 0.956067959874305 0.9867190509538759 0.9564431135082129 0.9718016929044033 0.9563413643031785 0.9862056366139024 0.9559021849348143 0.97192908781484 0.9559994840116279 0.9915753487868284 0.9560964713005571 0.9911527690655212 0.9559490256286267 0.98821287812363 0.9559849022447503 0.9880999755989351 0.9587802249824233 0.9868027769985973 0.9879948417431194 0.9910133542659162 0.9879948417431194 0.9978276414895855 0.988195069521077 0.9910969266937381 0.9865393605683836 0.9994168142463598 0.9558917472924188 0.987980574636724 0.9711827377921778 0.9719866380284873 0.9695799729530366 0.9910158168529664 0.9694260735899299 0.9692386584778419 0.9692991648873073 0.9882630047479086 0.971511006559602 0.9874414477511748 0.9971393896812081 0.9868917474010175 0.9776957559322034 0.9562621235709073 0.9915776344086021 0.9584318738489873 0.8826275504677499 0.9769268661233993 0.9581950956648099 0.9719663996337835 0.9879200902090208 0.9876952380952381 0.9993968735224588 0.9884660000000001 0.9768922412982126 0.9884560937840786 0.9582515751343773 0.9873524994533129 0.9873679225043783 0.9708544687791241 0.9873119356955381 0.9874215915061296 0.986706330563665 0.9874028116826503 0.9873788428851602 0.9874519167030092 0.8782573952985679 0.9873035839432247 0.9580076210625295 0.9579751200000002 0.9872192114615728 0.9872882186324408 0.9872441396176078 0.9582890415111939 0.9868587099684258 0.987279429309535 0.9872958376349132 0.8852614450288004 0.9583785128205128 0.9583061864801864 0.9873015840707965 0.8841894836956522 0.8842416679360242 0.8776218037556203 0.8844051034126165 0.9874238569206842 0.9875448496324348 0.9885319322183099 0.9876375950526799 0.9696581844946025 0.9491711460165871 0.9875124772878353 0.9872883507214206 0.9875791694425938 0.9875394940079895 0.9872852846975089 0.9871974688334818 0.9754936608863198 0.9748185560602437 0.9756744022131345 0.9756018542568542 0.9755608567990371 0.9753093473531544 0.9755047377285851 0.988365553047404 0.9882242288557214

GCA_004011355 0.8773675547619049 0.9575794528546713 1.0 0.9964955872516231 0.9960940879306701 0.957539610963749 0.8835378532742492 0.959383324664066 0.9575386458333334 0.9588083784373634 0.8804662008234218 0.959174572907679 0.8803791947694424 0.9964301501382853 0.9551237929354447 0.8858855924400293 0.9573703751363141 0.9955992163941388 0.9581110368613927 0.9954376975797815 0.9546357944589154 0.9948694036697249 0.9585237199219597 0.9948407489451477 0.9592510383351589 0.994470841995842 0.9591038098360656 0.9946282006269594 0.9594666766659525 0.994845780238145 0.958680358974359 0.9943923136678564 0.9585764820846906 0.9562529860618946 0.9581996434040693 0.995558758752387 0.9593206057292806 0.9948797302344885 0.9585926555023923 0.9946770856668741 0.9593667986870896 0.9947634559434748 0.9544651151745428 0.994656185246581 0.958696776229865 0.9945045925616383 0.9589791054451168 0.9946020725604671 0.9588268330006653 0.994481165695254 0.9574576215053763 0.9948321198252548 0.9592692805598074 0.9949538246929003 0.9586332220294882 0.9946743985040516 0.9586590523525513 0.9947201545853354 0.9587392220994475 0.9946861915692822 0.8752151752366335 0.9946445030214628 0.9588199825783974 0.9945532571191023 0.959325118865867 0.9947270183486238 0.9553028011681675 0.9954453105329417 0.9594201162535645 0.9954814623926251 0.9549181807539218 0.994839898298049 0.9593037041107937 0.994783153078203 0.9591721554381331 0.9948720706126687 0.958532358326068 0.9947115587380655 0.9587513554481547 0.9946278144458282 0.9587100066681485 0.9969462480063797 0.955051210974456 0.9547401130595293 0.9585279565884675 0.9547401130595293 0.9590931353135312 0.9584647893379191 0.95856551798873 0.9578227916758912 0.9581437945175912 0.9946777069536425 0.9586758373840035 0.9578583165499124 0.9592730052493438 0.9541684540257266 0.9585749673629244 0.9539577761870676 0.954466072272083 0.9552399065857077 0.9628142444966874 0.958816558570483 0.958038970266725 0.9590617634076364 0.9579017910447761 0.9590996583645581 0.9957352649006623 0.9591363423699169 0.9883930693069306 0.8828757096171803 0.9591999469964665 0.9953532001647447 0.9594104343971631 0.9571863384955752 0.9582005126512653 0.9575054093376764 0.9576335009671181 0.9592860593882564 0.959205516052575 0.9876661786912752 0.9586428090614888 0.9586424492878723 0.9588043649923229 0.9586439158576052 0.9586511120708271 0.9582867992926614 0.9587858734939759 0.9587895524956972 0.9588317620481928 0.8749945347593584 0.9621075935028853 0.9989453222094361 0.998969766283525 0.9624084475763399 0.9627185974576271 0.9624052498932081 0.9990231472957423 0.9592753196099675 0.9543979508587787 0.954431471149261 0.8929309000000001 0.9989788179218303 0.9989895494463535 0.9625506903143586 0.8894729810298102 0.886133890865955 0.8765092664785843 0.8864037784779448 0.9623827670311187 0.9622672598989048 0.9594134827810267 0.9545990543028071 0.9546764487117162 0.9421075189440236 0.9628541777777778 0.9627993416596106 0.9603752033390411 0.9626066965046889 0.9625669386020821 0.9627700551899809 0.9555521895346034 0.9546154943739527 0.9554144055944057 0.9554814092989641 0.9553962427606179 0.9551882468477207 0.9552702510258265 0.9584598151487826 0.958359907470097

GCA_001472005 0.8756398563508065 0.960356254383914 0.9993382621627365 1.0 0.9992257985257986 0.9598900821018065 0.8835713264781493 0.9604494572559982 0.9593303172314347 0.9602217660656497 0.8770179601377953 0.9607770773769003 0.8769053665761541 0.99934699938512 0.9578505419580421 0.8822795907795908 0.9603676935827611 0.9989006697612733 0.9603229848661233 0.9989400425151042 0.9586515195835399 0.9995812390158173 0.9603988326757948 0.9994473571428573 0.9607008072063642 0.9991956132596685 0.9604367627236812 0.9992292551540678 0.9607634091943561 0.9991663246554364 0.960352843668946 0.9987435851158647 0.9604199348534203 0.9590775946601942 0.9600275064327487 0.9994527598486534 0.9602832199440821 0.9996102562417872 0.9604639962564343 0.9990009316499783 0.9603706458381908 0.9991509206210366 0.9580029759841545 0.9990023071895425 0.9604741810545966 0.9989024142451388 0.9604893613053613 0.9990645215100965 0.9607126379351172 0.9989319759825327 0.9602405115323133 0.9995894180939834 0.9602595410065238 0.9995968107989466 0.9604839047619048 0.9990998211169284 0.9606516369684011 0.9989739127575625 0.9603017503519474 0.9990732916117699 0.8759801343363783 0.9990659108781128 0.9602293265834707 0.9989099694922642 0.9603463695450326 0.99896553098314 0.958941777438248 0.9992575576337538 0.9603277419354838 0.9992556959826275 0.9585901452128969 0.9992560950098061 0.9603782248658737 0.9991768239405855 0.9604843128517826 0.9996205688033253 0.9603596823914059 0.9992132264397906 0.9602770814849404 0.9990150709142482 0.960266874262798 0.9986800911656176 0.9585977544466404 0.9585086361468765 0.9605513292625646 0.9585086361468765 0.9606494274269558 0.9598985875316309 0.9603588149350649 0.9596508309659089 0.960421811781272 0.9990857669776524 0.9603040742481203 0.9589472046242776 0.9603171415270018 0.9575513782845811 0.9605317497670086 0.9571799232863153 0.9579551303492376 0.958094155463212 0.9598867130518234 0.9600818710581641 0.9603180290602297 0.960492493626883 0.9604567439681424 0.9607385167690129 0.9994738867016624 0.9604130534883721 0.9905370319432121 0.8799241104140528 0.9603444139237509 0.9990756700149604 0.9604751791607733 0.9601633553407216 0.9604457625118036 0.9604027827733082 0.9599088956056732 0.960356624462494 0.9610748055555556 0.9903991678004537 0.9600199513212796 0.9600222183588316 0.959751214368482 0.9600242304126101 0.9600085389610391 0.9596256857142857 0.9600659038372631 0.9600699190751445 0.960072108183079 0.8765611714909884 0.9595240524099203 0.9991452061421962 0.999146650546678 0.9592718395119663 0.9594503832671185 0.9592868472906405 0.9990984780323734 0.9598855031145185 0.9580275842418235 0.9580573835480674 0.882562733721814 0.9991048411371237 0.999119079305294 0.9595696374269007 0.8859391205533597 0.8830237667594233 0.8769547031840259 0.8832503070517825 0.9605927591817759 0.9605215909620313 0.9604504653882132 0.9576672407185629 0.9572795392278954 0.9445514278442352 0.9597331505250876 0.9595992091717362 0.9598157259570497 0.9597040842105264 0.9596353496011263 0.9596555805770584 0.9587475836431227 0.9587640910215187 0.9587460069358434 0.9587688589108911 0.958733358116481 0.9583263190642112 0.9586304043661623 0.959802350295858 0.9596323795966786

GCA_015681375 0.870255621483376 0.9577041873212584 0.9993468899120971 0.9992762949640289 1.0 0.9581097539044013 0.877334529147982 0.9581877174685945 0.9579836709770116 0.9576622980632972 0.8710464761904761 0.9586923970211777 0.8709170530550666 0.9992384880524425 0.958054781144781 0.8773205022588362 0.9576371726618704 0.9988229345516362 0.9575921253534402 0.9989348420373952 0.9575746973307913 0.9992071128998095 0.9582957358847154 0.9991396113602392 0.958476136687233 0.9988104063157894 0.9582937176135014 0.9987819484906058 0.9585973212205917 0.9989927823516993 0.9584253460620525 0.9986582785668857 0.9583749325762954 0.9594924689622863 0.9579392446985944 0.9989909855257557 0.9583253637660485 0.9992362565942182 0.9584491465149361 0.9991018670886075 0.9583186873961548 0.9990575047498418 0.9577878098877478 0.9990726877637132 0.9583687870172944 0.9989592573000424 0.9585401345609066 0.9989868415485509 0.9581599257307138 0.9988275416754588 0.957489162037037 0.9991954024931333 0.9583133151400095 0.9990822567025543 0.9583088713105076 0.9990164398901331 0.9584249242970441 0.9989422111511553 0.95791482668566 0.998957235841082 0.876147416168443 0.9989804181626188 0.9583023038516406 0.9989871913905887 0.9583019497153701 0.9990928100570463 0.9588160741282614 0.9989267109424416 0.9582794008559201 0.998862083509068 0.9585967706596809 0.9991006773580924 0.9583544669687575 0.99910538071066 0.9582966125566961 0.9992138636843216 0.9583801065593179 0.9989880265374894 0.9577943653618032 0.9990705698607009 0.9577788015248989 0.9986544652701214 0.9585361191895113 0.9578134440028059 0.9584490180694246 0.9578134440028059 0.9583319905100831 0.9576608097928438 0.958329054820416 0.9578111058601134 0.9577846671421777 0.9991336855398864 0.9575939225469233 0.9572863092735342 0.9583270489078822 0.9574017372679675 0.9583112849029817 0.9573840620558979 0.9578105921521999 0.9577723224431818 0.9585187350835322 0.9577034920257083 0.9583684929245284 0.9579524486873509 0.958595965989608 0.9583749152542373 0.9990337033151491 0.9583250154430981 0.9904759077615297 0.8734559994874422 0.9579643699284011 0.9987328770099957 0.9584088166106578 0.9576708181818182 0.9583926773729627 0.9576949126167107 0.9573775336959092 0.9579573876472003 0.9587618329411765 0.9900007544141253 0.9575285560243789 0.9575059259259261 0.9578579792746115 0.9575201617440225 0.9575499320524835 0.9570292767598844 0.9576061689679384 0.9575864553111839 0.9576190521881582 0.8769458117143621 0.9573777672881755 0.9992630964467005 0.9992895750528543 0.9573892474640247 0.957376898749115 0.9574141971233201 0.9990682320441989 0.9586390738963532 0.95775573342905 0.9577891009086561 0.8768342990412025 0.9990690980143642 0.9990870429084762 0.9573219872551334 0.8801891121730383 0.8768963664676875 0.8777927317579848 0.877412719621252 0.9582970805843545 0.9582157591499408 0.9587957482185273 0.9575152539608575 0.9572730654475458 0.9440447813267814 0.957623587264151 0.957619338374291 0.957743124704771 0.9575685312204351 0.9575687464522232 0.9576553913454717 0.9580835299760193 0.9577040877653232 0.9580880028735633 0.9581298229241445 0.9581046059880239 0.9578143074324323 0.9581184615384615 0.9580709506704982 0.9580349281264974

GCA_001472195 0.8745283921267895 0.9882986156932678 0.9585909176470588 0.9594161117633248 0.9573922941468253 1.0 0.8838524148606811 0.979153089649005 0.9899812727272728 0.9888681243112188 0.8752460533465053 0.989865957781271 0.8752482239286599 0.959188179463024 0.9707225267346432 0.8822124549201443 0.9884095562063527 0.9576266601800048 0.9887452831445701 0.9577785956297568 0.9776896088845968 0.9576342792362768 0.9893151986531986 0.9580203338136408 0.9893531892865182 0.9578552253116012 0.9893335070770615 0.9568847074597758 0.9891967594254938 0.9578970735785953 0.98990666890681 0.9575596678700362 0.9892983134429182 0.98976359832636 0.9711080983004134 0.9584535764308139 0.9717616279615294 0.9579100972485769 0.9892655407239819 0.9574248843310279 0.971851632605121 0.9576485946074921 0.9701138258317026 0.9574052088804009 0.98932418431726 0.957000592042186 0.9893681728449221 0.9577558781362007 0.9787823019397055 0.9575857200762995 0.9887207425414363 0.9578155775184568 0.971647868544601 0.9576739746956315 0.9892817029880925 0.9575620200812813 0.9886518019846641 0.9573359865092749 0.9891026437038695 0.9574680850552619 0.8791576695141484 0.9574430062530063 0.9772834151939407 0.9574317543442037 0.9715991713483146 0.9573890841702535 0.9889655275065492 0.9584405779376499 0.9716648349604835 0.9583202866013854 0.9882638111490328 0.9566995251937985 0.9717223483779971 0.956728114992722 0.9893757980529772 0.9579583392729866 0.9891976897018971 0.957741412940057 0.9891497658862877 0.9576563690476191 0.989051757821292 0.9592475791478219 0.989007342850564 0.9888287326507396 0.989357015909091 0.9888287326507396 0.989689236865942 0.9889425384101536 0.9892593379712747 0.9893876012672551 0.9885369705093835 0.9573691500474835 0.9892248889387482 0.968940467160037 0.9717376704944926 0.9701614271701814 0.989359796931408 0.9697108724340177 0.9701529016553069 0.9696574874611894 0.9886195334821429 0.9715645998608211 0.9896447772277229 0.9893725529040971 0.98911928762244 0.9787961702621202 0.958269357228196 0.9893069707865167 0.9584164206385934 0.878922902816544 0.9783819806649376 0.9582637643607921 0.9718058325404377 0.9995403392330383 0.9889410874006811 0.9883830777877698 0.9891489318479687 0.9783622246014753 0.989530024122807 0.958886079828938 0.9891845900197846 0.989111020228672 0.9708193114754099 0.9890651230228471 0.9891616816882831 0.9884426666666667 0.9892009409958326 0.9891667778021496 0.9889381391797557 0.8784728367071525 0.9884414345804736 0.9585830456610087 0.9586007286192069 0.9881736333553939 0.9881733964757711 0.9881209754479098 0.9587770806566738 0.989042296079315 0.9888070589590051 0.9888110124252186 0.8822669234693876 0.9588012782133524 0.958474640569395 0.9884592317910117 0.8838869052419355 0.8816543060222282 0.8772779218708479 0.8823775986322988 0.9900193161094225 0.9899488464893152 0.9884140909090909 0.9889512195676905 0.9705095067155068 0.9507047384536873 0.9884528130511464 0.988581416184971 0.9885414203666888 0.9884062890019917 0.9884580457472797 0.9885760583778967 0.9766830561581106 0.9767127872801734 0.976739949373192 0.9767945564127289 0.9767638621022178 0.9767276342525401 0.9766392476489029 0.9887202953203089 0.9886514957944986

GCA_015681395 0.8928726115125841 0.8853350777722028 0.88175014518594 0.8844547458467642 0.8771665567176186 0.884591268134067 1.0 0.8865071378708551 0.8822001696658098 0.8866539309494451 0.8926176439412191 0.8854250125187783 0.8925431062801933 0.8836848862215554 0.8774166720779221 0.9963068142548598 0.8852597749683945 0.8770720252145923 0.8855755781637717 0.8772284387617765 0.8825683059682042 0.8769299041278297 0.8851598674337169 0.8771830616088243 0.8856441873278237 0.8770066970265203 0.8853616837885242 0.8770270416107383 0.8852160204081634 0.876954088 0.8852092549710545 0.877368988764045 0.8852803683287397 0.8771637817396003 0.8868752958727001 0.8770991048387097 0.8855278221323901 0.8768712141339 0.8854371629778672 0.876939190261974 0.885857777498112 0.8769453310289816 0.8802011921755222 0.8769257286698463 0.8852588813474108 0.8768140388711396 0.8846868901071518 0.8768965155958413 0.8868897885728667 0.8770364977447598 0.8858877659574469 0.8769278428761652 0.8857801055806939 0.8769555833777303 0.8852918459229616 0.8769238535031848 0.8848913281052366 0.8770200930851064 0.886584968804592 0.8770620484816196 0.9679377586206898 0.8768890071675074 0.8856977493092186 0.8769549391212281 0.8857542070872078 0.8769697505969752 0.8795308243243244 0.8766756569148937 0.8850299724448897 0.8765389381470666 0.8792529841021631 0.876816807436919 0.8859290934343435 0.8768259952102182 0.8855405971277399 0.8768362436818303 0.8853746795677307 0.8767959372507313 0.8863626676602087 0.8768794886062534 0.8863516035131744 0.8828750393700787 0.8787604864580595 0.8784687182148418 0.8852990688259109 0.8784687182148418 0.8856918743693238 0.8833279749373434 0.8852138475 0.8830882857864176 0.8856319108439767 0.8767645893847373 0.8867315480649188 0.882033124042879 0.8858368009061164 0.8789472479999999 0.8851011675893307 0.877279404698345 0.880057711886644 0.8788601293558607 0.8821064600409835 0.8865015664754303 0.8841626360672186 0.8872417545588961 0.884385895447724 0.8871272799800548 0.8773838965517241 0.8856210977443608 0.8825689947748196 0.9126613425047438 0.8854508594539939 0.882849942946058 0.886163141986284 0.8851965580345952 0.8849236106239039 0.8853411729019212 0.8850600588957056 0.8853733409844428 0.8849069742063492 0.8838375063163214 0.8859934431508545 0.8860021104780779 0.8838425893769153 0.8860489420218037 0.885926480931154 0.8872931561629968 0.8861415567086731 0.8861446009389673 0.8861575512725476 0.9957017109410176 0.8838702807721234 0.8809236908113645 0.880810504481434 0.8839371149021575 0.8842415419274094 0.8837734020100503 0.8818817754635511 0.882477759938838 0.8770301604278076 0.877111202565473 0.968020340985068 0.8819228469931489 0.8817759192483494 0.8842617972465581 0.9334656144411017 0.9674092956462275 0.9330261439205956 0.9325159832535884 0.8835392063094643 0.8833880271084338 0.8851791672975019 0.8773110508474576 0.8772682352941176 0.8786955297923967 0.8845235505617979 0.8840950175967822 0.8844777134986227 0.8844543606393606 0.884044537456008 0.8840690606900025 0.8796525424628452 0.8794165500794913 0.8796318225292242 0.8795935130111525 0.8795759452273333 0.8787083605679079 0.8790756743070363 0.8820931358529112 0.8816656594110116

GCA_001524915 0.8790730012330457 0.9776846225784902 0.959559557831045 0.95952953962437 0.957989474727453 0.9789337960088692 0.8862493006636037 1.0 0.9782349064683343 0.9781811684901531 0.8812612476235743 0.9777211249152926 0.8810946817640047 0.9592938000000001 0.9714238793312333 0.8861647108673978 0.9776661321605774 0.9574431476997579 0.9779177928428539 0.9575914100684263 0.9982240906047758 0.9583414056896963 0.9776056067039106 0.9585970859488171 0.9782500912825194 0.958069240172579 0.977582732362822 0.95761740913556 0.9782422234611953 0.9579740243320611 0.9793406041189933 0.957815376292378 0.9775933094170405 0.9773891509202455 0.9729130984364678 0.958673210742802 0.9741395479848586 0.9585238883587788 0.9775920698985344 0.9574711827701096 0.9741210369381398 0.9579064637058261 0.9712586976083354 0.9574255563497737 0.9776017258426966 0.9574259352517985 0.9787717239072258 0.957751090692124 0.9986147855482781 0.9576714618492987 0.9780967314058705 0.9583252022014835 0.9740867038110096 0.9583768137137377 0.977645656656208 0.9578663530529817 0.9790193731546672 0.9574823139702352 0.9783084932116627 0.957874435136429 0.8801031995748074 0.9577388023952095 0.9985557238633984 0.9574412405123338 0.9740646403197158 0.9575315182621152 0.977445293398533 0.9585521990026122 0.9737170339170915 0.9585721782412893 0.9771041851154028 0.9568721918139987 0.973925100222717 0.9569559169298033 0.9777291741877256 0.958422387774594 0.9776758333333334 0.9578477216690375 0.9783462809734513 0.9576188793922128 0.978317362760475 0.9594701243953008 0.9774315587815328 0.9763683486888732 0.9776408118498416 0.9763683486888732 0.9780755984467793 0.9772675689733152 0.977520811898904 0.9778509275558566 0.9780702481556004 0.9575491870111401 0.9784202151730257 0.9712065900468645 0.9741312664144224 0.9712902554216868 0.9775157905405405 0.970965605951524 0.9712416176124323 0.9716392 0.9778741209044762 0.9732155496688742 0.9780852622061484 0.9775861266336188 0.9779487188691945 0.9986327517760134 0.9584814631226054 0.977729247311828 0.9600926415514935 0.8848014529497806 0.9976088373092629 0.9584179789775442 0.9739627006970991 0.9791528581050741 0.9788250102156641 0.9777587800494272 0.9779458582333697 0.9975871180931745 0.9791414373626374 0.9598169748674198 0.9781506140155729 0.9781505628476085 0.9727272358276643 0.9781637986219162 0.9781991024498886 0.9776254833561332 0.9781929697912038 0.978185922718188 0.9782016988674217 0.8806915018706575 0.9769465513367781 0.9592036412535079 0.9592126817862987 0.9770381481481483 0.9771160822898032 0.9770298339317774 0.9594795635012769 0.9779385119595218 0.9772147359454856 0.9772211211089494 0.8860624533929904 0.9594959503940658 0.9593600254924682 0.9771310094001792 0.8867986529006884 0.8854953983862833 0.8799853697916667 0.8854449533181934 0.9785079916501868 0.9784130665487508 0.9789731866157646 0.9764155798635616 0.9713741878479788 0.9509692075000001 0.9773114311190371 0.9770283936550491 0.9774836896243292 0.9773813000671291 0.9771040864308107 0.977129029147982 0.9851882391713747 0.985244065959953 0.985306386138614 0.9852656146961848 0.9852184455958549 0.9849558222748814 0.9851982487609158 0.9780431434501844 0.9779302861758596

GCA_015681995 0.8762638042131351 0.9885114451124472 0.9586578468677495 0.9585155489067896 0.9574054611474219 0.9900038860103628 0.8817906422949989 0.9783705855263157 1.0 0.9891099870045484 0.8762180399230399 0.9889486523873271 0.8761737436640117 0.9584167666743595 0.9710401395908543 0.8825689432098766 0.9885921023109715 0.9573771034482759 0.9888275087873463 0.9576700862895494 0.9767024728132389 0.9585929432540617 0.9888779042576661 0.9585730405886542 0.9892147947970397 0.9582461120302529 0.9888890828729284 0.9576302562862669 0.9889434227892653 0.9581670791265556 0.9909585087719299 0.9580866320509794 0.9890176128318584 0.990920287885767 0.9722672036405006 0.9585950438077196 0.9719710264064294 0.9584622245712943 0.9889423353293413 0.9575535161744023 0.9720056477872048 0.9578460145951035 0.9709116228276186 0.9576384237726099 0.9887765958388669 0.9573137030918102 0.9906367891304346 0.9580867960479887 0.9779758361852612 0.9578593016170612 0.9889519374456994 0.9584267468742629 0.9719520917431191 0.9584787993382179 0.988890112657389 0.9579611680376029 0.989934713473316 0.9573050734249172 0.9890616447656592 0.9579382974698512 0.879179479936642 0.9577802763997165 0.9778257942442783 0.9576175498241501 0.9719013700823422 0.957421896795476 0.9895024146737869 0.9583828571428571 0.9720723411978222 0.958324549476135 0.9893236222071768 0.9570717198368912 0.971958897565457 0.9570653112232639 0.9888240097799512 0.9584557966580373 0.9889605614735908 0.9578756362784158 0.989123266419376 0.9578989925023431 0.9890064108203209 0.9583150473331793 0.9899383952855847 0.9890924398547435 0.9888200222617988 0.9890924398547435 0.9894430637530975 0.98925299048883 0.988775737596472 0.9983844987044052 0.9890411950244337 0.9577154689687353 0.9891120806840604 0.9704813743864346 0.9720030416857536 0.9703763969538315 0.9889592060323797 0.9707344665718348 0.9708478514712751 0.9709260454758556 0.9901994580731491 0.9724034717494894 0.9981148067192877 0.9882624784244303 0.9980609399636144 0.9778491700680271 0.9586095483267024 0.9889740309734514 0.9586871448118585 0.8813394129016671 0.9781324753609901 0.9576598973991888 0.9721780083371931 0.9899697462262087 0.9901927141608391 0.9885348443449048 0.9894677572329781 0.9780782804171494 0.9956013830465265 0.9581309236481782 0.9888619475982533 0.98882638209607 0.9716935230909921 0.9888534235807861 0.9888369009166303 0.9880986304736372 0.9888329847494554 0.9888046623093683 0.9888549542483661 0.8781543802742615 0.9883490024170511 0.958558085549133 0.9585559995372512 0.9883836635185592 0.9884287146923582 0.9883883922688337 0.9584050288350635 0.9902830333849215 0.9900115813739049 0.990014064516129 0.88201163204005 0.9584965074971165 0.9584927998154982 0.9884789183271295 0.8825226771457086 0.8827872497497498 0.8767723649177761 0.8827337166115491 0.9959049845520082 0.9957657036577806 0.9905821333333333 0.988656102575689 0.9705044600715137 0.9502168157248158 0.9886222005693016 0.9886636928462044 0.9887172617743702 0.9887460539000877 0.9886285029546947 0.9887782365826945 0.9769065191809838 0.9768172543488481 0.9767492828591582 0.976825625293841 0.976698519388954 0.9767766682430835 0.9767824029183337 0.9899263072183098 0.9899057001321003

GCA_001524995 0.8769738712895985 0.9876836011260287 0.9585230643274854 0.9585654222934311 0.9567627414561664 0.987923560026241 0.8837786800610377 0.9778297326557666 0.9876609243134629 1.0 0.8797066175783077 0.98844805160726 0.87952162954279 0.9581175746440055 0.9694357544881126 0.8830822647283856 0.9876041417012903 0.9564457549717652 0.9879491822279459 0.9566919298245615 0.9765229737335834 0.956827145664456 0.9886042255639097 0.9568054714640198 0.9884931835123877 0.9566643768472907 0.9886162491930278 0.9565420236920039 0.988404767868711 0.9566023706790882 0.9900937968952136 0.9563687863121615 0.9886592635658916 0.98804296875 0.9719272443751393 0.9568722737361285 0.9720807532232527 0.9568608562315996 0.9886751776429811 0.9563849168704157 0.9721323731107602 0.9564138939134686 0.9700280105845562 0.9563268832437715 0.9886096978851963 0.9564532531303707 0.9894964355385919 0.9564809541329409 0.9781303063506261 0.9563826503667483 0.9991620762129249 0.9569227312883436 0.9720753437006987 0.957018514243615 0.9886152019767943 0.9562521069335937 0.9884113746747615 0.9564528638036811 0.9988924611088951 0.9562122759462759 0.8786048642837946 0.9562133130031716 0.9778716618010329 0.9562973474865787 0.9721173785971223 0.9564568231841527 0.9871008251057828 0.956171636629323 0.9710628750000001 0.9561726739659369 0.9870280382339555 0.9564602733707591 0.9718792213947189 0.9563932477378333 0.9887159508802436 0.9567972615837215 0.9885624114853195 0.9563161636141638 0.9988831614305475 0.9563985791015626 0.998794258478828 0.9582144429001623 0.9873367375400091 0.9871297945823928 0.9886630008710802 0.9871297945823928 0.9890316349557523 0.987429006076389 0.9885840778159931 0.9876301989505903 0.987931411586684 0.9562839332683878 0.9990163401549772 0.9708281023213884 0.972124512635379 0.969675646772229 0.9885661585101777 0.9691298518518519 0.9699977485659654 0.969703637653737 0.9893818879252837 0.9721085910500663 0.9882480883639544 0.9880221786022433 0.9881983121993878 0.9780647832851744 0.9565838489646773 0.9887566178371391 0.9583405301314001 0.8835698755385352 0.9771330913190529 0.9575800952154251 0.9719366287015947 0.9880895425257731 0.9879258872377622 0.9876379245694352 0.9880340140395661 0.9772029029324847 0.9884903793176519 0.9584477303318636 0.9983307485566395 0.9982940430193189 0.9708947158570119 0.9982864183266932 0.998394291691572 0.9974908058907752 0.9982570772898868 0.9982665143084261 0.9982480675273088 0.8765269442942131 0.9984371781094528 0.9579038005138987 0.9578817363870065 0.9985492948207173 0.9985572770605761 0.998555803945009 0.9581536125896782 0.9876602313681869 0.9880189540412044 0.9880260918759901 0.8837086711711711 0.9582036403610276 0.9582781245658718 0.9985417079944456 0.8842793154761904 0.8835032355173287 0.8763163515312915 0.8834805803800718 0.9885757629693944 0.9885401956947163 0.9895079139362167 0.9870378866213152 0.9699111081409477 0.948994368355995 0.9981412141719748 0.9981022692078618 0.9982377383326686 0.9982592352121091 0.9982157542675666 0.998107591487669 0.97582564339981 0.975959527652504 0.9760255563480743 0.9760618784136784 0.9758824186267524 0.9756052435530086 0.9757275529131986 0.9875323288278308 0.9873714974450121

GCA_015683115 0.9862162196986242 0.8801373344140805 0.8759067708079081 0.8775219971126083 0.8703296010296011 0.8755387091305401 0.8917278743608474 0.8812590996034523 0.8761395866960137 0.8810238109475623 1.0 0.8782773752660203 0.9999329418103449 0.8769690992292871 0.8685994477115827 0.8914529882525697 0.8798290322580645 0.8711400864699899 0.8804405768343333 0.871130289224469 0.873684926035503 0.8699362980030722 0.8798833822501747 0.8703265319865321 0.8790372299364556 0.8706838391038696 0.8796966979142254 0.8705113323084807 0.8802882933643771 0.8707284318700838 0.8807262029190207 0.8709416517514703 0.8798024375437777 0.8682111320754717 0.879546515613994 0.8708535242966753 0.8802945971122496 0.8700342619595806 0.8799677868852459 0.8706222660596863 0.8805927095426052 0.8705738919878296 0.872415559129144 0.8706870921985816 0.8799396607393543 0.8705411506223013 0.8798496906491682 0.8705814703637751 0.8809390063724334 0.8705706561945782 0.8798344498494325 0.8700547006141248 0.880353959591268 0.8703187634408601 0.8800080442890443 0.870065677814654 0.8812212923879534 0.8708326680244399 0.8804648704179313 0.8701575690184048 0.8863327865111561 0.8700017908163267 0.8808056013179573 0.8705319681254744 0.8804011332560834 0.8706291582150102 0.8735414047680081 0.8703371515458692 0.881537625613461 0.8702327196758672 0.8731405566551214 0.8704464786238301 0.8803907087899276 0.8705574277016743 0.8798368091639113 0.8699565371837465 0.8798430023364486 0.8705512474696356 0.8802938084166472 0.8706074360273626 0.8800298213026101 0.876530400390625 0.8727422890664004 0.869962243718593 0.8800309788235293 0.869962243718593 0.8793906074214134 0.8766124442312305 0.8798186067101584 0.8782265020281557 0.8805139256007395 0.8704542363039637 0.880802935010482 0.876866351768336 0.8804470967741935 0.8711901417721519 0.8795332270168855 0.8709979259634889 0.8723500858895704 0.8714365794023214 0.8762361332032202 0.8795573112986403 0.8792249952516619 0.8790801688951444 0.8791385815939279 0.88111310110251 0.870813560222672 0.8799731575246132 0.8765750710900474 0.8904306872533085 0.8800866279895808 0.8762618689016762 0.8804360644554222 0.8788825816374822 0.8825677233294255 0.8800251606145251 0.8802905207373272 0.8800981759502748 0.8831391872710623 0.8788992260208929 0.8796828967428968 0.8794321744159149 0.8762622267595483 0.8794646429396812 0.8796238395746648 0.8806687550391272 0.8797854155124654 0.8797732564043388 0.8797772704199354 0.8853411994851994 0.8762633183004985 0.8746587233521196 0.8747189204406365 0.8761992380726324 0.8767563767430868 0.8762000071208167 0.8759772931242461 0.8764238534336327 0.8685469319640565 0.8686242802155504 0.8924264372469637 0.8757764490291262 0.8757024721009219 0.8766750343032884 0.8946397720469722 0.8919366110183639 0.8853961426456072 0.8907515294974511 0.8799759244486157 0.8799006501766785 0.882963027001421 0.8700724701789265 0.8683905814863103 0.8713409994874424 0.8769879656227926 0.8762988406827882 0.8768725343114057 0.8766008023668639 0.8762840839061389 0.8762582941316228 0.8713242943396228 0.8710775421383647 0.8713454837080071 0.871367266296109 0.8711813948228199 0.8707269686943242 0.8708782373827034 0.8770414828341856 0.8762146903304773

GCA_001525015 0.8755790669014085 0.9990543677187037 0.9600602650991242 0.9600924632103238 0.9581316508400292 0.9897875717488789 0.8837994925679139 0.9775177569666513 0.988646760758908 0.9891096557772419 0.8772570649038463 1.0 0.877035699106496 0.9601020031760436 0.9707272771200773 0.8840570231958763 0.999065493867011 0.9580960481927712 0.9990828667790893 0.9581599587778855 0.9769523369175626 0.9582344316270128 0.9926768102142394 0.958423282350085 0.9992965492205852 0.9582873264223721 0.9926305108225109 0.9581066279351247 0.9998650919493258 0.9580929925605951 0.9904135574544637 0.9581439015518914 0.9926386900889179 0.9894530724838412 0.9721237094531974 0.9583441062801932 0.972346954513969 0.9582393886358187 0.9926434228920925 0.9581895430622009 0.9724005794090489 0.9582110729716754 0.9708935672227675 0.9581001723312589 0.9926862668698303 0.9581383109082172 0.990795791925466 0.9580773613445379 0.9772724084868267 0.9581371373771278 0.988737952618996 0.9583463570914327 0.9722398245208959 0.9585005237866411 0.9927080789245447 0.9581549029475197 0.9891562588450126 0.9581724002883228 0.9889342949576082 0.9582138199279712 0.8779450198991775 0.9581178261910462 0.9773573260919807 0.9580556783919597 0.9722827891469303 0.958242688713156 0.9891133845046775 0.95809175035868 0.9721277970813064 0.9580856043956044 0.989221601110597 0.9581389529046139 0.9723600717758741 0.9581488140872065 0.9926747716845096 0.95833130184608 0.9926605075147028 0.9580289787132265 0.9889142876135609 0.9582139976387248 0.9887749093351242 0.9596883911007027 0.9891600255338905 0.9900218838967972 0.9925933384547848 0.9900218838967972 0.9989780894308945 0.9903557360627177 0.9926355062743402 0.9886193505317944 0.9991365383797576 0.9580529890267175 0.989289018254675 0.9717888163640542 0.9723714433940512 0.9706497907434762 0.9925963575005443 0.9707037191697192 0.970847640776699 0.9715325801011804 0.9899050652024707 0.9721455545265109 0.9891200542372882 0.9989409766277131 0.9891938725711705 0.977504688077634 0.9588059604519774 0.9926652472885032 0.9596474266418258 0.8817872099378885 0.9775251382380504 0.9592172096317281 0.972403168849392 0.9900770344517001 0.9890272282608696 0.9991062387483927 0.9905091212653778 0.9775025041380941 0.9896706241670369 0.9597425118708452 0.9889843249225321 0.9890347332300199 0.9719905017261219 0.9889726029216468 0.9890290203900709 0.9883495605646629 0.9890273663278834 0.989035260715864 0.9890242055248618 0.8780292449755567 0.9885627785175323 0.9593830335260115 0.9594307610455702 0.9885423507130127 0.9885772709119146 0.9885260374414976 0.9596066269477546 0.9892242988084325 0.9896143861712137 0.9896282189849624 0.8836133662363959 0.9597398051799221 0.9597640256762953 0.9885914862466726 0.8854459432799012 0.8848134937343357 0.8787547541414672 0.8855991662404092 0.9891333950892858 0.98917158918194 0.9896317738309353 0.990113143692377 0.9706797962690231 0.9507415960665658 0.9886908582834332 0.988523209519573 0.988787674366948 0.9887061408575873 0.988551589492431 0.9885448875027847 0.9763579626485569 0.9763718223620523 0.9764063962218456 0.9764071725974341 0.9763273758177853 0.9762002825133951 0.9763229011896093 0.9894031756450882 0.9893809002267574

GCA_015683495 0.9867465539803709 0.8809097461343179 0.8761337999515619 0.8780527385496183 0.8709405662311044 0.876140942622951 0.8921781150314162 0.8820660681976341 0.876970009699321 0.8817512342857144 0.9999804621698786 0.8788089749645724 1.0 0.8774465264164475 0.86921250380904 0.8919643920233464 0.8805807157845652 0.8716482445459158 0.8812932451701931 0.8718256985951469 0.8743206182531895 0.8705542366412214 0.8806419953596287 0.8708107444616178 0.8796374325908558 0.8713815858919057 0.8805086995096895 0.8710459943834568 0.8809162917439703 0.8712207709807888 0.8814635697620158 0.8714089210459507 0.8805616267163137 0.8690294671814671 0.8801377038075217 0.871513956687898 0.8807194275161588 0.8704271007121058 0.8806808169934641 0.8711584708249497 0.8811355629749023 0.8710753791887126 0.87297620486367 0.871121806743835 0.88067748193052 0.8709548397678526 0.880551019935095 0.870989072998232 0.8816934603622677 0.8710233568193257 0.8806774338319907 0.8704473180661577 0.8808238920913074 0.8708026311767704 0.8806861054342777 0.8705211173184358 0.8820702308054641 0.8712560030318341 0.8812447273149223 0.8707462096158737 0.8870281264165197 0.870487497456765 0.8817417554932212 0.8710443435613684 0.8809705879650896 0.8711124848790323 0.8741674477840041 0.8708507818411095 0.8822239786691398 0.8706711558801308 0.8740511160384331 0.8708850843191543 0.8810413427316847 0.871112393939394 0.8804665574929311 0.8704712252160651 0.8807323678696157 0.8709941803072273 0.8811352596353565 0.871052545958197 0.8809036576239475 0.8768579180804654 0.8734261137323069 0.8704709510664994 0.8809447225474127 0.8704709510664994 0.8800019266055047 0.877112138063279 0.8806429254701648 0.8793011205875385 0.8813433425541723 0.8709865528496108 0.8817388775746355 0.8774077966101697 0.8808529755872868 0.8716655750878954 0.8802991976608188 0.8716905988928033 0.872916840692852 0.8717796467991171 0.8768699027237353 0.8803645615259363 0.8800851190756898 0.8797051930727825 0.8800413935969869 0.8821065274212369 0.871205321239607 0.8807948949089212 0.8773069616380325 0.8910609854604199 0.8806518466981134 0.876844739195231 0.8811656027045931 0.8796434443141853 0.8830867831353365 0.8807361213107135 0.8811983912543153 0.8805755910390849 0.8839847183098591 0.8795258779165686 0.8804503723282003 0.8802167027151404 0.8768368360773086 0.8802769204231831 0.8803852344827585 0.8815202099056605 0.8805508882258435 0.880539784304727 0.8805359316200092 0.8859382082695253 0.8768640623523856 0.8749323661148978 0.8748954406037001 0.8768225950413225 0.8773526417313573 0.8768275200755786 0.8762639353293413 0.8771458337349398 0.8691246328984396 0.8692033188442855 0.8927648345935728 0.8760271073582631 0.8760027875572703 0.87728850718154 0.8948218988789751 0.8922490030783803 0.8862499313893651 0.8911524782818533 0.8804072826846889 0.8803556235404019 0.8835863700399155 0.8707114782608696 0.8689746044098573 0.8721681887950884 0.8777020501405811 0.876719740382346 0.877423929750118 0.8770734433962265 0.8767850672645741 0.8767718407372401 0.8721576041144005 0.8719431394182547 0.8722233971774194 0.872314234256927 0.8720942355889725 0.8715422899212998 0.8717986342943855 0.8772801890450799 0.8765146539961013

GCA_001526005 0.8750989029642767 0.960324318501171 0.9993444849648113 0.9993713319672131 0.9992255677002008 0.9599039388235295 0.8830328608313969 0.9601447000000001 0.9595282161771784 0.9600888319814601 0.8767403010279001 0.9607861776061776 0.8765903289958263 1.0 0.957914417560489 0.8821314263363754 0.9602946152024627 0.9987588780918728 0.960298407925408 0.9988561529859092 0.9583974604743084 0.9995351239306866 0.9603273012552301 0.9993687010676157 0.9606177132146205 0.9991869916188796 0.9603713397017708 0.9993954162976084 0.9608093681569345 0.9991533668561435 0.9602508038661008 0.9987788906145996 0.9603780405405405 0.9594421577669903 0.9598581773514273 0.9993490776053215 0.960217466168922 0.9996036871996506 0.960451690934711 0.99891063167066 0.9603402659822678 0.9991766892186819 0.9582753099530749 0.9989626487900588 0.960399347215723 0.998764263870686 0.960628210207411 0.9991043527863097 0.9603932098182676 0.9988469562363238 0.9600108785175018 0.9995327153188691 0.9600913959450011 0.999492555847569 0.9604504354913834 0.9994015739130435 0.9609340285714286 0.9989650602937953 0.9601789621976989 0.9993980209698559 0.8761778965702037 0.9994471264118159 0.9602200330110823 0.9988818671023966 0.9601782814677194 0.9989141840087622 0.9588082555780933 0.9991940008678672 0.9602839911152677 0.9991831332611051 0.9588342709613966 0.9992233507284194 0.9600916631677163 0.9991672120418847 0.9605464979995293 0.9995807372566178 0.9602743919550981 0.9991490808102811 0.9600824468333723 0.9989446979280261 0.9599990531286896 0.9986428251900109 0.958782589484078 0.9585400500357397 0.9604992112079116 0.9585400500357397 0.9603784213006598 0.9599447565197322 0.9603198931475031 0.9596926169107228 0.9604412218574109 0.99904361720617 0.9602029227508244 0.9588285197974217 0.9602769037656904 0.9577526069750186 0.9605445846943538 0.9575878976007915 0.9581714142156864 0.9581675512195122 0.9598511606714628 0.9599950491343004 0.9602962685163415 0.960259853692522 0.9602098542890717 0.960261894885694 0.9995385389823105 0.9603406915017462 0.9908069723953696 0.8793138266331658 0.9600591319772943 0.9985992166989455 0.9602122151300237 0.960054700591716 0.9605404024621212 0.9603756394525719 0.9599794756467023 0.9601093727641307 0.9609751162250116 0.9906724472190692 0.9600019001161441 0.9599226932899931 0.9598621347270616 0.9599438992104041 0.9599763002556356 0.9595747126162653 0.9601062812789621 0.9600862844567987 0.9601295295480882 0.876688581967213 0.9592210473289596 0.9993471913905887 0.9993421652962259 0.9589883035714286 0.9591732498830137 0.9590147040864255 0.9992344753476613 0.9599999158856044 0.9581981124597474 0.9582299232103049 0.8821402873855544 0.999260575795645 0.999266105638231 0.9592081679925129 0.8858137654320988 0.88212259627646 0.8768972670974691 0.8829255688935281 0.9604001236295779 0.9603867680074837 0.960594624794166 0.9578916838570399 0.9574416923459244 0.9446514307496823 0.9594512689880814 0.9592425239990635 0.9596706087567314 0.9594178136704119 0.9593053687177079 0.9593513303613328 0.9588028468780972 0.9588251508407519 0.9588107970297032 0.9588093466963622 0.9587719073569484 0.9586380915650661 0.9586487239324727 0.9601919796111902 0.9601158099762472

GCA_015683555 0.8690759444589992 0.9714100540540541 0.9582370812685828 0.9577154507389163 0.9584445810473815 0.971460974950884 0.8774153545904344 0.9720856269479741 0.9714998938009386 0.9711699422104502 0.8691336319426671 0.9718849688995217 0.8691792710472281 0.957636564190851 1.0 0.8781920352035204 0.9715230539070229 0.9583044433374844 0.9714236213190557 0.9584178214107054 0.9726268818484993 0.958407994049095 0.9714196893346378 0.9584599549662245 0.9717938862793573 0.9581998257839722 0.9713531433463796 0.9582784932533734 0.9713584350775193 0.9582753817550819 0.9724748873101422 0.9580167106245335 0.9714406386102278 0.9713366593191282 0.9913117420118345 0.9583839726027397 0.9925515038972949 0.9584241245039682 0.9715297787066633 0.9582088751545118 0.9925589608606089 0.9581888526211672 0.9925742233573253 0.9582409156149467 0.9714798846342662 0.9581378957816378 0.9722728595890412 0.9581542598561865 0.9722875608568646 0.9583307882994546 0.9707988399128118 0.9584044042605896 0.9925584457142858 0.9585577217391305 0.9714056161369193 0.9578248639287482 0.9723425924088317 0.9582958448959366 0.9707720757465405 0.9577958102081268 0.878312652291105 0.9578571555775416 0.9722480349344979 0.9580716559565002 0.992540534490635 0.9583498440594059 0.9726866558684276 0.9578938543209877 0.9915722976810223 0.9577423847102344 0.9728107071688943 0.9581890360850223 0.9925108243398394 0.9581944997523527 0.9715648891625616 0.9583661471754212 0.9714620868072585 0.9581258314801088 0.9707237890152431 0.958512737920937 0.9707249147173489 0.9575734486166008 0.9727778961417133 0.9712749068473264 0.9715257554133858 0.9712749068473264 0.9714943374969385 0.9712427546983184 0.9713643230694038 0.9712923434835566 0.9715769028935752 0.9581646681470517 0.9711076611730488 0.992654077046549 0.9925700503432494 0.9919313640661939 0.9712851708876322 0.9921945531315974 0.9926476986869386 0.9923733125433125 0.9725441065675341 0.9913715531914893 0.9714611832718327 0.9717120196926032 0.9714930118110237 0.9724758589774655 0.9583015044031312 0.9714250660469668 0.9578759217603912 0.8733909146503589 0.9721027210552028 0.9573154196157735 0.9925054435950891 0.9716273075036783 0.9717777673267327 0.9715495681145114 0.9710214795052147 0.9721305499631719 0.9722597276926817 0.9578500075056293 0.9707979484686436 0.9708353014098201 0.9926183830673145 0.9708499805589307 0.9708051311953352 0.9701994058911632 0.9708050958505218 0.970809359223301 0.9708304587378641 0.8776826429761243 0.9708732065875515 0.9576785643930067 0.9576946218280363 0.9708644732380722 0.9708702832929782 0.9708803148462098 0.9576782996550024 0.972199351552795 0.9714804157495722 0.9715081300415547 0.8760394926749865 0.9576867504311407 0.9577704781858516 0.9709062600048508 0.8776268869426752 0.8776660622317597 0.877227515444534 0.8797989316702821 0.9722599902319903 0.9722954750244857 0.9721847888367497 0.9710548930481284 0.9916242545198404 0.9481158348248531 0.9708626295399515 0.9705636658595641 0.9709394937015503 0.9708545456748244 0.9705317094430993 0.9706270877788556 0.972427620215898 0.97243115412889 0.9724071172718352 0.9724663961755332 0.9724204639175258 0.9723868471572731 0.9723971792985038 0.9720813437195716 0.9720755855855856

GCA_002264125 0.8908905609631147 0.8848731137416368 0.8828299036458335 0.8826114222683624 0.8771319538632574 0.8829164063704082 0.9959474015050909 0.8862736386386386 0.882532696053306 0.884904550373615 0.8917304832713755 0.8850201269759005 0.891564719940254 0.8824038841033673 0.8774061920714686 1.0 0.8844204567997911 0.8767144396435321 0.8847611242299794 0.8769533958673194 0.8802295020408164 0.8768317777777778 0.8849571432251417 0.8772319423868313 0.8845231388961549 0.877547500676224 0.885116830967742 0.8770398365198117 0.8849179871134021 0.8772803609913793 0.8837909018987342 0.8772703193773483 0.8849086573715467 0.877247612939208 0.8848556119556815 0.8765609915091755 0.8843823788431475 0.8766731135135136 0.8850770197300104 0.8766823948908993 0.8845595338541667 0.8771020263370062 0.8772890646304882 0.8766462231075696 0.8849910406419881 0.8767488514056225 0.8847009816584861 0.8771510183189655 0.8859766389826109 0.8766809949454643 0.8836816178376976 0.8768730414746543 0.8842310249739853 0.8772242954113495 0.8849525483621357 0.8771718640410034 0.8839945224425887 0.8772435453569511 0.8845418072603813 0.8771368262987014 0.9675336061946902 0.8771023252032519 0.8854448068006182 0.8765830425531915 0.8843963197508435 0.8768679090909091 0.8791334238227149 0.8762062850971921 0.8844991203104786 0.876260697737069 0.8786129549114331 0.8772889602612958 0.884783909422176 0.8773672528672856 0.885274085349987 0.876647431590355 0.8848717648578813 0.8769798121309714 0.8843992245641425 0.8769701459660297 0.8842521248025277 0.8837100780843311 0.878610955465587 0.8791082452374565 0.8851649153866181 0.8791082452374565 0.8849564772132007 0.8853116944373238 0.8849343383110195 0.8830808680203045 0.8852199251805987 0.8768791048728813 0.8845561846234311 0.8850929115853658 0.8844017221644122 0.8780427552409477 0.8849386645962732 0.8763621128680481 0.8772904260515604 0.8782565906623236 0.8844332102644671 0.8848501261585993 0.8837639974489797 0.8847136700077103 0.883996213814119 0.886098528272657 0.8764584241931109 0.8852124903225808 0.8835294930991218 0.9108335029354209 0.8870747480719794 0.8806993929712461 0.8848451060487039 0.8845034545923632 0.8846252820379518 0.8846671254219683 0.885421914408711 0.8868043621719928 0.8852075450164848 0.8837263570877284 0.8845621522580647 0.8845843896774195 0.8845140378140378 0.8847580572755418 0.8846249974213513 0.8859970870712401 0.8847356664951106 0.8847380905815747 0.8847948044261452 0.9965736040386304 0.8842528530633438 0.8821892902208202 0.8820807020773073 0.8839215400624348 0.8844971917098445 0.8842338367559137 0.8832878599323093 0.8832637271778822 0.8782684625887492 0.8783568787547787 0.9668329327478624 0.8831905231650182 0.8832064369310793 0.8844630424650441 0.9333707166430929 0.9674460400368154 0.9319980917008198 0.9329483620063 0.8843425493626594 0.8843257092731831 0.8852725677789364 0.8781757004310344 0.8771936140254003 0.8773069916201118 0.8846568276931025 0.8841971539859778 0.8846439087610161 0.8846764296794208 0.8839575461398492 0.884119093985941 0.8787362780514506 0.8784631848004374 0.8786238638228057 0.878584761904762 0.8785444499178983 0.8785244043022615 0.8783855278310941 0.8832132984018861 0.8831725530799477

GCA_015683565 0.877531593118923 0.9999667218160831 0.9582758421294171 0.9598483770114943 0.957042843857073 0.9884787764550265 0.8846550827182489 0.9781464215904042 0.9882901562146253 0.9889421236847757 0.8807626582867784 0.9988799021852238 0.8807416573295985 0.9596307685206554 0.9704225024414064 0.8848147517911976 1.0 0.9569092051030421 0.9998670695332171 0.9570405209876544 0.975820899976128 0.956770279316157 0.9918448778947369 0.956752532199271 0.9984464436105899 0.9563924427296842 0.992399951312447 0.95624313925279 0.9989049837662337 0.9566067044905842 0.9907519868421052 0.9564508085209391 0.9918196919831224 0.9894537928604978 0.9722258840388008 0.9573184945972496 0.9729335581862855 0.9567838279932545 0.9918762592671044 0.9564530353280462 0.9729464805716838 0.9564982868142444 0.9700905859842144 0.9564338402309913 0.9918712973544974 0.9562555456516504 0.9899251670568207 0.9564990214509521 0.9782678788561135 0.9564834256138661 0.988239491598449 0.9567576486161251 0.9728368156424582 0.9569856973938226 0.9918445745577086 0.9564279327731093 0.989376261682243 0.95662095249578 0.9886600129757785 0.9565042209358419 0.8803162145855195 0.9562439711191335 0.9775335604199241 0.9564244615384615 0.9728067914438502 0.9565472147327877 0.9882070938542162 0.9569136973972271 0.9726141062584421 0.9568613730255163 0.9877085629290617 0.95652489673391 0.9728903525713003 0.9565184347198846 0.9924497655084205 0.9568516783048397 0.99193560093102 0.9564864921968788 0.9887085541909071 0.9564341516245487 0.9885499369702239 0.9591822255884409 0.9882523934807919 0.9888688208304658 0.9918313697175621 0.9888688208304658 0.9985440041279671 0.9891547592756185 0.9918442598441778 0.9876520004411117 0.9999661313444402 0.9563510871130311 0.9887277756202804 0.9714197175011485 0.9728830975228744 0.9703263956904995 0.9917307690677967 0.9697956385021604 0.9700572301474085 0.9703200970414203 0.9889446762589928 0.9722950133570792 0.9888807367953839 0.9978122543352602 0.9883268903508772 0.9781893200626258 0.9569488102738066 0.9924296065157606 0.9591292106470859 0.8836911441090556 0.9773844303797469 0.9587584609878311 0.9729492965392446 0.989130803649001 0.9887971967357743 0.9999895864734301 0.9893006515342582 0.9774241517753539 0.9899257928942808 0.9589463121226196 0.9879771406860617 0.9879717470664929 0.9714535255964789 0.9879954988046078 0.9880385704975017 0.9872946291331546 0.9880093924929486 0.9879788050314465 0.9880188611713666 0.8777862665585293 0.9878351589403975 0.9583665996728208 0.9583328608187135 0.9877275824662387 0.9878162959700507 0.9877665206812652 0.9586024930555556 0.9879841593517893 0.9883520252868182 0.9883688865497076 0.8851359119183877 0.9586015848007415 0.9586071517960602 0.9877874851223275 0.8844846757090012 0.8840381460816639 0.8775176366517031 0.8843804799999999 0.9887595648332229 0.9886618671096344 0.9897634310080909 0.9886359514540876 0.9701946832966496 0.9498871802543007 0.9879734612839179 0.9877745601237843 0.9880820123702232 0.9881140840707964 0.9877674524546661 0.9878095837023915 0.9763655625000002 0.9756074916387959 0.9763214093637455 0.9763131268011528 0.9763510045662099 0.9760589816158683 0.9762160356025981 0.98913677462888 0.9890409472259809

GCA_002333465 0.870287895421436 0.9573264461201655 0.9984532297734627 0.9987756290253327 0.9986911279018834 0.9580465207044266 0.8760339357104268 0.9578082620066573 0.9579693609838437 0.9575430957575758 0.8711700050543341 0.9585692753966375 0.8710721328937356 0.998840405231561 0.9576986479151246 0.8758377777777777 0.957413155697446 1.0 0.9573565423728815 0.9992765310757795 0.9568252166504383 0.9991914240639461 0.9577971780028944 0.9989969274809162 0.9579885211267606 0.9990067074435 0.9580530902017992 0.9984663655595515 0.9584593815413892 0.9987840999174236 0.9584591923171633 0.9989662068965518 0.9578584907027288 0.9592082405184832 0.9572214889975551 0.998931732942654 0.9577817707317074 0.9991431400460541 0.9579621952992489 0.9993648613640963 0.9578028874269008 0.9989458964307819 0.9575022676399028 0.9993833851119629 0.9579308500847664 0.9990126345724153 0.9579228875968991 0.9989540499277904 0.9572069793713164 0.9992605302570331 0.9573176063703351 0.9990797794580971 0.9576653516670723 0.9992144743481918 0.957882974927676 0.9988129350104822 0.957805696233895 0.9992834452405321 0.9575415790760206 0.9987429471458774 0.875008205402514 0.9988231762726126 0.9573348318713449 0.9993026318981201 0.957678148238153 0.9993266097560977 0.9579225903614458 0.9985329338842973 0.9575853195121953 0.9984644075144509 0.9578811597748961 0.9990733465305272 0.9576917029074029 0.9989950813516898 0.958204616891065 0.9991041248418389 0.9579516223404255 0.9989992143150965 0.9573282509135201 0.9993481926494346 0.9574032680538556 0.998495076335878 0.9579624577723378 0.9578200522813688 0.9580431737545564 0.9578200522813688 0.9579770571010249 0.957795744069015 0.9578562680810029 0.9574126597789525 0.9573919290573372 0.9993927648006445 0.9576256436612824 0.9557330801886792 0.9577182964224873 0.9569177905273438 0.9579907656893627 0.9560082294445792 0.9574906047411708 0.9573094472727273 0.9579633227228209 0.9573199731117087 0.9573979616766467 0.9577300146270114 0.9574040971059554 0.9572698311720087 0.9983171270140198 0.9581509601361206 0.9898663980716252 0.8736614806141034 0.9576232542206999 0.9968019673577415 0.9576843451501725 0.9567731515598132 0.9580845836422042 0.957483558116724 0.9575096050399807 0.9576809698914117 0.957994956542733 0.9894438733705773 0.9574568085618087 0.9574247113997114 0.9575123571945048 0.9574171545301612 0.9573941798941799 0.956965462871287 0.9573451031669866 0.9573488507677543 0.9574047191550648 0.8765012749658003 0.9574316375968994 0.999016045562003 0.9990323899506119 0.9574665900290417 0.9574565012106537 0.9574873184898354 0.9988769166666667 0.9578877936117938 0.9574588140161724 0.9574831178055351 0.8762738236856081 0.9990231772287862 0.9990267934665807 0.9575222539990306 0.879792787053686 0.8762749960306959 0.8769023705073995 0.877641104525862 0.9589313245655237 0.9588802799341332 0.9580586614557891 0.9578287247204379 0.9570699188989924 0.9441741264222502 0.9576823663198644 0.9574993799951562 0.9577536745088528 0.9576585658820677 0.9575208163759691 0.957497481193885 0.9566585128205128 0.9573516425594508 0.9577612099556433 0.9578138955922186 0.9566617647058826 0.9579656396931452 0.9577926040896771 0.9578648463996068 0.9578598156342183

GCA_015683595 0.8776600959409595 0.9991726515297288 0.957933854214123 0.9594046508983398 0.9565638683127573 0.9881530243690166 0.8850202334923425 0.9775003256325633 0.987552858420935 0.9885089330234523 0.8809728091181213 0.9986912975206612 0.8809414080924856 0.9593056055758684 0.9701024631018631 0.8845851751953617 0.9991524912075029 0.9567251498903242 1.0 0.9567862606994375 0.9751901393481341 0.9563935900502273 0.9915572630701937 0.9563610793804453 0.9979159573158219 0.956020340527578 0.9922251948051948 0.955837395808239 0.9985190845352564 0.956300268456376 0.9906523229461756 0.9559624837545126 0.9916133896537338 0.9888279744483159 0.9718382441253263 0.9569757428641131 0.9726465797037364 0.9564171349928197 0.9916283728742389 0.9561364495347173 0.9726597194610118 0.9562466331417626 0.9700109645153607 0.9561349438738954 0.9915687025780759 0.95605765748503 0.9897994308082946 0.9561779947292764 0.9779997399422093 0.9562275340827554 0.9877757203479738 0.9564449892318737 0.9725270813079981 0.9566286426858515 0.9915798581264345 0.9560009806728705 0.9887865591397851 0.956276453390846 0.9883034891700622 0.9560035032304378 0.8807348936721993 0.9560455847883284 0.977083991615181 0.9561451503579952 0.9725543703214442 0.9561800023894863 0.9874398678310126 0.9565258538350219 0.9719438003113186 0.9564970040973728 0.9868588567555355 0.956230847659981 0.9726180554938957 0.9561916642754664 0.9922184569858382 0.9564578631251496 0.9915677896277707 0.9561234287759486 0.9883773486786019 0.9562022272618763 0.9882322124084447 0.9588064522821577 0.9874517474167624 0.9883160489352062 0.9915711349757844 0.9883160489352062 0.9981609858866842 0.9884761327529925 0.9916401145594668 0.986595162132753 0.9995781153846154 0.956121660710031 0.9882762818045757 0.9710503631411712 0.9726466137566137 0.9699251633986928 0.991512163239614 0.9690407503539404 0.9698709662974311 0.9696152372049543 0.9885841269487752 0.9719075483304042 0.9879423194748358 0.997728910688739 0.9874474041170099 0.9779853824049514 0.9565340532757379 0.9922706701570682 0.9583458719746665 0.8841665173410406 0.9768292929061785 0.958442381857041 0.9726859588734912 0.9886967808952668 0.9880760373669347 0.999160627313462 0.9891065445462115 0.9768115959409595 0.9887209300341298 0.9584220032272935 0.9873143116716545 0.9872104666095892 0.9710611802378774 0.9872013720034246 0.9873625903743316 0.9865321128925983 0.9873509628522631 0.9873361571306578 0.9873486653854368 0.878489229747676 0.9870569180470794 0.9578478705202312 0.9578115640906988 0.9870262805410123 0.9871103322475571 0.9870581584115209 0.9580002658721064 0.987117733924612 0.9876979612098822 0.9877135902168896 0.8853829361389772 0.9581229426605505 0.9581208109965637 0.9871058451163803 0.8848154339346661 0.8844336302562827 0.8778743967758711 0.8843986502912129 0.9882502292576419 0.9881461041803458 0.9893617878064656 0.9880051583710407 0.9698199733913885 0.9497980029585797 0.9872791646726126 0.9870611675016336 0.987378873392896 0.9873885390318361 0.9871002765679444 0.9871051036891509 0.9754563820171267 0.974833231678487 0.975505231646472 0.9755088783269961 0.9754761251189342 0.9751848576214406 0.975407011904762 0.9883164231961044 0.9880719165001111

GCA_002333485 0.8700430087809917 0.9573666948130277 0.9986127571489545 0.9987260084925689 0.9987999071073667 0.9581912169187146 0.8763322516733603 0.9580213605281773 0.9580694601051123 0.9574260394610202 0.8708292218875501 0.9587031151771054 0.8707915152277877 0.9987757779664613 0.9576675250427559 0.8757865072655218 0.9573895695525292 0.9993894964610719 0.9575028794428434 1.0 0.9572544053923929 0.9992160462692791 0.9576684162679427 0.9990644891965598 0.9582347890040994 0.998929 0.9578857745121658 0.998629668624161 0.9583829444967075 0.998866217817736 0.958370348865577 0.9989533875890133 0.957735002394636 0.9591636021759699 0.9570396734397677 0.9988974984443061 0.9575767172447013 0.9991919663970131 0.957869036840838 0.9993248369021415 0.9576970647724536 0.9990110206164522 0.9575319142309535 0.9992764211369095 0.9578288926255105 0.9990726297786721 0.9579752798462646 0.9989371326530614 0.9575719722425129 0.9993204671210906 0.9573150317871439 0.9992738871538621 0.957426051491819 0.9991274610106052 0.9577600885379277 0.9988614051473641 0.9577860338069575 0.9990862725613465 0.9574720596074631 0.998854407098121 0.87513328 0.9988759053969578 0.9575983574529667 0.9992069887955181 0.9574723330129747 0.9993105168946099 0.9583833739130435 0.9983938433217426 0.9574824094640272 0.9984274258236137 0.9581253032508491 0.9991757882789931 0.9574414071928554 0.9991292217175888 0.9581375424551191 0.9992600292825767 0.9577431533477324 0.9990560500610502 0.957297618357488 0.9993796262242655 0.9575895673310646 0.9987628335932278 0.9580453286441912 0.9579840706713781 0.9579102749638204 0.9579840706713781 0.9581474794586758 0.9579429501187647 0.9577493492822967 0.9576842136569118 0.9575580781287678 0.9993792210904734 0.9575924526469162 0.9556296355991591 0.9575138298894764 0.9572080529254673 0.957889853506244 0.9557321186644248 0.9574586308243729 0.9574130216346154 0.958053518833536 0.9571804069767441 0.9575514865827595 0.9577606758387641 0.9575319468186134 0.9577467062530295 0.9983881968911916 0.9580334031791907 0.9898841243452516 0.8738802141935483 0.9578764979413902 0.9969965117801047 0.9576405677387915 0.9570430668286756 0.9578889802390826 0.9574842368357196 0.957529569608079 0.9579001708984376 0.9582253836930456 0.9896215138888889 0.957263538791052 0.957218134665715 0.9570588666666664 0.9572341736028538 0.957197288740776 0.9566851728364795 0.9571509337134712 0.9571489662547529 0.9571620313613686 0.8764406970436671 0.9575426344860712 0.9990678883443427 0.9990904770017036 0.9575658665386462 0.9575751069454458 0.9575646301633047 0.9988528619885522 0.9581363237445147 0.9576592912621358 0.9576829296116505 0.876491664468478 0.9990836832020439 0.9990365146024303 0.9576168517183369 0.8799982284263961 0.876466544736842 0.8769335772145285 0.8776410326519777 0.9592364011180994 0.9591738567428838 0.9580177715944833 0.9579478314104074 0.956985462082419 0.9440038638638639 0.9576788091236494 0.9574605451488952 0.9577799423076924 0.9576094874879693 0.9574067483189243 0.9574175072184794 0.9567902585790238 0.9574052812803103 0.9578753195121951 0.957899922046285 0.9568037342995169 0.9579581110839246 0.957867068292683 0.9578599780648307 0.957855708018523

GCA_015683615 0.8725695695618755 0.9761272873726604 0.957750041013269 0.957974062123766 0.957687085995086 0.9778885354367788 0.8818356071805703 0.9988267782884659 0.9772625071839081 0.9773879288990825 0.8746221012533792 0.9774379616643292 0.8746203743842366 0.9578383293211363 0.972232906949352 0.880671873168132 0.9763047426120115 0.9573727825030378 0.9762940808650682 0.9574236465706615 1.0 0.9576749335267101 0.9755883956716066 0.9578732885742187 0.9762644433872503 0.9572719448476051 0.9762306982248522 0.9574694248111137 0.976839733460257 0.9573207777510234 0.9789198655660376 0.9573006532297232 0.9756283851224105 0.9775978106078954 0.9720396294580824 0.9578861995597946 0.9720518017806936 0.9576632858178303 0.975675069591885 0.9572240263788969 0.9720516760168303 0.9572238820697956 0.9719410398126465 0.9571905114045619 0.9756800637544274 0.9571713793103449 0.9776267361273706 0.9571364197828709 0.9986724773880434 0.9571380364858378 0.9769447498270694 0.9577936844648466 0.9719206543584951 0.9578053360735009 0.975634274611399 0.957402067723343 0.9774199710982658 0.957411454413893 0.9774651476400837 0.957503018322083 0.880440891635981 0.9573852321385614 0.9982738004484305 0.9571877937649881 0.9718624195804196 0.9572557894736842 0.9772430634730539 0.957595337837838 0.9720333640226629 0.957445456296653 0.9770351456536938 0.9571588458764125 0.9719036446416064 0.9572744439094848 0.9762776945039258 0.9578112542290963 0.9757019179051664 0.9571999136069116 0.9773831363004173 0.9574462884888678 0.9773648380330925 0.9580319070785697 0.9773449051300069 0.9772482921665492 0.9756294457603032 0.9772482921665492 0.9763502293358814 0.9771291770334929 0.9756359205266871 0.976530789038507 0.9764634979910187 0.9571569659961685 0.9775830023201856 0.9714414502729648 0.9720963848668847 0.9714428205741628 0.9756672504130282 0.9712436944642434 0.971856655791191 0.9718277429173494 0.9780372416285233 0.9721825697674419 0.9767354814289094 0.9775325797438883 0.9765342419962333 0.9987048285651889 0.9575276684924983 0.976277657764122 0.9570094933712123 0.8793383646044625 0.9990713399339934 0.9577018588002963 0.9720278697806086 0.9775200376205032 0.9776613567110036 0.9762860242972845 0.9758599765807963 0.9990606966940315 0.9775260551948052 0.9579013360616273 0.9762014761684406 0.9761964012959962 0.9709279193205944 0.9762384501503586 0.9761396046242776 0.9755298885199242 0.9761857598152425 0.976162604479335 0.9761720133918264 0.880688948076406 0.9768455696787612 0.9576771118087419 0.9576589430501932 0.9768326720554273 0.9767980166435506 0.9768135011547345 0.9576349782608696 0.9769131497005988 0.9772885519060178 0.977303572113081 0.8795776900815575 0.9576934194638976 0.9573506815992292 0.9767482501155803 0.8818743535687072 0.8803172998430141 0.8794547202887342 0.8804868039884545 0.9784714113836188 0.9783577411873841 0.9779311538461539 0.9760155566104914 0.9719804957882069 0.9517065123762376 0.9772181617987946 0.9770283873957368 0.9773429015784586 0.977290354659249 0.9768656661279149 0.9770869177288529 0.9851815552460538 0.9852036533579505 0.9853790674418604 0.9850990171933086 0.9853678810408922 0.9851957845651668 0.9852435083643123 0.9774866618739516 0.9774526845637582

GCA_002333505 0.8697737917433606 0.9569056275795095 0.9982060729706389 0.9995716713635379 0.999180535161433 0.9583093319169028 0.8762805014990461 0.958578729742612 0.9589487120291617 0.9577358950465914 0.870189883086516 0.9587970626195029 0.8699652537080407 0.9996728487994808 0.9578264619225968 0.876093373851972 0.9568807216242662 0.9994336156980486 0.9570447764080251 0.9995882214983715 0.9574899803391499 1.0 0.9580708627833293 0.9996030075503356 0.9580833914538311 0.9996002939949957 0.958338019169329 0.9994985729454237 0.9587016294696425 0.999664686523641 0.9582889018633541 0.999465813068302 0.9581437084960939 0.9593096806966619 0.9565400441501105 0.9994860101975781 0.9580718545994066 0.9996420556127703 0.9581743434590887 0.9996089344432882 0.9581301312205992 0.9996725985068436 0.9573250136645963 0.9995572679983356 0.9581713145654834 0.9993529568315173 0.9577580688151199 0.9995633395599833 0.9576878230616303 0.9995131227850741 0.9573518714011516 0.9995762867799419 0.9579858250988142 0.9995775270157938 0.9581755308762511 0.9995158222866611 0.9578924309529734 0.9994821629567407 0.9578159584980237 0.9995270556252613 0.8753765411324786 0.9996115288481566 0.9572790372366486 0.9995089731305978 0.9579981593881075 0.9996238942509443 0.9584129941639178 0.999538586683417 0.9580069367588934 0.9995206869910211 0.9575919853479854 0.9995930746582545 0.9581139563167039 0.9995436952861952 0.9584359723251791 0.9997140066500415 0.9581933235366153 0.999703044375645 0.957635596149099 0.9996956321599667 0.9578189724497393 0.9993980168296567 0.9581778809582613 0.9578470839328539 0.9581945637748834 0.9578470839328539 0.9583243209267933 0.9576593038433647 0.9580434373476354 0.9576618929535969 0.9570524927184466 0.9996082777202072 0.9579197847600199 0.9559680400572246 0.9581191207705606 0.9567346170948617 0.958382917278512 0.9569324404030476 0.957278436651025 0.9575538727624577 0.9577925875152998 0.9574811265508685 0.9579810961255517 0.9581369133974835 0.9573109529582929 0.9576625753830946 0.999467388345385 0.9583898403340703 0.9902130348027842 0.8717497557738254 0.9581726877175534 0.9986461452513967 0.9580964237584227 0.9573354637645315 0.9582250074812967 0.9568704132029341 0.9566690452018369 0.9581674881160872 0.9583413878048781 0.9898114420358153 0.9576095822200631 0.9576412988589463 0.9575405185004869 0.9576279286061196 0.9576197595920352 0.9571975975975977 0.9576674890829695 0.9576316642406599 0.9576852401746726 0.8763285131034484 0.9574994146460936 0.9997433847155882 0.9997288438177875 0.9575314561918746 0.9575342111709945 0.9575413680861479 0.9996733064164122 0.9579286457819437 0.9575418739184178 0.9575806913580247 0.8761820463629095 0.9997281579518333 0.9997404951140065 0.9575406367866766 0.8796895283260703 0.8758864985163204 0.8755482025792586 0.8764317684095265 0.9591882504165676 0.9590385120611415 0.9577588236732697 0.9575932517985613 0.9573057898658718 0.9441837381501408 0.9578139843367596 0.9577012013701982 0.9578723290022064 0.9577815799803728 0.957716013712047 0.9577709021819074 0.9582201096983296 0.9578179781962339 0.9581483731938216 0.9582024433938792 0.9581540512948208 0.9583255561109723 0.9581763063511831 0.958012082198564 0.9580115895023522

GCA_015683635 0.8767034694382578 0.9917120570824524 0.9589147345337027 0.9594981366742598 0.9571711148977604 0.9883230250990752 0.884439850101626 0.9769299022439458 0.9875811230907457 0.9889504660029655 0.8801119845541774 0.9923692612305728 0.8801007623739152 0.9594138591678097 0.9697803004603829 0.8844011997966446 0.991697902605724 0.9568303673965936 0.9918211271434547 0.956976418820088 0.9745311429247516 0.9571520154851199 1.0 0.9571185198821798 0.9920553374363329 0.9569747198641767 0.9998475083382382 0.956965468292683 0.9921585920804524 0.9570165667311413 0.9899972120817035 0.9568448902439025 0.9998557961658842 0.9883390160502442 0.9715879312638582 0.9572537151929653 0.9719943215405284 0.9571590590227383 0.9998466285488958 0.9569289723225031 0.9719596891075822 0.9568973665136506 0.9693452298850573 0.9568171628131021 0.9999653261927947 0.9568385814403095 0.9901630233554746 0.9568434374243525 0.9775102747747748 0.9567341961918534 0.9881709135226792 0.957118545498548 0.9719907152436299 0.957216063030303 0.9999703430897461 0.9567823203087313 0.987833978118162 0.9569641283292978 0.9884677841880343 0.9567688918461166 0.8795422993948961 0.9567242487922707 0.9767640455967813 0.956802258064516 0.9719444451871658 0.9569263441119691 0.9867097804532577 0.9567869254883048 0.9718377074479981 0.9567159003370246 0.9871793999083829 0.9568580809053695 0.9719417080814866 0.9568720014485758 0.9999722847551342 0.9571601569669163 0.9999499727626459 0.9569093903614458 0.9885247536108751 0.9568057596917892 0.9883770262140096 0.9583274435575828 0.9871934954128438 0.9886064045454545 0.9997916161616162 0.9886064045454545 0.9920041751269036 0.9890270812349463 0.9998559766081871 0.9872276234364715 0.9919469442096366 0.9567866154584733 0.9886066737649063 0.9706256907449209 0.9719846589129949 0.9690301738290681 0.9997118299881937 0.9689004102074888 0.9693384549560257 0.9696791684434967 0.9886046873604287 0.9716688847255895 0.9877254579234973 0.9915333179142137 0.9875925202009173 0.9776298030438676 0.9569965971722982 0.9998460416666666 0.9583053919563735 0.8830264893617021 0.9765874154478976 0.9582977556818182 0.9720163474135985 0.9884817260747462 0.9886520373626374 0.9917065382978725 0.9898898165529011 0.9766269799122606 0.9878741161400513 0.9586345981524248 0.9879840094238596 0.9879209927101201 0.9703839092970522 0.9879244639794168 0.9880472191252144 0.9873474845679013 0.9879695035309223 0.9879770960838862 0.9879835830479452 0.8774663697705805 0.9874869242490205 0.9582377497687325 0.9582438594869425 0.9876168453159041 0.9876681401909723 0.9876207430812813 0.958568108232057 0.9879309520609318 0.9874311141741328 0.9874447315282241 0.8837343605086013 0.9585649816513763 0.9582507102078102 0.9876837877143477 0.8838576456071077 0.8840392667001506 0.8769008624769921 0.8843367175961294 0.9874504957599478 0.9874448289387665 0.9891005113141862 0.98847172398087 0.9693361306653809 0.9491216483516484 0.9876982707745715 0.987433699912892 0.9877770735422107 0.9878232164634146 0.9872034022188383 0.9874530975769481 0.9749157963757749 0.9744909577999054 0.9750068827454719 0.9746373087008344 0.9748436779418771 0.9746614206995688 0.9749594986870375 0.9878849410980217 0.9877358489304814

GCA_002333525 0.8700883159812597 0.95687315915628 0.9975549978804579 0.9993152411575562 0.9987525688869603 0.9581218844630794 0.8769614697950376 0.9587030792754646 0.9588348359281438 0.9575847314949202 0.8702766991286519 0.9586012671071259 0.870137968147958 0.9994036431784108 0.9576946553421961 0.8766118417535418 0.9568244945717733 0.9991343378321383 0.9569560242972844 0.9991044434913147 0.9574190479651163 0.9994622210849541 0.9576091258405381 1.0 0.9579311596312471 0.9993478285009255 0.9578756063907045 0.9990012659819745 0.9586684091986724 0.9993914663116936 0.9579158107116655 0.9992020825515949 0.9576614653512994 0.9591362282582797 0.9565519720009654 0.999283690152624 0.9579279902676399 0.9995125692182412 0.9577094272595457 0.9993436169338266 0.9580325261875762 0.9992259562282676 0.9574971926380369 0.9993332407978615 0.9577361187545258 0.999142829096921 0.9573550536352801 0.9993088267813268 0.957916140651801 0.999267013760526 0.9572362349040965 0.9993270032706459 0.9578533592799805 0.9993028442807449 0.9577061856661857 0.9993258133824433 0.9577063596276336 0.9991420406049306 0.9576389690470388 0.9991395947901593 0.8757583755274262 0.9992777045126725 0.9573407279322854 0.9992187451222015 0.9578122594752188 0.999144219426686 0.9580992424619986 0.9993451962809918 0.9579266642300195 0.9993360268595041 0.9571385975463075 0.999246393442623 0.9579348412310698 0.9992186082795922 0.9580173339819994 0.9994171654027466 0.9576273110255176 0.9994467401060791 0.9574682920310983 0.9993350440483508 0.9575913752743234 0.9990805461901551 0.9577005255474454 0.9577031198298275 0.9577301018181819 0.9577031198298275 0.9580121576258819 0.9575590061963775 0.957627095844343 0.9572081391976801 0.9569955717703349 0.9993412492320295 0.9576952257749572 0.9555562335526316 0.9580127841323923 0.9568373453482708 0.9577968030813672 0.9568254587267007 0.9574452022417154 0.9573049012139968 0.9574770304200869 0.957567581827064 0.9575096384671005 0.9578760874848117 0.9568954235266045 0.9577867202337472 0.9991913446256796 0.9579120498547918 0.9899190274599542 0.8725290213101815 0.9583714499142787 0.9984163768434955 0.9580722164821647 0.957062391994142 0.9579628876873925 0.9569057094675982 0.9565111904761905 0.9583603853754941 0.9579240120192308 0.9895725221033039 0.9574965963060685 0.9574689832134293 0.9573298822115385 0.9574880882705685 0.9574937769784174 0.9570197924388437 0.9574448922413792 0.9573853039731928 0.9575026209870628 0.8766981294374658 0.9572957904899831 0.9994070418904406 0.9994046457707171 0.957304156000966 0.9573215423606084 0.9573223190154441 0.9994083956756759 0.9575146123936817 0.957475647087497 0.9575039800146236 0.8765481832501969 0.9994092310996563 0.9994147345798409 0.9573615031416144 0.880129987283825 0.876352167199148 0.8759462390438246 0.8770256152185646 0.9590047716092762 0.9588390472829922 0.9576012460833936 0.9574912952877103 0.9569748393426539 0.9440317392405063 0.957584850458273 0.9575057714561236 0.9576254119352501 0.9575445921084483 0.9574510115886046 0.9575467988394586 0.9578117683226759 0.9573480669599219 0.9577791789577188 0.9578216699410609 0.9577678343166176 0.957909129684418 0.9577558806190125 0.9575728310168252 0.9575531007562822

GCA_015683655 0.8764228607089366 0.9983304568003308 0.9595915829669059 0.9601517372495447 0.9582340901339831 0.9890353686990975 0.8856753528965343 0.9782804255798244 0.9890254265510948 0.9895506592226755 0.879361020696143 0.9994054131113425 0.8791973457371642 0.9599830237877401 0.9709733113553114 0.8846069370522007 0.9983228131545873 0.957786032635168 0.9987433606221859 0.9579179398091511 0.976071083849452 0.9578333647798742 0.9925718513827317 0.9579385125579128 1.0 0.9578738616995375 0.9928459639065819 0.9576745643153527 0.9991954785478548 0.9578022782355794 0.9904182193576578 0.95780306640625 0.9926005771670191 0.9894651815642458 0.9733580791722898 0.9578488510223953 0.9731037398190046 0.9577460638297872 0.9925608789808918 0.9576428768115942 0.9731618945708493 0.9578956166707052 0.9711683983622351 0.9576794902150279 0.9925292898028409 0.9577107643775783 0.9901930105900151 0.9577497961165048 0.978480346781505 0.9577726742497581 0.9890286854054054 0.9578609049116865 0.9730745442238267 0.9578762969241946 0.9925922374815127 0.9578125211915719 0.9892631119964587 0.9576825490671189 0.9895427616974972 0.9578048834951457 0.8799136303543098 0.9578271124031008 0.9779283966530982 0.9576382999275537 0.9731251507650767 0.9577536974383761 0.9895038675213675 0.9577218353054818 0.9727015302893309 0.9577014541062802 0.9889667331042382 0.9577613771442378 0.9731076611626329 0.9577749975798644 0.9928312280701755 0.9577905926463474 0.9925204768969903 0.9577363741841914 0.989551798483207 0.9577425531914894 0.9895425027370265 0.9598348798521257 0.9891675472132658 0.9891870951509607 0.9925049360613812 0.9891870951509607 0.9998272155505801 0.9894419032967033 0.9925389594765723 0.9891384118169703 0.9987359636438753 0.9575829253012049 0.9896678804347828 0.9721633248847925 0.9731334159531321 0.9706031500241196 0.9924452671755725 0.9708488758107134 0.9711161229370963 0.9716444220748461 0.9895100112283853 0.9733168995731296 0.989541399339934 0.9988662344656173 0.9895339753737907 0.9785112195121951 0.9574482314725699 0.9929320812074831 0.9598940249433108 0.883157970417071 0.9773735049413929 0.9592031680113907 0.973157190346084 0.9893120612691466 0.9894150832039051 0.9983288689107255 0.9903643328303514 0.9773311772885284 0.9895615546400695 0.960108080574207 0.9890137674020133 0.9890306307659301 0.9721330108269984 0.9889875717959709 0.9890214533762058 0.9883519017188189 0.9890264626578216 0.9890067693624305 0.9890213695698694 0.8785170913978495 0.9883545892661556 0.95897001394376 0.9589397513362771 0.9884659034028542 0.9885042897727273 0.9884799232624424 0.9591430662983426 0.9890357110812024 0.9886637494220989 0.9886821293302539 0.8851913284963723 0.9592487998157107 0.9589750598526703 0.9884443150984683 0.8850308074534162 0.8843850000000001 0.8780924809861002 0.8845462164922135 0.9887913004385965 0.9887699098108226 0.9899373628552545 0.988841705479452 0.970472625548513 0.950254834834835 0.9886003893263342 0.9885243023511318 0.9886815878230398 0.9885889278666958 0.9883065876152834 0.9885493509350936 0.9764286580397795 0.9757621108995718 0.9764813461077845 0.9764898659324874 0.9764429214559387 0.9756954440154441 0.9763117771373679 0.9894083400089008 0.9892842394460577

GCA_002333545 0.8695644129133038 0.956729882381181 0.9974862317614718 0.9991741882908 0.9985068739168113 0.9586165589130944 0.8766171652691371 0.9584062770970784 0.9587173323720259 0.9578424830261882 0.870571978582356 0.9585416038853353 0.8704254336147353 0.9992050128479659 0.9575139122936684 0.876635222963952 0.9567180837164289 0.9992004943443654 0.9567653280839895 0.9992644211640211 0.9574445072815534 0.9995343274372686 0.9577158116570327 0.9995337003947643 0.9582126322203267 1.0 0.9579878341013824 0.9990520033181253 0.9584823292886034 0.9991743527738265 0.9583634227362205 0.999305862139497 0.957792048192771 0.9590601170848266 0.956623039476871 0.9991001360117179 0.9579887325666748 0.9997069424534099 0.9578734068330508 0.9994287228426396 0.9581895161290324 0.9993249272139103 0.957329000245038 0.9994408612634574 0.9578131615868409 0.9990793280228758 0.9580017959183674 0.9993142382495949 0.9576687235612396 0.9993568578401464 0.9575393255758727 0.999561686201933 0.957911046596731 0.9996242777777777 0.9578273993733432 0.9991435075850759 0.9581258948407801 0.9993327254056275 0.9577963422982885 0.9990795699813935 0.8755408878876226 0.9990845238587069 0.9571617557436518 0.9993015034538806 0.9579033852898198 0.999244752293578 0.9582876438080884 0.9990827046482929 0.9577457003177707 0.9990028069815194 0.9576712345679012 0.9993855272876487 0.9580173445912873 0.9993573909465022 0.9581540792369773 0.9996710006189397 0.9577799758278946 0.9994342351046699 0.9578802169673329 0.999454225924421 0.9578749705882353 0.998536759363086 0.9581796474045053 0.9576731086853347 0.9580048968696917 0.9576731086853347 0.9580821604486711 0.9576994240460762 0.9577343706377858 0.9571323839458415 0.9568149520613614 0.9993966626189033 0.9579879711209007 0.9554167051614423 0.9580384869225128 0.9570633945628216 0.9580063455953534 0.9569712472647701 0.95727552355382 0.9573409734513274 0.9579383924235064 0.957397678396872 0.9575319207538052 0.9578777160944729 0.9567270367717288 0.9579227613219095 0.9985210213383054 0.9580775302956859 0.9902551503327978 0.8725549788471709 0.9580343095823096 0.9983086488869296 0.9581294981458591 0.9574366895033032 0.9580375191310787 0.9567938708897485 0.9566317921146954 0.9580676567901233 0.9585357253384913 0.9897008523521191 0.9575668520738431 0.9575478672417924 0.9573126724553787 0.9575704027811076 0.9575703930968361 0.9570415918065153 0.957530538664113 0.9575039731929154 0.9575447498204452 0.8767338438356164 0.9576670937272949 0.9992915435809362 0.9992800729613734 0.957678617098571 0.95766429886171 0.957666793320426 0.999289463404164 0.9580794012707723 0.9572923758257892 0.9573277573978969 0.8763477022058822 0.9992807834299207 0.9993271938117749 0.9577030370549772 0.8800578764675855 0.8759129452418927 0.8762763580901858 0.8773550936736356 0.959544086833412 0.9593674121557454 0.9575954813739719 0.9574745402708481 0.9568045329737682 0.9437835093011563 0.9580279796757804 0.9577714611097651 0.9580418725775194 0.9579673907766989 0.9578173467409742 0.9578218695441318 0.9574575577395578 0.9576999067026762 0.9580027355698075 0.9580191270036992 0.9574203632793323 0.9582022789199901 0.9580020744943265 0.9580965062607415 0.9580965569744597

GCA_015683715 0.8765368052516411 0.992357519949601 0.9590212574302698 0.9592765799506617 0.9572349601930036 0.9886390398432399 0.8844436009975061 0.9768697036223931 0.9876938620230702 0.98920002507837 0.879924443670151 0.9923543898340685 0.8799811947931194 0.9591932191935121 0.9696943195407798 0.8843429710144929 0.9923759081242025 0.9570297420443588 0.9924671837411861 0.9572758879184862 0.9750591404415218 0.9572835076775432 0.9999757676669895 0.9574065417376492 0.9922006655433867 0.9572099061823428 1.0 0.957134003868472 0.9923270078577338 0.9572519333173424 0.9901051559829059 0.9572057464311639 0.9999924179452322 0.9885909366391183 0.971528997378768 0.9573921915820031 0.9720880507726269 0.9573002158273382 0.9999919793089987 0.9571673662846228 0.9720660061714789 0.9571143971291867 0.9699178243371211 0.9570743212237094 0.9999656282875512 0.9570531152647976 0.9901447633136095 0.9570407328207593 0.9774596886120995 0.9569972194304858 0.9887679869993709 0.9573061531077514 0.9720374498567336 0.9573214869084795 0.9999696759169416 0.9569586551806653 0.9881761809153713 0.9571324903938521 0.988742730145355 0.9570074520613615 0.8798853985507247 0.9569824820488272 0.9767662119205299 0.9570950787589498 0.972013589179679 0.9571417663954044 0.9875859292242792 0.9570757153092907 0.9717796012337518 0.9569250978053436 0.9877231302568766 0.9570629427207638 0.9720227218543046 0.9571446214662196 0.9999826239466981 0.9574347132229423 0.9999295675886248 0.957083541218638 0.9888046571608304 0.9570359842143029 0.98867222010582 0.9584041945630196 0.9877851823154058 0.9892863078651687 0.9999303549019608 0.9892863078651687 0.9921649580888517 0.9896009967567568 0.9999846500386697 0.987645871599565 0.9926205044136193 0.9570054214285715 0.9888431063472046 0.9706740089485458 0.9720710865724382 0.9692432020997376 0.9998099472759227 0.9692320941788926 0.9698726851634141 0.969681381486676 0.9887722003538258 0.9715987364071336 0.9881080216919741 0.9919707796539504 0.9881303577623591 0.977664877185218 0.95727495127169 0.9999788983543079 0.9581291922130231 0.8829615065243179 0.9765801039313149 0.9582326134768367 0.9721587330517892 0.9891578867521368 0.9892065459670426 0.9923840486772487 0.9905643043844857 0.9765146584418553 0.9885497444561775 0.958506042047532 0.9884655827307529 0.9884010583475298 0.9703645900537634 0.9884509158679445 0.9884559655099 0.9880029334500876 0.9884601954951127 0.988469725881853 0.9884596026349343 0.8776486627597229 0.9877805759724909 0.9584179116098008 0.9584121420389462 0.9878398171256456 0.9878911656310264 0.9878534960223607 0.9586073634096576 0.9884280399556049 0.9880690718835305 0.9880797295454546 0.8835892965686274 0.9586926356413167 0.9583864470108695 0.9878502420218461 0.8840942964946448 0.8837946184243023 0.8765522080624187 0.8845214314214463 0.9881557674919268 0.9881255514785235 0.9892053464921691 0.9891451980198019 0.9690464749227478 0.9493551134122288 0.9878543359040274 0.9877213440860214 0.9879402727663231 0.9879745368579411 0.9874648861194674 0.987762526406553 0.9752616071850626 0.9751178249587167 0.9751939641340255 0.9748743494687132 0.9750448690110928 0.9748274827995255 0.9750750236406619 0.9880409174714662 0.987886927140656

GCA_002333565 0.8702681894515876 0.9571350410033769 0.9979699169505962 0.9994811848648649 0.9989700043449925 0.9580696267942586 0.876995345484222 0.9585432150615495 0.9586339685271701 0.9578897801660968 0.870888149001536 0.9587727900881582 0.8706358788811907 0.9995290487489216 0.9579591328047572 0.8770102603036876 0.9570605551497444 0.9989291730279899 0.9571588729016787 0.9990311056247317 0.9580013956340446 0.9996085946961788 0.958155290118961 0.9995819704641352 0.9582773943833943 0.9994605340206185 0.9583404716520039 1.0 0.9586996626794259 0.9994276257503621 0.9584437873918418 0.9992135522201377 0.9581986686103013 0.9593778173076923 0.9569746056475171 0.9990117862433862 0.9581428708016669 0.9996834726554377 0.9581946026328622 0.9992868262852406 0.9582099534427837 0.9992164589352044 0.9576168921254012 0.99930179991715 0.9582272058465285 0.9991504404737169 0.9581105311443747 0.9991830797850351 0.9584555936413314 0.9991747996678432 0.9575807546717776 0.999605489175687 0.9580683933382318 0.9996103588566659 0.9582383733916 0.9994954668611922 0.9582485551142006 0.9991341176470587 0.9579328037383178 0.9995524277335568 0.8757106719787515 0.999504358277592 0.9578193528693529 0.9991922484961627 0.9580159843482514 0.9993137702534274 0.9585348178137654 0.9991578143336127 0.9582826971260133 0.9991058162411051 0.9578540662283906 0.9993688374506954 0.9580410351610523 0.9993200291363163 0.9584053288987435 0.9996597863426897 0.9582143719571568 0.9993096988448844 0.9578900956351153 0.9992779444674679 0.957993493946133 0.9990687807076853 0.9584165854258987 0.9579474012921754 0.958360107632094 0.9579474012921754 0.9581873519334313 0.9579013268998793 0.9581284869059166 0.9578674932746394 0.9571914437213787 0.9993064662120275 0.9578968596059113 0.9570239369326327 0.9581800220318237 0.9571337460083518 0.9583433114035087 0.9573345163654129 0.9576231311394893 0.9578015833733781 0.9581998900024445 0.9577161850849126 0.9580957013685241 0.957985917217732 0.9573870255915017 0.958352624691358 0.9986320999367488 0.9583375268817205 0.9904923503582158 0.8726777535847053 0.9587805075513742 0.998568311804009 0.9582037586547972 0.957578752153581 0.9582802128186093 0.95715621634732 0.9569454862782861 0.9588965046123161 0.958500619383046 0.9899972671827351 0.9578608899127062 0.9578275854545454 0.9578944768147608 0.9578268969696968 0.9578191272727271 0.9573629922635389 0.9578503290587951 0.9578292864054184 0.9579301936108423 0.8771146132672722 0.9577311799122379 0.9995951619170985 0.9995877753779698 0.9577573750913965 0.9577707242136065 0.9577874890404288 0.9994190564411892 0.958310684560453 0.9576158568969757 0.9576379842674533 0.876635387463634 0.9995881828003458 0.9996250410899654 0.9577820702267741 0.8800171164699847 0.8763287235183695 0.8771189010402776 0.8773461561398753 0.9590015086621752 0.9589181579581026 0.9580178661800487 0.9579396408905915 0.957516937515436 0.944183148850139 0.9579112308440769 0.9579470112140419 0.9579763399170935 0.9578755490483164 0.9579966780487805 0.9580043458058205 0.9576157536945813 0.9579134105470675 0.9582898960653303 0.9583246302250804 0.9575874427762737 0.9583802757763975 0.9583272284088098 0.9582292448173741 0.958229624784004

GCA_015683755 0.8785812229862475 0.9987365239448052 0.9598564597961494 0.9602183514532949 0.9580634313022701 0.9890444944812362 0.8854478969849247 0.9782398085387324 0.9880054757107678 0.9889978912720324 0.8813329317548746 0.9992833273524722 0.8813346193247964 0.9602985347903658 0.9699713323747904 0.8854862338648444 0.9987379409351929 0.9581067592814373 0.9989543183183185 0.9580553038674033 0.976161681774809 0.9583794379614194 0.9921044037460979 0.9584457998556651 0.9988635401157981 0.9581116746297181 0.9922062961418143 0.958229784172662 1.0 0.9582018501783591 0.9906763170466885 0.957915924501082 0.9922495830727539 0.9881771061020037 0.9727797683142102 0.958531808816483 0.9727841236428098 0.9583896028537455 0.9921982553994547 0.9579924549335865 0.9727675165855817 0.9581073401473735 0.9704117815683486 0.9579625456702253 0.9921050513304002 0.9579096672212978 0.9901905213675214 0.9581358910302166 0.9780127604750167 0.9579616196348115 0.9882885058675608 0.9584489666666667 0.9727908106914072 0.9586137223148369 0.9921166034985425 0.9580898387096776 0.9891053972783144 0.9579756336350643 0.9885996344872071 0.9580259847764034 0.8791052394586153 0.9580720744428639 0.9781272026578074 0.9578628405591093 0.9727640520282187 0.9581174887146591 0.9880774230493916 0.9580627761972498 0.972866052983081 0.9580444952606635 0.9880353456221198 0.9581023275657738 0.9728764306326304 0.9581252529090477 0.9921211281835404 0.9584160351794628 0.9920956485355649 0.9580249454717877 0.9886862296834902 0.9580046926180869 0.9884564557235421 0.9595579039301311 0.9882296525364838 0.9884965097259063 0.9921669471912477 0.9884965097259063 0.99889532800328 0.9890675377418668 0.9922067923349301 0.9880821297107801 0.998935940032415 0.9578872888573455 0.9887021970021412 0.9716967547169812 0.9727320925597873 0.9703657488507138 0.9920337951301429 0.9697622734839477 0.9703055495283017 0.9707347935833924 0.9889848395781917 0.9727898196216453 0.9885629637760701 0.997919689985723 0.9886210845225027 0.9780936775200714 0.958048582230624 0.9922557676261994 0.9598285012395763 0.88496158059092 0.9772100045475216 0.9593888796296297 0.9729238294538946 0.9897783416108831 0.9891006743528388 0.9987386474319624 0.9894970302648173 0.9771832530949105 0.9893629039161139 0.9595410383275262 0.9884690187621309 0.9884574983797798 0.9716485219141323 0.9884510820734341 0.9884703321829164 0.9877262153163153 0.9884642262931034 0.9884712006898038 0.9884809913793103 0.8786062644450416 0.9874581599650352 0.95941522737608 0.9594249863698318 0.987596398249453 0.9876376819172112 0.9875920669291338 0.9595654109434812 0.9885329099099098 0.9886052006495013 0.9886232074159907 0.8847820588235294 0.9596420486815417 0.9592543464566928 0.987643326072751 0.8866894471387003 0.8846947446169254 0.8783058967606004 0.8847331262781188 0.9885082492905478 0.9884278210457229 0.9901817430191971 0.9884409838602697 0.9698812475915222 0.9500734624876604 0.9877210666086198 0.987548035010941 0.9878114419720768 0.9878430907103827 0.9875426644808742 0.9875635365051525 0.9757359389895137 0.9756991315726862 0.9757031269349845 0.9758053978084803 0.9756625291736128 0.9753084971236817 0.9756658536003815 0.9884934110592939 0.9883666287878788

GCA_002333585 0.8700513523410933 0.956840486981678 0.9976787430287429 0.9991577022375215 0.9989758894760017 0.9583847189695551 0.8766808500814774 0.9584935309400094 0.9588031915404952 0.957618172304697 0.8704919959058341 0.9586187312544633 0.870495248845562 0.9992731957876639 0.9575771339796171 0.8765591873830527 0.9569152617482347 0.9990929108869273 0.9569480499519694 0.9992233539577555 0.9570089417215314 0.9996492318810656 0.9577330004835592 0.999547054651648 0.9579350537371765 0.9994255064456722 0.9579154837141469 0.999119976919849 0.9585542047357093 1.0 0.958101911692156 0.9991316566576931 0.9577763407550824 0.9590951384541296 0.9563716998542983 0.9992179427850232 0.957997111274871 0.9996586372950819 0.9579193695228823 0.9993870930943165 0.9580149815860545 0.9993993284641915 0.9571279432098766 0.9994271387871387 0.9577684270479884 0.9990706102805652 0.9575982844080848 0.9993162426455672 0.9575217831563348 0.9993539592252805 0.9573503769983297 0.999554974263949 0.9579207095506997 0.9995900536857321 0.9578100434887654 0.999210818256579 0.9580012945544555 0.9992994362139918 0.9575480073619633 0.9990965636024071 0.875530722251726 0.9992375976845153 0.9570128921806702 0.9991962012195122 0.9577485528893241 0.9993756703567037 0.9580315258097166 0.9990160218151884 0.9579776477832512 0.9989167310855264 0.9573914181641574 0.999378229359687 0.9579967528948017 0.9993503555923092 0.9581272903384012 0.9996784851607586 0.957830631808279 0.9995029477084596 0.9574472224938875 0.9994792144892145 0.9574699384388081 0.9988273271577045 0.9579149152542374 0.9578871152007649 0.9579627385892117 0.9578871152007649 0.9579158180484225 0.9578261668273866 0.9577295678416224 0.9572886572610071 0.9569818639980711 0.9993630287682335 0.9577454447174448 0.9554469314678683 0.9579519357212956 0.9569410999015748 0.9580465558252428 0.9567000929549903 0.9570926583210604 0.9575304953113728 0.9577784368151936 0.9573814507007622 0.9575417971789882 0.9576757345392325 0.9565894933973589 0.9576366446240511 0.998894271620487 0.9580544447150513 0.9902278403863846 0.871944395778364 0.9579105447374908 0.9982022728277692 0.9580687955390335 0.9571559558823529 0.9582188806155374 0.9568916820388349 0.9565551237088638 0.9578967769211638 0.9581304847309744 0.9898071558775415 0.9575250421788383 0.957421816429776 0.9570286286407768 0.9574238785834739 0.9574247374759152 0.956907511155181 0.9575075643183456 0.957505152680933 0.9575150865800867 0.8765170712328767 0.9576254198101729 0.9993633125000001 0.9993676551724139 0.9575761323279008 0.9575868822957199 0.9576053719008265 0.9992045681671333 0.9577960343137255 0.9576996825006154 0.957716360059026 0.8762806743940992 0.9993670159551532 0.999365369011653 0.9575811484184914 0.8798510763358779 0.8759727807486631 0.8761681288261912 0.8772062316076294 0.9593108197495867 0.959209891098485 0.9573929876423553 0.957663465583174 0.9570796561108361 0.9440643213649097 0.957752537639631 0.9574306775133561 0.9578022262773722 0.9577100048697347 0.9574424993927617 0.9574613844282237 0.9570568284869393 0.9574094681113026 0.957777479574152 0.9578138257856966 0.9570246481299212 0.9580611461959224 0.9577552711067095 0.9580021571041616 0.9579912463054187

GCA_015683815 0.8767730318883906 0.9906272467248909 0.9592869759289553 0.9599765520403485 0.9579583943039529 0.9897477952069718 0.8849106148785425 0.9791188906215191 0.9902717992003554 0.990426177889658 0.8814866651130272 0.9904160324419113 0.8815289104059728 0.9597756257175661 0.9714441737236874 0.884592876923077 0.9906127737387092 0.9579799754178957 0.9909001037165083 0.9579936494181729 0.9784013366220343 0.9578918006352309 0.9903714866609294 0.9580824326328801 0.9905124662309368 0.9580880478185851 0.9903296298687326 0.9580749370214868 0.9908907609165586 0.9579368437806073 1.0 0.9579856650246305 0.9904040624327233 0.9908018864003695 0.9739516864767886 0.9581270169825252 0.9737641086319586 0.9579259740894647 0.9903906658019888 0.9579551844612755 0.973859015066337 0.9579557817470028 0.9716073998068565 0.9578525384427629 0.9903321112788441 0.9577693633692459 0.9982894830473 0.9579597645327447 0.9796910676556528 0.957986105263158 0.9903998944479628 0.9580378650036684 0.9738277545515847 0.9581099143416545 0.9904033814033576 0.9578900635230884 0.9900874935289043 0.9579454259759392 0.9904328844926612 0.9579079289215686 0.8795540934430583 0.9579688595203133 0.9790236306588974 0.957804967097246 0.9737980941704035 0.9579298288926913 0.9902647818871505 0.957857548828125 0.9734102065095398 0.9578747914125395 0.9900025768967875 0.9579658793356132 0.9739119846466472 0.9578860875948128 0.9904107665580892 0.9579209836465706 0.9903387157349449 0.9579539267399269 0.9904864338468032 0.9578863587487781 0.9904562198505871 0.9596654318975553 0.9900959491641858 0.9897137371897062 0.9904002889419944 0.9897137371897062 0.990507136304063 0.9900253546409807 0.9903302966466037 0.9906785947712418 0.9909645725473135 0.9578660350706284 0.9905000740270729 0.972956502620187 0.9737877715313695 0.9706057771896053 0.9902481459822395 0.9713385170826265 0.9714644380541577 0.97196838740458 0.9899913748062872 0.973801000895656 0.9908829230100044 0.9907951013734467 0.9909281152173912 0.9796914617791684 0.9579156943765282 0.9904036306830424 0.9591758198403647 0.8847744901866922 0.9792298196166854 0.9593310438512869 0.9740580858310627 0.9909170548977394 0.9903103499562554 0.9906258267543859 0.9901868652904874 0.9792301934015929 0.9912848647515202 0.9597275186567165 0.9897531793015333 0.9898264099829353 0.972156821599453 0.9897375149317407 0.9897550788240307 0.9888750468920394 0.9896110610248777 0.9896206804167552 0.9896144822453754 0.8776913759479956 0.9890379827400216 0.9589602733644861 0.9589948527349229 0.9891456093344857 0.9891801376640139 0.989149574514039 0.9593669027777779 0.9900014540588761 0.9901086848691695 0.9901120532060028 0.8837896192637418 0.9593612809821636 0.9593323945294391 0.9891395692440234 0.8873666340508806 0.8832643644174881 0.8764800877192983 0.883676304737516 0.9908937145877379 0.9907848335806656 0.9983542959527825 0.9890367122351335 0.9711668482207699 0.9506165753080211 0.9892310150537635 0.9889318346643643 0.9893213602069412 0.9893303854435831 0.989105356525497 0.9890108656135036 0.9775420953757226 0.9775105817307693 0.9775098509973567 0.9774469317635753 0.9774874452730333 0.9772649588776005 0.9774162144578312 0.9894970921052633 0.9893767812293457

GCA_002333605 0.8699819672131146 0.9562151065357912 0.9970951584470095 0.9986433019674936 0.998379167383821 0.9579164723782772 0.8763757914640733 0.9577436348684211 0.9582546433700334 0.9575101432386502 0.8702844328585962 0.9582699905370238 0.8702084132653061 0.9987278876548484 0.9577367062608265 0.8759056794701988 0.9562265612170973 0.9990785104209237 0.9564460362508943 0.9989540644361833 0.9568357781014809 0.9990580364828858 0.957687019532192 0.9990441086552366 0.9577814337788578 0.99902577162352 0.9579051153192522 0.9986375118434603 0.9580716579572449 0.998797692929293 0.9582752341054706 1.0 0.9577786296743064 0.9588142316898037 0.9560149239681391 0.9986981298106927 0.9580764275220373 0.9990806265306124 0.9578024478914202 0.9992311700437029 0.9579978489366905 0.9990699172886827 0.9573810441962117 0.9992703297576481 0.9578500072674418 0.9989645111731845 0.9571374005749881 0.9989017790791599 0.9571281702232034 0.9991094886025768 0.9570214319639041 0.9990565137238836 0.9578348926829269 0.9991255767260807 0.9577274783027966 0.9988419693564862 0.9580370919881306 0.9989761257810925 0.9575731150745175 0.9987143878812862 0.874635944591029 0.9986835465953509 0.9565893712212818 0.9992823765125968 0.9578306979014154 0.9992224750499001 0.9576868107564716 0.9985795555100369 0.9575811632453568 0.9985941418905065 0.9571177888108501 0.9990665197786431 0.9579914503067485 0.9990237371663245 0.9580086378087553 0.9991293301828642 0.9577832350096713 0.9990403659762719 0.957481995608685 0.9993376388888889 0.9574937401768173 0.9983365271872903 0.9576750367647059 0.9575387324946594 0.9578648322800194 0.9575387324946594 0.9576170131450829 0.9574730455635492 0.9576942857142856 0.9572387491030854 0.9564143017964072 0.9992919023136247 0.9576017838765009 0.9554689232585597 0.9579551512195122 0.9567372222222222 0.9579564221738079 0.9555132380029083 0.957407502415459 0.9571139516515079 0.9571838405621516 0.9570387212713937 0.9574936457334613 0.9575182318558209 0.9567656353722143 0.9571555539287457 0.9980190576565408 0.9580786047075952 0.9893756095890411 0.8737660805194806 0.957672427660618 0.9968134512302286 0.9581107305596831 0.9566493061324213 0.9579662114102248 0.9562783449771141 0.9563717459937814 0.9577497972304648 0.9581469588377725 0.9888991566265061 0.9573799375600386 0.9573811196540125 0.9573474117931369 0.9573892195004804 0.9573709029779058 0.9567196419753086 0.9573349101365923 0.9572922616195496 0.9573449065196549 0.876404527580557 0.957560907546712 0.9988697600171417 0.9989553941730935 0.9575579097963144 0.9575542493330099 0.9575758191953466 0.999026715906658 0.957660753240401 0.957150362834028 0.9571628676470587 0.8759802783613446 0.998946510182208 0.9989093884120172 0.9575675466666667 0.8798840222615735 0.8760398193411265 0.8759059066947812 0.8768774320323016 0.9592154998834771 0.9591497661365763 0.9571061659409773 0.9575694895536562 0.9568926274316671 0.9438430608651912 0.9578280392632089 0.957626137079196 0.9579083911671924 0.9578043561710398 0.9576031601941748 0.9576389679456047 0.957396331448242 0.9575932383992144 0.9579827239082163 0.9580317065351419 0.9573520230788117 0.9581864301662119 0.9580056931425752 0.9579227949852508 0.9579137447750184

GCA_015683835 0.8772450704922088 0.9919206300211417 0.958774072117593 0.9592654535121617 0.957290073046019 0.988826357615894 0.8848600329781837 0.9771036200399025 0.9878228504043126 0.9894016457846676 0.8805602530459231 0.9925748303819074 0.8804555675675677 0.9592460369609856 0.9700298086703802 0.8847197154471546 0.9919461165670367 0.9567782311606496 0.9920399100982646 0.956833434761441 0.9746990924131411 0.9570673967640668 0.9999668729260199 0.9570855813385442 0.9923318409863946 0.9568890360862194 0.99994985669415 0.9568989922103213 0.9925151992449665 0.9569329954139513 0.9902470784568703 0.9567436065175098 1.0 0.9886396696906258 0.9718532759001547 0.9571545414740937 0.9724119043361645 0.9570709466312485 0.9999485185913831 0.9568283938446741 0.9724117909781153 0.9568208552156107 0.9699322020105313 0.9567036503362152 0.9999586688884533 0.9567097031136856 0.9903548789890769 0.9566914527435338 0.9776287897803676 0.9567020173326914 0.9887665881104033 0.9570926950012075 0.9723618011591617 0.9571986776459193 0.9999622230904475 0.956796802311582 0.9881994541867603 0.9567931819280018 0.9890266688075359 0.9567693725868727 0.8799129234828498 0.9567539946962392 0.9768840214237894 0.9567542778178323 0.9723568521816564 0.9568019518652227 0.9873056217494091 0.9567801994712811 0.9720572991720744 0.9566852944003845 0.9875958696649839 0.9567968116290244 0.9723129318435754 0.9567343555769694 0.9999112983919 0.9571114130696889 0.9998841650333203 0.9567321378813357 0.9890899361837907 0.9567115427196151 0.9890006845493562 0.9583267783094099 0.9878104374856089 0.9891028773048032 0.9999135608308606 0.9891028773048032 0.992196583403895 0.9893869940931964 0.9999433709834469 0.9875743336992318 0.9921721718024484 0.9567249724418884 0.9891400874014069 0.9710949184782609 0.9723895334821429 0.9695095920830317 0.9997904615989001 0.969342275878556 0.9699206912114015 0.9699224312144213 0.9887258927374302 0.9718912001761727 0.9877884460494637 0.9921700993027679 0.9878485342375848 0.977789687080912 0.9568947945532728 0.9999925206286837 0.9585688886363636 0.8831909508831359 0.9767495843799954 0.9581697418285174 0.9724258383974792 0.9888955644290957 0.9888741393712904 0.9919434651360544 0.9903563651877134 0.9767123718685361 0.9883289504061565 0.9585831166436147 0.9884359978517725 0.9883823784946236 0.970690320018157 0.988372447311828 0.9884646279569893 0.987951196637912 0.9884485290959845 0.9884564626288661 0.9884639458995277 0.8777839287641662 0.9879759052194803 0.958164832679437 0.9581549907663895 0.9880395561871449 0.9880932927094669 0.9880472012235089 0.9583851301964367 0.988102825745683 0.9879398339483395 0.9879823013130614 0.8838970254110615 0.9584620411899314 0.9581317848187827 0.988075579016108 0.8839960410484669 0.8840785775213247 0.8770158561643837 0.8842107768469154 0.987942285028372 0.9878613738788012 0.9894246028645833 0.9890544606613453 0.9696518212560384 0.949409628243513 0.98811304716366 0.9879008575169103 0.9881924912891985 0.9882308662448178 0.9877927702702702 0.9879226551573428 0.9753558341300191 0.9748264362081254 0.9753341288782816 0.9749856924546323 0.9752240759312321 0.9750205615550754 0.9752815198659646 0.988140082515611 0.9879893072625697

GCA_002333625 0.8688030777338606 0.9892512657342657 0.9580257364897179 0.9578879567078972 0.958120672753484 0.9897593482734965 0.87649822427369 0.9766768561792338 0.990124971936389 0.989287744138402 0.8683603268983268 0.9892745939201453 0.8683226310353732 0.957907950059453 0.9701820861568249 0.8765894294294294 0.9893002091656874 0.9579019789423308 0.9892955678926673 0.9579209319169484 0.9766038964181993 0.9580724737344793 0.9890573999537359 0.9582597519267821 0.9894155240529863 0.9581232390991855 0.9890223999074289 0.9580614089925463 0.9886353811354899 0.9579294256434698 0.9908034465234467 0.9577928395657418 0.9890444050925925 1.0 0.9702571115921116 0.9584101367234348 0.9702600364697301 0.9581336753154013 0.9891217870456663 0.957993419861872 0.9702742027224113 0.9579664006669844 0.9703091511285574 0.9579733539928488 0.9890644416066868 0.9578606759016002 0.9907333782857143 0.9579201003104849 0.9768785316393844 0.9579335981865904 0.9884011091234348 0.9581641710338255 0.970276931101407 0.9581887923627684 0.989049326388889 0.9579970245179721 0.9888302117263844 0.9579999162879694 0.9891716077981652 0.95799416407558 0.8753676393799293 0.9579936341986188 0.9766198379293661 0.9579333071802186 0.9702036658595641 0.958006641221374 0.9899728986197048 0.958010782774209 0.9702671331554694 0.9578884268060837 0.9898264536225196 0.9579414509990486 0.9702606911657339 0.9579603524648725 0.9890859105728924 0.9581884527493455 0.9890678123548537 0.9579332777910022 0.9893033264746228 0.9579968335717692 0.9891931311966797 0.956736188143067 0.9900028960739029 0.9892414185994146 0.9890421306884482 0.9892414185994146 0.9894439860302678 0.989025829555757 0.989042792417938 0.9899666337088985 0.9893607232558139 0.9578303540874525 0.9892825482980682 0.9702582995283018 0.9702728290432249 0.9699541317073171 0.9890883116581515 0.970264742638257 0.9702887493905411 0.9705162929997595 0.9909074532008321 0.9701773823246879 0.9898415943374334 0.9891047090069285 0.9898290118302019 0.9768836061194754 0.9578296826533047 0.9890801528839472 0.9565669461797214 0.8722433731105806 0.9768499344978167 0.9572519741021256 0.9704928268899287 0.9894340766389658 0.9889492711027511 0.9893089295774649 0.9895510505836577 0.9769072913616397 0.9899994938158497 0.9567575339087547 0.9889961820250285 0.9889996747042767 0.9698583776722091 0.9889975199089875 0.9889880686988172 0.9881125023408242 0.9889842510213346 0.9889958329550613 0.9889948898978435 0.8766366127262755 0.9890064027303755 0.9578913622291022 0.9578980437886722 0.9889514480563765 0.9889524243802593 0.9889569697658559 0.9578796144693003 0.9901406165971257 0.9899797743332573 0.9899770364464692 0.875575013477089 0.9578883432516068 0.957694126606378 0.988968622414185 0.8797146012903225 0.8756304947566551 0.8749207723577236 0.8770534653194976 0.9901657398897059 0.9901178217821781 0.9897389658377876 0.9890792259367288 0.9701923279789019 0.9498591817026683 0.989116616609784 0.9891989116575594 0.9891912411751309 0.9891353608012747 0.9889803117178612 0.9892186541970803 0.9769711119150989 0.976908583815029 0.9768948590701035 0.976901636933462 0.9769400987713803 0.976900550857695 0.9769232466281309 0.9891573057354304 0.9891523340272959

GCA_015683855 0.8780963229904716 0.9720849362437863 0.9580553592542066 0.9593750145838008 0.9571610887974836 0.971099087460955 0.8867043385455448 0.973111853679468 0.9720422879235843 0.9734860697374086 0.8809164817383266 0.9724575083873853 0.8808173469387756 0.959118396120009 0.9901247582519758 0.8857118631368631 0.9720379642545772 0.9569338463396956 0.9725887320625403 0.9570403530557584 0.9714456476564304 0.9565334097968937 0.9718704902386116 0.9566928981660232 0.973581877352225 0.9561390009560229 0.9719453198872262 0.956256953594614 0.9726848534838289 0.9562266046733429 0.9744503290351267 0.9558658892617449 0.9719328751357221 0.9701576735280039 1.0 0.957318652585579 0.9902724782146654 0.9566113589682351 0.9719872225855679 0.9561379562217464 0.9903158397108844 0.956252717313646 0.9888781201638598 0.9561652690476191 0.9719064511212715 0.9562253328561202 0.9726026063368056 0.9562444121154304 0.9737181294964029 0.9561135976190477 0.9729267505376344 0.9566121079016473 0.990316515923567 0.9566624635951301 0.9719303860333984 0.955906489311164 0.9734590941385436 0.9562730183727034 0.9732147694306127 0.9560604254813406 0.879418496183206 0.955990512942294 0.9726266889704296 0.9561430194959581 0.9902851664899258 0.9561159442060085 0.971103885948027 0.9568476007677543 0.9912056640542258 0.9567824310716855 0.9701754188906939 0.956260563346803 0.9902790651618399 0.9562710999999999 0.971927730155259 0.9566082967032967 0.9719734951033732 0.9561520869978607 0.9731952820844099 0.956170057129255 0.9731515089402529 0.9579559855170854 0.9711110679840037 0.9697223815082886 0.9719606754098359 0.9697223815082886 0.9730064568146677 0.971051450927789 0.9718747604595707 0.9717683144318434 0.9726928916181858 0.95613367671493 0.9730998749730546 0.990195428323574 0.9903001361412467 0.9894188331008832 0.971799882250327 0.9895716951501154 0.9889942786069652 0.9889309986504724 0.971272534698521 0.9997836828793775 0.9731411259754738 0.9721038852242744 0.9725511167400882 0.9737487210058531 0.9563693831012964 0.9719961992619927 0.9588261485434734 0.8850681908831909 0.9728294991055455 0.9582134502923977 0.9902481872852233 0.972676968832309 0.9735134647950089 0.9721322971503155 0.9717779355939433 0.9728759711256486 0.9734419576490925 0.9587618988203266 0.972717953293677 0.9726166357820429 0.9897492422926618 0.9726255403617117 0.9727790407938259 0.9722871761248023 0.9727498526500991 0.9727529909830659 0.9727490629124504 0.8785929855923159 0.9711256151279201 0.9582067663981587 0.9581884826157036 0.971305301983508 0.9714432194364322 0.971361603920695 0.9584931698973774 0.971474269072636 0.9705744153992395 0.970558973871734 0.8857043672876844 0.9584824954379564 0.9582349270738376 0.9714150800355715 0.8862436402981486 0.8838703272186416 0.8779592122933885 0.884433193044355 0.9721075338212464 0.9721317807914629 0.9730241217850077 0.9693815052508751 0.9899429075397741 0.9477132725480533 0.9714935643125969 0.9714104609218437 0.9716683488681757 0.9715865950376606 0.9713791196087149 0.9714810459411242 0.9713455574082897 0.9712769131985731 0.9713473893384105 0.971375084543939 0.9712525958561561 0.9710826754491018 0.9712655955216771 0.9716418928651812 0.9713803408577877

GCA_002333645 0.8698693632075472 0.9576191636096846 0.998901401646447 0.9995632019917732 0.9991658530701755 0.9588036994082839 0.8760271424694708 0.9589963110264348 0.9589886888406846 0.9577465253411306 0.8706708260869566 0.9585001045130642 0.8704920343766035 0.9995441084936245 0.9577736525612472 0.8757859412873686 0.957723827586207 0.9994561264319051 0.9576391725309391 0.9992982452749142 0.9577524711373127 0.999594846703066 0.9578708565206873 0.9995346390006353 0.9579571456405261 0.9992739052013424 0.9580844942724837 0.9991226525821597 0.9585450155020272 0.9993481844680409 0.9579209569495695 0.9990084961767205 0.9579232687651331 0.9591507742090124 0.9575455730447615 1.0 0.9580109698750917 0.9996183465387825 0.9579880063214199 0.9992959400544958 0.9580776206812057 0.9991580871014796 0.9571349384236454 0.9992933144297418 0.9579531644034919 0.9988729764408919 0.9582736418853256 0.9991519378389654 0.9580671672439387 0.9993102017654476 0.9575444661582461 0.9995532340604029 0.9579515855150479 0.9995788872559311 0.9579084027105518 0.9995255471539594 0.9577239851485149 0.9991867117783888 0.9578120499632623 0.9994035159720754 0.8747779865410498 0.9995364380671028 0.9578026725620241 0.9991200858998533 0.9578778068459659 0.9991867024636766 0.9585324268415742 0.9993116818747407 0.9579029015670912 0.9993215333609615 0.958035910316099 0.9992189458086368 0.9579887892927309 0.9991575297113753 0.9582434240196078 0.9995922018927444 0.9579602746051035 0.9993180461442528 0.9576306330970423 0.9992956552736424 0.9577898107177976 0.9991859855170853 0.9580433071638863 0.9577035383881736 0.958144124390244 0.9577035383881736 0.9580189314466943 0.9576551673489044 0.9578765166908564 0.9575152898197761 0.9577017624052799 0.9991873387601753 0.9579452187807276 0.9561365411040038 0.9580322828480549 0.9568037843137256 0.958146140776699 0.9569703512195123 0.9571962205304521 0.9576724520048602 0.9582888539104772 0.9578046566576421 0.9575735026737969 0.9577538233855185 0.957652351797862 0.95806129405965 0.9994525777310923 0.958156742996346 0.9904653897756188 0.8717832716212642 0.9583750961538462 0.9987733474576272 0.9581817974746224 0.9573720838471023 0.9580770296296297 0.9577275835791543 0.9573874713763703 0.9583557068322982 0.9580526845800048 0.990161544065805 0.9576574698213424 0.9576567125271542 0.9572310699588478 0.957643568324481 0.9576598407720145 0.957291574235265 0.9576581454720616 0.9576120105947508 0.9577069067694531 0.875807589997252 0.9575580233122876 0.9996242878820725 0.9996138574832142 0.9575608574204517 0.9575906022340942 0.9575941082261588 0.9993242504332756 0.9578286750245821 0.9572813567222768 0.9573068366846493 0.8755414679144384 0.9996164903533493 0.99961625 0.9575917181044958 0.8790058451980326 0.8752208025682182 0.8761000133049496 0.8767077654687923 0.9590687352524775 0.9589328720626632 0.9580406352710327 0.9575850632609216 0.9570736623889439 0.9443642384105961 0.957745932038835 0.9576340160155303 0.9577954266958423 0.9577469133398248 0.9576745158942005 0.957649846752615 0.9572476602013258 0.9576218239921337 0.9579103557312252 0.957928612688225 0.9572599337260678 0.9580497103243377 0.9578753428712382 0.9578760992385164 0.9578643376751045

GCA_015683875 0.8769284871296745 0.9730150773309766 0.9591137085635358 0.9595201194500789 0.9576727089895809 0.9716202695294655 0.8855412295700277 0.9742354371881102 0.9716613865740742 0.9731156306207501 0.8806644429129336 0.9728276228956229 0.880686935632184 0.959179184733514 0.9914740603875619 0.8849722281234194 0.973057260762882 0.9573569677888107 0.9733108670899934 0.9572342175711852 0.9717697877798507 0.957633726392252 0.9726840840707965 0.9576162003412138 0.9732776324689967 0.957311702643706 0.9727043492695883 0.9573336168657081 0.9729398261826182 0.9573749250845818 0.9740015497076024 0.9574162521294719 0.9727857256813649 0.97049359192607 0.9907457314200043 0.9575477464103189 1.0 0.957455660468486 0.9727669632925473 0.9573247582391148 0.9999636916248554 0.95747645098986 0.9981525738125804 0.9572536856042371 0.9727914358632934 0.9571425108538351 0.9738782300688429 0.9572973131801693 0.9741194261752373 0.9573428819277108 0.9726537356321839 0.9575424014510279 0.999883074845679 0.9576866167230547 0.9727799070796459 0.9570343264621285 0.9737891220508167 0.9575750072568942 0.9727824390784227 0.957094998797788 0.879255843045843 0.9569648644758936 0.97387813020605 0.9571672012503006 0.9999306877287613 0.9572828592092575 0.9710400509337862 0.9571286386554623 0.9912083630888363 0.9570211169702779 0.9712284939329051 0.9572605081888246 0.9998098332040342 0.9574141604252236 0.9728613874637521 0.9577093452524765 0.9727552989553234 0.9572297930204573 0.97282539650983 0.9572315824915825 0.9727182607724938 0.958910111872146 0.971357253218884 0.9704140095465394 0.9727137003126396 0.9704140095465394 0.9731685281579537 0.9717123684210526 0.9726871283185841 0.9720209270172256 0.9736054076685764 0.9572541052125869 0.9728334269911506 0.9912415941101153 0.9999918812644565 0.9906414670039091 0.9726046236080178 0.9910368360277138 0.9982577971507549 0.9905934834701576 0.9722188642723881 0.9906737650343643 0.9727370654883299 0.9727098548459133 0.972690790307971 0.9743498688811189 0.9568867162389062 0.972847949456883 0.9588272829006268 0.8845830645161291 0.9730341707976566 0.9587291451385628 0.99975118521416 0.9730962575723581 0.9736368505539227 0.9730900133570792 0.9730156712450154 0.9731013137032842 0.9742040026391027 0.959950752860412 0.9722317027449229 0.9722629243809949 0.9915073075268818 0.9722506064659977 0.9721747690247712 0.9717086138613862 0.9721376125724477 0.9720941622103386 0.9721644905239687 0.8785511807228915 0.9709573191011235 0.9584552847955903 0.9584224396967609 0.9710525331833522 0.9711733788931213 0.971071093855503 0.9587663313609467 0.9721154905616406 0.9703543541015157 0.9703927036770007 0.8854609500247402 0.9587753882942381 0.958465147794452 0.9710749024882313 0.8853925176699977 0.8841877043868394 0.8785230970442062 0.8845200733249052 0.9727919349775784 0.9726566988981336 0.9741316372176247 0.9700553760114231 0.990464430350012 0.9483898701298701 0.9715528139013454 0.9713655015744489 0.9715978625954198 0.9715663167380041 0.971211347103727 0.9713607948660211 0.971323824852071 0.9714033980123048 0.971462060146815 0.971529607007576 0.9714707748815166 0.9711511113755356 0.9714580203646698 0.9717657284691756 0.9716618215441516

GCA_002333665 0.8700609754811496 0.957605695863747 0.9980502274696708 0.9997430002177226 0.9991464206313917 0.9585735153664302 0.8767651464549684 0.959232005730659 0.9593252730375429 0.9581234587020651 0.8701781518921721 0.9592163782358581 0.8700603039750585 0.9997383933999132 0.9581627111444277 0.8763266847090663 0.9576128995826173 0.9995663759982734 0.9576918018890773 0.9994805090670745 0.957821992090954 0.9997419737390579 0.958279619140625 0.9997302869198313 0.9584326660108216 0.9997790764063811 0.9585741849028767 0.999507312954254 0.9591628688524592 0.9996576033402923 0.9584213479234021 0.9994918085332202 0.9584002836185819 0.9594848481166465 0.9571850343305542 0.9995927749360616 0.9586683523573202 1.0 0.9584968225449175 0.9996715564853557 0.9587481406288686 0.9996933458177278 0.9579603961136024 0.9996264655532359 0.9584313444553485 0.9993143713204374 0.9581376232095168 0.9997082665832291 0.9581933450351054 0.9996714264798159 0.9577382754462131 0.9996649325306207 0.9584922263404991 0.9996373609664653 0.958388096285435 0.9996814919774952 0.9580465386533666 0.9995168538851352 0.9581311659637806 0.9996078046727006 0.8759093805309734 0.999630258186398 0.9576527371007372 0.999611220735786 0.9585324950641657 0.9995932702418506 0.9584940490171049 0.9997167911857292 0.9584206574051103 0.9996713693467336 0.9577158255984368 0.9997022191722974 0.9586598907103826 0.9996250487908358 0.9587755856079404 0.9997840162907268 0.958384952264382 0.9997740692371475 0.9579746085453198 0.999796989763944 0.9580442665340627 0.9993244849589791 0.9581745087849542 0.9582947590361447 0.9585211800936191 0.9582947590361447 0.9584883839131507 0.9582486371379898 0.9583538271906272 0.9576257027027026 0.9577646729880865 0.9996751456512692 0.9581916090524746 0.9559402649164679 0.9586673391089109 0.9573556856435644 0.9585718834190547 0.9574977019704435 0.9579225402327309 0.9580861700581396 0.9580272919743967 0.9581313941949888 0.9580097791952895 0.9586021777777778 0.9573486134045653 0.9581535540674604 0.9997247846481877 0.9587347468043266 0.990133258400927 0.8722717952127659 0.9586030414171657 0.9987317759317468 0.9586965407703852 0.957272326156892 0.9585731226580064 0.9577540328915072 0.9571291850594229 0.9587207839677337 0.958455919863181 0.9898669853980216 0.9578816403017766 0.9578099294403893 0.9579053812316717 0.9578687731256086 0.9578931143552312 0.9573313934837093 0.9578579616132167 0.9578022778047596 0.9579183280680438 0.8764495164410058 0.9578243849742205 0.9998327826276736 0.9998330418665503 0.9578501963190185 0.9578761094747177 0.9578697105004907 0.9998232638279193 0.9579267580924142 0.9578534044135877 0.9578885866600545 0.8761961667109104 0.9998431841990397 0.9998290015293861 0.9578839464636542 0.8797477535301669 0.8757240516545602 0.8758479147558674 0.8770309001367989 0.9595237544696066 0.9593349258018191 0.9579401198044009 0.9579984248554914 0.9574397359900374 0.9443817618312756 0.9580742906234658 0.9580885668711657 0.9581908177799606 0.9580462109663144 0.9580936173870335 0.9581052064896755 0.9583356477329348 0.957931737298637 0.9583278361553786 0.9583725566343044 0.9583176992031873 0.9585880070017505 0.958318643603783 0.9580934910802775 0.958073002727498

GCA_015683885 0.8773026686361398 0.991710115159129 0.9594152980132451 0.9597558184691805 0.9577116807738816 0.9887908855196659 0.8850555062972294 0.977579445056155 0.9881515582383188 0.9892220054713805 0.8805378015338137 0.9925608387914642 0.8804600675360967 0.959772306122449 0.9705148146364951 0.8851626117706493 0.9916963690099221 0.9571239537683601 0.9919229098445597 0.957272299491156 0.9752454393796992 0.9575164136440069 0.9999413418406806 0.9575451530612245 0.9923640084121977 0.9572389708363461 0.9999688543085004 0.9573200363901019 0.9924185260535603 0.957268164785011 0.9899944695017183 0.95710458655706 0.9999735473887814 0.9887815049780041 0.9720172713314323 0.9575971217264793 0.9725394840566874 0.9575449411482105 1.0 0.9571511615678776 0.9725111492471215 0.957205721822542 0.9700900781990522 0.9571934878165314 0.9999439157445157 0.9571327362110313 0.9900980819881054 0.9571148906512859 0.9780697417056335 0.9570969913834371 0.9885053606073386 0.957574090799904 0.9725425553097345 0.9575980726660251 0.9999349254598258 0.9572235575772087 0.9883806040122111 0.9571704413528425 0.9889520993419657 0.9573021262299015 0.8801338254507447 0.9572500023975067 0.9774228806401424 0.9570835992357297 0.9725138551556636 0.9571447224880383 0.9876814238644387 0.957206624401914 0.9725265063460253 0.9571943690248567 0.9878056819739548 0.9572146019603156 0.9725540895985808 0.9572328722128987 0.9999494222833564 0.9575572282217423 0.9999344686224986 0.957127027479092 0.9888896176594846 0.9571295625149414 0.9888277993182786 0.9586383593573207 0.9879843220338983 0.9888795068988917 0.9998927151799687 0.9888795068988917 0.9922460234653258 0.9893708766586905 0.9999978239042286 0.9878394890829695 0.9919748643006263 0.9571333659117999 0.989024778293919 0.9714808147647985 0.972522802477328 0.9696585738295318 0.9998961533969243 0.9696390861618798 0.9699723457371644 0.9701621898496241 0.988880475661258 0.9720877753496504 0.9882983532738743 0.9921581143934631 0.9883623331158948 0.978054136858476 0.9574046449774402 0.999968823987539 0.9590148337480209 0.8833708607838423 0.977282534121929 0.9585718329411766 0.972563437639696 0.9890074130528124 0.9892844022687609 0.9917239705263158 0.9901428874284505 0.9773891607429489 0.9885980246389126 0.9592109758897819 0.9886907429547396 0.9886775422098738 0.9712795110410093 0.9886786175213675 0.9887383885445609 0.987984032967033 0.9886850437566703 0.9886886958377801 0.988692930004268 0.8780863846360463 0.9879109339111593 0.9588818688374227 0.9589099885373682 0.9880412472885033 0.9880929574422121 0.988048024289742 0.959024697864607 0.9885681420276909 0.9877488489702516 0.9877856061299176 0.8846114608695652 0.9591286406001365 0.9588544865109954 0.9880477031114953 0.8846003733726358 0.8848603325831458 0.877372248102591 0.8848547101999493 0.9881878634886241 0.9881258369565218 0.9896131698357823 0.9889219116647793 0.9700479006717849 0.9495555128840435 0.9882019261498597 0.98781544312026 0.9882700864864866 0.9882664047619047 0.9876943580861658 0.9878439370932754 0.9757681876484562 0.9751213682472281 0.9756957908465733 0.9753549323843417 0.975625933127816 0.9753773350797811 0.9756605559515323 0.9883249590073122 0.9882329837813819

GCA_002333705 0.8704194063567114 0.9569343985507247 0.9977909004739336 0.999003618903755 0.9990268378140151 0.9577557695027105 0.8768755633994028 0.9579693747028055 0.9583513947876448 0.9573461469009272 0.8708485442875481 0.9585804238095239 0.8706678764478764 0.9990872339508832 0.9578962018394234 0.8756199893532073 0.9569557389822255 0.9994873102447117 0.9570244412400866 0.9995673735040942 0.9571317749023439 0.9996079816704854 0.9576263530552862 0.9994244381313132 0.9580440702781844 0.9995655635838151 0.9578852701051088 0.9990213950042338 0.9584898351254482 0.9992883590693845 0.9582517716827279 0.9994702427484056 0.9576627202135404 0.9592794757187727 0.9565783681906614 0.9990892949670843 0.9580186075638507 0.9994465210188445 0.9577621493047085 1.0 0.9581106915154487 0.9995363744367063 0.9576469360351563 0.9997971534306863 0.9576432375152254 0.9995482145021208 0.9572885955191521 0.9995467575881871 0.9573602054455447 0.9997182780791641 0.9571137628496293 0.9995440137185617 0.9579418682664056 0.9995579529362765 0.9576873666343356 0.9992195305359368 0.957781159851301 0.999673642289672 0.957484942217851 0.9990882490597578 0.8755283382235263 0.9991308782936009 0.9567075820170111 0.999619622944244 0.9578908744504152 0.9997682700080841 0.958052890070922 0.9988864827155353 0.957705683771176 0.9987656231823846 0.9574620759308834 0.9996361678756476 0.9580503319400049 0.9996275088229188 0.9580095715341049 0.9994363287442637 0.9577357915245982 0.999506829318089 0.9573503898014221 0.9998072521530142 0.9574861430332922 0.9989156913328809 0.9579212610619467 0.9579006510292005 0.9578938516160628 0.9579006510292005 0.9580522890682319 0.9578292394978273 0.957638793019874 0.9575799566160521 0.9569704413892908 0.9997492783094099 0.9575848891625617 0.9554972111742425 0.9580527735618114 0.9569969151291513 0.9578889183922047 0.9557566788499026 0.9576348314334223 0.9574599422938205 0.9575086477910668 0.9575323567035671 0.9575525918808552 0.957870685770267 0.9569158541221193 0.9574683353793273 0.9987828347457628 0.9579484517704517 0.9898422634979234 0.8737194852364778 0.9581083687592684 0.9971148639079443 0.9580919632663194 0.9569119823356232 0.9582134119106699 0.9570003796544171 0.9567953309265944 0.9580677150671306 0.9581749344341914 0.9894408905554254 0.9572797339138849 0.9572535082205029 0.9573158894813378 0.9572775918762089 0.9572636750483561 0.9568523276076674 0.9573282520521487 0.9572972283920811 0.9573477686549143 0.8767761637458928 0.9575268519873201 0.9992589565780946 0.9992352708827973 0.9575115622715087 0.9575180550816477 0.9575187624847747 0.9993137556658752 0.9577542131107292 0.9576566518956179 0.9576921358267717 0.8768551319261214 0.9992531620979926 0.9992109723422645 0.9575856013661869 0.8797928469569645 0.8765555182520649 0.8764958882978722 0.8771458748645721 0.959191225593978 0.9590318590951934 0.9571372230320699 0.9576501914333574 0.9572356192830654 0.9445060591384146 0.9577218274853802 0.9573825097560975 0.9577693655441681 0.9577044884004885 0.9574700756466568 0.9574205889540568 0.9573877805178792 0.9574977923038973 0.9579036874690442 0.9579172864570439 0.9573434500616524 0.9580988830845771 0.9578671853320119 0.9579295180425111 0.9579283213844252

GCA_015683915 0.8768230218446601 0.9729487049723757 0.959285684915685 0.9598492961876832 0.9578084223360156 0.9716287905807042 0.8855325207130303 0.9740870078397212 0.9719561295565359 0.973144552238806 0.8804228768382352 0.9727188166741371 0.8805396498502649 0.9595410083653628 0.991597660488246 0.8849247193294386 0.9728791793041929 0.9576808767657089 0.9730697498903027 0.9577390117704758 0.971824167835437 0.9578437775623939 0.9724946653368793 0.9578699608035278 0.9731255050618672 0.9576653664475285 0.9725077831966306 0.9576858659491192 0.9727313757429012 0.9575670450145208 0.9739660153084196 0.9575236079960995 0.9725150300066682 0.9704506056920458 0.990788349806618 0.9578529846003422 0.999966373519705 0.9577299442559379 0.9725811850033474 0.9576247030420088 1.0 0.9577622257205135 0.9982266208082545 0.9575766932559826 0.9725784721603563 0.9574523473837211 0.9737525177777779 0.9575986265469546 0.9739097768448963 0.957555327532028 0.9727110629834255 0.9578728564500487 0.99983785327139 0.9578495880785074 0.9725387871396894 0.9572579307025987 0.9737832628467485 0.9577560325163795 0.9728215251607182 0.957481464414958 0.8791486721991701 0.9573682048192772 0.9736221152154794 0.9575918526570049 0.9999118304692026 0.9576285285575993 0.9711323080672679 0.9574503228137797 0.9912822909482759 0.9572594031287605 0.9714319207826294 0.9575603384094756 0.9998037310532452 0.9576281732397776 0.9726323240223463 0.957869915130941 0.9725303314794215 0.9575004589371979 0.9730077353071144 0.9575618691137406 0.9726837259722844 0.9591021227387222 0.9714253664359036 0.9705168994280267 0.9725971543444272 0.9705168994280267 0.9729712856181288 0.9718448844657972 0.9724526419698315 0.972089925153096 0.973345366122629 0.957532831644959 0.9730445563442769 0.9915651474989312 0.9999717689181342 0.99081632780083 0.9724292082961641 0.9913185677926886 0.9982872245943639 0.990869708110517 0.972185534796824 0.9906779599310642 0.9728058462238398 0.9724926608579089 0.9727971029311521 0.9742176155025182 0.9571425595381285 0.9725951031728423 0.9587736516476126 0.8846204796336467 0.9730395589558957 0.9586368101623148 0.9997762102785406 0.9728447973885637 0.9734401382279628 0.97294794517495 0.9728161300309599 0.972939834053194 0.9742198810834619 0.9598502803953115 0.9723914113443501 0.9724218683035715 0.9916609502262443 0.9724045748716804 0.9724317314566578 0.9717370391705069 0.9723872534582776 0.972374389917466 0.9724106873465744 0.8784049462365592 0.9710351542445395 0.958491399907749 0.9584889806273061 0.9711294997746732 0.9712121633752244 0.9710947386210004 0.9588822184144391 0.9722936764705884 0.9707787277108435 0.9708201131984586 0.8851152030708273 0.9588470409329979 0.9584714856230032 0.9711023713193977 0.8850197778320313 0.8838914260782846 0.8785845195077246 0.8847219059656218 0.972801569375842 0.9726926244649695 0.9739201341981659 0.9699977502972652 0.9904595416078985 0.9482991478942213 0.9714991084662026 0.9713514752252251 0.9715154060742407 0.9715473572556984 0.9712275843454791 0.9714308572073088 0.9715065141095566 0.9714355631803123 0.9715187677725118 0.9715652903531643 0.9715484775907044 0.9711187789172432 0.971441592689295 0.972120567867036 0.971953803567292

GCA_002333725 0.8705128190928271 0.9572904300291545 0.9979467178710515 0.999135905050943 0.9992099977885892 0.9586049988193627 0.8769151060358891 0.9584101281746975 0.9590387257751938 0.9579069703649278 0.8708795220966085 0.9588384740492706 0.8707955855855855 0.9993355279099957 0.9576939197607773 0.8769195163890382 0.9573104854130914 0.999495293121693 0.957425476766699 0.9995170786035553 0.9571289931406175 0.9996447253433209 0.9581571921301919 0.9996497352384955 0.9583061027784607 0.9996998515157764 0.958382196376102 0.99909521454238 0.9587787367915466 0.9995585969965027 0.9585638396624472 0.9995076021684738 0.9581795772594753 0.9591579636363637 0.9565808223523671 0.9992544168787109 0.9582287487636005 0.9997703118546056 0.9582537481662591 0.9995965689089418 0.95831114017769 1.0 0.9575375713222526 0.9997006572191416 0.9582566756097561 0.9994070650154799 0.9579066052822873 0.9997059062052017 0.9576443929633301 0.9995925195392842 0.9576705794662179 0.9996866971904267 0.9581424642328565 0.9995262276321266 0.9582176810889643 0.9991313329187397 0.9580475037332006 0.9995631013710012 0.9578428141941844 0.9992096711353164 0.8757013506285104 0.9992216976792808 0.9570605074408391 0.9995182398849867 0.9580967807881774 0.9995590884663084 0.9577978919330289 0.9992347083246835 0.9576023255240445 0.9991880124481327 0.9574125855327469 0.9996592045690551 0.9581531618010887 0.9996174286309648 0.9584990906850664 0.9997528171953255 0.9581697710109623 0.9997382738944366 0.9577510759027266 0.9997111472039474 0.9579323506074883 0.9989040127243809 0.9578539411910059 0.9578672024952014 0.9582454739162379 0.9578672024952014 0.9583842435424353 0.9578619354838709 0.9581540543821316 0.9573718546611205 0.9573406852976913 0.999695672466735 0.9581035105067985 0.9553397762970013 0.9582588104639683 0.9568434405144695 0.9582850646814741 0.9572141787737011 0.9575043706811451 0.9576869052224372 0.9578497892673364 0.9574613059609202 0.9577588374938817 0.9582115416769117 0.9569934695357835 0.9577795885686129 0.9990819492063493 0.9584158033749083 0.9902568849803287 0.8721905507016152 0.958008776319049 0.9982193532560214 0.9582504415066102 0.9571513958641064 0.9582642743340801 0.9573219633251833 0.9568778180062756 0.9580058056457658 0.9585430231990233 0.9897945441729323 0.957768175872093 0.9577939428433035 0.9572555254815899 0.9577807655038759 0.9578019331395351 0.9573027188825143 0.9578310299806577 0.9578259937137331 0.9578307738814995 0.8766812833930048 0.9578228700758503 0.9993489409722222 0.9993466348655682 0.9577858704156481 0.9578025886958649 0.9578087826937178 0.9993544367267202 0.9578343424623735 0.9579157217004449 0.9579527265991604 0.8764795096739997 0.9993494574652778 0.9992977502714441 0.9578393414932681 0.8801337899309992 0.8762359645827743 0.8765053260288618 0.877127004381161 0.9595367444629674 0.9594197486234138 0.9578634121374604 0.9579587533029066 0.9571067974180736 0.9445568172484601 0.957997874419741 0.9577407944267905 0.9580432069471625 0.9579359294636296 0.9577469889840882 0.957774818805093 0.9576217433234422 0.9577679965372249 0.9581513323390505 0.9581742101341282 0.9576304796044499 0.958329376247505 0.9581684483614697 0.9579784192269574 0.957968049566295

GCA_015683955 0.8710328745556122 0.9710416930342385 0.9563968398677375 0.9576286149519568 0.957636126040428 0.9706908800186742 0.8801852620599739 0.9717282403628117 0.9718338236669786 0.9715553939811623 0.8732142027938343 0.9718097023387668 0.8732469615663526 0.9573984074941452 0.9917108251561105 0.8790150262881177 0.9712175403225807 0.9575494917257683 0.9711714439503861 0.9574047001427891 0.9717188248795043 0.9574198857142858 0.9707238610129564 0.9575146880998081 0.9717656873957589 0.9573310472328244 0.9710096791696156 0.9573612700694945 0.9713885232857478 0.9571070496083551 0.9722899459713412 0.9573454787360417 0.9708005442978324 0.9708472633596934 0.9903294142728905 0.9573345885167465 0.9991119363674674 0.957284527540361 0.970906665089877 0.9574374114057733 0.9991161248948697 0.9571177580071175 1.0 0.9574473554301833 0.9707825402081364 0.9573729788235297 0.9717224309263989 0.9570987978142076 0.9717497473587505 0.9574971341320179 0.9710191578705828 0.9573653231939164 0.999113429951691 0.957558779733587 0.970784410171886 0.9571912917753024 0.972094940098661 0.957438979398532 0.9716176484230056 0.9572025545540797 0.8802424316658072 0.9571481770833333 0.9719186109839817 0.9574756516643226 0.9991250776332353 0.9573957692307691 0.9723509219348659 0.9571657660430973 0.9908636792452831 0.9570642742316784 0.9716558699719365 0.9572440568720378 0.9991200147772853 0.9571771781472684 0.9710962645452387 0.9573118341648847 0.9708272192007568 0.9571185365853658 0.9715530826197639 0.9574327328172649 0.9715066387531985 0.9572801705352914 0.9720172530573846 0.9712840181124881 0.9708430377045294 0.9712840181124881 0.9714028931855694 0.9709258612440193 0.9708100894749235 0.9707184385150812 0.9713300094184129 0.9574242027628189 0.9715798058252427 0.9910455942350334 0.9991354816369359 0.9908569501466274 0.9706339696466684 0.9906688508612874 0.9999594789379118 0.9916302615248228 0.9714656080760093 0.9903485413870248 0.9715481790837577 0.9714628323563892 0.9715488459759484 0.9716343628460307 0.9569987651677373 0.9710204639660858 0.9572263877266388 0.8811473095823095 0.9715872434701492 0.9562194432445406 0.9990647601790663 0.9708781188811189 0.9716242265128325 0.9711090982439489 0.9709126478089498 0.971641661971831 0.9715749267399266 0.9571236882310815 0.971228254444701 0.9712296744400832 0.9909828384376387 0.971246094579008 0.9713136945471349 0.9704702479732952 0.9712134194143417 0.9711892761641309 0.9712431273062732 0.8785008077025943 0.9709691252624214 0.9568018776671409 0.9568008479393652 0.9708108508158507 0.970826763814409 0.9708358545115412 0.9567053447050462 0.9710945417853752 0.9705638278649549 0.9706018009028273 0.8784005625327055 0.9566785714285713 0.9566995519203415 0.9707414652356511 0.8800608575765243 0.8778039314674341 0.8786174231861607 0.8797347868421054 0.9725150733468744 0.9724085054347826 0.9720571318732525 0.970564206180703 0.9911926335260117 0.947971324390244 0.9708636875145313 0.9708590438432836 0.9709630905695612 0.970822073797291 0.9708447818987637 0.9708662007001168 0.9715314446265513 0.9712080056048576 0.971520386869871 0.971497003516999 0.971507174269006 0.9711914101360862 0.9714851298853264 0.9708426036484246 0.9707685568720379

GCA_002333745 0.8700952123754588 0.9570716593992248 0.9977532095032399 0.9990061228905236 0.999088728118768 0.9577662121928165 0.876814812295974 0.95787115421228 0.9582395035117461 0.957532582538518 0.8706186179487179 0.9586860678451983 0.8705535245690764 0.9991680384948097 0.9577900548491648 0.8755802644937215 0.9571630853927086 0.9997216503642041 0.9571874734555985 0.9995917804466921 0.9570787754102374 0.9995239699122439 0.9579110097323603 0.9995372203389832 0.9581810034347399 0.9995585770505385 0.9581240353027702 0.9990225969074348 0.9585048019207684 0.9993954917355372 0.9582557791563276 0.9994568471993411 0.9579613614223088 0.9593061249697263 0.9566666528421566 0.9992098999148212 0.9580409610645638 0.9995508087930319 0.9580670293398532 0.9997859437751005 0.9581321639586411 0.9995238182191218 0.9576005665930833 1.0 0.9579773551698851 0.9994831795183161 0.957153 0.9994674666392938 0.9571964736451374 0.9996048973843058 0.9573337224352829 0.9995982954308368 0.9579058427518427 0.9995960175695462 0.9579720248538013 0.9992862783440815 0.9579051596009974 0.9996136421267893 0.957461175745625 0.9991652975814931 0.8756762106666667 0.9991661725293134 0.9565855514974433 0.9996774764103593 0.9578976645383104 0.9997144178983599 0.9579263255459624 0.9989370039641143 0.9577536280563103 0.9989254740570952 0.957332588609142 0.9995566002490659 0.9579991578167447 0.9995434299416179 0.9582810850222443 0.9995353528795812 0.9580285532746823 0.9995418328896171 0.957425933120236 0.9997747431781702 0.957355256378499 0.9986124236252546 0.9578872774480712 0.957502428674179 0.9581046475067551 0.957502428674179 0.9580565544359795 0.9574523255252355 0.957931888077859 0.9577686600436154 0.9571564537977746 0.9997218370607028 0.9575794180024662 0.9555937660485022 0.9580840693385787 0.9568623385904386 0.9581813629702002 0.9558288279911916 0.9576081904761905 0.9574200964785335 0.9573801026894867 0.9575259565539374 0.9576661735557167 0.9578965035652816 0.9569973924474188 0.9572708842364531 0.9988760271646858 0.9581970227885321 0.9896135630057805 0.8734360507720493 0.9577389411182583 0.9970234295227525 0.9581065811752989 0.9568416756889764 0.9581563683818047 0.9571817179111761 0.9565654391891891 0.9577228496503497 0.9582052936875457 0.9894423715693174 0.9574419379092893 0.9574017244724716 0.9574712426900586 0.9574124703030303 0.9574028737581779 0.956936967806339 0.9573954910498306 0.9573979898403484 0.9574573384950399 0.8767737998350289 0.9576797628941579 0.9993061284722222 0.9993052992194277 0.9576803538311371 0.957654865591398 0.9576878125 0.9993106711409396 0.9576972989639861 0.957575349814586 0.9576369839307788 0.8766468215417106 0.9993166883539364 0.9993195635179153 0.9576839907135876 0.8800657157464212 0.8762553541833735 0.8761020165598291 0.8772863170798147 0.959124458854044 0.9590471516441921 0.9570877125456759 0.9574698344926842 0.9572145452286281 0.944286769820972 0.9577388609756098 0.9574172275054866 0.9577995285784074 0.9577520342298289 0.957471739448646 0.9574868892149669 0.9574256382978724 0.9576153070827143 0.9579906523904381 0.9580592892644135 0.9574096406443618 0.9581859131736526 0.9580376181004475 0.9580726524541399 0.9580614558531748

GCA_015683995 0.8770168716182982 0.9918421958394621 0.9589796923783288 0.9594922194570136 0.9573235806060605 0.9888598569542254 0.8849273710691824 0.9771453932832523 0.9876395855734766 0.9892271917229731 0.8804832838245532 0.9924292640509014 0.8804329886125959 0.95936897115603 0.9698667994207099 0.884366196365472 0.9918294585987262 0.9568970198355105 0.9919936437331116 0.9569275819373635 0.9747671691436661 0.9571822179363549 0.9999828530203925 0.9572297635876187 0.9919036006753905 0.9570138387252535 0.9999458559437829 0.9568912943176298 0.992237658821079 0.9570523099133783 0.9900164185544769 0.9569164108790676 0.9999509211037699 0.98842436064052 0.9718733113311331 0.9571947319912686 0.9723152128603105 0.9571441839191142 0.9999437050781249 0.9569593790457924 0.9723139209591475 0.9569501511879049 0.9697389538754162 0.9569040182210501 1.0 0.9568696727622714 0.9900370435153584 0.9568693588816582 0.9778491012743125 0.9568758213256485 0.9884044571187158 0.9572321542168676 0.9722877814716312 0.9572910255791505 0.9999906572860447 0.9568849459783914 0.9881546293065853 0.9569045664739885 0.9887246407312925 0.9568980149181907 0.8796059494660068 0.9568113506368662 0.9770206382032466 0.9568870189312246 0.9722647501105705 0.9569932547510225 0.9869983459178857 0.9569137700983922 0.9720118347806725 0.9567391904191618 0.9873150721318985 0.9568603330138956 0.9723139933333335 0.9569185783016598 0.9999789012925969 0.9572265149325627 0.9999299014492755 0.9569505468937396 0.9887926766821836 0.9568699256416406 0.9887639470311834 0.9585060349920472 0.9872186943552089 0.9890338430034129 0.999868480392157 0.9890338430034129 0.9920437402978813 0.989396120576671 0.9999555695466873 0.9873269131481077 0.9920651017838406 0.9568409601146405 0.9887878851451579 0.9707337401839803 0.9723088965364122 0.9690192814227347 0.9997442066131873 0.9690024400095034 0.9697460307328605 0.9696880585043645 0.988699941990183 0.9718303026315789 0.9875694727668847 0.9915788459126071 0.9875955553135888 0.9779027937569678 0.9570807047619048 0.999945073313783 0.9584760524530862 0.8830221812080536 0.9767319158134242 0.9585439468735308 0.9723880941704036 0.9889888474795347 0.9887720188101488 0.991838303401648 0.9902798786459442 0.9767940577716644 0.9881772161874334 0.95878265625 0.9880523200170903 0.9880326251604622 0.9704450767148015 0.9880368827556697 0.9879804253954682 0.9875634044894366 0.9880568082835184 0.9880634777967547 0.9880716741405081 0.8774353953363709 0.9875298891545317 0.9583390177133656 0.9583397516099357 0.9876558634188779 0.9877111111111111 0.9876658568943019 0.9585684112149533 0.9879223555754592 0.987464369266055 0.9874785135135135 0.8835049740420271 0.958579790241678 0.9582058965047664 0.9876677907732295 0.883846997299288 0.8838487863715493 0.8768711713836479 0.8839210291897333 0.9874254662029993 0.9873774711140179 0.9890797530864197 0.9888974982911825 0.9693730754444979 0.9494995917351259 0.9878244983748645 0.9876955524140932 0.9878999218241041 0.9879492819843343 0.9874727363616606 0.9877327755991285 0.9750563372231484 0.9745812565073355 0.9751048536759459 0.9747029995242626 0.974922909134158 0.9747887222354907 0.975060981887512 0.9881526568758345 0.9880579571810885

GCA_002333765 0.8706377713837301 0.9569840884912959 0.9976606486486488 0.9988966168831168 0.9988746050011065 0.9577622182849043 0.8768963535762484 0.9576922504752851 0.9581146782776973 0.9575008339429409 0.8707632114548709 0.9587097925113284 0.8706630266529984 0.9988967200346172 0.9575091734921819 0.8757968812930578 0.9569909616792777 0.9991553798256537 0.9570435420178184 0.9992021561181436 0.9570495916870414 0.9992013745560896 0.9579953848021364 0.9991295515125873 0.9581225006110977 0.9993237116818559 0.958197703396042 0.9989023841760102 0.9585855316091955 0.9990606685926536 0.9581525939663699 0.9992262276188515 0.9580903229723167 0.958985486127865 0.9564171365960554 0.9987278276595745 0.9577114783035058 0.999295175566175 0.9580985114690093 0.9995164074743822 0.9577739842983317 0.9992137662070385 0.9575723416179338 0.9996021190188983 0.9581636264003897 1.0 0.9572522040028936 0.9992312229771464 0.9573574950592886 0.9993634240225716 0.957245418660287 0.9992244490818031 0.957688858123009 0.9993488525963149 0.9580703372967727 0.9989897980428899 0.9578500024844722 0.9994466298568507 0.957541766297663 0.9989657175828788 0.8753809257294429 0.998937457591623 0.9567356948493684 0.9994928304914744 0.9575902200488998 0.999464202310967 0.9578867719120991 0.998883942247332 0.9574764073619634 0.9988030574712644 0.9573541715399609 0.9993671568219634 0.9576210698475159 0.9993315856457333 0.9583722033481044 0.9992334721640854 0.9580479566488067 0.9991851894327257 0.9574448710390568 0.9995000281293952 0.9574836875926842 0.9983700701198825 0.9578746061053669 0.9577619631314341 0.9582186232239097 0.9577619631314341 0.9582077900416974 0.9577232174752596 0.9580162327272728 0.9575245248432224 0.9570418703121221 0.9995354345214257 0.9575817196353782 0.9550232978219697 0.9577248016650343 0.956893752764807 0.9582103700998296 0.9557638852258852 0.9575066068831799 0.9572253775853777 0.9575118959198632 0.9573085408464568 0.9575033870967742 0.9578073934345909 0.9568085734698739 0.9572606457156887 0.9985445072217501 0.95826290913532 0.9898252596353566 0.8738180321910696 0.9576260723069356 0.996637103218646 0.9577342087042533 0.9571743823818898 0.9582692839383394 0.9571134631297153 0.9566214230676618 0.957738479880775 0.9581983041788145 0.9895613044496487 0.9573574032882012 0.9573277568286198 0.957200504854369 0.9573553191489363 0.9573708996372432 0.956917184079602 0.957418269787645 0.9573890494571774 0.9574254756156446 0.8768449181222708 0.9576382609756097 0.9990626913901541 0.9990932408411013 0.9575330377588307 0.9575463468095471 0.9575575188702216 0.9990695170023826 0.9579059164619165 0.9575851354679804 0.9576133489288353 0.8764022216372729 0.9990937294015612 0.9990946714378659 0.9575856891924862 0.8798158853373923 0.8765595991505177 0.8762680708245243 0.8773017258195612 0.9591120320226043 0.9590266933962264 0.9573543357664234 0.9576021727686049 0.9566640621915103 0.9443650331294599 0.957722103725347 0.9574382269849003 0.9578632454856028 0.9576240243902439 0.9574653399951255 0.9574833040293043 0.9572310199556542 0.9574192387287511 0.9577980900544286 0.9578287209302326 0.957189106351551 0.9580430748197865 0.9578301460396039 0.9579308135509398 0.9579212119713084

GCA_015684015 0.8785614303208424 0.9891004071729959 0.9588444790474009 0.9593882105025918 0.9573970629882811 0.988419944680851 0.8850067463876432 0.9781824286647789 0.9899833670997579 0.9897515579034597 0.8810209722863742 0.9899064939810834 0.8810426811761982 0.9593075373472157 0.9702107911928382 0.8850701854636591 0.9891093068254305 0.957241433447099 0.9897078906577295 0.9572156617647058 0.9763345522562544 0.956797126106724 0.9897353239079975 0.9568349419167472 0.9896144151105388 0.9570233590920069 0.9897249292203677 0.9569215047249818 0.9897656998100064 0.9570377906137185 0.9976315075062553 0.9565863539651838 0.9897677655522642 0.9899730832570905 0.9716096923412612 0.9575786506199855 0.9732402419533851 0.9568899521416607 0.9897197912673056 0.9566108815097946 0.9732486579414374 0.9568102348430385 0.9702087071428572 0.9564927023155885 0.9897388848768056 0.9564001655073159 1.0 0.9567666698795182 0.9783651443685255 0.9565576334051208 0.9896048631710883 0.9569914398658362 0.9729765342800089 0.9570190591333494 0.9897211080566717 0.9568373425903758 0.9894045130079555 0.9565756491480681 0.9897442170706007 0.9569270701248799 0.8800128493929215 0.9570487560038425 0.9774759482569612 0.9564436669851888 0.9730477234513275 0.956700731414868 0.9888296241484615 0.9573766038191928 0.9726507715981535 0.9572704273297924 0.9887471064866061 0.9568456453154877 0.9729455758520829 0.9568288422818794 0.9897236530655843 0.9568356551889049 0.9897456730360935 0.9569011624013394 0.9897458239230447 0.9564093785850861 0.9897568472280999 0.9591749965556833 0.9890033333333332 0.9888669849133078 0.9897122198505871 0.9888669849133078 0.9899440499784575 0.9891470150537635 0.9897261064189189 0.9902376681127983 0.9896090408077409 0.9563733444497381 0.9899308471377647 0.9712076722783392 0.973255866962306 0.9701263941500841 0.9898149149298171 0.9698348689852311 0.9702175769048745 0.9703475735637552 0.988678391959799 0.9723780528052803 0.990001876479449 0.9896739059829059 0.9894283180558517 0.9783991633815154 0.9571856122203513 0.9897728444632291 0.9593129667863555 0.8842997473182359 0.9774649798115747 0.958630065681445 0.9730298607680216 0.9897029617901626 0.9896087705272257 0.9891412725344644 0.9895160394190872 0.9775116311735387 0.9906075932132384 0.9595693483249197 0.9889106994328921 0.988943240117746 0.9712589422423555 0.988850741908365 0.9888766897856242 0.9883320436379347 0.9889096622613803 0.9889349139739824 0.9889145071308724 0.8779612771595124 0.9880189548744147 0.9587283936022253 0.9586774275110185 0.9882619104477612 0.9883157795242141 0.9883019188900748 0.9591334202103338 0.9895832460732983 0.9894910746064339 0.9895603761112378 0.8845209275079328 0.9591255730954015 0.9589256796892849 0.9882813015737983 0.8865151873338168 0.8845717946186126 0.877336118077325 0.8848078635907723 0.9896557088204037 0.9895692353567626 0.9961116528239202 0.9886150608108107 0.9699440880503144 0.951012750310559 0.988808096657441 0.9887595146461408 0.9888489833759591 0.9889668564725955 0.9884971184266781 0.9887855026795285 0.976518304964539 0.9764181109799291 0.9765403546937811 0.976519203497991 0.9764995553453168 0.9761967600950119 0.976394950331126 0.9886704149377593 0.9885434888304862

GCA_002333785 0.869927891566265 0.9569790368753032 0.9978698083135903 0.9990902276176025 0.9990955508942372 0.9583098961284232 0.8764390442573989 0.958708687470224 0.95860380295328 0.9577407029145236 0.8703383226632522 0.9585686560038197 0.8701708132375577 0.9992014230103807 0.9576877935323385 0.8761192346802248 0.957007587890625 0.9993323689771768 0.9571296041515811 0.9993362273018587 0.9575170360382447 0.9995907245173346 0.9579185662211421 0.9996108079748163 0.9580444212849436 0.9995985687808896 0.9581105603131883 0.9990810149820638 0.9585061551352645 0.999418721789085 0.9581770500743678 0.9993189872624766 0.9579643471918309 0.9590961108422073 0.9569678057114962 0.999205917721519 0.9583496545768567 0.9997072475738179 0.9581187515269973 0.999642358490566 0.9584429117357003 0.9995832925336597 0.9575360965346534 0.999704206854094 0.9579658502073676 0.9993323731958763 0.957690087124879 1.0 0.9576715681424685 0.9995345912078882 0.9576653502879079 0.9996677519701369 0.9582980729423362 0.9995868478937538 0.957969875942593 0.9992496234223051 0.9577068491786958 0.9996235569358178 0.9579402321560879 0.9992821303258147 0.8751548417132218 0.9992600395668472 0.9572296486090776 0.9994431664272523 0.9582423334152939 0.9996148243075652 0.9581006259503295 0.9989922279792747 0.9579030206794683 0.9989745381110191 0.9574371404193077 0.9996088895794489 0.9582989990113692 0.9995948220603539 0.9583524753451677 0.9997493773427739 0.9580114560506452 0.9996277314343845 0.957659704942218 0.9997232615826158 0.9579202384500745 0.9988558871515976 0.9579987870553359 0.9577394685180751 0.9580969488622462 0.9577394685180751 0.9581216879293426 0.9577686868198307 0.9579127191065792 0.9572279462759464 0.9571447987390883 0.9995790222494388 0.9580257793171698 0.9555586332303304 0.9584427035027134 0.9571825703703704 0.9582166919130222 0.9569554735034348 0.9574182339901478 0.9576557266811281 0.9579402230938955 0.9577830577492596 0.9575052426237504 0.9578757765801078 0.9568269737159394 0.9577950884955753 0.9989671999154692 0.9582393392070485 0.990078773432076 0.8722440783898305 0.9580890348923533 0.9981720698864901 0.9583915107018417 0.9572475375153752 0.9581011533681333 0.957031188384578 0.9567073717176584 0.9582184868913857 0.9582653484405458 0.9898833505882353 0.9578026574612405 0.9577655256782948 0.9575485981990753 0.9577955972861644 0.9577828793019875 0.95730255994006 0.95775116194626 0.9577310019361085 0.9578208861985472 0.8762041538461539 0.9574836794527242 0.999289880900823 0.999289757628219 0.9575154345703126 0.9575201148301978 0.9575230029296876 0.9992757525951558 0.9578059068506652 0.9574781598421312 0.9574942441430334 0.8760394296276737 0.9992828849902536 0.9992952579107066 0.9575077707162063 0.8796826670066293 0.875722645799893 0.8760220666666667 0.8766675941479275 0.9589649702876158 0.9588743075453678 0.9575650401753106 0.9576995951126018 0.9572079271014134 0.9441446560981845 0.9578172625152626 0.9575642591687041 0.9579505135730007 0.9577845102840352 0.9575936445856758 0.9575596300832926 0.9575952909135925 0.9577585417696491 0.9580968504719324 0.9581308614697122 0.9575896932211777 0.9583071596009975 0.9581325869781313 0.9578278270564916 0.957828275520317

GCA_015684025 0.8780753343058595 0.9784895490367775 0.9599563605130556 0.9604579275427544 0.9582087239709443 0.978767313865359 0.8876252501238238 0.9990620373755842 0.9778406197881451 0.979241003415884 0.8825389322617682 0.9781082045556052 0.8824962310170275 0.9602607744411831 0.9714461070329149 0.8873232000993049 0.9785166777335103 0.9573998212128534 0.978919233441911 0.9576287190082644 0.9983843836805555 0.9581263909593652 0.9782795646466856 0.9582481321350498 0.9785503611788167 0.9578856399132321 0.9783124168853893 0.9581782548543689 0.9785851955184534 0.9578681695892385 0.979729212667693 0.957458308433735 0.9783151544359255 0.97766004575006 0.9736576507187299 0.9581193834951457 0.9741811113508848 0.9581511592111592 0.9783735093509351 0.9574575215105163 0.9741389336492892 0.9578464268585131 0.9711791929179122 0.9574645204496531 0.9783485334796926 0.9575563865546218 0.9795232251082251 0.957667496399424 1.0 0.9575159372755566 0.9789240778159932 0.9581209215591916 0.9739335812850367 0.9583918337349396 0.9782937234740757 0.9578567449101796 0.9794198869680852 0.9576086211866444 0.9791072939145447 0.957997056848165 0.8812998923904689 0.9580215726290515 0.9982448986346713 0.9575580910179642 0.9739029647160069 0.9575589887371198 0.9775753238963532 0.9580730314676916 0.9741932979414951 0.9579310307544451 0.9775790740740742 0.9577848945855295 0.9740432577720207 0.9577592016303045 0.9783148151408451 0.9581652441664663 0.9783061246434058 0.9577896981312891 0.979100782627392 0.9574287210136266 0.9790039687364308 0.9601267084854014 0.9776695651157354 0.9770814161578205 0.9783317085537918 0.9770814161578205 0.9786192403376276 0.9781884377104377 0.9783194338797815 0.9782423092236231 0.9789853487863548 0.9574156672236809 0.9793676387988767 0.9727703148392779 0.9740577828054301 0.9714258963486455 0.978391290393493 0.9712842017005197 0.9712308685714285 0.9718742682363803 0.9777651418198321 0.9737174532157454 0.9787290145502646 0.9782788505747126 0.9786196331282954 0.9999147692307692 0.9579894403074707 0.978383952266258 0.9597122251832112 0.8853277127281346 0.9983741670172487 0.9594963963542885 0.9741725070729054 0.9798056849912741 0.9792938087843832 0.9785743946089263 0.9786560312635694 0.9983523445788245 0.9796701835456705 0.9601589849795176 0.9784014497492914 0.9783124934497817 0.9725947169394075 0.9783872167648986 0.978413038637852 0.9776043925859759 0.9783709457398126 0.9783194358527554 0.9783629389978216 0.8805903771097046 0.9776346040150011 0.9597444958563537 0.9597206242801198 0.9776031407146007 0.9777076323303319 0.9775994304635762 0.9600052510269285 0.9785297844827586 0.9773088886255926 0.9772983562292754 0.8862232243902439 0.9600349463347796 0.9598973529411765 0.9775084062431304 0.8868600723065799 0.8858797124600638 0.8787659025270759 0.8855646104057755 0.9783943546255508 0.9782666041482788 0.9793605622402101 0.9763144832468145 0.9714133633562456 0.9517958361942128 0.9778975698262592 0.977680302094818 0.9779269620811288 0.9780199889940567 0.9776659572121748 0.9777249701920953 0.9852892025664528 0.9852277190574239 0.9853339061784898 0.985247108874657 0.9852614874141875 0.9848775051879181 0.9851524868209949 0.9783678763258858 0.9782622252374491

GCA_002333805 0.8706479538904899 0.9572801907291164 0.9978708614393127 0.9989832347863618 0.998954157105031 0.9581125194254768 0.8770159455084974 0.9582817953576505 0.9585105033718689 0.9578858129882812 0.8709521168631473 0.9589518891268142 0.8709434146341464 0.9990471302285469 0.9580394433399602 0.8756609483443708 0.9573071634146341 0.999433789604473 0.957373872985326 0.9993194697128485 0.9573767521367522 0.9994141947565544 0.9581112391093902 0.99938492 0.9583836021505375 0.9995177404737384 0.958323972201902 0.9990162452431289 0.9588533628318585 0.9993481616741896 0.9584431736231168 0.9993021701602959 0.958175203685742 0.959320451145959 0.9569902283770649 0.9990864618644066 0.9580618261933905 0.9994940318906608 0.9582653193564116 0.9996517365149389 0.9581556758080314 0.9993838913934426 0.9576838407208962 0.9996743689903846 0.9582958836416748 0.9993173296947645 0.9576718184007709 0.9993514634146342 0.9575141623360555 1.0 0.9576323106423777 0.9994673078520979 0.958006819960861 0.999395187032419 0.9581325665859565 0.9991143980904942 0.9579579394991322 0.9995654034729315 0.9578286798029558 0.9990864448629421 0.8755008666843361 0.9991656042579838 0.9569248711089494 0.9995523545872478 0.9579790265918517 0.9996606618240518 0.9580162332065907 0.9990567477646081 0.958251237745098 0.9989847028262677 0.9574046926829268 0.9995119622876089 0.9581050490677134 0.9994700997920998 0.9584338357511679 0.9994670672776504 0.9582250024342746 0.9995190484922576 0.9577818472119873 0.9997174874974996 0.9578494238377843 0.9986692564971752 0.9579522509861934 0.9579859001910219 0.9582443455882352 0.9579859001910219 0.9584310292311472 0.9580701781844451 0.9581175447508468 0.9577007771896053 0.9575356364956438 0.999613355263158 0.9579027633851468 0.9557022043138185 0.9581591905111276 0.9571150442043224 0.9583015871470301 0.9557074258629072 0.9576215429262395 0.9575470226070226 0.9576854507696068 0.9578278051181102 0.9577240312124851 0.958105785225049 0.957184744351962 0.9575408041186565 0.9988110185967878 0.9583736594291291 0.9897252129864148 0.8740388097713099 0.9579958024691358 0.9970813799203893 0.9581758370480438 0.9570300859317458 0.958279923096006 0.9573750390434357 0.9569041700696612 0.9580703284399104 0.958407638483965 0.9893318394570559 0.9576532422630561 0.9576042263056093 0.9578006842999273 0.9576159052453468 0.9576045915901402 0.9572156470881035 0.9576655646718146 0.9576662958494209 0.9576704490584258 0.876681031096563 0.9577843832276938 0.9991451794982701 0.9991523520518358 0.9577939239580795 0.957802332276165 0.9577935153583619 0.999179098962835 0.9577682637524558 0.957661565731167 0.9576941067913386 0.8767161409483888 0.9991413442835533 0.9991424762110728 0.9578408367894609 0.8797653036848793 0.8765862383859835 0.8764311710037176 0.8776249837750136 0.9592481007025763 0.9591742410714287 0.9575609123999032 0.957832843137255 0.9571796762234306 0.9443137166115493 0.9580600535931791 0.9578458016569201 0.9581574572962421 0.9580980161250918 0.9578141459248413 0.9578153567937439 0.9577104370370372 0.9578364136061128 0.9581684542977458 0.9582149158415842 0.9576690036991368 0.9584316732968672 0.9581536320871503 0.9582969629629631 0.9582868733020499

GCA_015684055 0.8774899112863479 0.987069407710532 0.9559666456551102 0.9589805278396436 0.9561141644815256 0.987944445887446 0.8844004446119067 0.9778102696777022 0.9879548615111898 0.9994615347303066 0.87928680398056 0.9883225234641639 0.8791646160983531 0.9585858569832404 0.9693174008175041 0.8825261598785733 0.9870404797047971 0.9557876753030664 0.9875166624068158 0.9559892102736437 0.9752886601761707 0.9560924762357415 0.9879093687778014 0.956072611725132 0.9883441594265857 0.955950879695673 0.9887514558979809 0.9557269465648855 0.98819899516502 0.9558052789598108 0.9901154603717156 0.9558393856084151 0.9880295309847199 0.9877349248035916 0.9722057975460123 0.9562866315789476 0.9720945409652078 0.9561485771875741 0.987954901793339 0.9557945190262349 0.9721547056189838 0.9558294042553191 0.9692651163337251 0.9557218132387708 0.9878792695911414 0.9556793038124556 0.9898630458676813 0.955744037458511 0.9779817660142348 0.9557171114265437 1.0 0.9562305716318785 0.9721024017857144 0.9562344924098671 0.987873161795849 0.9556482269837533 0.9879312537377188 0.9557212271540471 0.9992555257009346 0.9557340824058728 0.8769200420278433 0.955732701108752 0.9778171628526994 0.9556670793950851 0.972094566911601 0.9557594126006632 0.9859945821791322 0.9557673221855865 0.9714625299069561 0.9557251684334511 0.9861906745588566 0.9558099269040321 0.9720710720680394 0.9558658996212123 0.9887030711369009 0.9561843327802798 0.9878707707047051 0.9557505828220859 0.9994118069259044 0.9556461720226844 0.9993121699038651 0.9576561040145986 0.9863516819914778 0.9859766354733406 0.9879430826906598 0.9859766354733406 0.9881448051382539 0.9864279070256626 0.9879410972251641 0.9871930523571584 0.9873972036082475 0.9556071051390853 0.9995765953723508 0.9708148319511131 0.9722045822162645 0.9685271489766778 0.9879193931005112 0.9680274330042313 0.969265368986455 0.9699091747002117 0.9889391095890412 0.9722309045335658 0.9876543704340353 0.98843657014157 0.9876231030042918 0.9779311206706376 0.9560087054098747 0.988807617120954 0.95841586627513 0.883226972757163 0.9771117267637177 0.9578620217441592 0.9719152270160379 0.9879874177908938 0.987348799140709 0.987160518918919 0.9873880893970893 0.9770578134176644 0.9865914357157742 0.9581721859180856 0.9979789853639242 0.9980036084718924 0.9706267270318022 0.9979734349030471 0.9980570627847098 0.9971066097213749 0.9979865519968367 0.9979818438735177 0.9976551604014958 0.8760283391281091 0.9985717880531852 0.9572230840488025 0.9571942346823423 0.9985324254983224 0.998511673549656 0.9985322281428854 0.9574922164832697 0.9864435426695843 0.9871881840408979 0.9872650011108642 0.8831759077306732 0.9574980103417267 0.9575873068283918 0.9987788576519502 0.8853035273074172 0.8827113602015115 0.8758275091098386 0.8829210911392406 0.9875727923322685 0.9874218348623853 0.9892595715213157 0.9857445539599651 0.969840562635249 0.9495625147637796 0.9983504283749264 0.9983069859210787 0.9983916686343959 0.9984731682389938 0.9982751618026603 0.9983157423580786 0.9758649515938608 0.9751698329018593 0.9760847235349716 0.9760111756373938 0.9759454539007094 0.9756859724203519 0.9758219038598153 0.9876976178879787 0.9875723054690976

GCA_002333825 0.8701613008989952 0.957049321002677 0.9979914662629759 0.9995613688792166 0.9990552294391488 0.9585224462809917 0.8767629198473283 0.9589432936129648 0.9592529795520934 0.957954699386503 0.8703357039149598 0.9589302445456725 0.8702124128058304 0.9996147644888214 0.9580240539865034 0.8764735105234754 0.9571456813159833 0.9993936792249731 0.9572421297192643 0.9994562734000871 0.9575020536813594 0.9996626029962546 0.9581685086648767 0.9996402150991144 0.9580943573359549 0.9995750188679244 0.9583886083107942 0.999433443921233 0.9588484724560982 0.9995335377555279 0.958231569162122 0.9994986791250796 0.9582382958984376 0.9592836387878788 0.9567934852941177 0.9994266383340416 0.9584271098982883 0.9996564021492045 0.9582824398034397 0.9996339754269055 0.958535023532326 0.9995276447395726 0.9577838129496403 0.9995417136297223 0.9582642303921568 0.9993140785879386 0.9577468303895477 0.9995863715710726 0.957822100024882 0.9995567182597783 0.9577122371902813 1.0 0.9582361034141517 0.9996674979201332 0.9582080375884793 0.9996260768431984 0.957892897755611 0.9995742845100106 0.9578986547972305 0.9994921292217328 0.8759541434689507 0.9996580700650305 0.957258515797208 0.9995361601170569 0.958240631786772 0.9995672580306529 0.9584462222222223 0.9995598091042586 0.9580017743132888 0.9994842698512466 0.9575620292682928 0.9995899177735611 0.9582929238024324 0.9995405185185186 0.9585537177579364 0.9996864489286457 0.9582701345072145 0.9996581897086175 0.9578016756889765 0.9996239587242026 0.9579264870903674 0.9993540388571428 0.9580413478153542 0.9580217818444499 0.958343800246609 0.9580217818444499 0.9582813269325456 0.9579915483714148 0.9581677195121953 0.9576666871015204 0.9572002721749696 0.9996962144192811 0.9580868663366338 0.9560523346860825 0.9584214985163204 0.957054589108911 0.9584115775269872 0.9572012133891213 0.9578296582466567 0.9580423229873908 0.9578910215580598 0.9577262527881041 0.9580489327772326 0.9579901800246609 0.9573042275042445 0.9579029977739303 0.9994833077578857 0.9584075442477876 0.9903505456234037 0.8726713656975192 0.9584851480467778 0.9986918706645783 0.9583787334167709 0.9574364915758178 0.9583909700000001 0.9571930242231466 0.9568011100362758 0.9585747036664992 0.9581372816007808 0.9900670259433961 0.9578135651750972 0.9577246415552856 0.9578577726161369 0.9577587214389888 0.9577595697617891 0.9572202226670001 0.9578153833090735 0.9578113024496726 0.9578294878640777 0.8766946951724138 0.9577251838235294 0.9996623185879276 0.9996471838955386 0.9577502033815242 0.957756842234199 0.9577815275397797 0.9996117604554412 0.9579083210059172 0.957858543111992 0.9578861168895493 0.8760404994686505 0.9996508999782089 0.9996493699585786 0.9577844229355552 0.8797556415530984 0.8758083760453198 0.8753928154552825 0.8768943558114035 0.9591164906019509 0.9590515946526617 0.9576284082031251 0.9577735411651419 0.957544906600249 0.9443616208579504 0.957928124387855 0.9579093465491925 0.9579925079754601 0.9578561291114386 0.9579482724822347 0.9579624263984299 0.9581901518924303 0.9577404080118693 0.958177697090276 0.9582221043478261 0.9581556559621608 0.9584051748251748 0.9581978001491425 0.9579877554563493 0.9579887847222222

GCA_015684095 0.8774591414634146 0.9728332712353072 0.9593109553550777 0.9596375667118952 0.9577974615196679 0.9715766506520247 0.8856545383253225 0.9739628337696334 0.9717984422227389 0.972998449339207 0.8810894034682082 0.9725351178451179 0.8811614010670379 0.959236892412231 0.9915293683495585 0.885117078967544 0.9728343186395166 0.9575069337554798 0.9730768270709733 0.9576658525232729 0.9713312371615312 0.9576661755409677 0.9723706759751772 0.9576960362567368 0.9732115993683735 0.9575171121426828 0.9724390457168219 0.9575569928869267 0.9726678739810531 0.9575575425376762 0.9740873392010833 0.9575597404505386 0.9724093920568003 0.9704460648148147 0.9907791772560844 0.9577551835535977 0.9999921909020217 0.9576305660835763 0.9724965105908585 0.9574300580691991 0.9999604710215159 0.9575525006063547 0.9981477978106891 0.9573873910939013 0.9724550812374806 0.9573000896752302 0.9736951326644371 0.957405296836983 0.973807454746137 0.9575385658820676 0.9725801286887066 0.9576806517509729 1.0 0.9578788458728998 0.9724376718403548 0.9570718379160635 0.9737530965391621 0.9575783580276901 0.9727903741648107 0.957168385693572 0.8792823858000521 0.9570782219541617 0.973579129287599 0.9573216549891278 0.999988286321484 0.9575323981571291 0.9711733414813004 0.9571946755126658 0.9909542114347357 0.9571078404434803 0.9712950346475507 0.957424264955195 0.9998446623856783 0.957406804848485 0.9725904287628404 0.957701229946524 0.9724559813291843 0.9574275351089588 0.9728296025754883 0.9574023862536302 0.9726010251233738 0.9589347296058661 0.9713823575873625 0.9703837195413283 0.9724958802502234 0.9703837195413283 0.9730218079604228 0.9718196334478809 0.9723974589800445 0.972274813466788 0.9733094307862681 0.9574010043457267 0.9728267385504669 0.9911186506746628 0.9999988620089028 0.9909177621247112 0.9722156343366778 0.991125826388889 0.9981896583386718 0.990657322064057 0.9720255985095483 0.9907173235484568 0.9727716154021415 0.9725683859413476 0.9727695355191257 0.9740436236016671 0.957037021686747 0.9724621884700665 0.9589658393177739 0.8847084244917715 0.9730792633476009 0.9587707995283019 0.9998502871189774 0.9730034152389538 0.9736122146584978 0.9728386578829834 0.972938961615265 0.9729848797094871 0.9741955438132982 0.9600513460653474 0.9720977028238459 0.9721134386200717 0.991716801724138 0.9721196932377967 0.9721597106974659 0.9716174375000001 0.9720753369151557 0.9720315864846721 0.9720987505597851 0.8782961469873007 0.9709560871528562 0.9585529026303646 0.9585161738929889 0.9710036739769387 0.9711430609948232 0.9710426915254236 0.9587928404936014 0.9723241711480008 0.9703711170084439 0.970413932963588 0.8857629247633284 0.9588401807366735 0.9585624657534247 0.9710414758900406 0.88523862956607 0.8843542245051366 0.8781879543067227 0.8846952655654384 0.9729957338727805 0.9728717470694318 0.9739583224792274 0.9700742707589818 0.9903049517760528 0.9485859125521987 0.9714530207394049 0.9712615215916799 0.9714995235944908 0.9715173211050724 0.971086916309497 0.9712573703284259 0.9714240531561462 0.9711775491822708 0.9712892479240808 0.9713142575901329 0.971196535451743 0.9709124105865523 0.971225504630729 0.9721022847222223 0.9719497237697308

GCA_002333845 0.8697839422572178 0.9570351464536431 0.9982583098896344 0.999613776330076 0.9992090623617869 0.9584881974958659 0.87644908569869 0.9588011882129278 0.9588526660180006 0.9578391216050893 0.8698929548387098 0.958823122614504 0.869748582166926 0.9996635695708713 0.9579473766816144 0.8761962035541195 0.95700389201075 0.9994812036838724 0.9571587493975904 0.9994169828148792 0.9576714425907753 0.9998109096573208 0.9580582506082724 0.9995811374606507 0.958058809115413 0.9997044712041886 0.9582135154336109 0.9995678541046364 0.9586824844274078 0.9997299916475255 0.9582856042595345 0.9994641786317567 0.9581262448284256 0.9594434050785974 0.9570140377543515 0.9994973461128859 0.9582230482996551 0.9997434022750775 0.9582451114922813 0.9997022490628905 0.9582982405125676 0.9997108753370672 0.9575924274013403 0.9997052755741129 0.9581410752688173 0.9994673769117955 0.9578640628778718 0.999716003327095 0.9580183374628345 0.9996095119916579 0.9576247935669707 0.9997962396179402 0.9581346297662975 1.0 0.958093336578243 0.999646799501868 0.9580925292215866 0.9996316003369131 0.9578049753694581 0.9996433431146511 0.8753208888888889 0.9996981858129317 0.9575337301393303 0.9994937776387149 0.9581181434184676 0.9997319429290812 0.95849320730779 0.999559178197065 0.9581926944924674 0.9995699392924428 0.9577833162393162 0.9997425010526315 0.9582448701459313 0.999707211762217 0.9583128620774735 0.9997763988343047 0.9580993754574287 0.9997125853154085 0.9577058820638822 0.9997380998959418 0.9577408358061326 0.9994568059972742 0.9581093218249075 0.9579088747600768 0.9582881282240234 0.9579088747600768 0.9581890110429448 0.9578711897261935 0.9580593025516404 0.9576527769607843 0.9571583159680155 0.9996991058091287 0.9579700915162009 0.9561051798047154 0.9583015210435639 0.957038661902415 0.9583335824175825 0.9571299411476213 0.9575550813208475 0.9578124535585042 0.9579271642156862 0.9578038261515603 0.95775586366968 0.9580480083599704 0.9573151730815783 0.9579277142857143 0.9995303961661343 0.9582348445532436 0.9905392910101947 0.8717959485274609 0.9582779896142433 0.9987650480876762 0.9582917774458553 0.9574898045038357 0.95831800996264 0.9570571418110813 0.9566944465878949 0.9584129116766467 0.9583570145985403 0.9900150801508723 0.9577877219796217 0.9577925078826098 0.9577097759922086 0.9577493014795053 0.9577358917738414 0.9572345284431137 0.9577990801258776 0.9578049213265553 0.957808985472155 0.8763322971114167 0.9578206751467713 0.9997947673913044 0.9997985165073849 0.9578502544031312 0.9578447260273972 0.957874883891469 0.9997647080691013 0.9580557548100641 0.9579344353842352 0.9579566592537684 0.8761179925650556 0.9997997347249401 0.9997971636917719 0.9578624920440636 0.8795535857289529 0.87564522335845 0.8755834970477723 0.8767443074829931 0.9592137408022786 0.9590100691299166 0.9578766130212144 0.9577179659390741 0.9572471198213843 0.9441135139279326 0.9579629902200489 0.9579702247191011 0.9580567670093001 0.9579257635829663 0.9579748973105134 0.9580030227997057 0.9581343168316832 0.9577086509108813 0.9580907715133532 0.9581250902348579 0.9581238467243511 0.9584044734227521 0.9581913603759584 0.957981286737466 0.9579816473203259

GCA_015684115 0.876917941757157 0.9915457603881038 0.9590412995391705 0.9594154942058625 0.9571795239251882 0.9883092975115614 0.8847519934227169 0.9770672458943631 0.9872228606686113 0.9891129010383557 0.8803118742724099 0.9923499808306709 0.8801205622958471 0.9593698036977858 0.9696512369633763 0.8844528807106599 0.991534662262945 0.9569464421206226 0.9916688100208769 0.9569196176327326 0.9744952222222222 0.9572033550724637 0.9999732354078082 0.9572642668298653 0.9919162169031985 0.9570590928935241 0.9997422152519114 0.9571019902557858 0.9921797594645473 0.957081252417795 0.989753261811877 0.956783035540409 0.9998537270597423 0.9881578064666202 0.9717450509525918 0.9572943010490365 0.9720222480446927 0.9572720255915017 0.9998058266168667 0.9569598173515982 0.9720640696895244 0.9569375392038602 0.969423780487805 0.9568877879297908 0.9999822574372934 0.9569517302123554 0.9899998179871521 0.9568868946350895 0.9775445413769903 0.9568923028695443 0.988296042814752 0.9571859173313996 0.9720496454058877 0.957298551005573 1.0 0.9568602579556413 0.9878085686188811 0.9570231301402998 0.9885729897567223 0.9569127296905222 0.8795906743940991 0.9568270942028986 0.9768597857621067 0.9568953620399328 0.9719711729356779 0.9570649867374005 0.9868235106382979 0.9569008924705317 0.972030046958855 0.9568307024296366 0.9870295804676754 0.9569453379841233 0.972003707012059 0.9569749855421686 0.9998728574237954 0.9572263396955787 0.9999047978227061 0.9569165960519981 0.9886007191344931 0.9569029510961216 0.9885527132447492 0.9583869241285031 0.9870195039044558 0.988638221919054 0.9997578448786264 0.988638221919054 0.9919254042643023 0.9890810916648437 0.9998045212144803 0.9868891529515031 0.9918087257792755 0.9568663339731288 0.9887175787234043 0.9705469364292156 0.9720188710397145 0.9688446537997588 0.9995679176470589 0.9688307622677464 0.9694470599429116 0.9696305683163629 0.9886242732362823 0.9718557625248291 0.9874306612832824 0.9914038742905191 0.9873790635232483 0.9776680049261084 0.9570692495219885 0.999842120023534 0.9581681225879684 0.8828775186701999 0.9766412685375315 0.9581643390872547 0.9720659065251749 0.9886369086195722 0.9886881110136024 0.9915599383896324 0.9899712633376013 0.9767164233072316 0.9877757243439301 0.9583994444444445 0.988068486277873 0.9880646274425595 0.9702646089322149 0.9880532724930212 0.9880335407725322 0.987464225165563 0.9880837805400774 0.988090747963995 0.988101364902507 0.8776122135346455 0.9873737412891985 0.9583769584295613 0.9583449515011547 0.9874936851286524 0.9875501758957655 0.987507922842197 0.9586085220773279 0.9877718692634879 0.987464658227848 0.987474690733502 0.8837340950958428 0.9586692951945079 0.9583523101482326 0.9875277715030408 0.883940029615005 0.8840461623246494 0.8770198450223273 0.8845254051987768 0.9872779282608696 0.9872640479302832 0.988898824168659 0.9885928037383178 0.9694194062273713 0.949264391521197 0.9877372113715278 0.9875803205407762 0.9878949989118607 0.987874484861686 0.9873577656794424 0.987595209606987 0.9748872504169646 0.9742556926355671 0.974948935460824 0.9745528942353502 0.9747871326506311 0.9745107508369202 0.9748092133492251 0.9879469968833482 0.9877734390352836

GCA_002333865 0.8702253825857521 0.9569494921020656 0.9981556076989619 0.9992275156621301 0.9990264121292609 0.9585094135729487 0.876450906122449 0.9586817173396674 0.9586913898305085 0.9577709652981427 0.870035930713547 0.9586445737547894 0.8699265412558381 0.9996277813643211 0.9574652653874907 0.8765818547516199 0.9571058723926382 0.9990326791076792 0.9570364926275078 0.9990378613344925 0.9574023718420408 0.9997152105921601 0.9577300925474915 0.9996175925925926 0.9582432177121771 0.999289473464271 0.9578856148946595 0.9996085677694772 0.958649218186192 0.9992790820149875 0.9579403373852643 0.9991701119324182 0.9577604504504507 0.9591984136594818 0.9566482143727163 0.9994140250903678 0.9579657529527559 0.9997494844720497 0.9579216164584864 0.9993168722650553 0.9580116621521794 0.9992099709904682 0.95733140385566 0.9993047255717256 0.9578749914446345 0.9990778003348681 0.9578065510550571 0.9992055203150912 0.9578816352824581 0.999193185324161 0.9575207819621012 0.9997123207978393 0.9578060609786084 0.9996700562148657 0.9577715453881723 1.0 0.9579622944693573 0.999210571007164 0.9578470509977828 0.9997302100228739 0.8757104918032788 0.9997135985871599 0.9570224407525042 0.9991890229166667 0.9577559243800638 0.9993321758287871 0.9580724317492417 0.9991393759131707 0.9580297860304967 0.9991151227632127 0.9574632200488997 0.9993343223366254 0.9578830723781389 0.9993396145745577 0.9580672238658776 0.9997122196748646 0.9578536181640627 0.9993126713459951 0.9576062042720354 0.9993079020833334 0.9577989864029667 0.9989610291789187 0.9579393349814587 0.9579345400095831 0.9578652941176472 0.9579345400095831 0.9583439876543208 0.9579226476282672 0.9577288683377951 0.957462550548112 0.9571738047711783 0.9992357977621218 0.9578865358993338 0.9555614272144385 0.95801838296825 0.9569611116600791 0.957971154316459 0.9568989852579852 0.9572446625615763 0.9575339821083171 0.958034445534838 0.9575024452105393 0.9576072970996832 0.9579956373031496 0.9570681693198263 0.957729529092702 0.999376375452802 0.9580420107896028 0.9901142301458671 0.8721120944503735 0.9582138305252725 0.9984441927603821 0.9580228305631358 0.9574730355380059 0.9579315114541832 0.9571452113364282 0.9568649662487946 0.9584411119459054 0.9582466024390243 0.9897426318267419 0.9576145350813304 0.9575419650655022 0.9572461705237514 0.9575650800970873 0.9575665833535549 0.9571574200799201 0.9575573849878934 0.9575376029055691 0.9576318987648341 0.8765775683890579 0.9576650134179068 0.9996520998055737 0.9996486312607945 0.9576697485965341 0.9576751696363193 0.957707201756526 0.9996442157929084 0.9577681755424063 0.9574662996790916 0.9575086013813516 0.8762298542660308 0.9996523822894168 0.999661827027027 0.957734271062271 0.8801467892030849 0.8759015216805817 0.8760682087941731 0.8771807209429825 0.9589769907297361 0.9588843747016706 0.9577024597757192 0.9576118358531318 0.9570353066799107 0.9439302198926106 0.9576861199122593 0.9578214574701437 0.9577497047340167 0.9576248717948719 0.9578369658536586 0.9578226970954355 0.9582846000993542 0.9578366074074074 0.958285233366435 0.9583300421522439 0.9582748573554949 0.9583989514321296 0.9583125868917578 0.9579282057001239 0.957917171541894

GCA_015684135 0.877434157444668 0.9896978671406355 0.9592942970574496 0.9603968324849607 0.9582044115470023 0.9890510366122629 0.8849961987261147 0.9795057238605899 0.9903110549744049 0.98999816057774 0.8817178072460337 0.9899757934734019 0.8817786171726755 0.960271092047299 0.9716476777367773 0.884429375159398 0.9896749787234044 0.957854411182583 0.9899274884640739 0.9580931665421958 0.9774005130057803 0.9578332851189018 0.9893480556163783 0.957927917285785 0.9896994205150976 0.9580401754819574 0.9897438268892795 0.9578395448924067 0.9901720681318682 0.9578080904399114 0.9906086076223474 0.9579245011141372 0.9894439324116744 0.9893992757660166 0.9739643192594265 0.9579205603554677 0.9739835295459731 0.9578306462668299 0.9895204876434245 0.9577942524509806 0.97402992481203 0.9577941570552146 0.971533378476421 0.9577294409024032 0.9893198194627917 0.9576305630686011 0.9903221541118066 0.957777752394989 0.9799151548974944 0.9577232090284592 0.9896098079385404 0.9577981446078433 0.9740246783359855 0.9579941746382143 0.9894161302345978 0.9579170674846627 1.0 0.9577307927129494 0.9900535497088635 0.9578322647998035 0.8802487723512142 0.9577615563725489 0.979673912556054 0.9576747024246878 0.9739895552530067 0.9577048490056471 0.989999029535865 0.9577741079754601 0.9742299015439696 0.95779638548308 0.989654177927928 0.9578260494129158 0.9740427330895796 0.9578664752086402 0.9896732603406325 0.9578510673194615 0.9893531160537328 0.9577872498164913 0.990106275728988 0.9577637009803922 0.9900531178129747 0.9600767120075047 0.9900045028312572 0.9885425533890437 0.9894805137289637 0.9885425533890437 0.9900550579839429 0.9889846760500447 0.9894375323252247 0.9900130470793375 0.9900415958388668 0.9576602786604742 0.9901189233419466 0.9729684249252702 0.9739921365187714 0.9706879102316601 0.9894025413633357 0.9708794556840077 0.9714982522176935 0.9717066602824994 0.9908455803670131 0.974045340243353 0.9903742138364782 0.9900562979482606 0.9902519394594594 0.9797644844776797 0.957726352479136 0.989779569325423 0.9604634754024031 0.8838853801593048 0.9791982484298675 0.9594068409638555 0.9741397171763625 0.9897871593634183 0.9905116666666668 0.9897456300268096 0.9900162306034482 0.9791818343472158 0.9903788499893911 0.9603802397970949 0.9890755995717345 0.9890593716491529 0.9725385878829763 0.9891306176281365 0.9891067887746359 0.988182778508772 0.9890468092386655 0.9890512042780749 0.9890272540633019 0.877136400862069 0.9885583937145351 0.9592864501653283 0.9592722797070635 0.9887266287961546 0.9887733333333334 0.9887367962437215 0.9596386299765809 0.9902059646408841 0.9894633810623557 0.9894722606689736 0.8843778802425467 0.9596315827675017 0.9593138817480721 0.9887633623883199 0.8863918711122168 0.8842233383991895 0.8774397520443153 0.8848909214579055 0.9897899306157849 0.9897711730141459 0.9907756308158407 0.988459916782247 0.9715140088105726 0.9507311671288128 0.9889250010905126 0.9886544770240702 0.988993980752406 0.9890478900087643 0.9885708320594018 0.9886578785886478 0.9774074694464414 0.9773461358201817 0.9775238835649258 0.977540921714149 0.977487648467433 0.9772668404537775 0.9774651646238885 0.989325925515407 0.9892007239919803

GCA_002333905 0.8705316086842794 0.9571777820636453 0.99764917380137 0.9990376457929848 0.9990005921052633 0.9577449811142588 0.876929710888949 0.95789538882283 0.958147166988417 0.9573831295664882 0.8709924514314927 0.9587939478054567 0.8707260015368853 0.9991115290322582 0.9576649083250744 0.8759934369189907 0.957261419198056 0.9993961775025799 0.9572917146282974 0.9993981595989136 0.9574693399902582 0.9993913686396678 0.9579097968069666 0.9993315287428932 0.958388967616265 0.9994436318715256 0.9580624914758891 0.9989130659086125 0.9587635380952381 0.9992354681724848 0.9582110244384101 0.9993644139344263 0.9579698161586839 0.9591032395382395 0.9568019257821975 0.9989909217758985 0.9580474834922964 0.9993834628099174 0.9580601459854015 0.9996325390468562 0.9581609904622157 0.9994156597222222 0.9578571807992202 0.999572904647436 0.9579681990291263 0.9994050151118274 0.9572980552884616 0.9993830944091747 0.9575968431420832 0.9996026007619812 0.9572278701050622 0.9995012761983815 0.9580324120234605 0.9994721434511434 0.9580136565900848 0.999204533968517 0.9578069306930693 1.0 0.9575382056946491 0.9990935042735042 0.875866424 0.9991751197667152 0.9569385063045589 0.999457574970012 0.9579901803998051 0.9996490261569416 0.9578736763590392 0.9987694491789649 0.9580909269249632 0.9988111879543096 0.9571439815040157 0.9994998782501031 0.9580973480392158 0.9995100435413643 0.9581871981386236 0.9993515742285238 0.9579892052338259 0.9993527231960865 0.9572872582619338 0.9996595733173078 0.9574665539372994 0.9988676152802894 0.957674191248771 0.95777344293543 0.9579846974133723 0.95777344293543 0.9583294331785976 0.9577237608069165 0.9579030507246377 0.9574037854737856 0.9573178194945848 0.9996105321905522 0.9575694738136218 0.9557726898509582 0.9580990747070313 0.9568060401862291 0.9581037943607195 0.9561447017287558 0.957805858561395 0.9571969293413173 0.9573920506575742 0.957585583108274 0.957498288396726 0.9581442515262515 0.9568272179914327 0.9577452351788338 0.9986054975702514 0.9581634565799075 0.9897959977011495 0.8737853420365538 0.9582533957153411 0.9968507410674902 0.9582063494809688 0.9571535400833538 0.9581285133795838 0.957334827669903 0.9566574765681327 0.9583386947890817 0.9581632177497573 0.9893750989983694 0.9572633985507247 0.9573159792320697 0.9575210021786492 0.9572870593915981 0.9572606998069499 0.9568616463868885 0.9572831752527684 0.9572855320173327 0.957352766674693 0.8768596611095929 0.9574362542537677 0.9993500776196637 0.9993270400689358 0.9574437484796886 0.9574616387065403 0.957472021871203 0.9993481657696448 0.9576481544117647 0.9577135705491259 0.9577467634752647 0.876736180811808 0.9993276374218582 0.9993290987494611 0.9575192603406325 0.8801404252610133 0.8763468210862619 0.8763151191106405 0.8774031138211383 0.958923976442874 0.958854950471698 0.957254331148337 0.9576072703474537 0.9569933349790175 0.9442561471861471 0.9576882524271845 0.9574944795719844 0.9577492601606231 0.9576357887049659 0.9575631216545013 0.9575469202633504 0.9575389807502468 0.9576998469891411 0.958078935483871 0.9581173630731104 0.9574737299432239 0.9582506040268456 0.9580910329452563 0.9579925314581792 0.9579906071076012

GCA_015684155 0.8781288173359453 0.9884860618996798 0.958597091285353 0.9592765580768366 0.9569910876937985 0.9885454955541098 0.8860535363114549 0.9784813747564408 0.9883459810822701 0.9996229868877748 0.8810348175350012 0.9886624349881796 0.8809965869914962 0.9590030421076335 0.9691336135657989 0.8846671529352481 0.9884680622317598 0.9562441180740383 0.9888001645569621 0.9565166739606126 0.9768550486111112 0.9569938797682279 0.988666668761785 0.9569910160818712 0.9893830049579652 0.9563608486460349 0.9887549790268457 0.956794213592233 0.9887334813874789 0.956438524590164 0.9901283803706825 0.9563266188972552 0.988727127860592 0.988355506818182 0.9728250194805195 0.9570196594502555 0.9722866688756351 0.9569928554187788 0.9887071269206483 0.9563383229216723 0.972300919133893 0.9563397161414483 0.9700287364450729 0.9562949494949495 0.9886513038906415 0.9562011038804531 0.9900046608223754 0.95631707840772 0.9787878726198294 0.9561633044524669 0.999861297534875 0.9569988341781317 0.9723024173644779 0.9571489441894176 0.9886718575916231 0.9565817816091955 0.9894183965076659 0.9562473558504222 1.0 0.9566529997600192 0.8794355854430381 0.9566106807286674 0.9782970973412438 0.9561334118778553 0.9722967055714601 0.9563029172679173 0.9877894850382742 0.9567113164193394 0.9722120881772318 0.9566823025370992 0.9875509553853088 0.9563663949013949 0.9721032392502756 0.9563633453757225 0.9886926136603934 0.9570267471042471 0.9886664057239059 0.9563374747474747 0.9999268268497331 0.9562240413759924 0.9999184365952519 0.9579916598824062 0.9877014081632653 0.9873141376228775 0.9887410325856962 0.9873141376228775 0.9892341700753499 0.9878677978106889 0.9887208885163453 0.988547269984917 0.9887859927797834 0.9561235923261391 0.9997917669678908 0.9714221306376363 0.9723422433208215 0.9699001391220917 0.9885816237874313 0.9697148444549987 0.9699747448501873 0.9703663843266074 0.9892995950087565 0.9726551776868404 0.9893221396250809 0.9890336970857265 0.9892603119621342 0.9785920126196692 0.9567524321076664 0.9887318240837697 0.9590957832136445 0.8845765370196814 0.9777945629991126 0.958276090102708 0.9722078652434957 0.9892573202199663 0.9885390141752578 0.9885760796062486 0.9885303470490441 0.9777277137753962 0.9892741940857975 0.9589629178082192 0.9989185671936759 0.9989161471518986 0.9715080374464247 0.9989177171117706 0.9989628351126928 0.9981920325203253 0.9989017015396763 0.9989100532964865 0.9989037828232973 0.8777438860521366 0.9986593349215803 0.9581184911717496 0.9581276231950493 0.9987754124428544 0.9987928879651624 0.9987814640444975 0.9584446842344388 0.9880491397139715 0.9887308489079036 0.9887358370837085 0.8846044284651624 0.9584683534227882 0.9585025711035268 0.9987439591593973 0.8856804653996102 0.8840616286644952 0.8775323705438458 0.8846768928121059 0.9883937585324232 0.9883463187165776 0.989481275368511 0.9866432050134288 0.9696852206235012 0.9494949595489091 0.9988382186635035 0.9988258894611728 0.9988770027733758 0.9989170993272656 0.9987997087378641 0.9988190806419656 0.9769165766406065 0.9769664520710059 0.976980222643297 0.9770078322672352 0.9769068892045454 0.9768021729807005 0.9768946639753027 0.988181752033414 0.9880808541160891

GCA_002333925 0.8704668836601307 0.957067189384801 0.998005943456843 0.9991681515671963 0.999098349429324 0.9582887296340025 0.8770543311758361 0.9584686797819388 0.9588399758395748 0.957763794274624 0.8706665863041806 0.9586775510204081 0.8706247131463855 0.999588483097989 0.9577078011377691 0.8769500428724546 0.9571193575079096 0.9991380166346769 0.9572387194244604 0.9991289704295274 0.957753478684531 0.9996396193628465 0.9579452160270338 0.9994431057591624 0.9582281015129331 0.9992365689332502 0.9581960758570388 0.9994572636921126 0.9586971976190476 0.9993341654571843 0.9583087592501234 0.9990390304302205 0.9580240864108135 0.9592092239093758 0.9568770601066408 0.9993720639966095 0.9582469901960785 0.9996661402061855 0.9581040160155303 0.9993201159660386 0.9583262099339369 0.9993214162028449 0.9579890528905288 0.9993415475450591 0.9580584399224807 0.9991970916666668 0.9580464419836302 0.9993282762180016 0.9578821943419433 0.9992474823578249 0.9574660747441087 0.9996623413928498 0.9581380987292277 0.9995783688677292 0.9580137509051413 0.9996514520098441 0.9581817294350842 0.9992409763746604 0.9578320922851563 1.0 0.8758767993630574 0.9996536843189779 0.9574032086470732 0.9992412067178105 0.958126578818936 0.9993216256773656 0.958319937075258 0.9991143774683019 0.9580300390434358 0.9990972731048805 0.9576026347741623 0.9993705561363162 0.9581232037172903 0.9993960452925142 0.9583003033268103 0.9996731379883624 0.9580235331077817 0.999357832613613 0.9576519907520077 0.9993176344086022 0.9578268501228503 0.9987953337813621 0.9581544788593902 0.9580014894123245 0.9580977788602045 0.9580014894123245 0.958412037671233 0.9581132347282347 0.9579689823969133 0.9577751044196212 0.9572345121657433 0.9993313365007231 0.9579481321909424 0.9560053292423887 0.9582537979946197 0.9570464752086402 0.9581239226118501 0.9574513150818871 0.9579396446949277 0.9577851140456183 0.9578735759493673 0.9577476163645272 0.9581580863448946 0.9583042218421696 0.957416358187485 0.9578197531165974 0.9992601060670344 0.9582245593590678 0.9902231159253284 0.8727527157561362 0.9582159380378659 0.9985088323750553 0.9582746188008882 0.9575455172413793 0.9581228161346202 0.9571696360106771 0.9569922573687994 0.9583452095214482 0.9584849005337216 0.9899388483146068 0.9576568625090296 0.9576303948001926 0.9577824837074583 0.9576272105896511 0.9576335275704311 0.9571102777777778 0.9576082832411639 0.9576104208754209 0.9576745393312486 0.8769633698030634 0.9575388457807954 0.9996070748445206 0.9995996958011997 0.9575657533914728 0.9575809864275326 0.9575797529668201 0.9995736410698879 0.9579736059936136 0.9576120284872299 0.9576506773006135 0.8768202465897167 0.9996056015440702 0.9995985536480686 0.9576032226799127 0.8803373944020356 0.8764734282672345 0.8764228537170263 0.8773403466955579 0.9591632485136742 0.9591342802669209 0.9579247996113676 0.9574821957608954 0.9570411300271403 0.9443431628498727 0.957774715840387 0.9577277750906892 0.9578668580426357 0.9577120276497696 0.9577386045949214 0.9577427061105723 0.9583747639060567 0.9579592090395481 0.9583768543209876 0.9583996395951617 0.9583692273512714 0.9584367252910576 0.9583659111111112 0.9579574686346863 0.9579579434194342

GCA_015684175 0.8870321012465023 0.8812131565462465 0.8768748455696204 0.8763374639546859 0.8759978972207657 0.8803740246090745 0.9685456493202064 0.8807639708514446 0.8798802507257851 0.8804397776053788 0.8872177192118227 0.8795010072127768 0.8872398938795657 0.8765001363870303 0.8783775137398587 0.9681574687720954 0.8811339099099098 0.8759910811518324 0.8814305117107943 0.8762834402945819 0.8805949046391751 0.8760563734567901 0.8802818892339545 0.8762346822308689 0.8805772936259143 0.8759168688693856 0.8804801270417424 0.875915587243972 0.8795817429082545 0.875923295542386 0.8799140031397173 0.8763194260599794 0.8803780393680394 0.8763639008792966 0.8804012164896307 0.8760389574579833 0.8804704346714395 0.875865732647815 0.8806584401764859 0.876104532208589 0.8805824802043423 0.8759512811296535 0.8801886974464793 0.8761244242579325 0.8804526687435099 0.8762258017418033 0.8818668661257607 0.8757782407407407 0.8812278363776692 0.8761458459175837 0.8779350745118191 0.8759537349088108 0.8802871895758814 0.8761479454872718 0.8803235179911986 0.8759377926421406 0.8805825196437925 0.8762706916945231 0.880407854725332 0.8762731140801645 1.0 0.8759877202472953 0.8798887029930927 0.8761177379733879 0.8802640521205928 0.8761721613316261 0.8813685087014728 0.8755397625260959 0.87941125 0.8755735349322211 0.8813653932584269 0.875912286007176 0.8803530502821961 0.875917554585153 0.8805324895724713 0.8759634506680369 0.8805233047767393 0.875774071227261 0.8800584017671519 0.8761269480685597 0.880399582681273 0.8776787229041526 0.8801962473903966 0.8781187176808567 0.880746015115976 0.8781187176808567 0.8807891519709544 0.8777917522262966 0.8803524967658473 0.8790353169198534 0.8812374629535001 0.8760591749680715 0.8803276298026999 0.8760900077539415 0.8803985425722322 0.8789770376216786 0.8800986807181889 0.8784589238020424 0.88012609431061 0.8791719596320899 0.8804716113129217 0.879780700538876 0.8795288476613538 0.8803054460822344 0.8801508539731683 0.8811911479591839 0.8762717817429533 0.8807528456549935 0.8766470579190159 0.9078308170254404 0.8789970615989516 0.8767986294549267 0.8806200879461976 0.8799587864583334 0.8808415944676409 0.8814506124549667 0.8798797310327329 0.8790228016899923 0.8802839611901682 0.8777776252609606 0.8790098020317791 0.8790216432291666 0.8769150013017445 0.8790500338541667 0.8789747497393117 0.8802419781099841 0.8791529274004686 0.8791596826222684 0.8791596746486205 0.9688931387035271 0.8795332301690507 0.8759460810810811 0.8760539735271218 0.8794967342166797 0.8794137025481019 0.8794975863860743 0.8757080232858989 0.8798642968953821 0.8782737891662241 0.8783354340323866 0.9652254844649022 0.875608168868169 0.8755436278828711 0.8791511307972905 0.9286812137263933 0.965627772823281 0.9327082355840315 0.9297376278724981 0.8804416234266633 0.8802834576794445 0.8808513873130478 0.8761856856100105 0.876643287307488 0.8796964780042917 0.8795451898075922 0.8798590271257173 0.8795589213131839 0.8792299999999998 0.879849386903209 0.8798537901331246 0.8792767657992565 0.8791735602649006 0.879427412922095 0.8794353625498008 0.8793025690021231 0.8791911933937133 0.879107728237792 0.8782746151825584 0.8782787470449174

GCA_002333945 0.8699010150276826 0.956916358774915 0.9980839027027029 0.9992292704048495 0.9991726626693316 0.958250404376784 0.8763526970275429 0.9585349165076338 0.9584328950572194 0.9576451029916625 0.8702441884545691 0.9583518764959311 0.8701884150453956 0.9996217496229263 0.9576833109118087 0.8762561247637052 0.9568500660954713 0.9991164250376425 0.9570073401162791 0.9990272079337403 0.9574702427065457 0.9996824489795918 0.9575533893762183 0.9995013615221987 0.9579184643734643 0.9993723922143157 0.9577893550760176 0.9994642446196462 0.9581939798125452 0.999279686716792 0.9582325584286424 0.9991711360270784 0.9576250780107265 0.9592425200097017 0.95669299026764 0.9994007366167023 0.9582322512315271 0.9997053961012029 0.957703428011753 0.9993687377425413 0.9584200073946265 0.9992705042539946 0.9575176052501239 0.9993284528773978 0.9576073096695227 0.9990100566037736 0.9578956657773466 0.9992056641030971 0.9578075994035787 0.9991851846442729 0.9573096681096681 0.9997261255998331 0.9580948917322836 0.9996121942071265 0.9575932244699001 0.9996307136963697 0.9581264225000001 0.9992403367003369 0.9575842990884454 0.9996182392165034 0.8758573463911995 1.0 0.9571220635696821 0.9993123967459324 0.958112322612325 0.9992740339836376 0.9582874657534246 0.9991529623430964 0.9579069953282519 0.9991160300877558 0.9576284031349499 0.9993208305298571 0.9581214905149051 0.9993273639814423 0.957845688164072 0.999714809623431 0.9576610021999512 0.9993043111663559 0.9574185882931628 0.9992957044601919 0.9576249221260815 0.9987634011299436 0.9580955734456279 0.9578030297933687 0.9577463047759724 0.9578030297933687 0.9579620887792848 0.9577686282113428 0.9575358321167883 0.9572350734573948 0.957000580943121 0.99932148409894 0.957786121691813 0.9559645971000713 0.9582277564575645 0.956991112759644 0.9578038147605084 0.9571590676506766 0.9574559793307088 0.9577256808305167 0.9578872290338402 0.9575846984986464 0.9575104372252077 0.9576016407982263 0.9569304929917835 0.9576828061977374 0.999420790149893 0.9578620985535669 0.9900689921223355 0.8721657520000001 0.958207729528536 0.9983146723138654 0.9582884016902808 0.9574322540781017 0.9580932693266833 0.957008096521313 0.9567230758087881 0.9583246344516774 0.9581850379065786 0.9898878133836003 0.9574032385120351 0.957375701434476 0.9576575778967867 0.9573734014101629 0.9573829054218334 0.9568870463078848 0.9574214011656145 0.9574177829043227 0.9574377556473159 0.8764174951617362 0.9573750832109642 0.9996050551351351 0.9996141434435083 0.9573587062851553 0.9573634010276487 0.9573684303178485 0.9996153918154115 0.9578843771626298 0.9575671198213843 0.9575920367154552 0.8764582455676104 0.9996170307093426 0.9996150779220778 0.9573803864025434 0.8800626004119465 0.8760666765498653 0.8757069524067064 0.8766739550807996 0.9589509345794391 0.9588843334134038 0.9576785406464251 0.9573879927797835 0.9570188670807452 0.9441216765083442 0.9576196994869289 0.9574546811629611 0.9576907886358069 0.9575536762184668 0.9574350427350429 0.9574406529713866 0.9583646673311738 0.9580229630547978 0.9583706706556968 0.95840843874502 0.9583228674638764 0.9585881940970485 0.9583589962640099 0.9580787698807157 0.9580689982600048

GCA_015684195 0.8779183701863353 0.9777078945015646 0.9589711131768535 0.9597573473150496 0.9580259708498025 0.9774399412694829 0.8850980483460561 0.9989883923029175 0.9776236176606565 0.9788611678832118 0.8810863894139886 0.9782697882513662 0.8810480397727274 0.9597487118408882 0.9716948934624697 0.8851582427628237 0.9778563749154071 0.9573607067224821 0.9782205211798625 0.9577576812313804 0.9983129329102448 0.9572544420131291 0.9780053316842816 0.9573631491034144 0.9783970150616156 0.9573095600097776 0.9780332950967161 0.957326745271432 0.9787627767857143 0.9574078801169591 0.979359931880109 0.9570080713761917 0.9780276532912534 0.9774691573033708 0.9727653908830655 0.9578899258710156 0.9741363800000001 0.9573200510080156 0.9780541611953816 0.9566282002412544 0.9741458433200176 0.9572614518086914 0.9720097418896521 0.9566746819830715 0.9779914498644985 0.9567084924866699 0.9784428902765389 0.9571309108574205 0.9981330113030498 0.9566156720755632 0.9786415131286161 0.9572537900874636 0.9740848134162594 0.9574709953760039 0.9780290974566733 0.9570121928761813 0.9793726802259886 0.956870948568656 0.9788031826320501 0.9571548406713695 0.8795677330173776 0.9570919101941749 1.0 0.9565973666425296 0.9740378565101862 0.9567769752787203 0.9776682501843205 0.9576864569049951 0.9738729058693244 0.957636464399315 0.9770560748763834 0.9571483797529667 0.9740044330813696 0.9571396484848486 0.9780764329475834 0.9573152282661488 0.9780230275643923 0.9572013541666666 0.9787755092489414 0.956612753868472 0.9787645046264952 0.9595926844070962 0.9777500095533797 0.9762275825753949 0.978042089009991 0.9762275825753949 0.9785417915904936 0.9772045239733883 0.9780260085354897 0.9780889731940028 0.9781175173184359 0.956549491566265 0.9789098991709613 0.9725681616982838 0.9740827276765882 0.971657294032375 0.9778290072365445 0.9710338367346938 0.9719020028241938 0.9710685868544602 0.9784415159090909 0.9736047173575704 0.9780678130671507 0.9782039972714871 0.9775402628032346 0.9982418475506758 0.9575860947471773 0.9781259150179856 0.9596663200540176 0.8848904377431905 0.9980371824880798 0.9589335434207361 0.9741829921259842 0.9789577457817772 0.9793035246272029 0.9779249999999999 0.9778405892700088 0.9979868680236375 0.9797031476997579 0.9599119675174014 0.978715831287659 0.978684312849162 0.9728508873175183 0.9786837991071429 0.9787538602366599 0.9782617971611721 0.9786818720748829 0.978684740361043 0.9786776749888542 0.8783988924731183 0.9774564363636362 0.9591031790413534 0.959119586174465 0.9774260217983651 0.9775422568973315 0.9774599727520437 0.9593430067646372 0.9780434325946447 0.9773621190130624 0.9774042408123791 0.8851507178217822 0.9594064909006066 0.9592494456091312 0.9774776086956521 0.8860263752143033 0.8847532898696088 0.8783072380454664 0.8847207451984634 0.9784171770551039 0.9783491713832919 0.9801657183718372 0.9763188949880668 0.9711884234669242 0.9515547784730914 0.9776744128274616 0.9775063186563776 0.9777619253393663 0.9777122560145256 0.9775008882326215 0.9775896516393442 0.9857382976722314 0.9857666878232252 0.9857976333725028 0.9857989236192714 0.9857624423529412 0.9856406024667932 0.985758355325165 0.9780971685496888 0.9780263823393435

GCA_002333965 0.8707399189330544 0.957205257184255 0.9977342847924285 0.9990540198618308 0.9987674239753196 0.957907247706422 0.8772655750539957 0.9580098412698413 0.9584557225433525 0.957702768705825 0.8710525813893872 0.9588859427890346 0.870764294163024 0.9991173101811908 0.9578310514541388 0.8763015168390348 0.9572624646513894 0.9994279158897866 0.9573111931681502 0.9992834397312055 0.9574240078201368 0.9994236306924518 0.9581494949004372 0.9992360151483273 0.9582750219833903 0.9994888219517226 0.9583944610119776 0.9988564750633983 0.9587979122722914 0.9992544709266489 0.9583352533992583 0.9995044979423868 0.9581957174071377 0.9591999323834823 0.9565619597380548 0.9990012449714165 0.9580699042474834 0.999430041347943 0.9582407545787547 0.9996169857515552 0.9581421651511428 0.9993625772773799 0.957854063414634 0.9996141276510605 0.9581900901120313 0.999443191532258 0.9573270291075295 0.9992498464373465 0.957396295656466 0.9996289581246243 0.9573329526994745 0.9994680245118405 0.9579873406862744 0.9994810524126456 0.9582100970638194 0.9991480755657048 0.9581462261343913 0.9994795627415092 0.9576135652815343 0.999051921790046 0.8761294274300931 0.9991836551724137 0.9568801044196212 1.0 0.9579341650342802 0.9995938156831042 0.958063877344146 0.9988958811305071 0.9579304753736829 0.9988074568875962 0.957510829885617 0.9994582590673575 0.9580849163797345 0.9994772748075724 0.9584742952029519 0.9994196805178536 0.9581749671772429 0.999413893985729 0.9576203799019608 0.9997169641785072 0.9575166650173182 0.998713182230281 0.9580336491141732 0.9580097586042066 0.9582458850855747 0.9580097586042066 0.9582868377117604 0.958078647866956 0.9581658531265148 0.9574797210867998 0.9572890123158658 0.9996830569948186 0.9577469741060419 0.9552358124111795 0.9580841290480863 0.9570555651105651 0.9583347368421054 0.9562687411563797 0.9577749078117419 0.9575296032700168 0.9574585749145926 0.9574611119311193 0.9576349036608864 0.9582215361963191 0.9569319267364417 0.9575655776306107 0.9987454447496302 0.9584529558213326 0.9898362099930923 0.8744606258148632 0.9581093973820697 0.9972893801195484 0.9581565577018327 0.9570028382352943 0.958446776921164 0.9573191402825134 0.9568878360182735 0.9581492242665339 0.9583410276967931 0.9892091255552958 0.9575296375060417 0.9574960797101448 0.9577423736637511 0.9575250012074378 0.9575167093500845 0.956983403831799 0.9575601567398119 0.9575609693754521 0.9575685721177039 0.8770996428571428 0.9577302945472249 0.9992873692174665 0.9992912337942955 0.9577261961547823 0.9577304818690678 0.9577433787399661 0.9992973957207695 0.9578773962171456 0.9577807472256474 0.9578033004683262 0.8771300738396623 0.9992907048648648 0.9992923113513514 0.957803825896123 0.8803427166156983 0.8768652025586354 0.8767549429859454 0.8781736358710376 0.9594390143158884 0.9593370782280866 0.9573347466666666 0.9578073459149548 0.9569538362602028 0.9444245245398772 0.9579588272506082 0.9576901462344626 0.9580615142648136 0.9579013933626159 0.957749025103583 0.9577370539419087 0.9576516637867194 0.9578597928994083 0.9582951872055543 0.9583119722497523 0.9576303603158935 0.9585281200199204 0.9582143494423793 0.9581844285361639 0.9581800419649469

GCA_015684275 0.8774661767578126 0.9728646703662597 0.9591946326767092 0.9598519546998867 0.9578984513599608 0.9714580325613392 0.8860195518987343 0.9741919072615924 0.9716665766814058 0.9731129082082964 0.8808955265588915 0.9726830796938316 0.8809993723946271 0.9595545698070376 0.9917713148945818 0.8850490632976008 0.9728456905829596 0.9576594883040936 0.9730163866285463 0.9577619621342514 0.9717938371274351 0.9577599099756691 0.9724926048369205 0.9579267825443788 0.9730567292467768 0.9576668426197459 0.9724233888518766 0.9577257638376384 0.9727273967763305 0.9576310401948844 0.9740932602306128 0.9576032401960783 0.9725026306426507 0.9706609406303445 0.9909181519861829 0.9578145726181729 0.9999978961748635 0.9577863698296837 0.9726414237743453 0.9575344027138357 0.9999583595724003 0.9575983786407767 0.9982071897258797 0.9575045747516358 0.9726063238902523 0.9573854889589906 0.9736835425508154 0.957510605839416 0.973927897891232 0.957552722640136 0.9727461209964413 0.9578320380302293 0.9999780112643233 0.9580102487198245 0.9724955612358303 0.9573937765957448 0.9736481768459436 0.9577606019985377 0.9730246102300649 0.9574460949612404 0.879661746198217 0.9573077187046883 0.9737147793468667 0.9575079937000243 1.0 0.9575459907722196 0.9712436876832845 0.9574530785973399 0.9912360965576966 0.9573330379135474 0.9713678810320114 0.957541074199806 0.9999003689244584 0.9574952221412965 0.9726766053811658 0.9578883710737763 0.9726027244375139 0.9575735315533982 0.9730557959092931 0.9574690186576207 0.9729260998650472 0.9591556205551731 0.9714408231634363 0.9704799713124552 0.9726201612180922 0.9704799713124552 0.9730308361505522 0.9717893204775022 0.9724799178507992 0.9720906612133605 0.9732237511091393 0.9574917448042533 0.9729181481481481 0.9915802442159384 0.9999990465071027 0.990654099838225 0.9723750380824373 0.991280454861917 0.998310999144202 0.9908155969649631 0.9723505802526907 0.9908387993064587 0.9726666780587835 0.9723524137158225 0.9726256726610517 0.9742527677390922 0.9572360125664572 0.9726265131286159 0.9590388658865887 0.8847448074594333 0.9731415208522212 0.9587809526055174 0.9999060114016121 0.972780133273097 0.9736260650147762 0.9728655856057219 0.9730027997335703 0.9731520169608068 0.9741042561163765 0.9599717573800739 0.9723286281993713 0.9723453780569891 0.991668793177893 0.9723453095558546 0.9723629216258702 0.9715295530338119 0.9722561331540014 0.9722416364043936 0.9722785624579504 0.8782206890029721 0.9711346909667196 0.95861532839963 0.9586083791907514 0.971133885740195 0.9712779237192508 0.971181629646419 0.9589016624685138 0.9721805098222638 0.9705463313180169 0.9705872613004591 0.8854587182044888 0.9588735290071085 0.9585844075937786 0.9711688929055581 0.8850870701107012 0.8841955310067787 0.8784221188903567 0.8847581218144752 0.972806991449145 0.9726839841986457 0.9740627051945131 0.9702143429525398 0.9902776291578202 0.9486087721456694 0.9715321599638499 0.9713564217687076 0.9715824937768726 0.9716019146036793 0.9712048708068903 0.9713549670529424 0.9714208349191248 0.9714061163895487 0.9714514384213029 0.9714771309721892 0.9715162339514979 0.9710941168022977 0.9713983329364134 0.9720754079740381 0.9719277504066931

GCA_002333985 0.8706022667013257 0.9569274277456649 0.9978927105206772 0.9989031081953109 0.9990998925674195 0.9577212682010334 0.8770291368647487 0.9579001369863014 0.9581182136015325 0.957069745207474 0.8706970408940817 0.9583629181494662 0.870567484725051 0.9988953075268817 0.9576393703703704 0.8762454543053355 0.9569045353069643 0.9994432610491534 0.9569240824149496 0.9994440676550429 0.9570921232710506 0.9996365421336654 0.9577858076181291 0.9994994696969698 0.9579778689722358 0.9996993797650937 0.9580511321671525 0.9991465642105264 0.9581884007615422 0.9995081413021154 0.9578089226993864 0.9994235734752355 0.9578453112934363 0.9587571383950025 0.9563826211719315 0.9991181545608109 0.9577988586030663 0.9995736547594467 0.9578720906007753 0.9996966133546582 0.9578096982234121 0.999623759967287 0.9572772033898307 0.9997009686438986 0.9578732317368166 0.9993375925181013 0.95714090234562 0.99956541624054 0.9572923593020398 0.9996083848454635 0.9568885373987612 0.9995350539419086 0.9577971011673151 0.9995538252386882 0.9578715223160434 0.9991593443640127 0.9574935297017502 0.9995876024340771 0.9571942019066243 0.9991789357262103 0.8758578138185655 0.9991445577523412 0.9567161199806483 0.9995758564307079 0.9577369759533642 1.0 0.957601031446541 0.9990602926525529 0.95735048269137 0.9990011819692564 0.9570912020398252 0.9997341601158461 0.9577296851354652 0.9996560675787728 0.9582220499632622 0.9996321540062435 0.9578691702951138 0.9996397194551739 0.9570192305813386 0.9997316118092958 0.9572050147565174 0.9987524571686204 0.957517420936504 0.9574653044719316 0.9579593291200778 0.9574653044719316 0.9580167967797024 0.9574432484382509 0.957785886319846 0.9572561293413173 0.9569486054339986 0.999692359729407 0.9573291907797941 0.9550789121732988 0.957796975443715 0.9568022806159863 0.9580017524776407 0.9553976513317192 0.9572846650602408 0.957330983488873 0.9572620014556041 0.9572022759462759 0.957457900909526 0.9575610355750488 0.9566858238636364 0.95746265788832 0.998865933277027 0.9581413883495146 0.9900240390804598 0.8733593698132781 0.9577186342422008 0.9967027028218695 0.9577244874938695 0.9566719804878049 0.9579471918992344 0.956927390356191 0.9565567718794836 0.9578035149863761 0.9580138892917573 0.9896376599719757 0.9569674296028882 0.9570045594607608 0.9570883035282747 0.95697948026949 0.9569526973051009 0.9564008735461519 0.9570054779058598 0.9570081988472623 0.9570128417006966 0.8769659912972533 0.9571981165048543 0.9991879530273649 0.9992018591124516 0.9572249709020367 0.9572213398058252 0.9572383709090909 0.9992274197716993 0.9575178011238701 0.9569763115356356 0.95699680382072 0.876674151633987 0.9992039142610945 0.9991502372223421 0.9572452122241087 0.8800673969137364 0.8763294284202171 0.8761307874637873 0.8776254401502951 0.9588457012622721 0.958771832239925 0.9572180357574294 0.9574870993093594 0.9569193405511811 0.9441157186389031 0.957330491644466 0.9571213313967561 0.9573969626394954 0.9573021019417476 0.9571122747093024 0.9571207503642544 0.9572192080668961 0.9573929845246869 0.9577281279328229 0.9577827035027134 0.9571883546483031 0.9579003839484765 0.957764337527757 0.9574031286836936 0.9573930712530713

GCA_015684295 0.8720392608921164 0.9883761982719418 0.9574547856973714 0.9585332073228722 0.9586197030651341 0.9890844895229605 0.8789811723052715 0.9778732364475201 0.9900019685039371 0.9890349800708592 0.8732534038366946 0.9901485881032548 0.8732152131066766 0.958318035204567 0.9723734767194516 0.8790896835774059 0.9884334480401095 0.9579444468494468 0.9886116670421267 0.9578447686746989 0.9771817848130304 0.9581958417266188 0.9881713504388927 0.9581858827785817 0.9893484065187866 0.9577947803121248 0.9886277166779814 0.9579027268339769 0.9889341877256318 0.9578817959770116 0.9910176021675322 0.9576867387647198 0.988262358108108 0.9905404995479203 0.9713616655100624 0.9583788289156628 0.9714847096018736 0.9580968465227818 0.9883123179255919 0.9578185564304462 0.9714997891777933 0.9578770737547894 0.9716288203437721 0.9577867974168861 0.988192596934175 0.9579023069544365 0.9903585979013174 0.9577806997364008 0.9780682399999999 0.9578363103778097 0.9882988638373121 0.9581721366906475 0.9714769672131149 0.9582524688099808 0.9881656300630063 0.9579904481525627 0.9897094887752834 0.9578381031175061 0.9886921439714732 0.9580741963219488 0.8805426386333772 0.9579729949964261 0.9777504675081814 0.9578382787473104 0.9714524684136642 0.9578695175543349 1.0 0.958181435395271 0.9720619461988306 0.9580566221374046 0.9994964925220242 0.9578953873744621 0.9713919821721793 0.9579044484326393 0.9885105850340137 0.9582152721169983 0.9882032281334534 0.9579126213824445 0.9887051427933959 0.958028360462373 0.9886710964716392 0.9583418201284798 0.9993536469625691 0.989021106929378 0.988248991387126 0.989021106929378 0.9898172840909091 0.989014715666442 0.9882596352994146 0.9896903617399022 0.9884842899140659 0.9578259852099237 0.9886935292807837 0.9707254554012709 0.9713835320243105 0.9717084467631018 0.9880656380090497 0.9704556906592126 0.9715600444444445 0.9716199859616285 0.9906546944757609 0.9713224589405506 0.9901672271517303 0.9895422003577818 0.9900820199335548 0.9779364580901238 0.9574100164396431 0.9886020790960451 0.9573675578947368 0.8758095744138634 0.9774279797026197 0.9582201586526727 0.9716187023443051 0.9890993508225878 0.9895126438848921 0.9884564927073839 0.9891549768671515 0.9774535571903181 0.9888548195932648 0.9577489985795454 0.9879230965028774 0.9879276759628154 0.9704936566988461 0.9879446848042467 0.9880653823985813 0.9869901772324472 0.9878292879256967 0.9877971848739496 0.9878147004200752 0.878079132395861 0.9882477863948594 0.9581282037213741 0.9581304886769964 0.9881759309276068 0.988181161090184 0.9882042689410723 0.9579585513796386 0.9992550772200773 0.988378463271744 0.9883907113912652 0.8785286246719161 0.9579703 0.9571306158218853 0.9881550709219857 0.8806270558115719 0.8791689771547249 0.8791295103092783 0.8799586096673597 0.9906454037952338 0.9905949590979438 0.9913137297297299 0.9890116844681799 0.9716617341317365 0.9503876599902296 0.9884267569367369 0.9886610384700911 0.9885249810816826 0.9883928947368422 0.9883516685156424 0.9887240636829213 0.9769831743032593 0.976411384253819 0.977121133947555 0.9772892642536078 0.9770457227208312 0.9765892356990269 0.9769638663202647 0.9895435609314944 0.9894957078245138

GCA_002334065 0.8697981621906267 0.9577547067520946 0.9990357374890256 0.9993778384232185 0.9991186602977117 0.9591072968451243 0.8761045286103543 0.9593327683480756 0.9590881457274268 0.9579466421568626 0.8703522378877212 0.9585765008371202 0.8703188021638332 0.9994092989646247 0.9578146897582854 0.8760929592662531 0.9577314470412729 0.9993296049540892 0.9577526515151515 0.9991912356880537 0.9578976492353232 0.9997645327399873 0.957698885381253 0.9996811317928969 0.9581769832677165 0.9994196366708916 0.9579503574926542 0.9992231469133148 0.9586141502640423 0.9992411937172776 0.9584539299030572 0.9990904417413572 0.957805703163017 0.9594402661504962 0.9576243322636385 0.9993174486555156 0.9582908546798029 0.999820866554763 0.9578258718513084 0.9993580832282472 0.9584201133004928 0.9993266129369899 0.9576706260826526 0.9993747614042464 0.9578496676441839 0.9991287912087914 0.9584440684596578 0.9992979659949621 0.9582473599003735 0.9993178909703221 0.957684684576637 0.9997122588832489 0.9581361585665193 0.9996623029661017 0.9577967388132295 0.9992613655462185 0.9581378806789816 0.9992953736730362 0.9580140251417305 0.9991580186480187 0.875420699755899 0.9993155149080144 0.958200044620724 0.9992480915389461 0.9581407065750736 0.999337668009328 0.9585722394116155 1.0 0.9581081288418982 0.9997264953560371 0.9584493867457964 0.9992766461603734 0.9580987788697789 0.9992524408945687 0.9580286415187378 0.9997207133784929 0.9577583422720625 0.9994040600250104 0.9577941773084478 0.9994598363064008 0.9579695890071801 0.999105938983051 0.9582817859796879 0.9580102226478334 0.9578987082514735 0.9580102226478334 0.9582401652688702 0.9579939124122914 0.9577175194174758 0.9577004346764346 0.9578947131248461 0.9994119024696525 0.9580728539659008 0.9561222106013787 0.9582603832923833 0.9571736529906079 0.95794177734375 0.9570206431026018 0.9576447461803845 0.9578483317025441 0.9588037925043436 0.9577854155363749 0.9578818370334227 0.9579679410319412 0.9580741565090198 0.9582052118959107 0.9993616087325138 0.9579263720930233 0.990480965116279 0.8722265250862755 0.958649579915486 0.9984959746045889 0.9581963749378417 0.9575573851502359 0.9581326171485544 0.9578561250310097 0.9576262717258263 0.9586566291291291 0.9582804635276897 0.9900905908768612 0.9576935811467445 0.9576769825072887 0.9575630748663102 0.9577081287970839 0.9577040850546782 0.9571534724999999 0.9576973593598449 0.9576424139602522 0.9577555348047538 0.8762864317302377 0.9577054434400195 0.9993750747238466 0.9993712002595156 0.9577317728937729 0.9577281143136297 0.9577356375183196 0.9993786130913804 0.9581349888420532 0.9577746650124069 0.9578028812298537 0.8761944164882227 0.9993739493616102 0.9993916092700889 0.9577573685497677 0.8796119398340249 0.8758265369127517 0.8766488790560472 0.8771350380848748 0.9594284487270998 0.9593672940052543 0.9584232846895868 0.9575411625119847 0.9572016550695825 0.9441722372794682 0.9580412347486579 0.9579115390243904 0.9581036348081154 0.9580113377353877 0.9579612722832722 0.9579995501222495 0.9575761149653121 0.9579029452563785 0.9581629514321297 0.9582062978617605 0.9575690136307311 0.958355437062937 0.9581754391639713 0.9582415823412699 0.9582310171173407

GCA_015684315 0.8765394168307087 0.9726964412094066 0.9595512252964428 0.9598919440654843 0.958126626741628 0.971358024047187 0.8849242218292066 0.9739086164623468 0.9716231455507013 0.9725990927021695 0.8819985628326776 0.9726451072961374 0.8819516960556844 0.9596652488584475 0.9910334765258216 0.8851061819103118 0.9726758239277653 0.957653496947497 0.9728813027075012 0.9576775497665274 0.9719137372781066 0.9581108908957774 0.972517317181596 0.9581787851158207 0.9727396478555305 0.9578975569713306 0.9723834267289304 0.9579986787819255 0.972844346956908 0.9579018042366692 0.9735500988541901 0.9577671989207752 0.9724531098240142 0.9709480569758949 0.9908661714898179 0.958228702795488 0.9912807249837206 0.958038347147733 0.9725592742295669 0.9576008788540908 0.9913112824956675 0.9577824294747082 0.9899779939912179 0.9575872334223949 0.9726487888268157 0.9575743166869671 0.9732062259562237 0.9577077024390245 0.9737797755056679 0.9576925632911392 0.9724005385124698 0.9580601414979265 0.9912339207620697 0.9581862341153472 0.9725339212633451 0.9577679655674104 0.9737721751792116 0.9576726351680467 0.972386188787946 0.9578439995137369 0.8793653501843075 0.9577726146010187 0.9735667597888253 0.9575034126213592 0.9912608986822208 0.9578792184075969 0.9716768558845119 0.9580352832929784 1.0 0.9579500895450146 0.9713697415836889 0.9577615418287937 0.9911893313069909 0.9577848209500609 0.9726223871547273 0.958112299243349 0.9725489511269806 0.957829577053962 0.9722721351291677 0.9576611561816857 0.9721805995096946 0.959781299448022 0.971608093533763 0.9700848681397006 0.972582673245122 0.9700848681397006 0.9728034313503733 0.9713246201091902 0.9724531976873471 0.9722261820652175 0.9729872558659217 0.9575196509090909 0.972588358043815 0.9902639542483661 0.9913212900432901 0.9908077563499529 0.9724901230149854 0.9901525970987365 0.990055719531788 0.9906227565577544 0.9721017053550908 0.9908677725423003 0.9725633528748592 0.9722594334532374 0.9725471985575841 0.9738039673072675 0.9578872968369829 0.9725462964611618 0.9590829695203945 0.8841427138714355 0.9727708483056631 0.9591300593401377 0.9912819318181818 0.972528674966652 0.9729100133868808 0.9726998785971224 0.9727112634114299 0.9728594671849989 0.9736210410301177 0.9592411577726219 0.971782191446931 0.9716446239183492 0.9907026529710626 0.9716178243512974 0.9717560465631928 0.9713077876307412 0.9717615537848606 0.9717667950420541 0.9717878530323151 0.8782016014042666 0.9709609473208229 0.9591166697674419 0.9591357610969092 0.9709584603210492 0.9710651542445397 0.9709733129805517 0.95949484673888 0.9720963082271148 0.9706728239283119 0.9706978528201403 0.8846275879271639 0.9594274435223605 0.9593773802946594 0.9710111313950868 0.8864226144756278 0.8848813381510092 0.8771878018818611 0.8851645332996974 0.9725837427031881 0.9724842503376857 0.9736967022459417 0.969661320932423 0.9904930542563143 0.9483983815756356 0.9712192141409592 0.9710645442207646 0.9713172009029346 0.9712811018099546 0.9710145727848101 0.9710400769753227 0.97129717039505 0.9712511293390393 0.97132231025458 0.9713319043084979 0.9712872411330636 0.9708947479596735 0.9711826379928316 0.9717154799183118 0.9714331326949386

GCA_002334085 0.8701998683864174 0.9576430621774392 0.9988929750437828 0.9994044312283739 0.9990903707815275 0.959192262075562 0.8767363850795392 0.9592905704057281 0.9590542192046557 0.9577885811965813 0.870690362934363 0.9588219761336515 0.8705159008776459 0.9993938908070782 0.9576144056815349 0.8762814428803465 0.9576075712515489 0.9993324390765286 0.9576320424597365 0.9992930248648648 0.957760618226601 0.9997691615498624 0.9577604766536965 0.9997207165844239 0.9582780417177915 0.9993143719653789 0.9580353525954947 0.999292794656324 0.9587468280987774 0.9993683368288591 0.9582731720029785 0.9992001535508637 0.9578363463877402 0.9592372432170543 0.9575104538727183 0.9994989831950649 0.9581595904268443 0.9998397896950578 0.9578759637964775 0.9994684659090909 0.9581635837438425 0.9994647249529386 0.9575105581741505 0.9995098967986521 0.9578023236514522 0.9991152624894157 0.958502181150551 0.9994038043250053 0.9582889115815693 0.9993702062289563 0.957471103662916 0.9997259381617959 0.9581178778926638 0.999750105820106 0.9578213427389929 0.9993426099768471 0.9582247825000001 0.9993274564015313 0.9580102141274921 0.9992943676939426 0.8754643334238393 0.999393695468022 0.9579658063719438 0.9992784599200506 0.9580051743614931 0.9993853927813163 0.958528492628368 0.9997926709490261 0.9580962371134021 1.0 0.9584451871127634 0.9994840424628452 0.9581896323710831 0.999370004258037 0.958165884824518 0.9998330312035661 0.9578014463414634 0.9995056102649698 0.9576684779411765 0.9994549158603282 0.9579595132196689 0.9992848255550523 0.9582999380114059 0.9581321644295302 0.9579215390297957 0.9581321644295302 0.9581944449901769 0.958007302583917 0.9577661113813232 0.9573804807223035 0.9577329909068567 0.9994345608531995 0.9581525185368266 0.9558693592785952 0.9581509500369186 0.9570025987166833 0.9580077045177047 0.9570817472392638 0.9575324901283316 0.9577749633431085 0.9587942424242425 0.9577153068770027 0.9578656272838003 0.9579738030935429 0.9579262935020687 0.9582972533992582 0.999097091024825 0.9580783125153074 0.9904852221446848 0.8724991544801916 0.9585426033160108 0.9988061137123747 0.958280730616302 0.9576691720376798 0.9583213369917685 0.9576679960366609 0.9575527154750245 0.9586497528089889 0.9582706024390244 0.989862240850059 0.9575580402131785 0.9575444390598498 0.9574121295173416 0.9575035004844964 0.9575313465730202 0.9570513715710723 0.9575628433268858 0.9575617335589942 0.9575708585247884 0.8766529401993356 0.9576965682206492 0.9994521722198182 0.9994530233564014 0.9577226591851673 0.9577090678379697 0.9577243853658537 0.9994226741670272 0.9583240114257326 0.957621362962963 0.957630059215396 0.8762640531829169 0.9994497858070099 0.9994695495885665 0.9577653921329099 0.8798432354469856 0.876192662530348 0.8766012590604028 0.8774827498634625 0.9595450536608634 0.9593506417624521 0.9585629535343548 0.9574619516283527 0.9569339736907421 0.9442869472876153 0.9579701901048014 0.9578299829393128 0.9580339760800587 0.9579356214896215 0.9578843972669594 0.9579121576763487 0.9578535392980722 0.9578917354758962 0.9583541631434968 0.9584090836851255 0.9577953574078655 0.9585498653702318 0.9584119652173912 0.9581680272614622 0.9581769417596034

GCA_015684335 0.8717693342070775 0.987047355163728 0.9562651686746988 0.958380487923884 0.9580295894138017 0.9885911607949411 0.8788662679425838 0.9776750223266745 0.9898478022232514 0.9885381541950113 0.873235408977556 0.9900147566574841 0.8732126056514129 0.9581895556640626 0.9723427723735408 0.8789453312051078 0.9870879123414072 0.9578159786653437 0.987762329830973 0.9579942465753424 0.9766999693034238 0.9573099366626068 0.9884567361111111 0.9575688784816367 0.9887095599814729 0.9572058372150035 0.9889868078175897 0.957199653307106 0.9890085237543454 0.9575118317437026 0.9902758941774492 0.9572604181013281 0.9884601042149143 0.99048779178338 0.9702738222849085 0.957999134424603 0.9718357032569359 0.9573078479161589 0.9884725564084671 0.9571952517635611 0.9717981333011351 0.9572596730112249 0.9717597389412618 0.957281407660405 0.9884864785133566 0.957126568843238 0.9898444985350462 0.9572730073349632 0.9779528184216838 0.957207087804878 0.9877425197205318 0.9573891560975609 0.9717568005773395 0.9574031219512196 0.9884721153846154 0.9573155333170612 0.9894112613224638 0.9571823494860499 0.988255523415978 0.957414908245657 0.8804913459516298 0.9574878723404256 0.9775024347413385 0.9571726705653022 0.9717378459687126 0.9572673914104443 0.9993719125326371 0.9578163947757515 0.971871057669299 0.9576755325953261 1.0 0.9572915148563078 0.9718138972370335 0.9573772387695314 0.9890968370457209 0.9573641593567251 0.988431976798144 0.9573250121832361 0.9882218641354071 0.957390288184438 0.9882861882787264 0.9583251767304861 0.9994410263929617 0.9888405979990905 0.9884413369108727 0.9888405979990905 0.9889733605220228 0.988742019738352 0.9884680610828319 0.9894564207650274 0.9871958329529895 0.9571171366173259 0.988413248393021 0.9711249372586873 0.9718205327868853 0.9706028664573775 0.9883669432821944 0.9704890672088017 0.9717583417326614 0.971088410880458 0.9896959204780511 0.970912758374911 0.9901333619909503 0.9891482115121061 0.9893829451287793 0.9778913216068458 0.9573929359129385 0.9891117306350313 0.9577320572109654 0.8761161357234316 0.9771634963680387 0.9579893045443134 0.971831352140078 0.9889079882059424 0.9889200091157703 0.9870786384544619 0.9879808941646329 0.9772421936903888 0.987817755782918 0.957957493371897 0.9875354722096702 0.9875179643018527 0.9707638117134559 0.9875522076749437 0.9876787856819212 0.9864691496756255 0.9873882193635748 0.9873387006541845 0.9873858677499436 0.8773006936572201 0.9880777119416592 0.9577774858269658 0.9577956727451948 0.9879890933940775 0.9879964502164502 0.9880187155545435 0.9577045668135096 0.9990840845070423 0.9879103102570039 0.9879197039093223 0.8783088252375923 0.9576935015927468 0.9572747055949182 0.9879126812585498 0.8822764809608996 0.8790327157894738 0.8779726991954323 0.8801325151435343 0.9898410946944257 0.989762612693429 0.9901451127305854 0.9888199635452267 0.971856468996063 0.9507048653410521 0.9882133994980606 0.9882832333409663 0.9883450583390528 0.9881729360531744 0.9881649713762309 0.9883028558331423 0.9773627200193424 0.9763418607442979 0.9774165699782451 0.9775063684719535 0.977422091898428 0.9768037263004375 0.977336561064087 0.9892400598526703 0.989176497812572

GCA_002334105 0.8704871602005806 0.9571595796890183 0.9982463380281691 0.9992534091894235 0.9992526844444445 0.9576483183910831 0.8770793675027264 0.957796060458313 0.9578098309719115 0.9579539083557951 0.8710415838269381 0.9587696431137726 0.8708901007491603 0.9993650432525952 0.9578806918081918 0.8774068075245365 0.9571920858895706 0.999628133447758 0.9573861312984496 0.9995947618011745 0.957592028557361 0.9998087470850117 0.958157784533074 0.9997216368727116 0.9582702092050209 0.9998540752416983 0.9583214541441882 0.9993852007648184 0.9586439947026247 0.9996388961038963 0.9584240452511189 0.9995610121199235 0.9582620861733204 0.9592101868478525 0.956950014698677 0.9992760613258477 0.9582435082941321 0.999854642178594 0.9583894098922626 0.9998702167569821 0.9583847441285537 0.9998369494697442 0.9580192752179326 0.9998660591543428 0.9582256639765225 0.9997007481139982 0.957950947878788 0.9997509339740911 0.9578934032459425 0.9997715537432036 0.9577364593878042 0.9998223119188504 0.9581703559070688 0.9997755835628044 0.9582064337877313 0.9993739118515395 0.958248975256186 0.9996865446898003 0.9580260783341596 0.999359751328374 0.8759834246575342 0.9994124783252274 0.9575839896881907 0.9997858851774531 0.9581723397435897 0.9998707895842083 0.9581603855975486 0.9992417921527041 0.9578161885954087 0.999245537452391 0.9577402184585173 1.0 0.9581707488222168 0.9998940662086195 0.958441287128713 0.9998772256421141 0.9582209484233686 0.9998603091286308 0.9577696007885658 0.9998451630434783 0.9580629179104477 0.9988777598729006 0.9581263848251923 0.9579140269101395 0.9583148414843942 0.9579140269101395 0.9583082931034482 0.9579439568170791 0.9581773085339169 0.9577155087031136 0.9572781075164194 0.9998496575342468 0.9581980019856043 0.9570882979749277 0.9581412842677203 0.9569708110781404 0.9583697873380592 0.9573040162521546 0.9579202132936508 0.957809226854096 0.9580937487708948 0.9577902426944032 0.9579866683010542 0.9580825104602511 0.9573377549043351 0.9580815388421574 0.9989924319066148 0.9584215948064675 0.9903555805330244 0.8728207169509595 0.9582211376055639 0.9985273513634332 0.9582707630922694 0.9574587728847105 0.9585859139784946 0.9572677168054875 0.9569060672797677 0.9583235614035088 0.9585695894428152 0.9901377297297299 0.9578244884568652 0.9578698590179875 0.9577012792968751 0.9578374678007291 0.9578858677685952 0.9574114582291147 0.9579355056997332 0.95792244724715 0.9579476880155265 0.8770763311331135 0.9577372794117648 0.9993708054711246 0.9993801584545257 0.9577700293901542 0.9577789754901962 0.9577733390494857 0.9993970284349902 0.9582179227531569 0.9579126164519326 0.9579398860822191 0.8766700877893057 0.999379489019352 0.9993219161416468 0.9578201518863303 0.880435157490397 0.8764170959935466 0.8765884950547982 0.8774620633187774 0.958468971916972 0.9583925691893215 0.9579497264957264 0.9578779365079366 0.9573290818858561 0.944525358335474 0.9580086175678981 0.9577685280431056 0.9581160583762571 0.9579342899190582 0.9578205954422935 0.9578006009320581 0.9579255315985131 0.9581145162088592 0.958457043305127 0.958515042246521 0.957940838693716 0.9586542001497379 0.9584851789264413 0.9581935758629253 0.9581927787434815

GCA_015684395 0.8766098586744638 0.9726133989820757 0.9590244510794673 0.9593243575418996 0.9574557554479419 0.9713790376282782 0.8861555516461422 0.9738532693551865 0.9713736265502988 0.972606107836717 0.8809562809349221 0.9722374966352625 0.8810672915230876 0.9588589926124916 0.9909553713260042 0.8854195113350127 0.9725843997317236 0.9570553333333335 0.9727589358440991 0.9570824302692215 0.9713200900900902 0.9573733566433568 0.9717133848531684 0.957380380859375 0.9730291149243621 0.9569504986750181 0.9718099889575972 0.9572793028904542 0.9723607155399472 0.9569358397312859 0.9739585723905724 0.9567612720251027 0.9717586828729282 0.96994969286578 0.9903493395824456 0.9573557614768035 0.9996469404279931 0.9573102675988429 0.9718665874777976 0.9569371312260536 0.999641642651297 0.9570529366602688 0.9981178562313909 0.9569153903256705 0.9718459929078015 0.9568342672103622 0.9735377033917092 0.9570238981014179 0.9736683070521245 0.956960770338373 0.9721931038275407 0.9573817539203862 0.9996419638739431 0.9575148274197441 0.9717262599293911 0.956512189433603 0.973437747910549 0.957157053872054 0.9725518988739237 0.9565848367103696 0.879480768029159 0.9566215075017863 0.973208823016565 0.9569224401340997 0.9996559045611346 0.9570437916966643 0.9705611668273868 0.9567608355153536 0.9909276909944517 0.9566286268443598 0.9709762582938389 0.9569502274904216 1.0 0.9568881855225311 0.9719685065657688 0.9573354378769602 0.9717141389995573 0.9568459276993059 0.9725761782178218 0.9569135830540928 0.9724097219132369 0.9586032738767216 0.9708964514597673 0.9702502767835838 0.9718939128501556 0.9702502767835838 0.9728266030534353 0.9716687188855904 0.971739181196204 0.971800874439462 0.973019342743714 0.956908977544195 0.972446413306896 0.9908148000846204 0.9996724903846155 0.9908162861736334 0.9716753548387097 0.9908005988985774 0.9981424243706368 0.9906521592920355 0.9721557447301368 0.9901789610944849 0.9722422107158937 0.9720759038290294 0.9722507759784076 0.9737806511829824 0.956768614501077 0.9718364023851589 0.9589149744160179 0.8849622363986519 0.9729137095694848 0.9581577286478938 0.9998299709414955 0.9725427679964342 0.9733482115471358 0.9726550691038788 0.97217784296427 0.9729375770528685 0.9738952471566055 0.9598541867881549 0.9717943361456484 0.9718274411895251 0.9912830864726029 0.9717945254988913 0.9717861256660746 0.9711286303252404 0.9717746807095344 0.9717971751662972 0.9717990949423247 0.8783509978483056 0.9706459807218113 0.958178673655424 0.9581594229014598 0.9706667773043283 0.970797393343757 0.9707023654708521 0.9584786398553999 0.9719873271461716 0.9700932006713018 0.9700904861111111 0.8860367797869705 0.9584550339059675 0.9580446306306307 0.9707042537980342 0.8858567110358179 0.8848156352765323 0.878661277486911 0.8851447112462006 0.9726937530753746 0.9725971976665919 0.9738656024096385 0.9693423689826892 0.9898411841491842 0.9482365172917682 0.9710875 0.9708646233183856 0.9711104700089525 0.9711059793583128 0.9706887491608861 0.9708739030955585 0.9711241855524079 0.9708890883392226 0.9709905799151345 0.9711359579990562 0.9709649539985846 0.9705678530805686 0.9709127049373968 0.9718439921946741 0.971683928653625

GCA_002334125 0.8698042796944958 0.9572978710462288 0.998229168286887 0.9991462118918919 0.9991485284354945 0.9573569277254049 0.8761848275862069 0.9572563840155947 0.9576107722870625 0.957690141809291 0.8701728221649485 0.9586475532169338 0.869971069767442 0.9993084998920786 0.9576828194271483 0.8762879302832245 0.9573293591946969 0.999541314443971 0.957324860808521 0.9995588951230039 0.9571395604125736 0.999756328471782 0.9579161910549344 0.9996009515645092 0.9580801989194498 0.9997077619744823 0.958069064871481 0.9993795892441244 0.9586073877551021 0.9996039811912226 0.9581050557620818 0.9993784876085576 0.9579489262235208 0.9590581384133108 0.9570834605971611 0.9992734824693481 0.9580803744764721 0.9997640933950358 0.9580899045053869 0.9997891846921797 0.9581387876543211 0.999745951391774 0.9575745068322982 0.9997806529423996 0.958057630806846 0.999495448290242 0.9578637085451464 0.9996909616985845 0.9573671478088636 0.99965844115192 0.9574223259152908 0.9997194452647119 0.9579666519065191 0.9997347078675385 0.9580334137426902 0.9992940395124003 0.9578728891656288 0.9997526323498641 0.9576693589743589 0.9993975910245556 0.8750829355354287 0.9993879143821173 0.9569364144495974 0.9997262476547841 0.9579076851168513 0.9997914040594268 0.9580362261753494 0.9991999618886301 0.9577363453025086 0.9991869206349207 0.9575173011989234 0.999808191797846 0.9579468076162216 1.0 0.958209906219151 0.999840311837081 0.9580107609756098 0.9998153924207911 0.9575822730627307 0.9997593322238402 0.9576785038394848 0.9989272311523659 0.9580412990861942 0.9577107531782203 0.9581917034855181 0.9577107531782203 0.9581534343434345 0.9577465060532688 0.9578735431348726 0.9574786748466257 0.9573740754257908 0.9997903633720931 0.9577345833333334 0.9569783798748195 0.9581197761377613 0.9570867341897232 0.9582053136441299 0.9574971062992127 0.9574739294523927 0.9576718265281471 0.9578011225238444 0.9578408859822309 0.9578029970617042 0.957914744346116 0.9571343445602132 0.957573684988908 0.9990987373192316 0.9581701323853886 0.9901997522000926 0.871962432216906 0.9576838431081415 0.9984001251117068 0.9580648332503734 0.9572086958661418 0.9580337453369809 0.9573709217603912 0.9564907328833172 0.9577229061326658 0.9583300024384298 0.9900227781698423 0.9576547530266344 0.9575657758412007 0.9576607531074826 0.9575906198547215 0.9576091333817478 0.9571264871603091 0.9575948863636364 0.9575717500604303 0.9576547147001935 0.876022634039043 0.9574403682028775 0.999339222270364 0.9993118930041154 0.9574616979293545 0.957479434422233 0.9574697465886941 0.9993278285343148 0.9578909299457327 0.957480133201776 0.9574944452662724 0.8758223709976184 0.9993136013001084 0.9993168538594971 0.9574546318868845 0.8794752557544757 0.8753155311158797 0.8759718020917135 0.8762512799564272 0.9582573792093703 0.9581573688064439 0.9575035550122248 0.9576038024454567 0.9570167516712057 0.9444257183170858 0.9577536345919611 0.9574824055569096 0.9578498828411034 0.9576942707060836 0.9575104343582236 0.9575231256109481 0.9576251103396974 0.9578097867592362 0.9581304109589042 0.9582180487804878 0.9575407578008915 0.9583166974768925 0.9581930418535127 0.9579227051822465 0.9579030960059539

GCA_015684435 0.877173596963761 0.9922914417822615 0.9594538617045716 0.9595800270758124 0.9574117943743938 0.9887426118421054 0.8852523057581092 0.9772177631289828 0.9877956534344335 0.9891008783357849 0.8802774512534819 0.9926071241554054 0.8803080344266109 0.9595115994562755 0.9700115580057527 0.8854438734879032 0.9923123005291007 0.9574254133333334 0.9924979355509355 0.9573308641674779 0.9755393529411763 0.9576523400144893 0.999987962606014 0.957722785243098 0.992586428872942 0.9574506563332527 0.9998670823620824 0.957426166056166 0.99224237986744 0.9573969386769676 0.9900356560891939 0.9574853224628865 0.9998703035019456 0.9885655627306272 0.9719428783902012 0.9576315839416059 0.9723855043036856 0.9576879555340745 0.9998674995112415 0.9574323306102835 0.9723911932068814 0.9574179099446184 0.9702018486997637 0.9573740490502525 0.9999868564787915 0.9573284767413834 0.9901364462809917 0.9573181719391451 0.9779587186876524 0.9572280327474115 0.9887148471431583 0.9576839700555422 0.9723788340312983 0.9577300121006777 0.9999818032786886 0.9573356406551059 0.9884802648719062 0.9573604504938569 0.9887185871404399 0.9573809705456302 0.8802837093839355 0.9572064625030141 0.97714307436919 0.9573858962037483 0.972354990094651 0.9574441913714148 0.9876368349377788 0.9572896490384616 0.9720707721238938 0.957239963933638 0.9877654807033568 0.9573893703436674 0.9723358828729282 0.9573976396917148 1.0 0.9577026570048309 0.9999224292909724 0.957385119133574 0.9887987679022747 0.9573309523809523 0.9887877073585708 0.9585341164522682 0.9879238621794874 0.9894378129952458 0.9998374788925978 0.9894378129952458 0.9925768529411765 0.9897731548007838 0.9998680182453417 0.9878100438116101 0.9925735434554973 0.9572833077660594 0.9888201663508107 0.9709378406802417 0.9723670143329658 0.9695765286090496 0.9997738262910798 0.9697186879011171 0.9702080390312721 0.9700974307836697 0.9889055447082317 0.9720155286679747 0.9884147634619578 0.9916848728636933 0.9884534482006544 0.9780330753924387 0.9574429079198472 0.9998640478506129 0.95853375 0.8837491546934867 0.9770690353260869 0.9585708292224571 0.9723861269487752 0.9892508288712787 0.9891968327169893 0.992306244458518 0.9905396171742809 0.9770765162907268 0.9886604471286289 0.9590716877443091 0.9883121582887701 0.9883178341902314 0.9709747943358058 0.9883170351328192 0.9884348148148148 0.9876176083608361 0.9882713076923078 0.9882545118564409 0.9882764174321725 0.8780105103411227 0.9877328831562975 0.9588622814302191 0.9588711592532841 0.9878697528723174 0.9879148703543648 0.9878689047928865 0.959108051176605 0.9886568842199376 0.9877731205673759 0.9877854090493601 0.8841137750309024 0.9591145475319925 0.9587779576502732 0.9878110669546436 0.8848008918918919 0.8843465107018416 0.8771119653634217 0.8850406056018169 0.9881610325501186 0.9881385862516214 0.9894724042186828 0.9891340979147779 0.969541372220894 0.9496825099009901 0.9880509016393444 0.987753079420039 0.9881409254054054 0.9881342980748433 0.9875447838305234 0.9877858365843086 0.9755420118623963 0.9754893918599149 0.975590663507109 0.9751992701421801 0.975438691943128 0.9751696906971211 0.9755351602183717 0.9881585499557913 0.9880652050543117

GCA_002334145 0.8700924432717679 0.9574516743733268 0.9982803970651705 0.9997237861334494 0.999262243902439 0.9585242432113341 0.8768025198575734 0.959019866475918 0.9593170284740812 0.9581140904621436 0.8702268131011178 0.9590892117872545 0.8700143776041666 0.9997604296875001 0.9580355825000001 0.8763814618931381 0.9574991664617654 0.9994641054001302 0.9574783963178294 0.9994910920676775 0.9576990207202764 0.9996818913270639 0.9583856815410876 0.9996031620135364 0.9584199286417322 0.9997403221730891 0.9585635747606186 0.9994863778683253 0.9590275192678227 0.9997660067043788 0.9583657537313433 0.9995657908217715 0.9584584455832114 0.9595352450266862 0.9570694343780608 0.9995394670376713 0.9584107890818859 0.9998103115264799 0.9584919995093231 0.9996824937027708 0.9584505404065443 0.9996820209205022 0.957982006989516 0.999675396424816 0.9584486348039215 0.9993172203461377 0.9581551443123939 0.9996018237018426 0.9582026130402198 0.9996992749054225 0.9579592836468886 0.9996366194542803 0.9583334900990099 0.9996392227110832 0.9584599951183793 0.9996693641497595 0.958254068896655 0.9996253309214725 0.9581833432539683 0.999564596247101 0.8756990454423231 0.9997261259742996 0.9576382263222633 0.9996525494736842 0.9583302322708178 0.9996766024147427 0.958532741440981 0.9996029567966281 0.9582804253214638 0.9996116975178798 0.9575668234141563 0.9996538344272707 0.9583982682259267 0.9996741069906224 0.9586636561803319 1.0 0.9584504115629594 0.9997019418483905 0.9580616600790514 0.9997503896920175 0.9581735781872509 0.999484413573218 0.9581890720118783 0.9580605983112185 0.9584417246484085 0.9580605983112185 0.9585624006908464 0.9581424178232287 0.9583919697708434 0.9578020624385447 0.9575328164556963 0.9996697098121086 0.9583236460660214 0.9561212780697562 0.9584222112047596 0.9571746782178218 0.958557181372549 0.9571924649446494 0.9579688514016372 0.957899316694936 0.9581786807777505 0.9578910970777612 0.9580545150994354 0.9582932528339083 0.9575340972053463 0.9581391584158416 0.9996249925516066 0.9586050675509704 0.990532513368984 0.8726171641791045 0.9586567880299254 0.9989294078212291 0.958473797152136 0.9574631657171168 0.9585687471867966 0.9575267868530783 0.9571752464190337 0.9586833316582916 0.9584395499021526 0.9901405121548267 0.9581008760370913 0.9581301611328126 0.9576485832926234 0.9580839507076622 0.9580940936813857 0.9575521081761007 0.9580912600536193 0.9580889785470504 0.9581535390243903 0.8766093287292818 0.9580485458118397 0.999785609650076 0.9997884115601912 0.9580847274066798 0.9580867026761601 0.9580972312223858 0.9998130078809107 0.9580081539980256 0.9579405974219138 0.9579649950421417 0.8764081033564199 0.9997789019352032 0.9997775146898805 0.9581060388015716 0.8801065238709675 0.8760381862479697 0.8757911612206319 0.8773404699093159 0.9596455357142857 0.9594384106118545 0.9580268113809173 0.9580312442729685 0.9573811288312983 0.944467735897436 0.9582600957995577 0.9581978741705578 0.958331247846419 0.9582141806102362 0.9582604105211405 0.9582773579335794 0.9583775379636545 0.9579189782285998 0.9583173837353891 0.9583859433258762 0.9583624888115365 0.9585704938887503 0.9583682057654076 0.9581341695587507 0.9581441115241636

GCA_015684455 0.8768703807418325 0.9917020441176472 0.9587055123513267 0.9596266530981455 0.9576727310111273 0.9887233970136144 0.88511781053162 0.9770490041951867 0.9876668674429019 0.9893097358968941 0.8801970180722893 0.992535759439966 0.8801308637102392 0.9594733340903927 0.9702419560492636 0.884734668515251 0.9918117321163235 0.9569262780593772 0.99188947576451 0.9571045176926807 0.9748570666979802 0.957386649051165 0.9998943734359961 0.9574054638173871 0.9920909911429776 0.9572478872560829 0.999798421875 0.9571862971926427 0.9923620592530774 0.9571764688926255 0.9900898425026969 0.9571211559854897 0.9998629945588806 0.9887136414176512 0.9718619263830724 0.9574438379685611 0.972321192865106 0.95740672993753 0.9999149540027402 0.9570599689515166 0.9722845391807657 0.9570954612988257 0.9697950632005724 0.9570078484341383 0.9999408871279474 0.9569736981041517 0.9903222471190781 0.9570122703222704 0.9776958633013179 0.9570026358630598 0.9886751152463523 0.957377559452318 0.9722627406090242 0.9574792576524463 0.9998952368117059 0.9570107231800766 0.9877934108695652 0.9570074274058076 0.9889874813552099 0.9570398078308913 0.880385607329843 0.9569883969282457 0.9769681664441477 0.9569831787762907 0.9722389470183995 0.9570774575053866 0.9870379952941177 0.9569862075561932 0.9720868131132917 0.9569455314067352 0.987466944 0.957121790580923 0.9721949131789849 0.9570893215056342 0.9999376850763808 0.9573886530612246 1.0 0.9571062511961722 0.989060981802793 0.9570191937799042 0.9890025749143837 0.9583526873443513 0.9873170297482837 0.9888375776820141 0.9998649301868241 0.9888375776820141 0.9920588764990533 0.9892807047785293 0.9999126362754607 0.9873640026189436 0.9921041323529413 0.9569997068636797 0.9890194628450106 0.9709885030343898 0.9722536361612113 0.9696156637806637 0.99974626171875 0.9693229857142857 0.9698317195641876 0.970038604815864 0.9885565105908584 0.9719008635464733 0.9878349815016323 0.991586802763819 0.9878212956521739 0.977798539901917 0.9570796981221773 0.9998573446658852 0.9585972630866425 0.8833396847172083 0.9768109622383985 0.9584880442457049 0.9722331530520646 0.9888858747579083 0.9888490037142234 0.99176078012685 0.990322740425532 0.9769371543224031 0.9881374925500214 0.9587731709558824 0.9882644759871931 0.9882136217948718 0.9708901491862568 0.9881875154881437 0.9882977537935458 0.987602560492741 0.9882552636072572 0.9882618548559232 0.9882502582159625 0.8779777378718842 0.98756288624052 0.9581819257221459 0.9581981591013298 0.9876175856832972 0.9876750723698423 0.9876312318369119 0.958480849613812 0.9877737472085754 0.987795121671258 0.9878034839449542 0.8840164662027834 0.9584939627357418 0.9581430452488688 0.9876456636402939 0.8844715675675676 0.8843292280789408 0.877442324790795 0.8843740675403226 0.9873937345745831 0.9873298957654725 0.9893529298397574 0.9890267288444041 0.9697206910666987 0.949728326328801 0.9878504862762049 0.9875302623021895 0.9879392027729635 0.9879839436008676 0.987308943265483 0.9875542475570034 0.9753345064072142 0.9748091226415093 0.97536786628734 0.9749691797060217 0.975285968238919 0.9748961838970938 0.975225188791261 0.9876524418346998 0.9875165252166184

GCA_002334165 0.8705521012591815 0.9571851550387598 0.998057205787087 0.9993771283051582 0.9990091498335183 0.958631721947777 0.8767256592512207 0.958928377867108 0.9591612166747454 0.9579237735387625 0.8711529899820191 0.9587668493803623 0.8710144582043344 0.9994836751027473 0.95785365805169 0.8768840149692598 0.9571635642594859 0.9995149619611158 0.9572569585142305 0.9993487962369041 0.9576666846229186 0.9997174589311709 0.9580077031287897 0.9996501515151516 0.958188126073093 0.9997689334980442 0.9582204571009534 0.9991305947089947 0.958596142788692 0.9996460143589744 0.9582080554181098 0.9996042064810968 0.958134656470017 0.9593375199612874 0.956795635345667 0.9992476674413692 0.9582502852225229 0.9997931828488971 0.9580917044623263 0.9997076264591439 0.9583403954802261 0.9996205626911315 0.9576417263521857 0.9997044653830398 0.9581011287176986 0.9993822619293535 0.9578577339782346 0.9997317508692984 0.9577629331683168 0.9996727298791727 0.9576537994722955 0.9996897226247154 0.9580681256133464 0.9995663681695409 0.9580994487615349 0.999359486542443 0.9580850922233302 0.9995615216940005 0.9577910167818362 0.999244701695625 0.87580262107504 0.999427254697286 0.9575031553279689 0.99963664 0.9580851285819251 0.9996390820957096 0.9582615670312898 0.9993065554404145 0.9581115411127523 0.9991671464019851 0.9577861694335938 0.9996414657845771 0.9582166986942596 0.9996410099750623 0.9582187352071008 0.9997312282157677 0.9580281138963251 1.0 0.9575928694369314 0.9997840216724596 0.95784767839445 0.9989135171318358 0.9581198321402122 0.9579571199425425 0.9582022026431719 0.9579571199425425 0.9581904151314175 0.9579699226305608 0.9580421177610855 0.9575186415831908 0.9572901017935047 0.9997105054004483 0.9579885992601725 0.9559005591092159 0.9582689828009829 0.957288012804728 0.9582356064296151 0.957299329253366 0.9576459936055092 0.9576965888862161 0.9581790791084985 0.9578360521781935 0.9576258969978033 0.957987934915586 0.957092150615496 0.9578072550467749 0.9991345099281792 0.9582902297726716 0.9904626580226904 0.8725569755708976 0.9583177039050915 0.9984666971046771 0.9582860515873016 0.9574010355029587 0.9585236338729761 0.9572985546588408 0.956804571359575 0.9583922136261542 0.9583716204379563 0.9902307223529413 0.9578641422695379 0.95777870343493 0.9574241591846641 0.9578059230583111 0.9578169600967352 0.9574242415940224 0.9578737493964269 0.9578504683727669 0.9578865241545895 0.8765759107093947 0.9577678670901539 0.9994343672672021 0.9994184443480149 0.9577742637362637 0.9578026422466422 0.9578177945840449 0.9995262660013018 0.9580053573188764 0.9577202740740741 0.9577610167818361 0.8762946677215189 0.9994227690972223 0.999421698892027 0.9578092871093751 0.8803169430977289 0.8761104355959382 0.8765420581178351 0.8772668600217866 0.9594755687203792 0.9593951283269962 0.9577799878522838 0.957901103382106 0.9573828416110699 0.9441571257332312 0.9580081410100024 0.9578311916971918 0.9580563718543855 0.9578973300733498 0.9578848595848595 0.957854913139222 0.9575181094404732 0.9576195982252895 0.9580462091179385 0.9580774306930694 0.9575626597582039 0.9581390159045726 0.9580330014858842 0.9581509540286703 0.9581411547972306

GCA_015684505 0.8775061836935169 0.9882112781471156 0.9581015725155638 0.958801803868646 0.9567297138006307 0.9886401545157779 0.8855718892508145 0.9779890295267043 0.9883536291390729 0.9996246222394422 0.8805945108444854 0.9885994559585493 0.880597124047124 0.9584595217685541 0.9689624183162414 0.8841381707626046 0.9881925588934515 0.9560347313649565 0.9885792124814264 0.9562476433276409 0.9763767621247114 0.9565933317249699 0.9887269763513514 0.9565506403700998 0.9893472098068995 0.956138795064118 0.988776894659067 0.9563830969152295 0.9888511130063965 0.9562386917692492 0.9901842276766822 0.9561230459072141 0.9887091816451681 0.9884320063766795 0.9725279138568632 0.9567681857976655 0.9719803109729008 0.956722697416083 0.9887161440407904 0.9560865175565175 0.972026920691183 0.9561143101372501 0.9699824008498585 0.9559778392685274 0.9886898728544183 0.9559128916244266 0.9900617057837384 0.9561253333333334 0.9782270792187842 0.9560629624277457 0.9998522465858821 0.9566498309178744 0.9720465466195316 0.9568107662557411 0.9887102217997464 0.9561730083832335 0.9890751737451738 0.9559580033840948 0.9999912579587112 0.9561834029779058 0.8795151983077737 0.9563440873111057 0.9778024790656678 0.9559330461982676 0.9720219673577415 0.956136031822565 0.9876081268952648 0.9562400598659003 0.9719879612505504 0.9562199928246831 0.9875021892687343 0.955984637019231 0.9719419933701657 0.9561030045816251 0.988779555650502 0.9566540965018095 0.9887463297308753 0.9560258950914342 1.0 0.9559360466794995 0.9999871423032184 0.9576332955575703 0.9876520454029514 0.9874413452914798 0.9887493836638943 0.9874413452914798 0.9889790840190559 0.9878308798792322 0.988721191129884 0.9881658036677453 0.9885008119658121 0.9558384480690814 0.9998128529980658 0.9713409957768393 0.9720399557913352 0.9694974103299856 0.9886022614240169 0.9694113304924242 0.96992205206379 0.9700268219049848 0.9892689442493415 0.9725380585048754 0.9888063182897863 0.9888547992310979 0.9887522454702331 0.9780609605750381 0.9565193793601153 0.9887623472192852 0.9586535550561797 0.8841035023584907 0.977351176731794 0.9576750280767432 0.9720379675339115 0.9891546675191816 0.9883166601899828 0.988290038809832 0.9885136321839082 0.9772798410922113 0.9888872273297118 0.9585886025200457 0.9989296701112879 0.9989272110602745 0.9709875714607246 0.998928276959809 0.99893924055666 0.9980614087098754 0.9989187827640986 0.9989271247021446 0.9989204289970208 0.8771191163793103 0.9985713644524238 0.9576811452834519 0.9576740574052813 0.9986887517475534 0.9986962701412374 0.998693765715426 0.9579096113636365 0.9878768123620308 0.9888130805687204 0.9888185749718151 0.8840436907421196 0.9579189940828403 0.9579650261780106 0.9986529254683142 0.8849620994610484 0.8836043082706767 0.8772316618686208 0.8840817480394638 0.9884285047129392 0.9883559780739467 0.9893991164486381 0.9868904691358025 0.9694600719079578 0.949436034863737 0.998721672958474 0.9988213179374875 0.9987555155254779 0.9988524557917743 0.9988175383695436 0.9988271776447109 0.9763663268684957 0.9763520103945191 0.9764566690324107 0.9764448947243908 0.9764153122043518 0.976293391283639 0.9763284246737841 0.9881013706478625 0.9880343179805137

GCA_002334185 0.8705507332985387 0.9566329452880211 0.9973284739214424 0.9987268404049106 0.9986006768077601 0.9576861904761905 0.8767764480431849 0.9579486699740137 0.95821265625 0.957345629557608 0.8708203300921189 0.9584534169205532 0.8706715944629582 0.9986513151097718 0.9580573346398823 0.8764050477707006 0.9566592984165653 0.999091929933665 0.9566702783777298 0.9989396077197398 0.9572516390290795 0.9989951425005201 0.9575357142857142 0.9989722170803533 0.9578078611449452 0.9990682773542139 0.9578066585603113 0.9985392721518987 0.9580938610315187 0.9989530349075976 0.9579154919852033 0.9991515096272019 0.9576202388419783 0.9587990110683349 0.9563980411622275 0.998658124207858 0.9580041799852472 0.999100328241123 0.9577522977896529 0.9992806833667335 0.9579708769344142 0.9989748844817011 0.9577723781676413 0.999272142571885 0.9576359229651163 0.9990454292623941 0.9569709716890595 0.9990192646456371 0.957039275326596 0.9993616563436563 0.9570394183551848 0.9990281174761313 0.9578102133398726 0.9990348565488565 0.9576457716497339 0.9986642522783763 0.9577514805644961 0.9992546535257062 0.9572941732090284 0.9987132859531772 0.875924799362211 0.9987097098726779 0.9567210965550703 0.9992282600878946 0.9577751357964277 0.9992953697360468 0.9581268461730242 0.9986515424538094 0.9576953567050748 0.9986225704809288 0.957294368118717 0.999111382517049 0.9578580556239232 0.9990489336099586 0.9579270392156863 0.9990937570210109 0.957656652942601 0.9991058282458282 0.957252422982885 1.0 0.9572499827628663 0.9983361321605775 0.958060644223783 0.9576791650943396 0.9577335200974422 0.9576791650943396 0.9577437267839688 0.9572726298076922 0.9575600771828268 0.956294213040363 0.9567007699711261 0.9995501969667125 0.9574243197445972 0.9553054358124112 0.9579320950747366 0.955888643571603 0.957702934334868 0.9556373739342265 0.9577126194518555 0.9571075983685222 0.9573196026328621 0.9573929705159704 0.9570034254143647 0.9576212536092398 0.9565234347516045 0.9572728103616812 0.9982335709805562 0.9578484707026502 0.9883875486381324 0.8740619770773639 0.957599566395664 0.997019756475537 0.9580177596034696 0.956707430945979 0.957899712942341 0.9566958665048543 0.9562598873711957 0.9576446933200893 0.9579446921958313 0.9890126247389186 0.9571387964302943 0.9571056209307933 0.9571731278815822 0.957108623101037 0.9562075305682091 0.9558592499383174 0.95628304784689 0.9562864258373206 0.9563427285782671 0.8773512277823353 0.9575121015549078 0.9988776139062837 0.9988972760630261 0.9575047388875395 0.9574905371900826 0.9575232046613256 0.9989773981461523 0.9575273083517023 0.9572687702902115 0.957290233702337 0.8767839342105264 0.9989073402417963 0.9989079524838013 0.9575006272793581 0.8799009619289341 0.8782293873257954 0.8771318443051201 0.8789099096225412 0.9590839199625556 0.9590038868013151 0.9571358652447891 0.9573595395992367 0.9570134579207923 0.9434951876267748 0.9576088770312879 0.957266698250729 0.957674483596598 0.9575937059538275 0.957332347509113 0.9573047919201753 0.9571885320197044 0.9572804679802955 0.9576569851485149 0.9576953400939897 0.957075544603253 0.9578358264976385 0.9577011831683169 0.9579290167570232 0.9579121671597635

GCA_015684535 0.8786998080660837 0.9882113512940178 0.9584624663779349 0.9590769292568624 0.956670916988417 0.988587770590772 0.886100019846192 0.9781491546569155 0.9882085872877666 0.9995782525388006 0.8814248319999999 0.9885967116538131 0.8813271958762887 0.9588209528069783 0.9692493772284289 0.8844137512537613 0.988192559080264 0.9561221828268209 0.9886610621326617 0.9562257727713177 0.9766082919540229 0.9566822395958623 0.9885880475098979 0.9568275928172773 0.9894391189710611 0.9563844527483124 0.988640739039666 0.9564300411323494 0.9887379380791913 0.9562107875150061 0.9900506134840872 0.9560368911416849 0.9886458484658737 0.9884124711473184 0.9728055433848798 0.9569001548886737 0.9720988708616533 0.9567589682539683 0.9886298113998323 0.9562005360134005 0.9721333296919379 0.9563909978412088 0.9697171599718111 0.955990724880383 0.9885761743138487 0.9560461980293199 0.9900291276419395 0.9562337563085798 0.9785212665079022 0.9560174466554783 0.9998014975294564 0.9568612043269231 0.9721085499017252 0.9568060659287777 0.9885894304193616 0.9562407297877414 0.9892994377254403 0.9561955622296973 0.9999517931165621 0.9563520612147299 0.8788456704277091 0.9563092623537836 0.9781159746780179 0.9560096071856288 0.9720930782646608 0.9561104265516416 0.9872408062658374 0.9563326364719904 0.9721871972996516 0.9563084710645393 0.9870206064690028 0.9562656199138344 0.9720463081967213 0.9562812439961575 0.9886438184105683 0.9567732508409419 0.9886150836820085 0.9562532231800767 0.9999112407652966 0.9560130642069957 1.0 0.9576857200629073 0.987271421491327 0.9872437864293662 0.9886616708701135 0.9872437864293662 0.9891348598930482 0.9877104381114419 0.9886437064832186 0.9880734165067181 0.9886035985647954 0.9559975710532602 0.9997516338995993 0.9716253333333333 0.9721181087573707 0.9698345435041716 0.988584189359028 0.969561872500588 0.9697024632610217 0.9702372384059661 0.9892287231270358 0.9728055805003207 0.9887157609391676 0.9887866856540085 0.9886598145780053 0.9783214781297135 0.9565619474313023 0.9886933451585977 0.9590169966703662 0.8850687952369833 0.9775483114610674 0.9579015527230591 0.972101742290749 0.9891545208070619 0.988318551342139 0.988270406037415 0.9884465243399341 0.9775383663584325 0.988834514863749 0.958810425531915 0.9989584669672614 0.9989551531213192 0.9712851457823279 0.9989578892597684 0.9990182326402511 0.9982392982456141 0.9989458429606423 0.9989545495495495 0.998949318582338 0.8773971615837347 0.9985887839968467 0.9578789956729675 0.9578669143117593 0.9986992110453649 0.9987306898584906 0.9987286486486486 0.9582064058624576 0.9877131883741258 0.9886689100268576 0.9886710512189667 0.8846136195410808 0.9581942125451265 0.958253517599278 0.9987038708407167 0.8851586802523049 0.8841041565217391 0.8776067333160218 0.8839746234413965 0.9882096824724811 0.9880759201867969 0.9892466306191687 0.9869364392419175 0.9694310476190477 0.9493281467934651 0.9987321805691854 0.9987935739575139 0.998762250835463 0.9988782589899785 0.9988353664302602 0.9988125971208835 0.976352175958598 0.9762904955378112 0.9764307100869973 0.9764012514702424 0.9763744971804512 0.9762448284023668 0.9761810188679245 0.9878536387377583 0.9877859877488514

GCA_002334745 0.8753896105841239 0.9594826614209673 0.9985467145270271 0.9979422336409398 0.9976461398381296 0.9589845886739689 0.882686145380087 0.9592602046850125 0.9584019657116017 0.9592120369104579 0.8766657634512844 0.9593278771205869 0.8766150461613218 0.9978288734762505 0.9561744134215039 0.883420548049962 0.9593692264547792 0.9975723904293382 0.9596053107734807 0.9975977595505618 0.9576191891235736 0.998502410336378 0.9586151539855073 0.9984322785665991 0.9601939142526073 0.9973449944283487 0.9586588689936538 0.9977928827215756 0.9593609933623255 0.9976354021715048 0.9596376998597477 0.9970724372759858 0.9586759209629798 0.956684113753371 0.9587020789352916 0.998339993238675 0.9593122421318632 0.9984920311111111 0.9587294139110604 0.9977157909854176 0.95945379191548 0.9976968023899093 0.9570672924275627 0.9976465863365025 0.9587084171025699 0.9973495457567029 0.9598864642282035 0.9976246357174589 0.9596006119125174 0.9975731013079141 0.9585270657864786 0.9984392826910226 0.9592822584347029 0.9984229398663698 0.9587372426303855 0.997820806700463 0.9599402696998124 0.9976432178547634 0.9584667459410017 0.9977780509413069 0.8771953016550988 0.9977473736482013 0.9594353156069364 0.9975871870853605 0.9593359688502061 0.9976892297447282 0.9578498931411531 0.9981203144930725 0.9596552091954023 0.9980733772218565 0.9578018706081899 0.9977094613686535 0.9593432094517091 0.9977236321278012 0.9587900228154232 0.9985089452603471 0.9585916033659314 0.997736168472253 0.9585456550467076 0.9976856015037595 0.958530631773115 1.0 0.9577759334789998 0.9572888417540515 0.9586527116311081 0.9572888417540515 0.9598756549965222 0.95957630105553 0.9585870031688548 0.9593212343143965 0.9597284560185184 0.9977124757495591 0.9586091611276645 0.9584159119206825 0.9593658714121699 0.9571140876089063 0.958652552901024 0.9566379763342187 0.9570725859564165 0.957162693225191 0.9603816142557652 0.9588902224260489 0.9596162201286765 0.9594394747706422 0.9595944549001607 0.9595891545054437 0.9979706001765226 0.9586761116152449 0.9899987860525182 0.8800748499876022 0.9589967600459244 0.9971539263937282 0.9595505100857872 0.9592950244015802 0.9594881456491392 0.9595133123396313 0.9589856374002282 0.9589921160092808 0.960451134444191 0.9898765369305617 0.9593104468133364 0.959323710591971 0.9588609606081547 0.9593167445553539 0.9593159631985461 0.9588350547402748 0.9593395698437854 0.9593406361784016 0.9593874773550725 0.8766647127371273 0.9597378887884268 0.9983876547094609 0.9983943627965567 0.9599461301989151 0.9601123963963966 0.9599534079096046 0.9985567684954281 0.9602879116279069 0.9568796835443039 0.9569109686055001 0.8866529139234334 0.9985551474252492 0.9985449314499377 0.9600574189189189 0.8836808857001484 0.8833272679509632 0.8773137177783683 0.883896483796887 0.9611645534704952 0.9611431391659112 0.9601122381172127 0.9554389674042351 0.9547207160253288 0.9428173815461347 0.9601277703005832 0.9598078735891649 0.9601413303370787 0.9599621742066172 0.959715993669455 0.9598213843021941 0.9573699877390878 0.9573824050942935 0.9573737022526935 0.9573917732059759 0.9573641852487136 0.9572606927044952 0.957328788249694 0.959258360846315 0.9592343

GCA_015684575 0.8716979800367745 0.9883859533799535 0.9576936839542245 0.9584800146020929 0.9582988043478261 0.9890440636363637 0.8789691885377611 0.9779512336892053 0.9902580406654343 0.9888928298453138 0.8730701608848669 0.990264476168547 0.8730458385876421 0.9583354884287454 0.9726016484318015 0.879185931751533 0.988439218309859 0.9581383465346535 0.9888330011587485 0.9585449950248756 0.9770039390359166 0.9581242667649226 0.9884663361072583 0.9582267519245098 0.9891705773243682 0.9580921511484318 0.9888102088167052 0.9577860059612519 0.989186032923719 0.9580410273466373 0.9905860772075821 0.9579549802176064 0.9884767268100856 0.9904005529739779 0.9711544123957091 0.9582115593387615 0.9718469753384913 0.9580139391709591 0.9884854405952104 0.9580294783247613 0.9718595213923132 0.9580868662576687 0.9722429807926087 0.9580400171779141 0.9884837830319889 0.9579827649088221 0.9899587091615262 0.9579093236596162 0.9781934864603481 0.9580922126719057 0.9882884678335985 0.9581098649975455 0.9718363965267728 0.9581736126812485 0.9884389983807542 0.958159085995086 0.9896763483401547 0.9580617825443787 0.9887210310701956 0.9580776109467456 0.8796676691729323 0.9581343327598919 0.9781369601727447 0.9579820225545476 0.9717702963141411 0.958048781626136 0.9993752228047182 0.9580900343811395 0.9721627482567925 0.9579435786117243 0.9996816131739038 0.9580174730127577 0.9718248519417475 0.9581546556812591 0.9888115907498248 0.9581965431795879 0.9884468832675795 0.9580645731108931 0.9887676404494381 0.9583804502541758 0.9886607637286571 0.9582736276674025 1.0 0.9889330045662099 0.9884395753616425 0.9889330045662099 0.9895954927603924 0.9889816381304952 0.9885165210355987 0.9894730833901387 0.9885928402229448 0.9579522651663405 0.9888302205375602 0.9709027371208475 0.9717579319498069 0.9716250678623365 0.988329918661399 0.9706679239416407 0.9721460430263476 0.971637964028777 0.9907516747854325 0.9711364979708762 0.9903724205267938 0.9894748553719008 0.990342699456029 0.9782136363636363 0.9574065438342969 0.9888049721189591 0.9576352669208771 0.8757354821939174 0.9778179912557688 0.9581293176116408 0.9719371247254088 0.9890502905513613 0.9893932046864231 0.988449686256146 0.9889856650804442 0.9777402572268495 0.9886241915367484 0.9584033115626512 0.9881060556687034 0.9880973727087576 0.9702651977809937 0.9882140940963584 0.9881764130434781 0.9870064159702878 0.9878270889792231 0.9877945460704608 0.9878273255025977 0.8773481606708141 0.9885202141230067 0.9580443513513514 0.9580642131107293 0.9885196859353664 0.9885368382687928 0.9885505968109338 0.9579592168821663 0.9992603285870755 0.9882274988441979 0.9882335943635943 0.8783162673425826 0.9579611447400538 0.9570575200388634 0.9884752962625342 0.8802032383690538 0.8790150766790058 0.8789167596002103 0.8800865972778222 0.9906601777277841 0.9905755475439386 0.9911204630269523 0.9890722885230909 0.9718573945044162 0.95061235501765 0.9885670063839489 0.9888985095161661 0.9886665310786106 0.9885407574370709 0.9885376704805492 0.9889190029864462 0.9769111768970519 0.976319200863931 0.9769653918495297 0.9768248781076515 0.9768572957339119 0.9763939058709364 0.9768588821825206 0.9894071468144044 0.9893529515011547

GCA_002334825 0.8718461375387798 0.9884763416877443 0.9561282884386175 0.9569774355334754 0.9561685835556634 0.9887452220208427 0.8778248710449349 0.9758151141226817 0.9886696091738825 0.9877477200902935 0.8697641761868495 0.9892762147887324 0.869789373088685 0.957137 0.9702214410480349 0.8779801018220793 0.9884857501161171 0.9563318186191966 0.9886200641025642 0.9563961407766989 0.9764622439723084 0.9568269858070724 0.9888702724358975 0.9568056286407768 0.9891250763535401 0.9564996434594073 0.9890499518790101 0.9565692972711907 0.9887071361919573 0.9564022323353294 0.9897274029850747 0.9564418144379845 0.9889659642283881 0.9887562035982693 0.969784485065711 0.9569619268942144 0.9703869868995634 0.9569008355342136 0.9889761337946944 0.9563904393505253 0.9703661883952416 0.9563965476760901 0.9704000073242187 0.9563244898446834 0.9888680202345367 0.9562326067146283 0.9895902932484656 0.9563971432003845 0.9763154120213281 0.9562335510887772 0.9872768799646955 0.9569025966850828 0.9703050109090908 0.956876083714217 0.9889216150493232 0.9567525149700601 0.9881354765258216 0.9563697720182386 0.9878743274053807 0.9567511455331413 0.8771274819951986 0.9567889423307012 0.9755850913900913 0.9562695441527447 0.9703126674727932 0.9563771158449019 0.9877711745360582 0.9567510250896057 0.9701217297036858 0.9566899284180387 0.9880422569602921 0.9563611756272401 0.9703167282899537 0.9563229135743357 0.9890651676300578 0.956830146353167 0.9888497424103037 0.9563801674641149 0.9880194219390077 0.9567874522292994 0.9878712826984856 0.9568096263949539 0.988124296803653 1.0 0.9889250069316081 1.0 0.9889541987923828 0.9996055039385983 0.9889787377086668 0.9882678765545833 0.988544596402214 0.9562858605094026 0.988003823798627 0.9700167214285714 0.9704010084848484 0.9699375340136054 0.9890197310344827 0.9694834783659657 0.9703998835799175 0.9703929647397459 0.9893099953509995 0.9698436069474186 0.9891622735632184 0.9889975090334235 0.9893019972420133 0.9761728368964688 0.9572346655714621 0.9891171612015593 0.9560147812424462 0.8749359197324413 0.9767463860911272 0.9565384639804642 0.970331252758029 0.9884319391025641 0.9878439925198691 0.988532271461717 0.9990967505241091 0.9768703561046512 0.9881442999773087 0.9559879950799508 0.9878485010266941 0.9878868386213195 0.9699008353108686 0.9878353491556368 0.9879285737106344 0.9870739625292739 0.9877962121557022 0.9878261771402549 0.9877428038233954 0.8782880695605284 0.9878392049830125 0.9569337431498691 0.9569091305383518 0.9877829089261441 0.9877838989578613 0.9877975135869566 0.9569296831824678 0.9884304263746506 0.9880515564202333 0.9880506543125143 0.8785756141732284 0.9569375422719696 0.9568799595527004 0.9877510312783317 0.8795322695763801 0.8792766640584244 0.8768872882672883 0.8804667953769372 0.9892216425011329 0.9892590436165379 0.9891264328984158 0.9987628362512874 0.969931998544749 0.9506429344016024 0.9878144547301874 0.9878368607021517 0.9880193771234429 0.9878336613995486 0.9879453107985481 0.9878993390869861 0.9755318027698187 0.9754503793843953 0.9756418270838308 0.9757287452198853 0.9755677427821522 0.9755357306864387 0.9755662300167025 0.9883258230212466 0.9883751424567959

GCA_015684635 0.8767767492639843 0.9918502175277137 0.9588302228285585 0.9593598429084382 0.9571730931796348 0.9885338970106916 0.8848856751320091 0.976775562376672 0.987907190211346 0.9893239739550516 0.8804230357142858 0.9924272443601098 0.8802729556535871 0.959318527953111 0.9698056826923077 0.8848759283551967 0.9918318369932433 0.956606939117929 0.9920983754139073 0.956729091127675 0.9747531244153415 0.9570850000000001 0.9999447801774008 0.9570728488652825 0.992078454450262 0.9568714391762452 0.999961796511628 0.9569143001930503 0.992435223880597 0.9568562675888386 0.9898502971990593 0.9565552162421913 0.9999669791666668 0.9882439916935856 0.9717277277692812 0.9570805962971869 0.9721224568774878 0.9571068545497969 0.9999619383003493 0.9567921063880312 0.9720790404598718 0.9568037404761904 0.9695707181796634 0.956704034212402 0.999937756050339 0.9566621307563827 0.9901647763408946 0.9566758819311665 0.9774033163716814 0.9566150344827585 0.9886372179029208 0.9570970183814752 0.9721475011047284 0.957162485645933 0.9999462374517376 0.956780216409037 0.9882513582121935 0.9567441585103845 0.9889197061931937 0.9567893223574326 0.8798054758800522 0.9567521363853125 0.9767840331858405 0.9566993016627079 0.9720681376875553 0.9567774018558173 0.9870554466588511 0.9568134402470897 0.9719606965394852 0.9567630555555556 0.9873482878581173 0.9568062440645776 0.9720896059331415 0.9568028329759354 0.9999326834997065 0.9571378244274809 0.9999159299400039 0.9566826627078385 0.988912373843566 0.9567362021403091 0.9888432173913043 0.9582522514619883 0.9874165739685435 0.9889445434047351 1.0 0.9889445434047351 0.9920285365344468 0.9894646754938138 0.9999601020801234 0.9873343109079035 0.9922094911366007 0.9566937754618664 0.9890313076437144 0.9711638248536696 0.9721485439681838 0.968854504784689 0.9998800873616774 0.9690348368022705 0.9695200636342212 0.9696080131517144 0.9887515434155009 0.9717492289261599 0.9878106865930257 0.99202997909699 0.987799763864818 0.9775367388422449 0.9568955440169932 0.9999537558229815 0.9585974652622143 0.8830904448696484 0.9766675733634311 0.9582577424631924 0.9721493453976766 0.9889049434606357 0.9891140591690233 0.991828902208202 0.9902376300211416 0.9767463512283895 0.9881987280423282 0.9586352847380412 0.9882632823454429 0.9882430453287934 0.970369676040495 0.9882460757608001 0.9883289855380689 0.9876907976398601 0.9882612415110358 0.9882697071307301 0.9882496136701338 0.8780300509110396 0.9878997624190065 0.9584176459854014 0.9583973905109489 0.9880109686486486 0.9880661563307492 0.9880170875675676 0.9586792758542656 0.9879694335850734 0.9877211979881115 0.987733395752455 0.8838532343479336 0.9587065867450804 0.9583609914375845 0.9880989003880983 0.8842374198286415 0.8842408015932288 0.8771089196242173 0.8845672951231776 0.9879382281763183 0.9878568464370805 0.9891234660068847 0.9889225559322032 0.9694475765550239 0.9491053308641975 0.9881477304048234 0.9878698421621622 0.9882533146309883 0.9882878555987894 0.9877433419689119 0.9878947922077922 0.9752003337278108 0.9747387155963302 0.9752949302435565 0.9748474491725769 0.9751100898557579 0.9748958194774348 0.9751700923514091 0.9879767650297292 0.9878748155511375

GCA_002334845 0.8718461375387798 0.9884763416877443 0.9561282884386175 0.9569774355334754 0.9561685835556634 0.9887452220208427 0.8778248710449349 0.9758151141226817 0.9886696091738825 0.9877477200902935 0.8697641761868495 0.9892762147887324 0.869789373088685 0.957137 0.9702214410480349 0.8779801018220793 0.9884857501161171 0.9563318186191966 0.9886200641025642 0.9563961407766989 0.9764622439723084 0.9568269858070724 0.9888702724358975 0.9568056286407768 0.9891250763535401 0.9564996434594073 0.9890499518790101 0.9565692972711907 0.9887071361919573 0.9564022323353294 0.9897274029850747 0.9564418144379845 0.9889659642283881 0.9887562035982693 0.969784485065711 0.9569619268942144 0.9703869868995634 0.9569008355342136 0.9889761337946944 0.9563904393505253 0.9703661883952416 0.9563965476760901 0.9704000073242187 0.9563244898446834 0.9888680202345367 0.9562326067146283 0.9895902932484656 0.9563971432003845 0.9763154120213281 0.9562335510887772 0.9872768799646955 0.9569025966850828 0.9703050109090908 0.956876083714217 0.9889216150493232 0.9567525149700601 0.9881354765258216 0.9563697720182386 0.9878743274053807 0.9567511455331413 0.8771274819951986 0.9567889423307012 0.9755850913900913 0.9562695441527447 0.9703126674727932 0.9563771158449019 0.9877711745360582 0.9567510250896057 0.9701217297036858 0.9566899284180387 0.9880422569602921 0.9563611756272401 0.9703167282899537 0.9563229135743357 0.9890651676300578 0.956830146353167 0.9888497424103037 0.9563801674641149 0.9880194219390077 0.9567874522292994 0.9878712826984856 0.9568096263949539 0.988124296803653 1.0 0.9889250069316081 1.0 0.9889541987923828 0.9996055039385983 0.9889787377086668 0.9882678765545833 0.988544596402214 0.9562858605094026 0.988003823798627 0.9700167214285714 0.9704010084848484 0.9699375340136054 0.9890197310344827 0.9694834783659657 0.9703998835799175 0.9703929647397459 0.9893099953509995 0.9698436069474186 0.9891622735632184 0.9889975090334235 0.9893019972420133 0.9761728368964688 0.9572346655714621 0.9891171612015593 0.9560147812424462 0.8749359197324413 0.9767463860911272 0.9565384639804642 0.970331252758029 0.9884319391025641 0.9878439925198691 0.988532271461717 0.9990967505241091 0.9768703561046512 0.9881442999773087 0.9559879950799508 0.9878485010266941 0.9878868386213195 0.9699008353108686 0.9878353491556368 0.9879285737106344 0.9870739625292739 0.9877962121557022 0.9878261771402549 0.9877428038233954 0.8782880695605284 0.9878392049830125 0.9569337431498691 0.9569091305383518 0.9877829089261441 0.9877838989578613 0.9877975135869566 0.9569296831824678 0.9884304263746506 0.9880515564202333 0.9880506543125143 0.8785756141732284 0.9569375422719696 0.9568799595527004 0.9877510312783317 0.8795322695763801 0.8792766640584244 0.8768872882672883 0.8804667953769372 0.9892216425011329 0.9892590436165379 0.9891264328984158 0.9987628362512874 0.969931998544749 0.9506429344016024 0.9878144547301874 0.9878368607021517 0.9880193771234429 0.9878336613995486 0.9879453107985481 0.9878993390869861 0.9755318027698187 0.9754503793843953 0.9756418270838308 0.9757287452198853 0.9755677427821522 0.9755357306864387 0.9755662300167025 0.9883258230212466 0.9883751424567959

GCA_015684655 0.8763334485330683 0.9987296972775296 0.9594135971553108 0.9598839663789187 0.9576831080426357 0.9896267406105864 0.8855623721518987 0.9782476808368573 0.9890309424558225 0.9896957804825967 0.8801170044361429 0.998969204031263 0.8800439012403465 0.9596903420752565 0.9705296591454634 0.8858864182509507 0.9987186796714581 0.9573334346135148 0.9990615387705907 0.9574281401025141 0.9761247097844112 0.9573466706846674 0.9924463723249533 0.9574053447015833 0.9999322597507037 0.9572543803056028 0.992802039281237 0.9571477278250303 0.9996150374115268 0.9573105839768341 0.9905976470588235 0.9571548530483363 0.9924301227121466 0.9891487277764951 0.973416407874364 0.9574074006782947 0.9730704809619237 0.9572820337349397 0.9924615914786968 0.9571802093358999 0.9731137917222964 0.9572599108433736 0.9706426649264356 0.9571962123765952 0.9924256690287621 0.9571607387735394 0.9909730718113612 0.9571664002897151 0.9787951918330715 0.9572451460294472 0.9887666573816155 0.9573127472262423 0.97306754503002 0.9574686065573771 0.9924826796842543 0.9572321691973968 0.989320296572935 0.957290360077332 0.9893609890109889 0.9573547064508335 0.8802937480397282 0.9572344471211757 0.9782704907013219 0.9571788174373796 0.9730085958675849 0.9571827253012049 0.9890648535663675 0.9572303631553633 0.9733407905935049 0.9571921303197884 0.9887407695820963 0.9571493121693122 0.9730360558659219 0.9573117486734202 0.9928220113732098 0.9573645568400769 0.9925388643467389 0.9571939720751083 0.989381108966109 0.9571964626506025 0.989338224480969 0.9593061999542438 0.9887928201603665 0.9889443520838077 0.9924114690451207 0.9889443520838077 1.0 0.9894644497921681 0.9924243966770508 0.9885755009909711 0.9990283995943204 0.9571189687500001 0.989344561667383 0.9723396717925178 0.9731051492869875 0.9702174777590767 0.992342531328321 0.9705901045627378 0.9705971542803385 0.971438754725898 0.989662808738297 0.9732537234513275 0.9897885448577681 0.9986568773006136 0.9898219479667687 0.978762904091214 0.9573731418269231 0.9927978707383394 0.9597885445477217 0.8838743480345158 0.9777314256433615 0.959216494918459 0.9732235233160623 0.9899677907986112 0.9895798966124065 0.9987513318284426 0.9901645168156662 0.977706896472744 0.9898167758620692 0.9600074517019319 0.9890883540239727 0.9890138580313104 0.9721791126824818 0.9889865859408487 0.9890954187192118 0.9884009036808463 0.9891132563115106 0.9890944544287549 0.9890654384088966 0.8787533082099597 0.9883963808900522 0.9588900946882217 0.9588623515823517 0.9885287745740499 0.9885748651587648 0.9885309630923783 0.9591270032051281 0.9887960413885181 0.9883214630779847 0.988406190257353 0.8854870472636814 0.9591526425463706 0.9591330126002291 0.9886243522752014 0.8853460044258666 0.8847735232120453 0.8778550091887636 0.8850468730886851 0.9888566086578204 0.9888185792230467 0.9900838466579294 0.9886354291553134 0.9700228829045443 0.9499199825740603 0.9887215771155101 0.9887353235679931 0.9888110235191638 0.988723680191972 0.9886626844755858 0.9887401379461355 0.9765443007915567 0.9759494686680963 0.9766134587727708 0.9766204120747485 0.976596941514861 0.9761729508196721 0.9765853205282112 0.9891111361129494 0.9889392479539925

GCA_002334865 0.8761934839031694 0.9884045566286217 0.957672935821575 0.9579573398014439 0.9556164106280194 0.9882234401756311 0.8819245711376941 0.9763522872579918 0.9881412949152543 0.9878080185225071 0.8758208652687395 0.9891533526135391 0.8757279134429401 0.9579792990018149 0.9692919131714497 0.8834490601981204 0.9884056162558296 0.9558283321334292 0.9886826847398339 0.956028117902875 0.9757245330132054 0.9561368082553395 0.9891889833843464 0.9561710887487874 0.989221046588651 0.9560475776547075 0.9893601051018175 0.9557249119845673 0.9887894681982557 0.9557583544606553 0.9897466556583002 0.9557922930702598 0.9892111916757941 0.9883984322227294 0.9707098135939987 0.9562253560222062 0.9709609912603496 0.956287855773838 0.9892246512651265 0.9558161051629788 0.9709932137931035 0.9559169232608176 0.9692568617021275 0.955799564181948 0.9892348704435661 0.9557481013868961 0.9895133362237155 0.9558647268806901 0.9771122781609196 0.9557570918610186 0.9874467556120289 0.956251304764185 0.9708579494252874 0.9563713114361064 0.9892058318739055 0.9559559699355762 0.9879546953405017 0.9559410541447052 0.9880504049026045 0.9560154789915966 0.8760463064259161 0.9559292316869482 0.9761300934153566 0.9557583555133081 0.9708636955524989 0.955890780057252 0.9877504908267811 0.9559198139312978 0.9705044393801276 0.9558651940938319 0.9875890973654066 0.9558598712446351 0.97102463099631 0.9558358367249221 0.9894009916077738 0.9563171120689655 0.9892059956043956 0.9559167541766109 0.9880205528950805 0.9558533999999999 0.9880069435874834 0.9582474078341013 0.9879087376180603 0.9992572350693748 0.9892141264927024 0.9992572350693748 0.9890054742907802 1.0 0.989208512450852 0.9877896515218351 0.9886292739273929 0.9557574857685011 0.9879648478879405 0.9704233499095841 0.9709679007808911 0.9695234925229136 0.9892418839305342 0.9687179249819667 0.9692913301320527 0.9694464363982849 0.9893348530066816 0.9707768523550725 0.9888450427913101 0.9892403981623278 0.9889123728070176 0.9770554348322301 0.9559210277711845 0.9894332114711034 0.9578356018202503 0.880949269183922 0.9773100181570585 0.9573575828047922 0.9710654365541327 0.9883320126886895 0.9873319715492332 0.9884705132743363 0.9986304805952142 0.9773589201555707 0.9879471663066955 0.9574947889951038 0.9875704259017818 0.9875534 0.970665484160863 0.9876255009780484 0.9876182391304349 0.9870133214603739 0.98765950595883 0.9876365641247833 0.987621891657638 0.8776460675965666 0.9878390115142299 0.9580820296803653 0.9580970584208126 0.9879328025200955 0.98796590426684 0.9879330737527116 0.9584439171758316 0.988581685669573 0.9874986251145739 0.987498636884307 0.8840873753411066 0.9583701697985058 0.9582885804844918 0.9879257096424702 0.8847880345414741 0.8837693333333333 0.8757866081718179 0.8844924250818434 0.9889987753341957 0.9889997361591695 0.9890746780261119 0.9984626004533279 0.969292142338417 0.9498953608760577 0.9880478528269313 0.9880588257000217 0.9883145041143352 0.9881216860715826 0.9879916800695502 0.9880426985854189 0.9749803112378237 0.9748546783764539 0.9751877727596862 0.9752233943862987 0.975073177925785 0.9748170176274416 0.9749525071157494 0.9881434129464285 0.9880676713552133

GCA_015684695 0.8770729326450344 0.9916935861344538 0.9591461994054424 0.9595361479303326 0.9575193423597679 0.988682144895719 0.8845123505775991 0.9775725160291842 0.9878569374999999 0.9892859814267624 0.8800697554157931 0.9923495940803383 0.8799100723791735 0.9595239391185826 0.9700291403804479 0.8845440945674043 0.9916656954767468 0.9571550253317251 0.9918330812383128 0.9572847783967063 0.9748620859760396 0.9574219294456444 0.9998997010869566 0.9575405327171004 0.9920388584667229 0.9573869891435465 0.9999322597656252 0.9571632509689922 0.9922374557936343 0.9573135110470702 0.9900659689253345 0.9571344891041161 0.999988583365721 0.9884284848484848 0.9718246419916281 0.9575114140681653 0.9721937927967986 0.9574603647708183 0.9999637321603129 0.9572483042751374 0.9722715915649278 0.9572964011516315 0.9696599501069139 0.9571502482692767 0.999915015618899 0.9571713966882649 0.9901542449414271 0.9572040153883146 0.9779712181493071 0.9571492845178273 0.9884049841605069 0.9574338494830488 0.9722575438986443 0.9576194492544492 0.9998821318809572 0.9571208141762452 0.9883683100436682 0.9573227023780928 0.988652214893617 0.9572670413064363 0.8799697784150157 0.9570604865771813 0.9774506766080571 0.9572187541846008 0.972123017489484 0.9572682000957395 0.9874936043233083 0.9571763629842182 0.9721870773000668 0.957063895793499 0.9877642321755028 0.9572169488283119 0.9722385886019591 0.9572209705248023 0.999920505250644 0.9575664881095364 0.9999001348182884 0.9571661267942584 0.9886836671607029 0.9572067360612587 0.9886131084543126 0.9585842025373812 0.9877100320806599 0.9888780872710831 0.9999169649881984 0.9888780872710831 0.9920336281322384 0.9892782496194826 1.0 0.9874579651035985 0.9919205204617 0.9571130345649583 0.9887185605257579 0.9708643226822683 0.9721912061306085 0.9692129547088426 0.9997267346138771 0.9691687022538552 0.9696662435110902 0.9697913290990355 0.9885789593062042 0.971816376398333 0.987941361757668 0.9918867742611611 0.987872990004346 0.978145352770977 0.9572137743467934 0.9999261759530793 0.9585940672990063 0.8834897673860911 0.977024860259032 0.9584320690465008 0.972311098356966 0.9888289872872226 0.9893016757821046 0.9916952964845405 0.9902303290171939 0.977010674002751 0.9886440710789529 0.9586744612216883 0.9881506717850287 0.9880845953448646 0.9704817004504505 0.9880886995515694 0.9881358591248666 0.9875083633172445 0.98814467930961 0.9881482310315431 0.9881345898146175 0.8776309657570893 0.98770210320902 0.9586023068103647 0.9586270472260432 0.9877634049479167 0.9878125632159068 0.9877734114075036 0.9588517045454547 0.9882701182244031 0.9877247760511882 0.9877371376398082 0.8838090960731044 0.958930930602958 0.958613090702948 0.9877970140540541 0.8842368899755501 0.883977963744723 0.8772432629658587 0.8842084026203072 0.9880969090514435 0.9881125809958686 0.989492667962843 0.9888653419384059 0.9694020445295667 0.9493773720814703 0.987936914273375 0.9876388559046588 0.9880239787970577 0.9880664674030756 0.9874306060606061 0.9876691039270994 0.9754056460807601 0.9747905997638725 0.9754711488250652 0.9751078123515439 0.9753256942796107 0.9750217178937337 0.9752527916369685 0.9882882518256252 0.9881415103127078

GCA_002850625 0.8774375673633845 0.9876465985790409 0.9590181123648333 0.9591730396270397 0.9573834536968804 0.9897589256012588 0.883128759350013 0.9779594224235559 0.9988056853002071 0.9890694562131469 0.8787579246194734 0.989033350527024 0.8786641008240426 0.9589986570296105 0.9708113701981894 0.8837423937246963 0.9877183637182085 0.9574252826983742 0.9878803716736309 0.9575520947265624 0.9764396290944122 0.9575642155425221 0.9882097121346324 0.9577198788328387 0.9890345041601079 0.9572562683643487 0.9886329537843268 0.9573288047512992 0.9889478458805241 0.9569908613958028 0.9910470691572159 0.9574186071949441 0.9882461263858093 0.990703774291735 0.9718798746581585 0.9575380409068508 0.9725924473257699 0.9575200609904855 0.9881961291043109 0.9572794885954382 0.9725920703215359 0.9569410867979578 0.9704140860990862 0.957279111431316 0.9881943429844099 0.9574182897919691 0.9910568347710685 0.9568745919610233 0.9781440448140449 0.9573150228420293 0.9886675764913053 0.9575786274987812 0.9725484234858992 0.9576466805283758 0.9882324390243902 0.9572195994173344 0.9898066060337177 0.9572219738435458 0.9890029317980512 0.9572349012435991 0.8789519407806192 0.9571556424581006 0.9781267595341402 0.9573256353591161 0.9725295808383233 0.9573254111405836 0.9892177181366754 0.9571910528869481 0.9722446885020612 0.9572024382566585 0.9894963493150685 0.9570095702840494 0.9724323615295482 0.9570476164918273 0.9887842487629328 0.9576070470616924 0.9882810623608019 0.9570409387159535 0.9890337607592141 0.9563663558311469 0.9889669266770672 0.9594950796950796 0.989669725211816 0.9884568847566576 0.9882442713004482 0.9884568847566576 0.988759719290366 0.9888956164079824 0.9881697521575571 1.0 0.9880114955555555 0.9563096000952155 0.9891321660008877 0.973072252028855 0.9726020180096976 0.9707659462010382 0.9882804507922339 0.9697004975241689 0.9704382238734421 0.9713984577114428 0.9900801587661602 0.9721853513759381 0.9986055032336298 0.9883140593617498 0.9986439789941426 0.9779078812199036 0.9569080569066148 0.9887583188211655 0.9597908681890119 0.8826492691929134 0.9772629846582986 0.9574070596727623 0.972534191142191 0.9897431732265957 0.9898624432194045 0.9877853042892433 0.9893543725490196 0.9773342014051524 0.9954982074866311 0.9580694340060311 0.9885472154382905 0.9886325398210779 0.9719458344733242 0.988520026166594 0.9886620987920621 0.9879332928981033 0.9886699311531841 0.98863080017208 0.9886918119216698 0.8776813439267439 0.9885371952846974 0.9585695419309372 0.9585141886171213 0.9886003824772072 0.9886785333925007 0.9886442666369909 0.9589467331323905 0.9903692585975883 0.9892854802259886 0.989279894117647 0.8845460983606558 0.9589988615241636 0.9589291774791474 0.9887316381798003 0.8842600551654966 0.8842092698892247 0.8775483796664019 0.8841637943444731 0.9952762202699807 0.9952623963925276 0.9908515622241837 0.9882773826425464 0.9702615059509352 0.9497645561685054 0.9887251062887512 0.9886782889480692 0.9887563930789708 0.9887941156914895 0.9886414758835297 0.9887138687430479 0.9768391860186738 0.9762541983824927 0.9768770303900454 0.9770614555901365 0.976831719348659 0.976867809937289 0.9767455273857927 0.9901016531105736 0.9900908183018038

GCA_015684735 0.877571937147066 0.9995859413343559 0.9576800997958721 0.9592180690595185 0.9563051101961735 0.9881196011333914 0.8853903116360894 0.9776944180939832 0.9877640416480071 0.9887565131578948 0.8808122370234267 0.9985436412371135 0.8808349170888989 0.9590791468978103 0.970170179090029 0.8851757391523714 0.9995494330097087 0.9563849015795869 0.9998474923488906 0.9567492642385726 0.975151080953505 0.9562561918396564 0.9917054886434673 0.9563198770491803 0.998028745881384 0.9559034552845529 0.9922366792294809 0.9555383081353491 0.9987211126760563 0.9561211761893378 0.9904466428106703 0.9557606329417406 0.99170013767209 0.988936896230805 0.9718779699411892 0.9567674561403509 0.972546299403842 0.9562505245588936 0.9916416281997482 0.9559755066603235 0.9725497883131203 0.9560058812306226 0.9696165246212123 0.9558761640903687 0.9917193447770568 0.9559178008595989 0.9896774509803922 0.956097088607595 0.9779729031541536 0.9559875952380954 0.9882121151385929 0.9561687574528978 0.9723744559699846 0.9564687082139446 0.9917525641025641 0.9558559633682208 0.9890700373708506 0.9560517359121299 0.9886455906357388 0.955899274636125 0.8801596746707979 0.9558103192756731 0.97693802518224 0.9558929180562172 0.9724287161418189 0.9559220004768719 0.9873295614241925 0.9563423421686749 0.9723787050680955 0.9563106982903925 0.9865069957274567 0.9560623721151559 0.9725287729789591 0.9559823219814242 0.992244355077053 0.9563165919923737 0.9916573010236056 0.956036191383004 0.9886505389221556 0.9559310311237823 0.9884872264191669 0.9585375051831375 0.9872649747822101 0.9883968845019286 0.9916013825757577 0.9883968845019286 0.9981058246474557 0.9884988951841359 0.9916598248905567 0.9870028047182176 1.0 0.9558115618324236 0.9886198714377545 0.971191979498861 0.9725695658880565 0.969880348668281 0.9914678947368422 0.9693295286836466 0.9695181833137485 0.9695476057984569 0.9882821589138661 0.9719847671595675 0.988224289785182 0.9976291889683352 0.9876201992635912 0.9779229514348786 0.9563333797407586 0.9922700377121308 0.9583853324256865 0.8836495628152774 0.976836049270073 0.958186587038789 0.9726428099910792 0.9889156514273449 0.988545504587156 0.9995507722082445 0.9892058993553753 0.9768079053743685 0.9892228705882353 0.9583277020492748 0.987691112065264 0.987585604726101 0.9709923988108851 0.9875678286082474 0.9876614672395273 0.9869622604856513 0.9876691550503967 0.9876599228130359 0.9876726781115881 0.8784583107928929 0.9871725098468273 0.9577544966442954 0.9577282885595182 0.9872060906701708 0.987288595113438 0.987236934706398 0.9580316819642037 0.9870798134162594 0.9877999397869384 0.9878131852879944 0.8853177543515568 0.9580812350780532 0.9580666322314051 0.9872724836316019 0.8849053210116731 0.8842064187671576 0.877464470954357 0.8845768096446702 0.988109615636602 0.9880764798598949 0.9891517672507031 0.9882440656851642 0.9698212684580005 0.9496080064150013 0.9875382969432316 0.9871755519125683 0.9876475431693991 0.9876003127734033 0.987172422319475 0.9871954026258206 0.975312230952381 0.9747367430867406 0.975475272294887 0.9754867863085334 0.9753547680228409 0.9750782129186603 0.9753257632769706 0.9882445424255886 0.9880626503340757

GCA_002923255 0.8705345933140299 0.9566031404958677 0.9976258932059908 0.9988998500651891 0.9988816537353137 0.9578217338709678 0.8769124495912806 0.9579327361641222 0.9583703202328968 0.9574717862407863 0.8708927734778122 0.9582707264444977 0.8707320206985769 0.9989499348392703 0.9578177413706855 0.8765890050815724 0.9565294217103651 0.9993872827903091 0.9566998670534202 0.9992352167445815 0.9570893092428712 0.9992826927116153 0.957379605166951 0.9992194815129621 0.9577714963144963 0.9993465543071162 0.9575980805105547 0.9987679910714286 0.9581771706730768 0.9991278878504674 0.9581481886227544 0.9992311051100956 0.9574158790538893 0.9590860038938915 0.9563284809197652 0.9987548771104937 0.9577081869758263 0.9993583023983316 0.9575010196078432 0.999592386914378 0.957751575443787 0.999372224515064 0.9573936747727831 0.9995918824479902 0.9574290811075717 0.9992608504577822 0.9572208434613515 0.9992575635461873 0.9574960751617719 0.999466107748184 0.9574273532963054 0.9992773874816832 0.9576749839862035 0.9992747440201427 0.9574441043139166 0.9988279022147931 0.9576965053629334 0.9994673616236164 0.9575234640198511 0.9988889301540411 0.8759179420445398 0.9989505557894738 0.9568429879990203 0.9995275777777778 0.9576007820954254 0.9995612266069976 0.9577829387755102 0.9987815895287959 0.9573574851924974 0.9987506832427916 0.9573262147585193 0.9994038378153012 0.9576284796044499 0.9993056160938811 0.9576975673671199 0.9993124281217209 0.9574650451991203 0.9993687137867271 0.9573450222551928 0.9997860762779552 0.9574810186799502 0.9984872901023891 0.9578446676587301 0.9577884266409267 0.9575091188776764 0.9577884266409267 0.9577452292899409 0.957728849428363 0.9574171863581 0.9562805441849267 0.9566621563865956 1.0 0.9576442353525323 0.9552362914381111 0.9577528388368655 0.9556639062116378 0.9575389269127352 0.9553757510414114 0.9574101684570313 0.9572167988394584 0.9573734455640207 0.9572654860252289 0.9574393070026654 0.9573862075750124 0.9568399760364246 0.9573481311637082 0.9986371547314579 0.9576476428746628 0.9884539465807046 0.8740988124014715 0.9581477235367372 0.9967051257511684 0.9577299651307597 0.9565745977295163 0.9580804204204203 0.9566554968184043 0.9566684990300678 0.9581598619824342 0.9581416413894326 0.9892306884480746 0.9574904362661468 0.9575121009017791 0.9572175494264097 0.9574863784705309 0.956556503513448 0.9561617244821564 0.956618749395259 0.9566182240503267 0.9566270989595935 0.8772686314921683 0.9576091607361964 0.9991346282608696 0.9991168585704975 0.9575760681873928 0.9576040029433408 0.9576189436274509 0.9991416282608695 0.9577444738796733 0.9575992944099377 0.9576538446264582 0.8768954889006342 0.9991199499782515 0.9990672875461857 0.9576446038753984 0.8799625575447569 0.8766489316239315 0.8761590374900292 0.877430960960961 0.9593015717324509 0.9591954030346136 0.9572498704473235 0.9578493508687259 0.9569226462534229 0.9442614847161572 0.957780338401177 0.957554862475442 0.9578335772158114 0.9576839204126749 0.9575859857598822 0.9575444092360599 0.9571162105785945 0.9573054990069513 0.9576812546769768 0.9577160667996013 0.957078315554453 0.957998992481203 0.9576689742949838 0.9580424993770247 0.9580418664340893

GCA_015684755 0.8776208837890626 0.988154054916986 0.9582984720951511 0.9589071092173134 0.9569200096525097 0.988566184808483 0.8858489296407186 0.9778143290975662 0.9883716985277962 0.9997156443249472 0.8809012628925053 0.9888248575101778 0.8809133386874711 0.9586966486608148 0.969391977709272 0.88503777693857 0.9881306638115632 0.9566198140096619 0.9885289223321406 0.9567994764905476 0.9763551206619169 0.9570299062725306 0.988952870603015 0.957025804416404 0.9894674064516129 0.9568952372922188 0.9889717972350233 0.9568164697336562 0.9888109073644229 0.9568045094746943 0.9901375457028787 0.9569324818577648 0.9889496225623821 0.988629947845805 0.9726867407727174 0.9571680174081239 0.9720230074692444 0.9570791064136441 0.9890103516529796 0.9566764470531863 0.9720467828183214 0.956775966930266 0.970143134781593 0.9565849832295161 0.9889527610842614 0.9566153618658332 0.9901803742203743 0.9565822187499999 0.9781900673620166 0.9565427338129496 0.9999908070978822 0.9571368309689829 0.9720753676148798 0.9571778327727054 0.9889475020938023 0.9565714872284555 0.9889650784896055 0.9565653668510946 0.9997542280330706 0.9566018157894738 0.8792630578080042 0.9565743140535372 0.978023761487965 0.9565997722914671 0.9720420327868853 0.9566298295318129 0.9873630313508529 0.9567069777989974 0.9721415066695823 0.9566567318702289 0.9873980035890534 0.9566866730447261 0.9719009491451118 0.9566119166267371 0.9890338994507816 0.9570958083113139 0.9889770260613703 0.9566917189370362 0.9998363499043977 0.9566114254911356 0.9996505141090066 0.9577592025230908 0.9877008496732026 0.9873046011173184 0.9889516972767575 0.9873046011173184 0.9890294799914402 0.9878539768339768 0.9889344093003771 0.9882464681857266 0.988442686029256 0.9565131794258374 1.0 0.9713638981173865 0.9720501553270618 0.9698833611044989 0.9889402512138484 0.9698242332155477 0.970095115196649 0.9704207978599674 0.9891899542483661 0.9726944365591398 0.9890861984536083 0.9886914321980115 0.9890949924876583 0.9781075324394465 0.9568357628742514 0.9890141748794297 0.9589899306797854 0.8848879461988304 0.9773524200308847 0.9581449895227009 0.9720056324635117 0.9893925586804823 0.988526041087096 0.9881798504912432 0.9881195571192053 0.9773227727171739 0.9888560265450022 0.9591800797448166 0.9989426658791101 0.9989428242018132 0.9711617629928315 0.9989418430908732 0.99902135199054 0.9980977764277036 0.9988768339236522 0.998885531286895 0.9988788412354909 0.8777140209227469 0.9986628712675498 0.9580099131031328 0.9579682513160905 0.9988411249752426 0.9988434194438968 0.9988407801980198 0.9582147780797102 0.9877926206140352 0.9888476928251122 0.9888513514119229 0.8843105057981743 0.9582526903898458 0.9583221219678079 0.9987625958119322 0.8854074975657256 0.8839774489287494 0.8775153755868544 0.8846411391129033 0.9885027663193706 0.9884332111655658 0.9891590738769428 0.986787364548495 0.9697576508466493 0.9498756381418094 0.9988163620248177 0.9986057496044305 0.9988345128205128 0.9989475996055227 0.9985747705696204 0.9985828548802218 0.9765455419415646 0.9765108509637989 0.9765944057425278 0.9765639905882353 0.976524945856874 0.9763066863905325 0.976450141509434 0.9879523201399519 0.9878386805708013

GCA_003261215 0.8753005843030452 0.9716993553604436 0.9589181917616942 0.9584530652920964 0.9570632729054386 0.9693033570608495 0.881672914375491 0.9714314974336087 0.9708039215236349 0.9722766704263479 0.8760300720288116 0.9720400683060111 0.8760078815599422 0.9582332067703568 0.9920159907834102 0.8852728734177217 0.9716683403558052 0.9561737356046065 0.971857068014706 0.9562157094430992 0.9709385652383246 0.95617154784689 0.9709418815489751 0.9562472203389829 0.9721268049695264 0.9558418809980808 0.9712582489112995 0.9570342365853659 0.9719423722709881 0.9556614879388583 0.9735534827264241 0.9557541835260115 0.9712653617216117 0.9709990055116223 0.9902714044068551 0.9562919415600096 0.9914183037085802 0.9560258666029166 0.9713102003684938 0.955645351441506 0.9913421209465381 0.9558946065534561 0.9907002069767441 0.9556281177031214 0.9710690838295923 0.9557555632623773 0.9722963860133428 0.9555537520898019 0.9728055804292638 0.955554073897497 0.9719733023049645 0.9563835928143714 0.9913801487314087 0.9564465978886756 0.9709628294573643 0.9557412642314991 0.9726914658210006 0.9560150944750061 0.9722328666209106 0.9558166348903718 0.8754713445602781 0.9559216051440818 0.9724948496583145 0.9556051920782628 0.9913250556404103 0.9558801670644392 0.9704081179705074 0.9556403910879356 0.9901549123974274 0.9555100993612492 0.9707461783284742 0.9567729290012034 0.9913008809993425 0.9568902948996858 0.9710672205646087 0.9561748529060035 0.9710876392390557 0.9555668712824174 0.972253301457195 0.9556456479275844 0.9721431841138659 0.9585390711785298 0.971065216755967 0.9708634668246446 0.9713451872399445 0.9708634668246446 0.9722241607732203 0.9718694439380127 0.9712653450639853 0.9729497850192351 0.9720425834879406 0.9554655613577023 0.9723097252747251 1.0 0.9914208069101247 0.9909547638072855 0.9712380041341295 0.9913512766945219 0.9907084723827132 0.9907579133590383 0.9722581785799098 0.9903250388629802 0.9719000532161036 0.971401382636656 0.9719520240573676 0.9729046510560149 0.9551807181796634 0.97124427720504 0.9577955167724389 0.879037173081825 0.972112881630762 0.9572009010727058 0.9914734746136865 0.9715875826446281 0.972450209205021 0.9716989570695286 0.9720553567331958 0.9721045825559701 0.9721354516640254 0.9586450743801653 0.9714308521303258 0.9714040318906606 0.9909730035933207 0.9714076735715911 0.9713282604735884 0.97065321612752 0.9713957253121452 0.9713984199772986 0.9712663086642599 0.8766545598255655 0.9714054530855701 0.9582239346919871 0.9581649489084998 0.9712013467244921 0.9713475596455351 0.9712353377453217 0.9588075827966881 0.9724190052231719 0.970625963459196 0.9706668240447798 0.8824241564729868 0.9586752134006424 0.9580007985398129 0.9716127295568753 0.884728757048296 0.8823931946206547 0.8754267203867849 0.8839883484573503 0.970599110707804 0.9706426410373067 0.9733770122656793 0.9701045396750649 0.9905943035001173 0.9471725144146401 0.9714027802995915 0.9713793446379468 0.9714992473851751 0.971569075802413 0.9710363411845415 0.9713474500344592 0.971616336237344 0.9715190531015039 0.9716388535781545 0.9714669033018868 0.9715143589743589 0.9715836275437768 0.9713468468468469 0.9726008877103989 0.9725949181461839

GCA_015684775 0.8770827063433749 0.9729373959255979 0.9593955635269613 0.9598264238711142 0.9578013369245838 0.9717801372369625 0.8856200305033046 0.9741763670085096 0.9716164343786295 0.9731914065255731 0.8806928018539978 0.9728082970608033 0.8807013395348837 0.9593936493860846 0.9914505366847827 0.8850083692307692 0.9729734444941808 0.9576288427734374 0.9733117010535557 0.9577264210267746 0.9714754319831421 0.957643054811206 0.9726583285302595 0.9577353901058331 0.973142837807354 0.9574978967962826 0.9725917972043489 0.9576327388847949 0.972932787464136 0.957575626523647 0.9740664840389406 0.9574103407697966 0.9726805153265216 0.9704710178179156 0.9905774569707402 0.9576976620825148 0.9999785962175862 0.9576761319376825 0.9727155260811414 0.9575835255633632 0.999940979401477 0.9576165580150814 0.9981938579117331 0.9575073498062016 0.9728023830734968 0.9574582741857073 0.9737792699263228 0.957457927423283 0.9739595577178239 0.9575936149550861 0.9727767931646694 0.9576761982464685 0.999886130223518 0.9578081614240429 0.9727102531645571 0.9570517021276597 0.973759598631699 0.9577933471578435 0.9728284158857653 0.9571523168077388 0.8795710567617055 0.9571159594006767 0.9737478871998236 0.9574653173449613 0.9999121785437645 0.9575723762135923 0.9711183132825927 0.9572190462357782 0.9909595263840832 0.9571076895219701 0.9711590850453894 0.9574747999029832 0.9998531403989049 0.957543951848249 0.9728001588011631 0.9576893279766253 0.9727258632212075 0.9574641699029127 0.9729023763035279 0.9575099079680311 0.9728281603773585 0.9591949095488894 0.9713241015812171 0.9704718563588642 0.9727322490498547 0.9704718563588642 0.9730678015301529 0.9718339107551488 0.9726968604393166 0.972143409194356 0.9736009125055236 0.9575207084139263 0.9729842056074766 0.9914179712631354 1.0 0.9906561967592593 0.9725272368421053 0.9908861180555556 0.9982371615252784 0.9906857735176103 0.9724606254375731 0.9904376165803109 0.9726292134062928 0.9727193226022804 0.9726330825353396 0.9741869283201406 0.957044659255679 0.9727397091474245 0.9589695242369838 0.8848508493550743 0.9731408463100881 0.9587871107430195 0.9997417958300551 0.9730214816482774 0.9736867917235107 0.9729764169453734 0.9730913044444445 0.9731690952597994 0.9740557101896781 0.9602266658986175 0.9724376295133439 0.9724480273481281 0.9913242588996763 0.9724490788883908 0.9724968604129264 0.9717392503470617 0.9724554950716845 0.9724117062248097 0.9724873476702509 0.8784943841562669 0.9712180759665385 0.9588024693641619 0.9587764113836188 0.9713391721330017 0.9714470761947699 0.9713489361702127 0.9590710925332112 0.9723633886810104 0.9703766884057972 0.9704215234186383 0.8858100824175823 0.9590753061224492 0.9586851555352242 0.9714146356868938 0.8855075122910522 0.884394823855058 0.8782830576771135 0.8848874591002046 0.9729316857720837 0.9728144291826165 0.9742070128118678 0.9702654841779681 0.9901590727229936 0.9486814633546484 0.9717640721533259 0.9715424960434095 0.9718296227693698 0.9717736441828883 0.9714010599909787 0.9715385264825713 0.9716216852864275 0.9715157831039392 0.971566478973628 0.9716207741629067 0.9715840442225391 0.9712521080305928 0.9715878396383535 0.9719594840351689 0.9717988958478312

GCA_003965785 0.8715101909994766 0.970725725925926 0.956998435105068 0.9573217641253392 0.9573937956748695 0.9707914766902613 0.8783538162930563 0.9717388085416161 0.9709502794117648 0.9708813364167479 0.8710593377995364 0.971147191259514 0.8709673716951788 0.9573445528656126 0.991507443411961 0.8785065844973979 0.970730844123506 0.9572533117529881 0.9708058607160374 0.957298900576008 0.9710397641050584 0.9571458811881187 0.9701186986301369 0.957152652293808 0.9709925877731402 0.9572351148277584 0.9704858627450978 0.9570889150588825 0.9711497045790252 0.9570737921626984 0.9710247775866306 0.9573040529735133 0.970231675238795 0.9709846100519931 0.9908051630695444 0.9571631002737 0.9913939754485364 0.9570372612568037 0.9702658251473477 0.9572209066205535 0.9914165763430726 0.9570975402526628 0.9908093564356436 0.9571570888888888 0.9701838314082083 0.9571019404466503 0.9716734310134311 0.9570670421836229 0.9717800489955903 0.9571898963474829 0.9703583827821011 0.9570046287128713 0.9912986016949151 0.9572226382345649 0.9701282692778458 0.9570343901834406 0.9707589817554241 0.9572802532274082 0.9710557771765865 0.9570850794044665 0.8784721774856203 0.9569772348860258 0.9718456695992181 0.957112950456002 0.9913216036628316 0.9572553067788223 0.971435403773585 0.956908961681088 0.9910881655534352 0.9568636484818565 0.9712008135841681 0.9569763269849123 0.9913101016788839 0.9570773412698413 0.9705110113468179 0.9571242825333994 0.9701623522197694 0.9569893799407114 0.9709625159391859 0.9561640378006874 0.9710685650777969 0.9576671951819076 0.9712372021484376 0.970904180845892 0.97028007390983 0.970904180845892 0.970798938685053 0.9707827099143207 0.9701030246762764 0.9712477825261158 0.971014800988875 0.956126938675788 0.9710516052114061 0.9912631015037594 0.9914023894637818 1.0 0.9703639995089615 0.9912213001867415 0.9908033380182713 0.9919878595929568 0.9715519732937686 0.9908111814042657 0.9712513381294965 0.9708639635647466 0.9712725958772772 0.9716661390243903 0.9570903344067379 0.9705244098922624 0.9586862919463087 0.8758662171398527 0.9709758635029353 0.9565915316230633 0.9912996379228204 0.9706584919653893 0.970971945467944 0.9707801815017405 0.9706210876937985 0.9710679537629119 0.9705218532725965 0.9559965984717771 0.9708291557813256 0.9709290024213075 0.9908507305080778 0.9709061406476559 0.9711659798754192 0.9706169412343252 0.9711299760994264 0.9711094241338113 0.9711482962962963 0.8781879322687223 0.9709424652862363 0.9571747849728125 0.9571753240969817 0.970948247322298 0.9709596445093742 0.9709691843194546 0.9571861823573018 0.9711955317577549 0.9703725030704986 0.9704170632973503 0.8789804903536976 0.9571779440870855 0.9571559331683167 0.9709397756097561 0.8787876480042861 0.87867634270415 0.8771859183121449 0.8797799319357473 0.9710917224765118 0.9711349613899615 0.9713545079754602 0.9701937451550388 0.9905566698795181 0.9482882210927573 0.9708567775061124 0.9707983247863249 0.9708627477367262 0.97088700048852 0.9707426039119804 0.9708507228620437 0.9711513930226885 0.9709243489900219 0.9711619386264003 0.9711538581525713 0.9711683304898856 0.9705801965119135 0.9711028334552547 0.9718052676192784 0.9717261540324533

GCA_015684795 0.8767926043446426 0.9914662339844571 0.9590189970299292 0.9593764569237709 0.9571595026557219 0.9882323537146614 0.8845368669851648 0.9768963297285366 0.9875764274536105 0.9892984084656086 0.8797643765962387 0.9921302691818568 0.8797642315740527 0.9592635931128228 0.9697773857315599 0.8844451371759376 0.9914606055473216 0.9567643053214545 0.9917598669161989 0.9567743392123701 0.9743491598216382 0.9569498558731684 0.9998438681446907 0.95699057496361 0.9918235312697411 0.9568403419215025 0.999800762463343 0.9567292508458194 0.992177655532359 0.9567396859266364 0.9896714784482759 0.9567976075398743 0.9998715859116559 0.9882779953757226 0.9716525346229941 0.9570104089039438 0.9720132792352156 0.9570072535380187 0.9998565968688845 0.9566417804295944 0.9720299777530589 0.9566932263473055 0.9694012860892389 0.9566514677804295 0.9998308119741733 0.9566835313847628 0.9898501385336743 0.9566253539716822 0.9773563348920062 0.9566842693135612 0.9886278146114864 0.9569614038876891 0.9720147501665555 0.9571261974537595 0.9998180837390459 0.9565551746411483 0.9878143756809763 0.9566760412173497 0.988843900467886 0.9566754878925917 0.8793920130890053 0.9566373855739276 0.9766726339186487 0.9565946076794658 0.97191476285461 0.956711470236672 0.9868291191709845 0.956645581339713 0.971921437792121 0.9565498042492242 0.987239963478658 0.9566927511333811 0.972003977728285 0.9567106085289889 0.9998223502668512 0.9570456631427199 0.999770175815589 0.9566929787234042 0.9889038613231551 0.9566716180688337 0.9888007049775689 0.9582297532820282 0.9872213743425566 0.9885738902686838 0.99982959913539 0.9885738902686838 0.9917640546792851 0.9890871534115603 0.999848372815534 0.9873267337122869 0.991762505766408 0.956592900999524 0.9890031393372982 0.9709991749323714 0.971999301242236 0.9691723467179609 1.0 0.9692205211803903 0.9693848685145701 0.9695831650943396 0.988734663846839 0.9716893032427696 0.9877728173913043 0.9917989297729185 0.9878533470984568 0.9774803827325323 0.9569369030116197 0.9998361797532799 0.9584144981916818 0.8826141450528339 0.9763970588235293 0.9581121128084606 0.9720427629513344 0.9887421771933608 0.988676319474836 0.9914944292044736 0.9898839290267744 0.9764656060260215 0.9881456515248453 0.9586162908758289 0.9882138583013231 0.9881364886800512 0.9706915116542204 0.9881394639043144 0.9882293421614695 0.9874978287596048 0.9882490697674419 0.9882006909788867 0.9882693068884624 0.8774737065740245 0.9877504918743227 0.9583917873510541 0.9583767270226909 0.9878836377440348 0.9879388543017725 0.987884499457112 0.9586072126306224 0.9880051774949764 0.9873144869445717 0.9873535966117216 0.883816534849234 0.9586263638428344 0.9583569943374859 0.987909254376486 0.8840051732552465 0.8838871506849316 0.8771258398539385 0.884244482151835 0.9878556510416666 0.9877496256800871 0.9890741204506065 0.9884848585652865 0.9694522694524497 0.9493439102405157 0.9880465982721381 0.9878890335861321 0.9881296885813147 0.9881735060711188 0.9876477672003462 0.98790468241925 0.9751612058753851 0.9746766329173534 0.9751412263257575 0.9749061312485193 0.9751191714015152 0.9747769835987639 0.9750409198672357 0.9880097140957448 0.9878676437930269

GCA_003985265 0.8711440116741841 0.9690381146953405 0.9558648979095772 0.9568072512896093 0.9568287023838781 0.9703194345600781 0.8767782524005487 0.9716140053178633 0.9715734274490546 0.9709291977836667 0.8716474240490171 0.9710750329027542 0.8715156184379003 0.9567550442260442 0.9915164331210191 0.8767273017170891 0.9693494979483466 0.9551915388373229 0.9692747329693806 0.955307259768985 0.9694195010793955 0.956183276492083 0.9694270817399617 0.9563347439684884 0.9705361923169846 0.9559067098825833 0.9704096725685181 0.9561422173058014 0.9704678185328185 0.9559618281669513 0.9720023562933596 0.9550145100440961 0.9694782112068967 0.9706289560572955 0.9898041770661644 0.956121577140054 0.9914925856405443 0.9561351314508277 0.9695823108335335 0.9550905906560155 0.9914643134957827 0.9561386897223575 0.9897876256458432 0.9551325958272682 0.9696172801537722 0.9549310148094198 0.9716695465567411 0.9558710645554203 0.9720141593137256 0.954889905385735 0.9694022661870504 0.9562176065254445 0.9915180575035065 0.9565008077110786 0.969451486712952 0.9561622855750487 0.9707483686023622 0.9554610564751704 0.9707345450133462 0.9561214418377322 0.8769860861321778 0.9560053107482331 0.971661610787172 0.9547901574993942 0.9915090711785297 0.955146859083192 0.9699411426456069 0.9558149599027947 0.9909468397237438 0.9558502475728156 0.9702151153291688 0.9560632830372353 0.991473242481203 0.9560922626311935 0.9705005719872891 0.9562128233861147 0.9695078703481393 0.9559203111327176 0.970876093372037 0.9549634181201551 0.9708020626376315 0.957137152301665 0.9702955297219561 0.9696364551790435 0.9696373781958515 0.9696364551790435 0.970118789410349 0.9696514958717825 0.96944551996175 0.9693869247009148 0.9695817783628531 0.9549011312545322 0.9707281562348375 0.9916867326963411 0.9915124572899603 0.9904787928221861 0.9692827277096854 1.0 0.9898020685634328 0.9914739742113746 0.9716600124007938 0.9899051647058823 0.9690896162097736 0.9704603407443209 0.9690981443544545 0.9721244591287322 0.9550607733076367 0.9704188506304557 0.9567881859682845 0.8733457345597898 0.9710198290598291 0.9559112515739109 0.991474911368471 0.9698279482169028 0.9701985190615835 0.9693945290291497 0.9696333492594363 0.9711125190651907 0.9694894946107785 0.9565215620402158 0.970102209631728 0.9700906734404537 0.9905855892731122 0.9701681379962193 0.9701115379163713 0.9690233146751034 0.9698712 0.9698738329411764 0.9698786756057398 0.8768118126888219 0.9706417649903288 0.9560256593673966 0.9560475547978569 0.9705780609284334 0.9705851220691323 0.970635328820116 0.9559385547445257 0.9706806836363637 0.9696179147493339 0.9696311573849878 0.8774453963988177 0.9559003502797373 0.9553511707789066 0.9706868153174988 0.877137215953818 0.8769441888619856 0.8767561429336188 0.8784987083560896 0.9698475883625871 0.9698612099301037 0.9720439499509322 0.9686854484891743 0.9900086339962121 0.9472169551934827 0.9708272839506173 0.9711857985436894 0.9709485032695568 0.9708671058367645 0.9708705428986911 0.9712350789793439 0.9709367447476455 0.9693779623899073 0.9712122093304326 0.9713653475417776 0.9711126291646548 0.9711161151603499 0.9712381580855692 0.9710865848375452 0.9710461725717039

GCA_015684855 0.8686163018200201 0.9708563701067617 0.9565846973995272 0.9578536257035647 0.9579974880268197 0.9704255674918263 0.8793989172306091 0.9715350973291083 0.9717731928838953 0.97129253039688 0.8730469223371001 0.971920009494422 0.8729428175463969 0.9577004643527205 0.9916530743469525 0.8779181906258184 0.9710495276717558 0.957779104477612 0.9709591026544516 0.9578406593668176 0.9716723495767561 0.9575626422280409 0.9709842830054516 0.9576505649038463 0.9717851734864801 0.9573375908221796 0.971115789223831 0.9574966794902622 0.9715223521093566 0.9572878924833491 0.9719713825534916 0.957726735566643 0.9710689099526068 0.9709368083074719 0.9900014789840413 0.9575808311377246 0.9992003274878514 0.957452730513308 0.9712026409707352 0.9576677937030076 0.9991862194092828 0.957303716793169 0.9999210923014914 0.957720356640075 0.9710655235602095 0.9578104928082999 0.9715058807147832 0.9572133103448277 0.9716102314390468 0.9577966737188528 0.970758671552909 0.9575486738095239 0.9991771229991575 0.9577587565507385 0.9710898150343847 0.9573977672434226 0.9720076187118007 0.9577529163705283 0.971448504867872 0.9574945811518325 0.8799312188223195 0.9574285819302822 0.9718858457312887 0.9576239849800515 0.9992369861286254 0.9577246490814885 0.9721810366291597 0.9573617736206488 0.9910292569241161 0.9572591216856061 0.9717112932647335 0.9575144982206406 0.999196316235693 0.9573525332699622 0.9714885574162679 0.957498468854018 0.9711172247324613 0.9573111738202513 0.971336044362292 0.9577002747768906 0.9714206739484081 0.95751614654968 0.9719551656095843 0.9710211260182079 0.9711726167105578 0.9710211260182079 0.9714776744186047 0.970765431533269 0.9710424224484964 0.970694142094265 0.9711762180094787 0.9576570782567947 0.9714287985212569 0.9908932822562736 0.9992014842105263 0.9907156089309878 0.9709513123359581 0.9906608450385139 1.0 0.9916914177777778 0.971536588907403 0.989996692066338 0.9714617479768787 0.9715817055359247 0.9714435190751445 0.9715888444850422 0.9572933015721772 0.9712719174181301 0.9573542657992566 0.8808899363992173 0.9715269692020533 0.9563702880460874 0.9992187347463071 0.9708146119682094 0.9718059595008243 0.9709388021951801 0.9678548866265618 0.9716712126909519 0.9714607487978018 0.9573152552480917 0.9709350644567221 0.9709352279005526 0.991199653718091 0.9710088656235619 0.9709375195762321 0.9702642928452577 0.9709181471264366 0.9709283444469992 0.9708825477121178 0.8779442906666668 0.9707303772706102 0.9570502016129032 0.9570674442863917 0.9705943322475571 0.9705833333333332 0.9705932402234636 0.9569499739274709 0.9711067868152715 0.970587894486692 0.9706334726840855 0.8779566874674989 0.9569250723262985 0.9569334899169633 0.9704822688096902 0.8794359310518371 0.8773494971339239 0.8778906687322489 0.8791325410049468 0.9723498175675677 0.9722543874321882 0.9720434839310664 0.967464177244946 0.9910415466297322 0.9477630926829269 0.9705678334493159 0.9706143535988818 0.9706867357875116 0.9705448997201492 0.9706665983224604 0.9706502379286214 0.9716961013781827 0.971473951105937 0.9716731000699791 0.9716985611007463 0.9716738109684948 0.9713093255269322 0.9716464557553117 0.9708541611531192 0.9707769172576832

GCA_003986775 0.8716093880673126 0.9704132683622534 0.957621122399613 0.958009886005336 0.9580143048992789 0.970241957659372 0.8784909373326193 0.9717571165209171 0.9711297038285577 0.9710803559641679 0.8712585199004975 0.971898507784431 0.8712021895261844 0.9578548278370516 0.9914545285647932 0.8784217252568958 0.9704873520249221 0.9577491907229212 0.9705833680473372 0.9579368792029888 0.971657967557252 0.9579732113427047 0.9703296218286263 0.9579539279588337 0.9717388875540606 0.9579049780594833 0.970440505510302 0.9578835546300835 0.9713378286805059 0.9577954233576642 0.9726284021500123 0.9579557265166342 0.9704646452540747 0.971029540229885 0.9893563988919667 0.9579954313967862 0.991143058637084 0.9578664439603003 0.9705646783907491 0.9577883136023193 0.9911692684031711 0.9577924600096946 0.991108480516486 0.9577357184255011 0.9704219514539775 0.9575834091458988 0.9712435021296734 0.9578168098010674 0.972157169267707 0.9577736682808717 0.9707640341718083 0.9579519757869248 0.9911461545413467 0.9579981101407085 0.9703496093937216 0.9577628813149626 0.9714214282244352 0.9579222216832401 0.9710259164677802 0.9576489661016949 0.8781327525855211 0.9575989071566732 0.9710076924904943 0.9576809325924137 0.9911038156411991 0.957845511982571 0.9714188988095238 0.9575430166096728 0.9905464657980457 0.9575223523670084 0.9704717156511352 0.9576136884057971 0.9911287363883848 0.9577825212121213 0.9704796094503376 0.9579082861292666 0.9703014056128568 0.9576005316578057 0.9710044373066857 0.9576923357487922 0.9710381885796545 0.9579084710443422 0.971329403957529 0.9708413577894236 0.9705349068473266 0.9708413577894236 0.9719143702984713 0.970870723383802 0.9704335271596076 0.9710782219061166 0.9704784489311165 0.9576110795864391 0.9711432959427206 0.9907079556761648 0.9911023426889997 0.9914993752849978 0.9704136516718787 0.9907882464998854 0.9911547758462946 1.0 0.9712993948196563 0.9904838334496395 0.9716841692491061 0.9704436866028708 0.9708574840839425 0.9721006154580154 0.9572371786757548 0.9705309487302348 0.9576842188235295 0.8760500444676954 0.9712198657396307 0.9577281059470265 0.9911129958772332 0.9706965559566787 0.9706071693057249 0.970513516299137 0.970259178243466 0.9713004795349964 0.9714300856938825 0.9579506793279766 0.9712958098174056 0.9713316769449715 0.9912115789473686 0.9713591615348176 0.9714166832740213 0.9708096549192365 0.9712431891252954 0.9712549739952719 0.9712761914893618 0.8778383365044851 0.9706890142517814 0.9579991489880517 0.9580009746588694 0.970652938804554 0.9706701470937129 0.9706769205219457 0.9578553967867575 0.9712283276450512 0.9717112081513828 0.971734262493935 0.8786766831040749 0.9579116646356325 0.9576217748601993 0.9706756328663027 0.8782530169491526 0.8776249694230259 0.8761033448642895 0.8793304538087519 0.9721397778839265 0.9721995861244019 0.971730120569086 0.9703914251724959 0.9905573797169812 0.9479834521158129 0.9705613824994072 0.9707657278556162 0.9706306273764258 0.970605675996205 0.9705366034155597 0.9708743503093765 0.9717182929171669 0.971744219611604 0.9717841546589818 0.9719098895292987 0.971692203023758 0.9713048756339049 0.9715723638108952 0.9716720211233798 0.9716133197311569

GCA_004011415 0.8770006927564415 0.9860806811931457 0.9617677210091159 0.9570508982035928 0.9545869300984866 0.9859892028529472 0.8838206466798812 0.9756065773681953 0.9865738897297297 0.9873962794543201 0.880494461361015 0.9871381014276581 0.880349400740398 0.9569259625250948 0.9680871769527483 0.8871094655004861 0.9862799037227213 0.9545355491744436 0.9867716754478398 0.9544762385762386 0.9734427469426153 0.9540252297902428 0.986754129290377 0.9539139857312724 0.9871321865020227 0.953982780279687 0.9867028767123288 0.9539319199618866 0.987087448611994 0.954204139237509 0.9874284073216916 0.9538838679021144 0.9867467869198313 0.9875984338168631 0.9695101346070344 0.9545809519253767 0.9703391086717893 0.9540002542372882 0.9869168673166597 0.9537836771616541 0.9703854918752746 0.95391336552049 0.9676585921177587 0.9537267693392899 0.9868086417405999 0.9535082472281198 0.9868601292838087 0.953934062869298 0.9750366360052563 0.9535278742937854 0.9872889194826867 0.9540512166941758 0.9703707457775829 0.9541060387157696 0.9867364883084053 0.9539281168984208 0.9881068691099478 0.9538010487862363 0.9874317078792959 0.9540362777515351 0.8784944618191294 0.9540016147308782 0.9762391248937978 0.953647367183827 0.9703065505913273 0.9537685314191575 0.986436207852194 0.9544999309523808 0.9706864313555652 0.9544434983341268 0.9851949911504424 0.9539874381916648 0.9704000286532951 0.9539656991275642 0.9869193319194062 0.9540652849740933 0.9867062230215828 0.9540084593639577 0.9873983917396747 0.9535782435927581 0.9875231607859709 0.9591930658838876 0.9862677939856375 0.9861185256553887 0.9867937909052273 0.9861185256553887 0.9874622609625668 0.9873206103678931 0.9867521111344981 0.9869432878755593 0.986678897338403 0.9535600164165104 0.98746025769956 0.9697411106184882 0.9703051238763429 0.9676999427890346 0.9867780088870081 0.9676927014104709 0.9676553399433427 0.9674518169112508 1.0 0.9702620873362444 0.9873772548605242 0.9872495372750644 0.9867794104118754 0.9750796251906735 0.9547423628591782 0.9869736812144213 0.9588750235546039 0.8826614355856909 0.975471099716343 0.956119459833795 0.9704263814180929 0.9870211861544955 0.986373708315745 0.9862914665815241 0.9861382640032949 0.9756047770700638 0.9877100946307344 0.9585283066461945 0.9876349291726545 0.987631059330733 0.9702394777434994 0.9876041875256462 0.9875848079687822 0.9868612025979466 0.9876911015561016 0.9876999938574939 0.9877447041965199 0.8764365724946694 0.9901083943833945 0.9616671991294885 0.9616732877009996 0.990300407000407 0.9904031856796115 0.9903764058382324 0.9618955305813702 0.987380341524365 0.9872954160125588 0.9873075078440161 0.8919043974924543 0.9619005383783783 0.9618856320346321 0.9903082232576985 0.8884754577218728 0.8866341919312486 0.8766055455942623 0.8873246090335115 0.9902127398093693 0.9901866917446117 0.9875521829393629 0.9858162691538973 0.9681470324427482 0.9475614744381329 0.9903686230567335 0.9902959124823554 0.988152 0.9903894496344435 0.9902564520048602 0.9902537598544574 0.9740462164073551 0.9739285603387439 0.9739867577756833 0.9740281082352941 0.9739740325241576 0.973679648253069 0.9739497084411004 0.9866118203259007 0.9864566087516088

GCA_015684875 0.8773296935244685 0.9721160181496237 0.958826450867052 0.9591608246488446 0.9572178427075726 0.971686749435666 0.8858110473313192 0.9732921547438946 0.972156638540479 0.9734796282688567 0.8803777288609363 0.972581136723164 0.8802958471605515 0.9589800955848885 0.9902256523754346 0.8854633677842197 0.972107567326953 0.9571099975675018 0.9726665944407966 0.9571063093489967 0.9719503243744209 0.9574429941719282 0.9717982134266346 0.9575343325985795 0.9733856334231805 0.957086814310051 0.971876017524644 0.9571764383561644 0.9726807012530226 0.9571074533559487 0.9742384870188004 0.9569638600341379 0.9718316648387805 0.9703459414024975 0.9999005878917657 0.9575238012695313 0.9902052939914164 0.9575105655339806 0.9718759308217669 0.9569453779280367 0.990273911273039 0.9571721228239846 0.9892995403868033 0.9570052840222384 0.9718242241758241 0.956965784883721 0.9733413885827639 0.9570497919187031 0.973856475751591 0.9568919801740813 0.9729016353424066 0.9574904951456311 0.9902625412117321 0.9576117930029153 0.9718143682942851 0.9568130398069964 0.9733921969189551 0.9570995101842872 0.9731907164567954 0.9568932664410059 0.8797295176348547 0.9568810077145614 0.9733378893263341 0.9569008599033816 0.9902252202737383 0.9569626378143135 0.971279830754352 0.9570175385728062 0.9908502334465196 0.9570599301373163 0.9712359080244017 0.9570460082105772 0.990297511817791 0.9570598838334946 0.9718897995153118 0.9574684546336729 0.9718515640123185 0.9569708763882184 0.9731667092167893 0.9570031610722047 0.9731183779044278 0.9583340833523115 0.9714551308653618 0.9702734662576689 0.9718886903710248 0.9702734662576689 0.9728836202645147 0.9716093997734995 0.971799124726477 0.9719329814773489 0.9726367979641515 0.9568864851842932 0.9731437266811281 0.9903625653310104 0.990238637923638 0.9896812800186523 0.971705670557146 0.9897440295407338 0.9893308095884216 0.9902922113413801 0.9721362284532292 1.0 0.9730276926523298 0.9724356996662961 0.9729929290669053 0.9738819390885187 0.9566861265394833 0.9718884374315144 0.9587496152985909 0.8845608910654563 0.9729695454545456 0.9584184287396939 0.9903665488835899 0.9728582644262658 0.9732508639212176 0.9722105074560428 0.972073070539419 0.9729871966621562 0.9733877488048674 0.9588582402745996 0.9726672324396188 0.9726279254493011 0.9901702700348431 0.9726095653138168 0.9727059485815603 0.9722324142630027 0.9726602564669468 0.9726647181074508 0.9726500176952001 0.8780805076964624 0.9716264762329837 0.9582724457274827 0.9582612719298246 0.9716204558659217 0.9717661432384341 0.9716711322018757 0.9586241479615208 0.9721458555657774 0.9706622325249644 0.9706919672131149 0.8841887332339791 0.9585829049255441 0.9583382425629292 0.9717242586399106 0.885753258261934 0.8839196619083396 0.8773119142259413 0.8851539342172362 0.972467995545657 0.9724537371709059 0.9736479959785522 0.9691236028202116 0.9899501609195404 0.9481128157503718 0.9719075155555555 0.9717356269522535 0.9720821670378621 0.9719853031312458 0.9716443472439187 0.97176607150838 0.9714204397432851 0.9713591953477334 0.9714673989538755 0.9714315073704232 0.9713620161673799 0.9711496082178691 0.9713351272900309 0.9718617636445648 0.9717311869771648

GCA_008369005 0.8765085278413425 0.98835937121042 0.9591109600000001 0.9599276502095947 0.9578395701581027 0.9902002265589951 0.883325955487337 0.9782990963030579 0.9987596456443788 0.9896728327121246 0.8788750484027107 0.9896690658844766 0.8787788940092167 0.959603069376314 0.9711805446892795 0.8834625789205705 0.9883915461573444 0.9572456518554688 0.988706844681798 0.9576354681739985 0.9760697251024837 0.957708364394311 0.9886569944382647 0.9578919076005962 0.9894755794183445 0.9575203863188978 0.9892587074372762 0.9577958788480636 0.9897089040788885 0.9572243794369645 0.9912943223933914 0.9574771341164955 0.988669074074074 0.9907291174377224 0.9729164167051222 0.9578878658083686 0.9729075554000467 0.9577449620004903 0.9886361004034067 0.9572900048297512 0.9729540666511303 0.9572388655256724 0.9705272326589595 0.957210572187349 0.988548434569004 0.9572548046590634 0.9908907588793292 0.9572615862913096 0.9787635419071807 0.9572519651500484 0.9890729885814667 0.9578643382352943 0.9728791940368041 0.957894283959715 0.9885914091718612 0.9574245234604105 0.9897125763612218 0.9572726006343011 0.9897307911745041 0.957652904808636 0.878624597887896 0.9572992536585366 0.9782642042318308 0.9571546837276678 0.9728967455346788 0.9571633785091964 0.9896834296541924 0.9575206186069165 0.972733931446975 0.9574653320360011 0.9896322994285714 0.957314541015625 0.9727559477124185 0.9573434378828718 0.989279233551888 0.9577827290543851 0.9886932430011198 0.9572575762753235 0.9898221809334219 0.9572909090909092 0.989732913403446 0.959985445822607 0.9900344062356717 0.9895158574712644 0.988564822072072 0.9895158574712644 0.989682105736783 0.9898047014595313 0.9884757088888888 0.9984456328600406 0.9887998857526882 0.957167489167068 0.9898112722419928 0.9721078284119973 0.9728546320447136 0.9705555443403029 0.9886697649429147 0.9693706637910375 0.970506023489933 0.9717227948226271 0.9905331584922797 0.973291081143384 1.0 0.9895617575757577 0.9999083429339478 0.978533001150748 0.9573889667806544 0.9893718873429085 0.9600714034275127 0.8833619354838712 0.9787435244755244 0.9574273164861613 0.9728359788235296 0.9899091394262842 0.9900409052771031 0.9884120320976493 0.9898850635129216 0.9786769995300754 0.9946905737529439 0.9591184653233364 0.9888723496076722 0.9888192148309706 0.9722460226746877 0.9888699105974705 0.9887540479302832 0.9881068652387326 0.9888155869565217 0.9888207521739132 0.9887664608695653 0.8768443684068165 0.9889078501556249 0.9591293189290748 0.9591168280075187 0.9890348732206407 0.9891206207049434 0.9890529640319716 0.9593508265424913 0.9906373149588799 0.9890766705716962 0.989080316085226 0.8841510951299522 0.9593961148570099 0.959103734911792 0.9890819409937888 0.8846365050251255 0.8842439748110832 0.8767417804232805 0.8846059117722493 0.9954019957310566 0.9952918047527297 0.991139364726788 0.9890476484123332 0.9713089072520968 0.9505210772715832 0.989237777284827 0.9893217364203029 0.9894041205247943 0.9893164574090506 0.9892018573021183 0.9893495471782289 0.9772482590898146 0.9763610893454372 0.9772719104908566 0.9773628523328525 0.9772518043844858 0.9769534919864014 0.9771192202166065 0.990689865531152 0.9905508482944345

GCA_015684935 0.876903175976273 0.9975957482430758 0.9590482317497103 0.9596995179904956 0.9568935420743641 0.9893458529542426 0.886502312703583 0.9771345956210903 0.9876645709123757 0.989235021312873 0.8793757059099439 0.9987209169054443 0.8792720127118645 0.9594485691537762 0.9698868260766119 0.8850995970788216 0.9975923175067978 0.9569450831295844 0.9979358672114401 0.9573228458887248 0.9766279551297032 0.9572845872899928 0.9917105820550536 0.9573470580994584 0.9987383291666667 0.9571877642177203 0.9921242799831081 0.9569844732974033 0.9980236248467512 0.9571937417943106 0.9906525356437818 0.9571477843137254 0.9921597106652589 0.9888161333955224 0.971685095896521 0.9574096144400787 0.971896522032374 0.9573938901045465 0.9921170749310124 0.9568372958565543 0.9719752435465769 0.9569613841670714 0.9702131677756655 0.9568949866601989 0.9917496620194339 0.9567832425200682 0.9903154034176942 0.9570509555069293 0.9776655384615385 0.9568794420184377 0.9889114430107527 0.9573514987834549 0.9718732891782668 0.9575821224688948 0.9917413152356902 0.9573070111867705 0.9889922598618229 0.9570181459854014 0.9889320730652833 0.9572395959104186 0.8798079013641134 0.9573021760272308 0.9771093065281228 0.9568394274623969 0.9718849440465532 0.9569531333495264 0.9886918256386221 0.9571794812121214 0.9717843002028397 0.9571419413475521 0.9882919583427666 0.957077394042141 0.9720453160854894 0.9569853616504855 0.9917106532343585 0.9574497710667317 0.9916708062473619 0.9569713468992248 0.9890115715823466 0.9571179019138755 0.9889456869565217 0.9588555006890216 0.9885378520885643 0.9890346129612962 0.9920791922421143 0.9890346129612962 0.9981586458333334 0.9894066251366122 0.9921229510961213 0.9874606703417861 0.9978769993813158 0.9567959288824385 0.9891664281095064 0.970686247446084 0.9720580385563775 0.9697832540645475 0.991840373752389 0.969707278148855 0.9700669149186897 0.9700052727272727 0.9892516169828365 0.9717049209882039 0.9887782267313631 1.0 0.988844239082488 0.9775188906390639 0.9575464722753347 0.9921572308991137 0.9582900954762446 0.8842157999520269 0.9769288853430591 0.9588866627330659 0.9719986948654152 0.9898054776511833 0.9887076471890217 0.9976156290289042 0.9897586589792867 0.9769353117092249 0.989176002595717 0.9590063532697229 0.9887700904003446 0.9887344710191769 0.9708187679344111 0.9887062392241381 0.988814790948276 0.9878633178500333 0.9887897656417975 0.9887580541818963 0.9887905848204688 0.8777438854530787 0.9882394071319186 0.9589192134570766 0.9588708526951674 0.988356536862831 0.9884256619411123 0.9883833457411868 0.9591587689829728 0.9886091323994578 0.9885610006965405 0.9885893781902553 0.8847444017835027 0.9592636541561133 0.9591890831605622 0.9883967102966843 0.8847741826215021 0.885869812761764 0.8790302971071151 0.8861313418290854 0.9883625092411394 0.9883440200348433 0.9896532708425233 0.9883775358688226 0.9689627211376235 0.9495189189189189 0.9883382608695652 0.9882128384279477 0.9885958251580554 0.9883501327818894 0.9881966644823068 0.9882490865384616 0.9758162782940194 0.975860271363961 0.9759488565983803 0.9759563309523809 0.9758813846520495 0.9752673136832017 0.9757445489260144 0.9885079474729641 0.9882870735489587

GCA_008369025 0.876489403517716 0.9873668733392382 0.9585792086330934 0.9597538721716817 0.9577765463535229 0.9895221668520578 0.8834144971780401 0.9775132236842106 0.998243174068617 0.9893199364035088 0.8784948579752367 0.9893712939585213 0.8783239863879436 0.9595593214787086 0.9709452861035422 0.8833989628876462 0.9874714260089686 0.9569893274853801 0.9876963348017621 0.9572901909424725 0.97538059569378 0.9566532258064517 0.988325344291426 0.9566895028165565 0.9893800067174207 0.9564241109219169 0.9889519802867384 0.9566943140617346 0.9892657632817753 0.9563644876153472 0.9913342035794185 0.9564857211770381 0.9884016180075774 0.9904927180094788 0.9723322850627139 0.9574227115716754 0.9726441921193751 0.9567145101842871 0.9883413266219239 0.9562632308059132 0.9727423334885886 0.9562993707647628 0.9703210867997115 0.9562029441260745 0.9883233839624748 0.9563106316042267 0.9898719226567622 0.9562328924418604 0.9779807865685372 0.95622403722262 0.9888603490669593 0.9566794979383945 0.9726492527932962 0.9567809232264335 0.9883370954818607 0.956438873613121 0.9893087107474569 0.9561370768119432 0.9895376402493321 0.9564870314769975 0.8795712042025862 0.9565659288824382 0.9771247278524254 0.9561619728053435 0.9726987738039945 0.9562624317855434 0.9898261080953504 0.9572348501096758 0.9725970126582278 0.9571665182481752 0.9890467951916534 0.9563739303843366 0.9726529011913104 0.9563743636363637 0.989048299073865 0.9567174515033948 0.9883529372347555 0.956275721537346 0.989548181214001 0.9562971428571428 0.9895345664351334 0.9597142252862818 0.990323148701448 0.9894531270133458 0.9882904272543289 0.9894531270133458 0.9894782751632515 0.9897884967899048 0.9882924800000001 0.9980273955178679 0.9877826632720833 0.9560556558935361 0.989524266340596 0.9722459827264239 0.9726328095903166 0.9708620838352205 0.9883929426211208 0.9689917321514134 0.9703327144563919 0.9709557638393918 0.9898559380610412 0.9731997485002307 0.9994243062476045 0.9893433677361455 1.0 0.9777263005252341 0.9569211919882755 0.9890414903630658 0.9598146791320408 0.8835351542385234 0.9784513751451802 0.9573328417006967 0.9728111946277097 0.9896197806823216 0.9898777976980965 0.9874663366115334 0.9889266355748373 0.9784418751466791 0.994618192513369 0.9588184228971963 0.9886217266187051 0.9886231245908793 0.9722939995368226 0.9886827356020945 0.9885409725250763 0.9879430693731877 0.9885745326086958 0.9885791847826088 0.9885346879756469 0.8771884095212334 0.9887827821054974 0.9591368438381938 0.9590435952941178 0.9889171622523926 0.9890011471045042 0.9889346534873391 0.9593558759038954 0.9903497009304385 0.988950955399061 0.988955248709526 0.8848686244704709 0.9594287933845794 0.9591408094905792 0.988934913410302 0.8848841751679521 0.8843469679373895 0.8767798416886544 0.8841879651906834 0.9949722375984671 0.9949386211312701 0.9903194444444445 0.98889232718894 0.9712170345679012 0.9502551309164149 0.9891472791833112 0.9890551612903226 0.9892959395152324 0.9892207393428065 0.9888510244988864 0.9890688621151271 0.9768121442307692 0.9758496328963051 0.9767944425756848 0.9768772725090037 0.9767873798076923 0.9766427473326869 0.9766289457252643 0.9905981710053862 0.9905012277940185

GCA_015684945 0.8779591120922247 0.97798853082641 0.959440403762331 0.9599414665465195 0.9578285235551238 0.978654338861497 0.8876083172002979 0.9986568267097035 0.9776343591485507 0.9786579910333049 0.8821969754515979 0.977913522448068 0.882251115750406 0.9596855904977375 0.9713329220932987 0.8865954878961817 0.9779414187541565 0.9572099078117419 0.9783584004352557 0.9575155189255189 0.9980043258426966 0.9575994811776063 0.97797531181379 0.9576812579229644 0.9786685806884219 0.9573027503015682 0.9779660808435853 0.9574523417721519 0.9782985610401057 0.95722775 0.9799367459435431 0.9571664000966419 0.9780057915567283 0.9775501448575569 0.9731135082813509 0.9578206599853337 0.9741907798960139 0.957592624216112 0.977976562085727 0.9570814073719482 0.9741652489177489 0.9571984134615386 0.9708366697523723 0.9572089019419804 0.9780375556535156 0.9571115869199328 0.9793422280243691 0.9572422786333011 0.9995656170634921 0.9571223519539679 0.9783994810508183 0.9576444648023145 0.9739403552859619 0.957817011105746 0.9779877394551845 0.9571837162000479 0.9792287131367293 0.9572519471153846 0.9786363943661972 0.9574217462221156 0.8814058260196178 0.9572946010064702 0.9979381816296911 0.9571442983716476 0.9739224562162161 0.9572148594763391 0.9771723132356475 0.9575122115384617 0.9737236830648673 0.9574587673860911 0.9772471823461091 0.9572485248260973 0.9736785082393756 0.9572317151792157 0.9780086573766866 0.9575927299058653 0.9780009872190393 0.9572487164107486 0.9785892219827587 0.9571027520958083 0.9784695260869566 0.9593376795076363 0.9773861045966229 0.9768131835205992 0.9779700266252497 0.9768131835205992 0.9785361631555357 0.977730871611024 0.9779631965774462 0.977831040834443 0.9785468845649322 0.9570688984468338 0.9787578225108224 0.9724008012394866 0.9741125086655114 0.9711685920492191 0.9779580481342458 0.9710243504030346 0.9708721035673188 0.971531361220245 0.9772257937956204 0.9733893870478666 0.9783702256138023 0.9779176705674542 0.9782186072607261 1.0 0.9575784963855423 0.9780409806508356 0.959322742258855 0.885531171950048 0.9980524059991549 0.9590504709465794 0.9737448981826144 0.9797202325071288 0.9790834635998213 0.9780360326927325 0.9785546509106677 0.9979758074798132 0.9795650358617692 0.9597672419281493 0.9780842891619131 0.9780342709705754 0.9724980660168364 0.9781026783754116 0.9781354950603732 0.9776454375703036 0.978136701754386 0.9780622726276572 0.9781308773853915 0.8808121758648007 0.9768394430992736 0.9589632037718491 0.9589292837402118 0.9768872821302818 0.9769775646646208 0.9768699339643407 0.9593042645381984 0.9777818725824802 0.9771504625655699 0.9771110819828408 0.8864990525030524 0.9593624435318276 0.9591731138952163 0.9769474736379613 0.8868049404617254 0.8860478085580016 0.8787974578469521 0.8855735136488856 0.978196654110348 0.9781720643729189 0.9791050253136695 0.9762433867924528 0.9711267419738406 0.9518527564259486 0.9772618297498905 0.9769466871839966 0.977323813186813 0.9774551417270929 0.9769482684268427 0.9769851739321884 0.9851927394106815 0.9851740500804043 0.9852516386651324 0.9851798068965518 0.9851666428900139 0.9849391121928605 0.9851144124423964 0.9779181776971895 0.977811010215664

GCA_013340545 0.8707487882753204 0.9571735389453315 0.9989764892940427 0.9993716677426298 0.9990365562770563 0.9586003149419844 0.8768592240675197 0.9586902577565632 0.9588658538350218 0.9575768556826479 0.871273734145104 0.9588950140252455 0.8711935445292622 0.9992780859291085 0.9576723291697282 0.8761373923639317 0.9570678108966528 0.9983539919183326 0.9569879455684008 0.9983156264775415 0.9571671335268507 0.9994435249204665 0.9576232604517059 0.9995731266094421 0.9579107660404977 0.9982835213604314 0.9578626355275895 0.9981469926082366 0.9582703349168646 0.9986087662066082 0.958047760236803 0.9979769456681351 0.9576780711880712 0.958885121540313 0.9567441157714005 0.9991015477450772 0.957463861748901 0.9996978423935947 0.9577359467312349 0.9984476005867561 0.9575016992665037 0.9985163624947677 0.9566199162148842 0.9983837235158382 0.9577205378678244 0.9980557700421941 0.9579668712249335 0.9984468107427613 0.9575277081779767 0.9983273294142347 0.9571728002859867 0.9995128214361365 0.9574396465138958 0.9995085377758913 0.9576881697931698 0.998921094875053 0.9573908759305211 0.9982037688229056 0.957643734052993 0.9989376705029838 0.8751246577181208 0.9991193612944433 0.9571412186115216 0.9982190157397692 0.9574114897760468 0.99832226869455 0.9577782580484153 0.9987547589359934 0.9577621828908555 0.9986564388115521 0.9577546853823815 0.9984532760084927 0.9574298972602742 0.9984289591315454 0.9580910019550342 0.9996765275142315 0.9576383843742465 0.9986564011676398 0.9575400318159569 0.9982893463374146 0.9576421046138665 0.9988734031531533 0.9576599297990802 0.9583357350868953 0.957873264563107 0.9583357350868953 0.95808195795649 0.9576661376168784 0.95761631212485 0.9571044845985932 0.9571657547855584 0.99831483521068 0.9576182187730968 0.9554080429650614 0.9574969682926829 0.9570818723823602 0.9578163318039121 0.9564763527980534 0.9566447740667977 0.9572607362557521 0.9585027337774489 0.9569731538839724 0.9573149514327344 0.9583155317096698 0.9573778146980355 0.9577252370370372 1.0 0.95797395157385 0.9898138141246837 0.8721885635359117 0.9580909775364108 0.998665137900356 0.9575724351691777 0.9567782046237088 0.9577862101359703 0.9571702808302809 0.9570741205171994 0.9580846420553755 0.958000830717218 0.9895897166276347 0.9571030552206414 0.9570998649951784 0.9567300821851583 0.9571013379942142 0.9571149168474332 0.9566568104089219 0.9572088827486784 0.95717182320442 0.9572198005766458 0.8768963143407652 0.957322079592332 0.9993836582741554 0.9993932078313253 0.9572944705597287 0.9572882957575759 0.9573002398836928 0.9987723536977492 0.9579464646464646 0.9571008641370868 0.9571245156555773 0.8765763757672805 0.9993687623762378 0.9994726045221843 0.9573914493809178 0.879259780134506 0.8783334804307853 0.8786683350703491 0.8794636982643526 0.9595185424528303 0.9594874153846153 0.9581665690580771 0.9572875671180804 0.9568866379310345 0.9444261311311313 0.957797390356191 0.957856229070614 0.957881395631068 0.9577546780072903 0.9576273944687046 0.9579202529798103 0.9565939377734566 0.9573782982885085 0.9576165126566725 0.9575086336779911 0.9565833049610893 0.9577980004930967 0.9576213822735083 0.9580493156080749 0.9580441186607582

GCA_015684995 0.8768839219099728 0.9922714949109416 0.9591359568545582 0.9594622532969531 0.957240356793744 0.9885297813121272 0.885071411105451 0.9769509784779232 0.98743436797121 0.9890901738024588 0.8801468494117647 0.9923455366269167 0.8801421802737142 0.9594468691482074 0.9697621928120447 0.8853806103166497 0.9922581837652602 0.9570721911764707 0.9924777747483222 0.9571123286661753 0.9749009957224334 0.9572706645569621 0.9999380961727182 0.957365473372781 0.9924020144958432 0.9571720312118995 0.9999365168759812 0.9571532489555173 0.9922406108786612 0.9570621032132424 0.9897206160520609 0.9569179690569745 0.9999720884955753 0.9882781225250407 0.971571348960637 0.9573695553917956 0.9719564245810056 0.9573184059381846 0.9999112141585922 0.9571470113829014 0.972010345982143 0.9571940364520048 0.9696407065868263 0.957052673939394 0.9999386761509584 0.9570564584346135 0.9898986142001711 0.9570812399512789 0.9773375028070964 0.956995707009459 0.9885684876805438 0.9571728303723533 0.9719821391925051 0.9574021749755621 0.9999258313679245 0.9569538343856241 0.9881951742274819 0.9571772335766423 0.9885186678052946 0.9570000024336821 0.8800760238410595 0.9569007561390711 0.9766968296494755 0.9570243587878787 0.9718775895837971 0.9570654838709678 0.9872183649289099 0.9569407513330103 0.9718007793657885 0.9568938141335914 0.9874391007608948 0.9571473777239708 0.9719271578711985 0.9571311232676878 0.9998775313059035 0.9573499756572541 0.9998532826086958 0.9570339244186048 0.9885409608843537 0.9571146125819772 0.988647224252849 0.9582927724578203 0.987615748322924 0.9889770978781656 0.9998920883054893 0.9889770978781656 0.992267389917039 0.9894322763157896 0.9999347843137255 0.9876588842244808 0.9924986493341789 0.9570588789562696 0.9887282684535206 0.9706661451247166 0.9719057407407408 0.9691532858178303 0.9997567819727219 0.9691743895418565 0.9695813854562738 0.9695560720208483 0.9886465427675772 0.9717384029916408 0.9879855130740495 0.9919698858833474 0.98798638480123 0.9773866069428891 0.9572569961427193 1.0 0.9583789786169244 0.8834687763508602 0.9763367222476016 0.9584700402748165 0.9719842342342342 0.9888588607320772 0.9891181073943662 0.9922739756981455 0.9902525459598118 0.9763858296892981 0.9883826669520548 0.9587609860788863 0.9883618524908346 0.9883610384283248 0.9704164032697548 0.9883246168789122 0.9883865011871358 0.9877474350433046 0.9883727656822591 0.9883843651649062 0.9883887235877533 0.8782193398533008 0.9877187723506324 0.9585399721254355 0.9585277130253077 0.987853753275109 0.9879003262287951 0.9878600327439423 0.9588395146077755 0.9882736976115366 0.9879495641143912 0.9879579470046082 0.8843662275000002 0.9589356044209072 0.9586435599448784 0.9879117728953667 0.8843997521070897 0.8845325806451614 0.8768615204678364 0.8847683941792187 0.9879861598951507 0.9879661519929918 0.9890464340073929 0.9888003699474766 0.9693495436986963 0.9492619180819182 0.9879088116445796 0.9876927966841187 0.9879943914652733 0.9880374187922389 0.987419779956427 0.9877376981873772 0.9750310906921241 0.9749791988555078 0.9750259389167263 0.9746919966603054 0.974920350667939 0.9746390146247903 0.974906661093862 0.9880250312082032 0.9879196399821109

GCA_014117285 0.8766683580980683 0.9591807544993078 0.9905386512352549 0.9898480990274096 0.9898438001889466 0.9585910187110187 0.8812012735727202 0.9597666860725482 0.9583963100591716 0.9593270414201185 0.8764035228377065 0.9599205321868464 0.8763376304241834 0.9900485660293464 0.9576691556990317 0.8837087882620795 0.9589528209340532 0.9897672966002344 0.9593431955668438 0.9897903303445021 0.9564816046399227 0.9901924802049371 0.9589315708989807 0.9901580937794533 0.9597063328697264 0.9897817637426901 0.9590659277275887 0.9899563908533711 0.9595963217072051 0.9898807327887981 0.9591938806671364 0.9894857246887478 0.9589007822655525 0.957629448934607 0.9584210191376528 0.9901074699079538 0.9589728492008339 0.990115650453805 0.9590039001632843 0.9895939193884643 0.9590605891866913 0.9897416046511629 0.9571016536203523 0.9896039193884641 0.9590447013711364 0.9893827940492794 0.9593664259344716 0.9898374761127943 0.959080787456446 0.9895819563703876 0.9590340263036456 0.9901420046620046 0.9589772802768167 0.9901083722014926 0.9590083503243743 0.9899220172173103 0.9596642106481482 0.9896203770949721 0.9595447707703049 0.9899181591227251 0.8758815686274509 0.9899155192532088 0.959618347846013 0.9895312497107152 0.9589856609857209 0.9896977832053966 0.9576293810831045 0.9899478256816592 0.9590403685786686 0.9899079525360632 0.9575462026832774 0.9899188385598141 0.9590501598702502 0.989926533892383 0.959020493798268 0.990155670703307 0.9588817629250116 0.9899394027422729 0.9594990795559667 0.9885476960332102 0.959418281835206 0.9904262753751103 0.9580123640319071 0.9570476131687243 0.9590980276411337 0.9570476131687243 0.959801976608187 0.9593712687082662 0.9588521296296297 0.9597279431974347 0.959396396103896 0.9885104340836013 0.9594596902654867 0.958254687141875 0.9589697665819275 0.9580137744034708 0.9588364192647743 0.956795534939759 0.9571674318403117 0.9577600685903501 0.9606095024412927 0.9586538392445877 0.9596878242774567 0.9587585113794707 0.9598108220124337 0.9591240498154981 0.9892121750232126 0.9589398770871985 1.0 0.879903187468921 0.9586015825688072 0.9898106780837769 0.959032655737705 0.9585177323680523 0.9598752539199626 0.9590613099415204 0.9591338299796519 0.9585866095811154 0.9592685054102795 0.9963663687392057 0.9599395835209366 0.959930892494929 0.9601823654682847 0.9599794036903692 0.9601990002231645 0.9604126215356656 0.9605729394876327 0.9605800220750552 0.9606024696266843 0.8777491915688367 0.9589223865199451 0.9905478471910112 0.9905556956619465 0.9590518248509858 0.9591858232473167 0.9590630187814934 0.9904373914978857 0.9601655852920642 0.9576922747306563 0.957769193351259 0.883711499500998 0.990456578243424 0.9899944395116538 0.9591895740783146 0.8835758731325005 0.8839761820448878 0.8772473702330507 0.8842511247758134 0.9593140255299748 0.9592575720164609 0.959897539555148 0.9563319462770971 0.9566048499634058 0.9428421250635486 0.9596143095183747 0.959534930971008 0.9597032890213155 0.9594343001376778 0.9594548907292385 0.9595391883790638 0.9581706395914397 0.9581572210065646 0.9581264073170732 0.9579623363348748 0.9580699390838207 0.9577182964601769 0.9578773733170135 0.9594930591200732 0.959333207893529

GCA_015685475 0.8882847572815534 0.8835168034557236 0.8782466075824716 0.8806855246461689 0.8730697190863744 0.8798021762812578 0.9116942169540231 0.8853864130694014 0.8818583227228208 0.8855581075418995 0.8906204650624712 0.8828885410628019 0.8907217114404817 0.8801879985283297 0.873108343429023 0.9104498324156093 0.8834702524271845 0.8738746887210213 0.8840342411988583 0.8740512827225131 0.8786115226754497 0.8722439195192057 0.8832156886227545 0.872352030095037 0.8832957731213874 0.8725719857969488 0.8836199760076775 0.8725063782643102 0.8849366944709247 0.8721853387392102 0.8844576484731907 0.8735460182291668 0.8832402855086372 0.8727984985461275 0.8846911630146882 0.8723956348788195 0.8845117495173745 0.8721391527196655 0.883600086809742 0.8735845951521404 0.8848420081770083 0.8722297907949792 0.8808631013916501 0.8737577251672672 0.8833342486174562 0.8736946014492755 0.8839535098227121 0.8722355971712937 0.8848719086935405 0.8737274523441525 0.8839415217391303 0.8721414543552184 0.8846893006488824 0.8724385175484546 0.8832793703615035 0.8721766780104713 0.8837581846448218 0.8733651560062401 0.8848579377524353 0.8723492638197539 0.9070113005491761 0.8721771626932148 0.8850878347784201 0.8737778784757981 0.8848112056567594 0.8737864451913134 0.876363020257827 0.8720680632514376 0.8844498964354527 0.8719354144942648 0.8760201291466195 0.8722811334552103 0.8848164061746262 0.8723397671376242 0.8836644875212017 0.872121682340648 0.8833300240384617 0.8720997806788512 0.8846630805575244 0.8737624182333249 0.8846363740731883 0.880416802383317 0.8756157995409334 0.8751526408895629 0.8834996102638587 0.8751526408895629 0.8838399180722891 0.8821395570534425 0.8831348695235814 0.8820680279990345 0.8839381355117734 0.8736700564102564 0.8850964861669425 0.8791841766169154 0.8847509381765697 0.8761030613292276 0.8829698356296833 0.873712412909836 0.8806338876570584 0.8759280030880083 0.8795754754829125 0.8846435830931797 0.8842270682730923 0.8847181919961888 0.8840702053824362 0.884749171897634 0.872793750976817 0.8839553687244776 0.880347943159923 1.0 0.8845270380237346 0.8785643227593152 0.8850604823386113 0.8837372216844145 0.884091224051539 0.8835373730043543 0.8841023799484657 0.8845160845139229 0.8837382213438736 0.8799071315136477 0.8827594385468271 0.8825119517958412 0.8808939985199804 0.8827343927221173 0.8826374457034939 0.8838878395953758 0.8829627159331608 0.8829657072252295 0.883009058602024 0.9058294597349642 0.8808246127946129 0.8780268613320079 0.8778447216699802 0.8808320231213873 0.8813618582375479 0.8807687912352516 0.8790474858269659 0.8795927929255711 0.8724642024145903 0.8725388395378691 0.9105894800000001 0.8790548190991878 0.8790373036197981 0.8810936686674671 0.9090925379310345 0.91031500833929 0.9096017030038759 0.9082575426785287 0.8872529484536082 0.887219168771527 0.8845297345559847 0.8742975753737017 0.8737427305983799 0.875337714740191 0.8813500863930885 0.8808402474771745 0.8812813290074502 0.8813198392899976 0.8807506005788713 0.880774527938343 0.875365654436638 0.874731184551581 0.8752462662506502 0.8752885235248248 0.8752057886517439 0.8740687968545217 0.8747586367197702 0.8804322536249692 0.8796342846553001

GCA_015681205 0.8770613467604952 0.9776618437640702 0.9596871229114214 0.9594289171544348 0.9575916158463386 0.9785296949152543 0.8853315806613226 0.9981461407160701 0.9781526416382254 0.9783905773822444 0.8807213454376164 0.9779119901168014 0.8806294675385332 0.9596809527042316 0.9715355547669744 0.8870521719681908 0.9776923021093219 0.9575256692160612 0.9780380013413816 0.957636175126293 0.998742554206418 0.9580005571496891 0.9775415535714287 0.9579611103065412 0.9779335952273751 0.9578579875269848 0.9775047511716136 0.9579951544401544 0.9777054389483066 0.9577471742243436 0.9793895192733717 0.9577490915645278 0.977545909801295 0.9776208313482612 0.9731570568783069 0.9581548677248677 0.9733509796550199 0.9580878422939069 0.9775428183856503 0.9575629650748395 0.973345965959328 0.9576570021464346 0.9713587441643324 0.9575038207322871 0.9775976761350929 0.9575142591710339 0.9785115960984261 0.9575615257879656 0.9983605953389832 0.95752039552061 0.9781589211618257 0.9580003538130529 0.9732114954716147 0.9581592206550322 0.9775548916685282 0.9579033484270734 0.9788648579868212 0.9575765379113018 0.9785404171240396 0.9580199021713195 0.8788376128016789 0.957993500833532 0.9981148921319798 0.957596297882465 0.9732194773980155 0.9577338096374046 0.9773702412545235 0.957975823809524 0.9730976262513905 0.9579553434751604 0.9768913706654171 0.9577050714626012 0.9732570707964603 0.95758694874851 0.9773893098021581 0.9580414586322332 0.9775465025732827 0.9576884496308645 0.9784795049288062 0.9575461360932667 0.9784337444739168 0.9588501170380375 0.9772865520489873 0.977320636426753 0.977444860548808 0.977320636426753 0.9779358154748476 0.9782769407603891 0.9775133585914866 0.9771579287953427 0.9781356741573034 0.9575016342504745 0.9785054232456141 0.9721324565701559 0.9732498916648242 0.9710455156950673 0.9774340224215246 0.9709463619227146 0.971419048723898 0.9712880133980134 0.9781438692098093 0.9731823317201935 0.9786883109919572 0.978072983277592 0.9787834652358597 0.9984097130196244 0.9579242578031928 0.9775749073867441 0.9589986468141012 0.8843452651605231 1.0 0.9587621458625527 0.9733805730211817 0.9788150168199148 0.9786066001360852 0.9777253045053204 0.9777210895883778 0.9999767655254778 0.978554831140351 0.9595323790047715 0.9780128019218169 0.9780810157273919 0.9731210121816168 0.9780140353557398 0.9778665063401838 0.9774033407821229 0.9779791412380121 0.9779470603617345 0.9779586707343648 0.8804037697993664 0.977427484139138 0.9595592928832117 0.9595624074920055 0.9774828790534619 0.9776306260907504 0.9775432661996498 0.959667841986456 0.9781119116977698 0.9774300988467874 0.9775253623529412 0.8861936018495985 0.9597268790960454 0.9595949706678699 0.977539430379747 0.8877349194130383 0.8861161243548784 0.8782039007456929 0.8864250528385352 0.9787861761499892 0.978708212803146 0.9788393711967547 0.9761603227293684 0.9717915896358544 0.9525374114832537 0.9778010488443087 0.9778522665497589 0.9779257161572052 0.9777976145787866 0.9778111781483106 0.9779875707702437 0.985737675005738 0.9857143237047226 0.9857523475469968 0.9856609047072331 0.9857534901330887 0.9856514486088755 0.9857250103187342 0.9776327549638988 0.9774678889139761

GCA_015685485 0.8755347626379266 0.9603471889838558 0.9987076124493713 0.9997791440975812 0.9992560044893379 0.9598167970426902 0.8849689362808841 0.9601871314741037 0.9590689622641511 0.9603787116279071 0.8781257160555005 0.9609353752860413 0.8781464172053705 0.9992847597254004 0.9574165494726269 0.8829901662769551 0.960339695881226 0.9978588893815119 0.9601737834664161 0.9979531417624522 0.9582839369883404 0.9993815599379572 0.9600750667291033 0.9993283142279163 0.9605983576814326 0.9989275372636262 0.9601330262541022 0.9990868170649989 0.9608830062054701 0.9989708313120177 0.9602749976348155 0.9977995501342882 0.9600920488148323 0.9589639513154659 0.9597610781544256 0.9993826201654372 0.9602137144869688 0.9994455570262518 0.9602554995287466 0.9978956516976999 0.9604602457865169 0.9988915349042062 0.9567258411677386 0.9978952795439596 0.9601893261328951 0.9977632598633459 0.9605663745019921 0.9988292737306844 0.9602694218415418 0.9977654248079033 0.9602036181943487 0.9993906490066227 0.9603377574906368 0.9994725138060524 0.9601342515811666 0.9989385902793051 0.9606603058542413 0.9981140349325669 0.9602640042323067 0.9988959197176887 0.8771900938086304 0.9989209786672532 0.9600171381267739 0.997742858712371 0.9602963063273408 0.9980488521700817 0.9585794398976982 0.9991639135206322 0.9605042924196197 0.9991412450679528 0.9583987720606513 0.9989944520246479 0.9604498850844279 0.9989372990282687 0.9602854721435315 0.9994551799514243 0.9600319623085983 0.9989641701472851 0.960237178646687 0.9978513306982874 0.9601715833135817 0.9985665312363479 0.9584250199302442 0.9585968449068473 0.9603005988165682 0.9585968449068473 0.9604492475670543 0.9603716153846155 0.9601269061549265 0.9588970111137386 0.9603633934504034 0.9978976054183963 0.9604121665490474 0.9587318522833178 0.960404984809535 0.9572845614035087 0.9601907129411765 0.9562803778274921 0.9567629921259843 0.9578712413962635 0.9597587159720551 0.959849016007533 0.958822120075047 0.9606798273045507 0.958829718771971 0.9603838179669031 0.9991180719170529 0.9602405328638498 0.9908887678890876 0.8805414684617303 0.9603234134729826 1.0 0.9605442901161415 0.9600866619217081 0.9606396426035504 0.9604720650095603 0.9600703391019646 0.9602413238827486 0.9610853944911298 0.9908013853904281 0.9600546983161833 0.9600074397942483 0.9597975770308124 0.9600002244563947 0.9599959060088848 0.9600028942973038 0.960068039673279 0.9600691085180865 0.9600702592246613 0.8772018934746235 0.959211643513068 0.9988394607218685 0.9988656673032038 0.9590811342155011 0.9592821603196993 0.959242821840433 0.9988987899301883 0.9596751764990329 0.9578238883324989 0.9578505479109332 0.8838134601634322 0.9989261642105264 0.9989200421318729 0.9593041970373853 0.8864321437422552 0.8833494790864767 0.8774489576547231 0.8835787106017192 0.9592449228971964 0.9590957713884993 0.960620221489161 0.9577904698149952 0.9569344713600809 0.9444774851689449 0.9594877600375673 0.9593037859500235 0.9597212146892655 0.9594313405456256 0.9593040462155153 0.9593281573352234 0.9582634460141272 0.9582815065392353 0.9582827078085642 0.9582944964753274 0.9582629936948298 0.9581875862068967 0.9580813501896335 0.9598678190088579 0.9596491011776017

GCA_015686355 0.8796847105694867 0.9734314035462508 0.9591232766825288 0.9606872678302091 0.957528328950514 0.971412580355136 0.8893743948891032 0.9746423835274542 0.9721940499889405 0.9736113598150484 0.8837234940554822 0.9725459618794327 0.8838766005291006 0.960308507881667 0.9909376119733925 0.8859700247831475 0.9734779641623489 0.9571855563480742 0.9736128078713501 0.9571509084389195 0.9716107499430134 0.9576972876516773 0.9731267023554605 0.9577577996637042 0.9738591132897604 0.9572086869647954 0.9731776718783465 0.9575019587133942 0.9733323003398471 0.9572162565073356 0.9746775969827586 0.9571158948119942 0.973144240085745 0.9702495684310921 0.9905113356657748 0.9577826393874134 0.9996090483299125 0.9577041807372176 0.9731940839612487 0.9572357007799575 0.9995848891786182 0.957316912878788 0.9978284131988231 0.9571463666430429 0.9732397806451615 0.9570817361440075 0.9743708185509724 0.9570895192990765 0.9743738849072513 0.9572950011823126 0.9731648117597292 0.95772312960761 0.9996035576208178 0.9578145670789726 0.9731382011149228 0.9570120418233082 0.9742652943732348 0.9573442226441966 0.9735312709883103 0.957109183529412 0.8797910807358201 0.9571306647874088 0.9739612612801679 0.957091032364753 0.9996121350398813 0.9572525479516931 0.9708742653690956 0.957197161335842 0.9914562567176519 0.9571703008225617 0.9711789862181733 0.9572840061378659 0.9998715269016698 0.9572060473372783 0.9732654007345 0.957773804089396 0.9731696433941998 0.9571382157186691 0.9734898686997037 0.9572305176081305 0.9733396365191362 0.9590653018783541 0.9712388001867414 0.9703884169611307 0.9732509202851588 0.9703884169611307 0.9737326862106406 0.9724454290756674 0.9731401028277635 0.9725140423692175 0.9738469916791125 0.9571071223191139 0.973448031329382 0.9914252725409836 0.9995952138341391 0.990764123897807 0.9730519052744887 0.9911028740068105 0.9978580480668757 0.9904270104438644 0.9722732065092826 0.9903764079273327 0.9731060965576965 0.9731441584158417 0.9730357152131573 0.9745777721943049 0.9569901843535807 0.9732198627493032 0.9597744877839691 0.8881785268990996 0.9735957968213059 0.9595362200313691 1.0 0.9733384894714224 0.9739079817905918 0.9734824381321282 0.972580280910832 0.9736631239168112 0.9747363251106894 0.960730807903403 0.97264880794702 0.9726620713218023 0.9914017657992565 0.9726809220917823 0.9726883475844378 0.9719859096916299 0.9726296350053362 0.9726170529236023 0.9726441494130202 0.8787843923749007 0.9709277405119153 0.9585482274774775 0.9585257670646542 0.9710766048565121 0.9711324285085791 0.9710342853987188 0.9589137686983701 0.9721600755148742 0.9703149183818311 0.9703930898345153 0.8864134854771785 0.9588784558659217 0.9585000958110517 0.9710415804534448 0.886237191011236 0.884845849520531 0.8788485385411278 0.8833124582223298 0.9727989980136834 0.9727233746678478 0.9747698734991425 0.9690046483825598 0.9899982579603137 0.9480999448308947 0.9714196038732396 0.9713201083351757 0.971473500110205 0.9714526508954233 0.9712268911459484 0.971356610619469 0.9715156174558961 0.971512079368763 0.971519249360911 0.9715964343786295 0.9715635810496981 0.9712433543859649 0.9715573032137867 0.9719265850340136 0.9717707345917671

GCA_015865585 0.874898375858685 0.9884950393198725 0.9572569157638736 0.9583122309711287 0.9564542474129821 0.9990637655172415 0.8850370588235293 0.9789536593059937 0.9891718102716469 0.9892425721784777 0.8795510351035104 0.9894599827400217 0.8795416602838477 0.9580935899122808 0.9691184067717995 0.8853530867469879 0.9885081983718937 0.9554795831381734 0.9888719689336692 0.9555277647058823 0.9767666073840613 0.956234932306256 0.9887553458456347 0.956268493408663 0.9894286784330608 0.9556747257876314 0.989031560718057 0.9558338164705882 0.989416259493671 0.9555691519516728 0.9909569635459817 0.9555773829588016 0.9888273800971898 0.9891906672558551 0.9712699030762748 0.9563603757088848 0.9719651802575107 0.9561722782446311 0.9888735213059149 0.9555052579227388 0.9719993367675468 0.9554923371203339 0.9697862727686701 0.9553807298424467 0.9887506990044481 0.9553271066697652 0.9901878498779495 0.9555006005586592 0.9791371221796509 0.9555062488415199 0.9886883931451614 0.9561826924871676 0.9718913057461406 0.9563159841232782 0.9887469985235182 0.9556829942196532 0.9890687492195629 0.955593495235882 0.9891037231210322 0.9556784383688601 0.87998487933635 0.9555336647398844 0.9782788579327946 0.955435079879602 0.97189067821994 0.9554089149084165 0.9879966384872079 0.9558387836583101 0.9715799596859751 0.9557644034354691 0.9882808111615835 0.9554572720962518 0.9716788664662945 0.955518108045444 0.98898584360391 0.9563312179188056 0.9888195364098222 0.9555098563484709 0.9892866068149359 0.9554357949548715 0.989192783483977 0.9579521703539824 0.9886970825168736 0.9879685169585457 0.9887811873406966 0.9879685169585457 0.989889223300971 0.9884698697972252 0.9888126856540085 0.989258518130371 0.9889033248730964 0.9553289332717617 0.9894194429708223 0.97002277992278 0.9718882234589041 0.969433436848204 0.9887556285896618 0.9691396543040293 0.9697032374263705 0.9692965570076613 0.9892356268593285 0.9713966561047741 0.9895198482643942 0.9893594226672348 0.9893297518610422 0.9792268186616002 0.9559872568636574 0.9890064247937381 0.9576306905537458 0.8838953178654292 0.9777238315513858 0.9572545996360328 0.9717664509846354 1.0 0.9888686175979983 0.9885511789833404 0.9872563503799547 0.977816735591729 0.9892555390862944 0.9583871523767215 0.9882917293851533 0.9881015760197775 0.970051238670695 0.9881731190770497 0.9883554755043228 0.9876500758054326 0.9882394250513347 0.9882559199178645 0.987949528475199 0.8784250949204722 0.9874751409618574 0.9573369911111113 0.9572909897686833 0.9873375609252374 0.9873783813245577 0.9873401259809995 0.9575906678421865 0.9884270871316515 0.9880774312733671 0.9880851801405975 0.8842227292212432 0.9576174482150728 0.9573098834139903 0.9875865716057036 0.8856457733175915 0.8844186262287221 0.8764175565151131 0.8847426924010681 0.9899099380037198 0.9898930035189402 0.9896087756796016 0.9857420499778858 0.9695598013398012 0.9500921684717207 0.9876113670990324 0.9875342601828763 0.9876914639175257 0.9877452020618557 0.9874505834717608 0.987556333402619 0.9762567606274155 0.9759933476978907 0.9762355818181818 0.976281505571981 0.9762433734939759 0.9758785091324201 0.9762388393263542 0.9886937903911565 0.9884230277185502

GCA_015959185 0.8775607413793104 0.9894946102208748 0.9593903514817368 0.9606423581479799 0.9582393740902475 0.9896991589811155 0.8845793073268319 0.9796705559232297 0.9902897460039414 0.989771050295858 0.8826658028104123 0.9902364232619152 0.8824924145101664 0.9604601686801916 0.9716773027433842 0.8846466775244299 0.9895005594405595 0.9583712933753944 0.9899153818103819 0.958458475609756 0.9774785238095238 0.9585299637155298 0.989885019404916 0.9585774498777506 0.9900442575126125 0.9584035877491494 0.9901789541918754 0.9582628369485743 0.9902935124677559 0.9584489050036258 0.9905037025043179 0.9583376818292387 0.9899275156756757 0.9897412647946159 0.9736925289145907 0.9584943947496355 0.9738663628179157 0.9584329667149057 0.989936743428199 0.9583246261456826 0.9739558069584736 0.9584831289232255 0.9715205780209325 0.9583176043425814 0.9899148373807459 0.9582565972390409 0.9907930762639245 0.9584321463178295 0.9795592921348315 0.9583492582749458 0.9892142038216561 0.9584847139753803 0.9739671422162406 0.9585773930901185 0.9898901552058632 0.958327882665379 0.9904234463638303 0.9584710375757576 0.9895998733089972 0.958303201064602 0.880025699712268 0.958281485148515 0.9796813370288249 0.9582624855491331 0.9739279064039409 0.9583667930451583 0.9892436831132732 0.9583894283646889 0.9736149546761 0.958215260115607 0.9893460715083798 0.9583463235294117 0.9739039111812444 0.9583863631972953 0.9902032071366406 0.9585212795750845 0.9898596743378203 0.9584463570566949 0.9896674086733604 0.95836132271301 0.9897478068280036 0.9604987468383537 0.9895433775877588 0.9889159365731235 0.9899021006755285 0.9889159365731235 0.9901954077819302 0.9892550219106047 0.9898743482952093 0.9903271112542956 0.9899351815038894 0.9582165503129514 0.9897015192307693 0.9727829694224235 0.97397551492035 0.9709291427889207 0.9898316525515745 0.9710946337994786 0.9714617120530429 0.9714727771184428 0.9893200528750827 0.9737354448635457 0.990695524400685 0.989921528412802 0.9907573684210526 0.979689479678428 0.958244376504574 0.990235409090909 0.9603243445945947 0.8842984107058266 0.9787177341251713 0.9600764478034955 0.9740395702592087 0.9900978905408317 1.0 0.9895566543156059 0.9901805513307984 0.9786529610868064 0.9905072566187201 0.9601341951779563 0.9891257707087959 0.9891066809238666 0.972629525439197 0.9890648963010478 0.9892557704637744 0.9886075425950196 0.9890502301790283 0.9890539812446717 0.9889949904112508 0.8772578885941644 0.9883347627340008 0.9595221079453324 0.9595281066048668 0.9884882868352223 0.9885212760416665 0.9884803531720079 0.9597812600597838 0.9896459012453572 0.9885375380009213 0.9885474097078445 0.8844913795656466 0.9598498781889222 0.9598254219360773 0.9885977452256945 0.8856369142717974 0.8847906305418721 0.877880581395349 0.8847221232534929 0.9904642878464819 0.9904488394956187 0.9911661979055353 0.9886487533875338 0.971509143753015 0.9518695728395062 0.988663193842151 0.9883912353197043 0.9887973191396914 0.9887107921653971 0.9883339813002827 0.9884343914652732 0.9775568363463367 0.9772829781680115 0.9775483269961978 0.977545695631529 0.9774919676806084 0.9771416415410384 0.9773932705601907 0.989901550591327 0.9897201386443663

GCA_015959425 0.8767586861675757 0.9999480829315333 0.9579754395604397 0.9595693847566575 0.956683164186387 0.9884286218302095 0.8842761233815689 0.9777464682451869 0.9877458242753623 0.988642176987358 0.8801507350901525 0.9987149646275489 0.879930319962903 0.9593376600644865 0.970454067755301 0.8846038109756097 0.9999507768498738 0.9566482957884427 0.9997653434225845 0.9565951818181817 0.9752771038251367 0.9563479874969946 0.9917800463939266 0.9562544091239991 0.9979833841336118 0.9558799975921021 0.9924381581178464 0.9556953189946835 0.9988862441505596 0.9561131760173368 0.9904538301141352 0.9558509180961585 0.9918147572815533 0.9890806521229182 0.9719651224354734 0.9566753535600684 0.9729309131502568 0.9563670913461537 0.991829142614601 0.9560439683680805 0.9729258474955278 0.9561850312349833 0.9703067490403071 0.9559955885878687 0.991799248518205 0.9559932297687863 0.9897420307167236 0.9560503126503126 0.9780289204417401 0.9561233325324363 0.9878413426724139 0.9563916626621816 0.972819419124218 0.9565692226233453 0.9918322679324895 0.955943459602014 0.9887559960070985 0.95618154530161 0.9883931413725915 0.9560800672107537 0.8802586520376176 0.9559768685412161 0.9771612380102609 0.9560875676971006 0.9728318139223562 0.956212102736438 0.9874159400285578 0.9564720529640428 0.9723527992799281 0.9564097816593886 0.986922195177434 0.9561402635993291 0.9728556034096008 0.9561355571565803 0.9924197628205128 0.9564236042317866 0.9918603619813716 0.9561415347721822 0.9884313533347723 0.9561012655800575 0.9882520017444396 0.9587412909217555 0.9876101856148491 0.988574945155393 0.9917157772572404 0.988574945155393 0.9982684788907286 0.9886660572687226 0.9918242905334177 0.98700875523243 0.9999043663885115 0.9560824317855433 0.988411735930736 0.9714941795166858 0.9729037349397591 0.9700257531227038 0.9916399405014876 0.9696795716678631 0.9702427698525914 0.9698511184521 0.9887990253761509 0.9720699221530249 0.9881585410199556 0.9978446599131694 0.9875966754385965 0.9778472057181148 0.9565152122527738 0.992418898771707 0.9587162979114069 0.8831373186653771 0.9768832826537662 0.9584954575866189 0.9730013962434941 0.9889720013037809 0.9886335745807592 1.0 0.9893509039429532 0.976879861175382 0.9891230913804278 0.9584377201952126 0.9877182826086955 0.9876609312445607 0.9712979003476245 0.9876806573791903 0.9877467051142547 0.9871579467680609 0.987756300477638 0.9877215367918385 0.987755426895503 0.8775434203444564 0.9872943932038836 0.9580320712954334 0.9580250559179869 0.9872864448374972 0.9873521527624917 0.9872881026094649 0.9583073712173712 0.9875034800179614 0.9878092568357093 0.9878235007006073 0.8846133994053519 0.958317973659889 0.9583069415569416 0.98736805335097 0.8843139163591637 0.8836295414462083 0.8768107214323329 0.8841200664621677 0.9881347808852676 0.9881310097216084 0.9894527155642873 0.9884183211344921 0.970057643889297 0.9496480751992032 0.9875098083278254 0.9872953554083885 0.987597362030905 0.987619538121547 0.98733600132626 0.9873068345482661 0.9757805927525798 0.9751624940334128 0.9758578516562649 0.9758844012479001 0.9757857486181206 0.9756460275295823 0.9757882684918348 0.9886065613091235 0.9884467063581217

GCA_015959505 0.8798365694117648 0.988390584602717 0.9572414528386232 0.9585806331636042 0.9563724732715609 0.9883287515736467 0.8848340145985403 0.9778805867400975 0.9886179835746706 0.988264904120767 0.8806833250283126 0.9898424364095019 0.8805166174130484 0.9583559238646484 0.9699262254786102 0.8852131652046784 0.9884316656269495 0.9562727108146736 0.9888639268940169 0.9564141786743515 0.9756208675588371 0.9561166111241493 0.9896075819842258 0.9562250367734282 0.9897899324324324 0.9558731778563015 0.9898545250467679 0.955798566019877 0.988645983151839 0.9555631628343502 0.9901531435747174 0.9556901603395426 0.9896518060757388 0.9889328574645351 0.971376766517952 0.956835095831337 0.9721354817566092 0.9560942719812429 0.9896172820191907 0.9557417708819411 0.9722059002830393 0.9556897799110279 0.9698304258747698 0.955677015169195 0.9896206964136781 0.9556128645100798 0.9895215280898877 0.955598840375587 0.9777567845448593 0.9557112692847126 0.987991617977528 0.9561670704225352 0.9720526017853256 0.9562075628081709 0.9896623268870868 0.9557932306255836 0.9890970196161147 0.955813305144468 0.9881848407775021 0.9558846458724203 0.8802139032421478 0.955694665575304 0.9772351764199657 0.9556518349265564 0.9720622752930959 0.955765218611176 0.9880368741511997 0.9563930314495153 0.9723565386266094 0.9562809174528302 0.9873657084055459 0.9555637149532712 0.9720669160305343 0.9555733684703678 0.9897950660515831 0.9562512922138836 0.9895715021906949 0.9555491378504672 0.9882092331414474 0.9556039622201494 0.9880953527575441 0.9579172109147842 0.9882031815181519 0.998600935207824 0.9895584193954661 0.998600935207824 0.9895775991561181 0.9982681447342617 0.9896667988374506 0.988389315068493 0.9887110658436215 0.9555976738776459 0.9882840518305224 0.9712857486095661 0.9721590574662605 0.9699354485828063 0.9894407154811715 0.9692985889145497 0.9698331164383561 0.969315601735556 0.9884477661169414 0.9718509299335764 0.9891780189673339 0.9892208694745622 0.988644203049927 0.9776513574366746 0.9560968373849421 0.9898623496357961 0.9581709320934256 0.8841867745664739 0.9767637392455327 0.9581076800185229 0.97202625 0.9889025186104219 0.9890878371554809 0.988464601531774 1.0 0.9768165654987726 0.9890901344469342 0.9578392321549201 0.9875764870466323 0.9875072742370771 0.9712544133333334 0.9875156713011 0.9876086211901307 0.9869518206867316 0.9874277344072696 0.9874347717413757 0.9873915888429751 0.8784774103896104 0.9869673600000002 0.9575589428312159 0.9575372051806409 0.9871365023177414 0.9872036657568759 0.9872073388116309 0.9577282272215973 0.9885176909871244 0.9882833273502355 0.9882921776183 0.8844542236467235 0.9577055828082809 0.9577899145875478 0.9871144957983193 0.8854696958981613 0.8837244010669253 0.8773153882646691 0.8844702498163115 0.9885812951432129 0.9886046433770015 0.9893268512756689 0.9982224630738523 0.9694589515749883 0.9494542676090666 0.9873061670146137 0.9872916484670308 0.9875073197820621 0.9874210130033558 0.9873692642421695 0.9873452884210526 0.9752517977788061 0.975012654805915 0.9753280972222221 0.9752841245947198 0.9752795276684418 0.9747884701492536 0.9751280459503364 0.9886742136880498 0.988481409786592

GCA_015959985 0.8771270913991669 0.9780387414500684 0.9603065771190366 0.9598048197170241 0.9581194478229142 0.9788914406585869 0.885824336372847 0.9981788688730626 0.9783708314140949 0.9784577032967033 0.8808776511954993 0.9780809359382099 0.8808033741464563 0.9599720380559377 0.9715400788718929 0.8872287227101631 0.9780648658564549 0.9579814968461912 0.9782886023933167 0.9579361461632157 0.9987059003914746 0.9584197647914647 0.9778075473826715 0.9584096943765282 0.9779851660600545 0.9582524467054263 0.9777466659137113 0.9584011062163486 0.977887283978398 0.9580398332124728 0.9796627691963288 0.9579723520818115 0.9777886598870058 0.9778571352912855 0.9731230575939516 0.9585118139761385 0.9734352597547381 0.958500189182634 0.9777928765571914 0.9579105495300073 0.9734553131268108 0.9581375761973875 0.9713651695713612 0.9578925585324644 0.9778296901153587 0.9578957855240862 0.9786818793296089 0.9579237309460442 0.9984296299447044 0.9577965250965251 0.9782807507175977 0.9584323114237207 0.973385793809842 0.958560723125455 0.977813598194131 0.9582933140236541 0.9791320082720588 0.9579302029966167 0.978624466622311 0.9583517303969022 0.879084503715499 0.9583883007009911 0.9981239324410455 0.9579195776007724 0.9733523754448399 0.9580161023907269 0.9776404147353013 0.9584087837837838 0.9731152452407615 0.9583432095490716 0.9773899833650189 0.9580165289855073 0.9733669282211324 0.9579871694464589 0.9777799432977999 0.958487668607472 0.9777870820117807 0.9580072740454327 0.9786564299933643 0.957862361312108 0.9785397056199822 0.9594668248175182 0.9776912920143027 0.9776685934940322 0.9777729041780201 0.9776685934940322 0.9780031815076293 0.9784608324034814 0.9777533678990081 0.9774543174603175 0.978379836400818 0.9578370967741937 0.9785429969012839 0.9718064284108789 0.9734732649877423 0.9709271050750536 0.9777478371040723 0.9706212565445027 0.9713530135704259 0.9712234352392065 0.9784762734768667 0.9731187533333333 0.9788841220338984 0.9780454334609321 0.9790134832730562 0.9985020991315399 0.9584465845410628 0.9778121046695242 0.9590004288888888 0.8848966000000001 0.9999879267078271 0.9592556201090823 0.973334779676259 0.9791568584371461 0.9788856808803301 0.9781049405034326 0.9779246933333334 1.0 0.9788653225448903 0.959934311100854 0.9780221264367814 0.9780252166224581 0.9730490436730124 0.9780087787102474 0.9778734645147026 0.9774281517553793 0.9779308498896248 0.9778995343191348 0.9779180335466785 0.8803824459855961 0.977792018612896 0.9601191816920942 0.9601299190938511 0.9778243650088811 0.9779745946542964 0.9778950077622532 0.9602532814288987 0.978638346764909 0.9779172414616671 0.9779358973747015 0.8866815858163014 0.9603068750000001 0.9601591678097852 0.9778309611135662 0.8880773195121952 0.8863775893743795 0.8783821311475412 0.8867872604790421 0.9790864180418042 0.9790413012792236 0.9790293595275948 0.976383515151515 0.971733604815864 0.9530418890772585 0.9781153941267389 0.9780541650033268 0.9781805661211853 0.9780958316766071 0.9780225016648169 0.9781452420071048 0.9857001044568244 0.9857244148319815 0.9857694016697589 0.9857343660009289 0.9857811363636364 0.9855833813953488 0.9857077324368189 0.9775702054325497 0.9774812371604656

GCA_016774285 0.8785061890547264 0.9890227191060472 0.9597354724866496 0.9606577637324751 0.958430611234843 0.9895851653164003 0.8835963358974359 0.9788756190686381 0.9950666696054663 0.9890903143585388 0.8819848496240601 0.9898049302634492 0.8818786756373939 0.960479630911188 0.9710566830708662 0.8851541238938053 0.9890391875000001 0.9583861615667074 0.9892556404641997 0.9584600909314327 0.9772593629664846 0.9582300291757839 0.9885755782312927 0.9583957075936884 0.9890346967340591 0.9584163958894055 0.9890231985940245 0.9584205133873741 0.989373684557853 0.9583128773815339 0.9912521673168617 0.9582022093880562 0.9887978654649375 0.9901726123399301 0.9724415246233418 0.958311214425908 0.9731790315980905 0.9582239110354887 0.988775360619469 0.9582103939688716 0.9732991786120592 0.9582771734892788 0.970053921096945 0.9582090690325717 0.988601543196307 0.9581285220355491 0.9907817631750162 0.9582473043052838 0.9793755608974359 0.9581553252131547 0.988209187741106 0.9582684830776724 0.9731312778029961 0.9583231052118852 0.9886180105286247 0.9582972108345535 0.9891634183562242 0.9582452767617654 0.9891937904100673 0.958298443684339 0.8789362311422414 0.9581902558479531 0.9791398606115108 0.958228596065096 0.9731520890798101 0.9583327314702308 0.9879576436244746 0.9581330340632604 0.9729743294329434 0.9580209572400388 0.9881311041009464 0.958158309859155 0.9732317339721651 0.9582941162109374 0.988948402039007 0.9582734022876612 0.9887251707424543 0.9582216857212359 0.9891762977346279 0.9582398297251277 0.9891848470948011 0.9605661035042934 0.9882579665234108 0.9880940955399594 0.9887073265351363 0.9880940955399594 0.989680885848845 0.9883774722161691 0.9886581329530496 0.9947037870330615 0.9893683340669014 0.9581424333494911 0.9892439605889997 0.9717395530216648 0.9731820979814017 0.9698838297362111 0.9886376697530865 0.9696634018646905 0.97001295117281 0.9712760941960038 0.9901234382620263 0.9724418932256618 0.9944858618477332 0.9893739331562639 0.9944190514847256 0.9794659085740278 0.9579646431163803 0.9890388171806168 0.959081735518635 0.8825102366578123 0.9787466811926605 0.9597540343243897 0.9733054792626727 0.9890632113910186 0.9895250555434545 0.98907939595825 0.9889625859491779 0.9787361599073002 1.0 0.9594743105849582 0.9885017563800129 0.9884427658662093 0.9718041141552511 0.9884356015440703 0.9885318334048948 0.9879493462628024 0.9885960029749257 0.9886020781980451 0.9886105633503401 0.8788082551944593 0.9882131661578718 0.9596537083137624 0.9595886876763875 0.988241009594418 0.9883043229732665 0.9882457292347939 0.9598420774073211 0.9890326674033149 0.9884712126822494 0.9884825652274302 0.8843158857574233 0.9598641114738807 0.9594378627906975 0.988340259033522 0.886082742014742 0.885625435106912 0.8787890163934425 0.8854698113688505 0.9976984925342608 0.997650508092604 0.9910648801882755 0.9879946579657966 0.9713588315151516 0.9510381872960081 0.9882694141807177 0.9883265622961515 0.9885855074983699 0.9883321133796699 0.9881336284533392 0.988375363299149 0.976494188580905 0.9763989327969712 0.9766490701545779 0.9765856682464455 0.9765277838479811 0.9763684591421041 0.9764329801956575 0.9896562842728669 0.9895077093866784

GCA_018067745 0.8760468753206773 0.9592801852720451 0.9905235924511345 0.9901081907968575 0.9899051929067818 0.9594789015151514 0.8839608398539385 0.9601960259379344 0.9588153989361702 0.9603800116631679 0.8796196202225448 0.960245 0.8793743248484849 0.9902071573261312 0.9575792848149083 0.8841847168347556 0.9592546285307382 0.9893816816674561 0.9597786502923977 0.9894745308760172 0.957897112037264 0.9897306051330351 0.9597594933457857 0.9896887407407409 0.9604263305124591 0.9894168664140217 0.9600510399061034 0.989256749762583 0.9603304911452185 0.9894837016965129 0.9604150155242417 0.9890803931785882 0.9599237172284645 0.9578874962216625 0.9592771926133707 0.9897539265267176 0.96091406052076 0.989655008233357 0.9598914373088684 0.98922714720337 0.9609463651277245 0.9894028272620446 0.9576157697090277 0.989216696282441 0.9599083970898851 0.9892516247945526 0.9605087461845504 0.9894157563619228 0.9605727476190477 0.9893156590056283 0.9598262470753394 0.9896372530063664 0.9607092808620286 0.9897910207449317 0.9598888753799392 0.9890509291412483 0.9608035035294118 0.9891709508119557 0.9602503826169106 0.9891852403393024 0.8772656605305901 0.989208594264222 0.9603450305307657 0.9892148783910197 0.9608622692217808 0.9893206966924702 0.9583678511979824 0.9893334126236457 0.9599458061465721 0.9893510865475071 0.9584783422459893 0.9895696006577402 0.9608428205128207 0.9895119054340157 0.9601607907196968 0.9896958162544169 0.9597383895921238 0.9895511716365344 0.9602103195488723 0.9892267542431994 0.9601994254510922 0.990562898714189 0.9585236550207671 0.9573740329724411 0.9600422920604916 0.9573740329724411 0.9604778515717325 0.9592971988663203 0.9598553103126459 0.958384784958872 0.9599739237826396 0.9892802569444444 0.9604073075106283 0.9591906701520913 0.9608575901639345 0.9560460201573254 0.9599948219306467 0.9572915710044534 0.9576503831891223 0.9581130308927268 0.961071701165834 0.9596236470312134 0.9598727189265536 0.9599906193853428 0.9598734644194755 0.9607017616580311 0.9895819247058824 0.960209649906015 0.997017931925894 0.880438988336714 0.9601061383558398 0.9896586886395511 0.960928136277303 0.9598530310942323 0.9604489947718631 0.9595156610009444 0.9595877036516853 0.9601608397129187 0.9605173010702653 1.0 0.9606292023121388 0.9606494796484735 0.959570903041825 0.9605829683529684 0.9596896780869164 0.9595544983435875 0.959850032154341 0.9597900758097864 0.9598675677537897 0.8773788809654416 0.959856041520877 0.9902425062146892 0.9902397693351424 0.9599980737800607 0.9601746302325581 0.9600288585434175 0.9902634185733514 0.9601432264150944 0.9581777374440578 0.9581700844510681 0.8841362534996183 0.9902931890558422 0.990330354657688 0.9601552744186046 0.8863998493827161 0.883042287247636 0.877424484897519 0.8835104243059195 0.9599589207459207 0.9598751003734828 0.9604886729411766 0.9573317383820998 0.9572759828629032 0.944874226673428 0.9602927793231341 0.9600532187938289 0.9603901791530944 0.9601351326815644 0.9600126063581114 0.9601740786701009 0.9584535040363271 0.9575701427498123 0.9583149987389659 0.9583636437042644 0.9582958707723372 0.9584061016949152 0.9583544803229062 0.9594165842534806 0.9593420024009605

GCA_019448645 0.8757341177951172 0.9875692585258525 0.9585698624067164 0.9589189704524469 0.9568317971442639 0.9884393037557655 0.8840663582626573 0.9779926839145107 0.9881943009290732 0.9990434840265221 0.8788005233380481 0.9887344336283187 0.8788213789846517 0.9586759689383402 0.9698580338733431 0.8837266357786357 0.9876028765597147 0.9564455623471882 0.9879996871581712 0.9566077460629923 0.9754128773919206 0.9566414626429787 0.9886662565445026 0.9567762010347377 0.9884494342105263 0.9565091737752863 0.9890530068086976 0.956380704467354 0.988543019366197 0.9564807613719291 0.9892935941266712 0.9564303871600097 0.9886913120489831 0.9883517215948376 0.9718495850433196 0.956734974271012 0.9719119378596088 0.9567063163017031 0.9887632036914963 0.9564185436893203 0.9719490432382705 0.9564826123936816 0.9704096809282088 0.9563666966237552 0.9886637916666667 0.9563850585080449 0.9890400886294854 0.9564911532846715 0.9778897571592211 0.9564353271710045 0.9986633670374114 0.956851408038977 0.9718384135717559 0.9567716114090686 0.9886479978142076 0.9565145715676728 0.988595930640913 0.9565251577402788 0.9989284325762227 0.9564615217391306 0.8779397289238277 0.9563430260277305 0.9781435772541913 0.9563719781553398 0.9718630422857143 0.9563343888213852 0.9873981242561296 0.9564493563274228 0.9716946054127814 0.9563968436363637 0.9870974459367174 0.9564564667314231 0.9719334750632039 0.9564587542579076 0.989017434705622 0.9566993429058165 0.9886297741228072 0.9564886154219205 0.9989802298616762 0.9564109238998297 0.9989002307850814 0.9590412197955391 0.9875177067583049 0.9874509057639523 0.9886863246351172 0.9874509057639523 0.9888479840496234 0.987865272767268 0.9886982139737991 0.9880225340659341 0.9879246280809859 0.9563502325018165 0.9991147646336472 0.9707204945054945 0.9719406819747417 0.9699420262390672 0.9887195654082528 0.9698744189930804 0.9703279042348412 0.9704933920914721 0.9897044227786396 0.9719691572012722 0.9883757833698031 0.9887489984659216 0.9883176202974628 0.9778332059158135 0.9563427299199225 0.9891281728232191 0.9595119487525289 0.8821629223300971 0.9770904290578888 0.9574637806637809 0.9720080404463041 0.9880946741523559 0.9880943613017489 0.987642340142096 0.9880283524572419 0.9770720838103961 0.9882865013951492 0.9592130817174515 1.0 0.9999902755676305 0.9716331347746089 0.9999937640667443 0.9996950068053666 0.9992636495913892 0.9999451763565893 0.9999559728682171 0.9999415875169606 0.876936609477124 0.9984738461538462 0.9582659463243876 0.9582515977575332 0.9985718506493507 0.9985924322136787 0.9985777741673437 0.9584597337346609 0.9879196449178873 0.9880832466940264 0.9880962069751539 0.8844014372623573 0.958512198565147 0.9582350196440952 0.9984799676244437 0.8849356461576477 0.8837640116869919 0.8777322418801161 0.8837990275986589 0.988367006313956 0.9883027633587788 0.9890262602164788 0.9872160577797999 0.9698085311284047 0.9494757832537087 0.9987991126418154 0.9985546188340807 0.9988505555555557 0.9988033387489847 0.9985835608066818 0.998596324489796 0.9769501101268853 0.9762799524601854 0.9770383361264063 0.9770046484935437 0.9770333125748504 0.9765365302191187 0.9769059798754193 0.9887233237695873 0.9884309105006647

GCA_019449055 0.8752250568038374 0.9874973952359946 0.9585551186598417 0.9586359496884376 0.9566956485355648 0.9881987362878454 0.8836011950655358 0.9775540274034141 0.9876911214108073 0.998793264321608 0.8786308402563493 0.9886834085379341 0.8785592082738946 0.9585129543875897 0.9699253026219065 0.8832883200206133 0.9874605230358335 0.9563606722072844 0.9878108573928259 0.9566433801428924 0.9752201201130476 0.9566862503043584 0.9883990775956284 0.9567065314650934 0.9885101687486302 0.9566203493769853 0.988750354001759 0.9563389901477832 0.988551178933451 0.9563759295261239 0.9892716512955643 0.9565676638943249 0.9884571444201312 0.988216433194637 0.9715769711429221 0.9567841631952952 0.9715781007128075 0.9566955404089579 0.9884112104569419 0.9563334997576347 0.9716047326141842 0.956384690759156 0.9701393023255814 0.9563319466666667 0.9883981009879252 0.9562912244401168 0.9888446275102569 0.9564165119105494 0.9774280086976426 0.9563292987139045 0.9985665439838219 0.9567254755533934 0.9715941282932417 0.9568414961013646 0.988383086176728 0.9563858546073425 0.9880221516483517 0.9562502339181288 0.9987279537074969 0.9564233024841695 0.8776700324939073 0.9563798153547134 0.9777635728859668 0.9562851477713179 0.9716165257142856 0.9561871598350716 0.9869647109207711 0.9563708907766991 0.9712463700340522 0.9563661588762413 0.9870088673655425 0.956352565470417 0.9716135008066376 0.9563789064398541 0.988765661340435 0.9567245379377433 0.9883641835387326 0.9564066295129634 0.9988307491856678 0.9563248168809119 0.9986754177841963 0.9589329137691237 0.9871826837645981 0.9875076161061543 0.9883797504416961 0.9875076161061543 0.9887275569816332 0.9879250913493287 0.9883982473776223 0.987566573810046 0.9878785471781306 0.9562716210984756 0.9989237530712533 0.9704802351598174 0.9715677752293579 0.969634686060606 0.988439385830226 0.969773224421655 0.9700833229216723 0.9705092071428572 0.9896334303488113 0.9717364421768707 0.9881602823374919 0.9886590548245614 0.9881993942707195 0.9775505626423691 0.9562818593939395 0.9888429392338176 0.9594228163632278 0.881694558071585 0.9765731764438382 0.9573836757276882 0.9717008724988366 0.9880256291390729 0.987877378210806 0.9875153092440699 0.9879027865655472 0.9765129449541284 0.9878905106200386 0.9591636088663126 0.999972495156916 1.0 0.9713525734618916 0.9999768739105172 0.9995748321366194 0.9992953386454184 0.9999790643161565 0.9999885957776486 0.9999751617276776 0.8768396427597491 0.9983366166328601 0.9581866004672897 0.9581622614593077 0.9984974761614932 0.9985070159821972 0.998504419407227 0.9585031794990724 0.9877284076291861 0.9877987853236099 0.9878391340018232 0.8837080384712731 0.95850421918443 0.958222142526608 0.9983772620731461 0.8845439321110011 0.8829279878203501 0.8772173150105708 0.8835899742665981 0.988112699586687 0.9879840610021786 0.9887970342541437 0.9871161249999999 0.9698027526724975 0.9489516813048934 0.9987339442311579 0.9983980573637103 0.9987027383120826 0.9987440219155844 0.998353865392436 0.9983798412052118 0.9765305969436484 0.9759296015180265 0.9766359020310634 0.9766127105702697 0.9765982788254952 0.9761331051873198 0.9764550597514341 0.9885836831639417 0.9883019725177304

GCA_019449085 0.8738929020891015 0.9713468688754084 0.9591987952646239 0.9591315220874342 0.9575267689663639 0.9707382047718323 0.8830942823651994 0.972728717716357 0.9716069108354787 0.9720527046750286 0.8756599636010678 0.9722753891805525 0.8756373971268566 0.958993337918386 0.9914807824471791 0.8841802666666666 0.9713704803390628 0.9569975906546606 0.9715604608615102 0.957051952894995 0.970059550454328 0.9574940624237991 0.9703922749031229 0.9574956910969011 0.9720835789473685 0.9570043166869671 0.9703723540145985 0.9573483198430219 0.9715870633484163 0.9572383649635036 0.9725462450592887 0.9569147315518511 0.9704524777549624 0.9698277131226054 0.989320330964016 0.957642244248654 0.9911949955927721 0.9574994633317144 0.9704824409809765 0.9569789840348331 0.9912316893972722 0.957202269864341 0.990619967630058 0.9569418529269473 0.9704885279304029 0.9567943040737149 0.9724491595077781 0.9570612363371387 0.9727420124195032 0.9568878988138464 0.9714239421579534 0.9575797404802329 0.9911951605807303 0.9576447481138962 0.9704388615103811 0.9570796522579087 0.9721078286247958 0.9569533114514952 0.9718686513233603 0.9572679447406689 0.8756698377062485 0.9572063911046652 0.9727063321084061 0.9569159821212854 0.9912521326910445 0.9571089723703345 0.9694949415568267 0.9572761313692344 0.9907391578473318 0.9571602871621622 0.9700983454633674 0.9571018520309478 0.9910501361440492 0.9571532532751091 0.9705582113259668 0.9575511005830906 0.9705058673703534 0.9571792931493586 0.9718442785273268 0.9568555964190659 0.9718275862867732 0.9590070081490104 0.969752918579367 0.9705623275047258 0.9704560272077473 0.9705623275047258 0.9719839183481933 0.9719833069441302 0.9703366848691696 0.9717331409671534 0.9718842943783531 0.9568721418234444 0.9719218563561266 0.9902363663157894 0.9911890937019969 0.9901299206534424 0.9703529828178694 0.989496400281228 0.9907351577250748 0.9905738502061382 0.9724131011608624 0.9892830332594237 0.9718152142038946 0.9716562937384899 0.9718158777752345 0.9727687073896135 0.9562476411241895 0.97041398813598 0.9595021539850983 0.8803297774405667 0.9726561238532111 0.9583463245426467 0.9910056373309688 0.9716752204015693 0.9718994698569082 0.9714645206766918 0.9716335399449036 0.9726720064874884 0.9719930711695376 0.9589610300734074 0.9721582751540041 0.972168431864871 1.0 0.9721339986313868 0.9720781469648562 0.9716577816736793 0.9721691666666666 0.9721472683815161 0.9721283914816492 0.8769377863224389 0.9711100900692841 0.9589859026651216 0.9589536138154846 0.9710547367210852 0.9711809988545247 0.9710768061623363 0.9593311853893866 0.9721434785697372 0.9693675781818182 0.9693872297624817 0.8836282388973966 0.9592753779002987 0.9582939443049533 0.9713453232114101 0.8860826998528691 0.8838284934998726 0.8754814289595219 0.8853700906031583 0.9721968599587442 0.9721836687342063 0.9730293099415205 0.9697562488174077 0.9900505875440659 0.9469723398826231 0.9715216887568557 0.9715475270799724 0.9717318698441798 0.971585246051728 0.9711409426135056 0.9715263732963734 0.9712029070596626 0.9710840218164571 0.9712285693916348 0.9712053756538279 0.9712404803804994 0.9707983937947495 0.9710597384688541 0.9721398257679964 0.9720950022956841

GCA_019449135 0.8758540010078104 0.9875108875219684 0.9586405276615528 0.9589956837016576 0.9569812853280903 0.9882086624343258 0.8835417668115573 0.9776822738496072 0.9881438353686116 0.998808921470175 0.878885394239849 0.9885935559964726 0.878830532292406 0.9587694173410404 0.969505826001955 0.883586307415961 0.9874892415480426 0.9566750903761603 0.9878282010466639 0.9567894964382216 0.9752504432916764 0.9567717963052991 0.9884661158284347 0.9569151072749692 0.9884203061447628 0.9567509449585971 0.9887596276828734 0.9566719032416504 0.9885446815985947 0.9566909839650145 0.9893555436212188 0.9565901733821734 0.9884461798733899 0.9882430969976905 0.9717080574555403 0.9569348603625676 0.9718686361545538 0.9568549793338196 0.988520798245614 0.9565471023278371 0.9719013633233877 0.9566874405628335 0.9703035897435897 0.9564846608527132 0.9884617968920991 0.956437143898882 0.9890020579334197 0.9566203497692495 0.9776772224760164 0.9565177165736473 0.9985660323559151 0.956976952288218 0.9718682184566063 0.9569391344517384 0.9884513711790394 0.9566235724708172 0.9883446479491117 0.9565708097560977 0.9988080934809348 0.9566562588349987 0.8778265740490963 0.9565194987834549 0.9778683985523638 0.956536000484496 0.9718789533820841 0.9565625539655592 0.9873520241763452 0.9565943255701116 0.9716189272727274 0.9565302978208233 0.9871680481708702 0.9565981813773036 0.971902552946593 0.9565445770539621 0.9889107348384241 0.9568116123540857 0.9884397236842106 0.9566296875756842 0.9988629792429794 0.9564943425521591 0.9987803424798846 0.9591235379981465 0.987530416 0.9876502672453176 0.988432414553473 0.9876502672453176 0.9887375447513813 0.9880780202153373 0.9884532912219559 0.9880810085507565 0.9878403933201494 0.9563167133204633 0.9989402666666667 0.9707047156885134 0.9719292108281715 0.9699691434793128 0.9885043636363636 0.9696854329210277 0.970253906887449 0.97034596912114 0.989499559929235 0.9719337877069631 0.9884300065588107 0.988639333187582 0.9883930224939944 0.9777672241183162 0.9564414237288137 0.9888636788930376 0.9594714957456337 0.8818691129423171 0.9768038276643991 0.9575588782128271 0.9720046890951277 0.9880383604753522 0.9880805562943262 0.9875776355988488 0.9881147156756757 0.976726455232425 0.9878423704496788 0.959100458631021 0.9999937613790433 0.9999714233152596 0.971212116838488 1.0 0.9995499243746365 0.9990866408114557 0.9999487425033856 0.999959880054169 0.9999445560069647 0.8767982944685467 0.9983671776155718 0.9584925011665888 0.9584976248250118 0.9984625375253552 0.998480515568136 0.9984692838303916 0.9587359402501159 0.987931941189476 0.9877917875824426 0.9878067295454546 0.8844736638909918 0.9587594774566474 0.9585041537751096 0.998397405536472 0.8848488565795958 0.8837816510508991 0.8772847513812155 0.8837023657847419 0.9881423885142483 0.9880778857890148 0.9889314042835063 0.9873480939414567 0.9698351054545455 0.9494395437452997 0.9986997004654928 0.9983689833265557 0.9987415953780661 0.998702734279919 0.9983802767602766 0.9984167956814015 0.9767828301886792 0.9761003696682465 0.9768305461483425 0.9768023392091472 0.9767850083552161 0.9763197071531446 0.9766666547277937 0.9885904274069178 0.9883013436464089
[truncated: 143,382 more chars]
